# Supplementary material for: End-of-life care for people with severe mental illness: mixed methods systematic review and thematic synthesis of published case studies (the MENLOC study)
Source: BMJ Open. 2022 Feb 22;12(2):e053223. doi: 10.1136/bmjopen-2021-053223 (PMC8867317; doi:10.1136/bmjopen-2021-053223)
Supplement: Supplementary data [file bmjopen-2021-053223supp001.pdf]

National Institute for Health Research (NIHR)
Health Services and Delivery Research (HS&DR) Programme

Protocol

Project title:	End of life care for people with severe mental illness: an evidence synthesis (the MENLOC study)
Project reference:	HS&DR Project 17/100/15
Version:	1.0
Date:	24 September 2018
Chief investigator:	Professor Ben Hannigan
Research team:	Dr Sally Anstey, Dr Paul Gill, Deborah Edwards, Mala Mann, Professor Michael Coffey, Alan Meudell, Roger Pratt
REC approval:	Not applicable
Start date:	1 November 2018
End date:	31 January 2020

Funding acknowledgement: This project is funded by the NIHR HS&DR Programme (ref 17/100/15)

Department of Health and Social Care disclaimer: The views expressed are those of the authors and not necessarily those of the NHS, the NIHR or the Department of Health and Social Care

Protocol version control:

Version	Date	Details
1.0	24.09.2018	Protocol reflecting full project description approved by HS&DR Board

HS&DR Project 17/100/15: End of life care for people with severe mental illness: an evidence synthesis (the MENLOC study)

FULL TITLE OF PROJECT

End of life care for people with severe mental illness: an evidence synthesis (the MENLOC study)

SUMMARY OF RESEARCH

The aim of this project is to synthesise relevant research and other appropriate evidence relating to the organisation, provision and receipt of end of life care for people with severe mental illness (including schizophrenia, bipolar disorder and other psychoses, major depression and personality disorder) who have an additional diagnosis of advanced, incurable, cancer and/or end-stage lung, heart, renal or liver failure and who are likely to die within the next 12 months. Outputs from the project will be tailored to stakeholders, and clear implications will be drawn for the future commissioning, organisation, management and provision of clinical care. Recommendations will be made for future data-generating studies designed to inform service and practice improvements, guidance and policy.

In this context, summary objectives are to:

1. locate, appraise and synthesise relevant research;
2. locate and synthesise policy, guidance, case reports and other grey and non-research literature;
3. produce outputs with clear implications for service commissioning, organisation and provision;
4. make recommendations for future research designed to inform service improvements, guidance and policy.

This review will be conducted according to the guidance developed by the Centre for Reviews and Dissemination (CRD) and will be reported following the Preferred Reporting Items for Systematic Reviews and Meta-Analyses (PRISMA) statement recommendations. Reflecting Evidence for Policy and Practice Information (EPPI) Centre principles, opportunities will also be embedded into the project to maximise stakeholder engagement for the purposes of both shaping its focus and maximising its reach and impact.

Searches will be developed initially using Medical Subject Headings (MeSH) and text words across health, social care and psychology databases from their inception. In consultation with a stakeholder advisory group, supplementary methods will be developed to identify additional material including policies, reports, expert opinion pieces and case studies. All English language items relating to the provision and receipt of end of life care for people with severe mental illness and an additional diagnosis of advanced, incurable, cancer and/or end-stage lung, heart, renal or liver failure will be included. All included citations will be assessed for quality using tools developed by the Critical Appraisal Skills Programme (CASP), or alternatives as necessary if suitable CASP tools are not available. Data will be extracted into tables, and subjected to meta-analyses where possible or thematic synthesis with help from NVivo. Strength of synthesised findings will be reported where possible using GRADE and CerQual.

Information derived from the processes described above will be drawn on in an accessibly written summary. Uniquely, this synthesis will comprehensively bring together evidence on factors facilitating and hindering high-quality end of life care for people with severe mental illness, who have an additional diagnosis of advanced, incurable, cancer and/or end-stage lung, heart, renal or liver failure, and evidence relating to services, processes, interventions, views and experiences. Implications will be stated for the improvement of relevant NHS and third sector care and recommendations will be made for future research.

BACKGROUND AND RATIONALE

We plan a rigorous synthesis of research and other evidence conducted according to internationally agreed quality standards. The project is within the HS&DR Programme's remit in addressing quality, organisation and access in health services. In the context of calls for parity of esteem between mental health and physical health care (Mental Health Taskforce, 2016) the health problem this project addresses is a highly relevant, but also neglected, one.

Coffey M, *et al. BMJ Open* 2022; 12:e053223. doi: 10.1136/bmjopen-2021-053223

HS&DR Project 17/100/15: End of life care for people with severe mental illness: an evidence synthesis (the MENLOC study)

In preparing this proposal an initial scoping review was undertaken (with updated searches run in July 2018), and a targeted search was made for relevant policy documents across the four UK nations. A search of the database of NIHR projects was conducted to check for overlapping or related studies. Searches of four databases (combining 'palliative care', 'mental health' and 'service provision' terms: see 'Search strategy' below) produced 4,754 citations, contained within which are numbers of relevant papers including two previous literature reviews (from the UK and Canada respectively), both now out-of-date having been published a decade ago (Ellison, 2008, Woods et al., 2008). Items discovered in this scoping search confirm the timeliness and feasibility of a new, rigorous, evidence synthesis: and particularly an EPPI-Centre style review which is sensitive to the needs of stakeholders and which includes grey and non-research materials (Gough et al., 2017). Items from this initial search, combined with general material addressing what is known about the burden of disease and the physical health of people living with mental health difficulties, have been used to inform this background and rationale (and subsequent) sections of this project proposal.

Burden of disease and costs

The overarching background for this project includes what is already known about the burden of severe mental illness, cancer and end-stage lung, heart, renal or liver failure. Mental ill-health is a leading cause of years lived with disability (YLD) around the world, with major depressive disorder the leading cause of YLD in 56 countries and the second leading cause in a further 56 (Global Burden of Disease Study 2013 Collaborators, 2015). Specific cancers, along with mental health, neurological and drug use disorders and specific organ diseases all feature in the leading 20 causes of disability adjusted life years (DALYs) in England for 2013 (Newton et al., 2015). The wider economic costs of mental illness in England were recently estimated at £105.2 billion each year (Centre for Mental Health, 2010). This figure combines the direct costs of services, lost productivity at work and reduced quality of life with the annual costs of the same in Wales estimated at £7.2 billion (Friedli and Parsonage, 2009).

Meeting the physical health needs of people living with severe mental illness

The term 'severe [or, often used interchangeably, 'serious' or 'serious and enduring'] mental illness' (SMI) as used throughout this project proposal has longstanding currency within the fields of mental health policy, services and practice dating back at least as far as the publication of *Building Bridges* (Department of Health, 1995). It continues to be used in research (see for example: Kronenberg et al., 2017), and has currency with the NIHR Dissemination Centre which published a Themed Review into Severe Mental Illness in 2018 (National Institute for Health Research, 2017). *Building Bridges* recognised the imprecision of the term 'severe mental illness', and endorsed a multidimensional framework definition encompassing five areas: safety; need for informal or formal care; disability; diagnosis; and duration. Diagnosis is therefore an important, but not the only, dimension used in the identification of people with severe mental illness and includes ICD-10 diagnoses of schizophrenia, schizotypal and delusional disorders, bipolar affective disorder, and disorders of adult personality and behaviour (World Health Organization, 1992) along with similar DSM-5 diagnoses including schizophrenia spectrum and other psychotic disorders, bipolar and related disorders, and major depressive disorder (American Psychiatric Association, 2014).

People with severe mental illness have high co-morbidities (Ellison, 2008), and experience higher mortality rates and reduced life expectancy compared to the general population (Taylor et al., 2012, Thornicroft, 2013). Higher mortality and morbidity rates for this group have been found across all age groups (Picot et al., 2015), with a 10-20 year reduction in life expectancy reported (Chesney et al., 2014). Inequities can be explained with reference to individual and system-level factors. People with severe mental illness are less likely to attend health screenings and may respond to symptoms differently (Disability Rights Commission, 2006, Carney et al., 2006). They may delay or avoid help, and are more likely to exhibit noncompliant and disruptive behaviours (Woods et al., 2008, Millman et al., 2016) putting them at risk of delayed disease detection (Ellison, 2008). Inadequate support systems are also common among those with severe mental illness, affecting their ability to access appropriate clinical care and navigate complex health systems (Knapik and Graor, 2013). Other factors influencing variations in mortality and morbidity for people living with severe mental illness include poor previous experiences of seeking help from health care professionals, the incorrect

attribution of physical symptoms to psychiatric disorder by workers and lack of experience by mental health professionals in determining how and when to refer onwards to other appropriate services (Woods et al., 2008, Shalev et al., 2017).

End of life care for people with severe mental illness

In this study end of life care is used to refer to the care of people with diagnoses of advanced, incurable, cancer and/or end-stage lung, heart, renal or liver failure and who are likely to die within the next 12 months. It includes care provided in hospitals, hospices and other institutional settings (such as prisons and hostels) and care provided in the home and via outreach to people who may also be homeless.

Beyond the inequities identified above, commitments to parity of esteem demand that end of life care for people with severe illness should be as timely and as high-quality as it is for others. However, evidence suggests that this group is poorly served, with England's Cancer Strategy 2015-2020 (Independent Cancer Taskforce, 2015) recognising people with severe mental illness as needing a targeted focus to improve diagnosis and care. Although cancer incidence amongst people living with chronic mental health difficulties is similar to that of the general population, mortality rates are double (Howard et al., 2010, Batty et al., 2012). This disparity may be related in part to late presentation and reduced use of interventions such as surgery, chemotherapy or radiotherapy (Desai et al., 1999, Irwin et al., 2014). The experience of severe mental illness can delay detection and treatment of life-threatening physical disorders as people are less likely to seek treatment, to verbalise pain and to access timely healthcare (Foti, 2012). Consequently, this patient cohort is more likely to present with more advanced cancers which are invariably more complex and costly to treat, with patients less likely to undergo invasive treatments and more likely to die (Ishikawa et al., 2016). Some cancers, other terminal conditions and/or related treatments may also compound mental illness and precipitate potential 'problematic' behaviours (McCormack and Sharp, 2006). For many patients, palliative care is therefore often the only meaningful treatment option available.

Once in touch with end of life services the symptoms of people with severe mental illness may be poorly recognised and undertreated, with staff working in end of life services lacking knowledge, training and experience in this area (Addington-Hall, 2000). Undetected and hence untreated mental illness can jeopardise treatment outcomes, reduce patient satisfaction and increase health care costs (Zabora et al., 2000). Variable adherence can be a complicating factor (Knapik and Graor, 2013), compounded by comorbid disorders such as substance misuse and social factors such as homelessness, isolation or lack of transportation all of which can exert an impact on care planning for end of life care and treatment (Woods et al., 2008, Picot et al., 2015). Assumptions about the capacity of people with severe mental illness to make end of life decisions, and concerns that end of life discussions would be too distressing or exacerbate mental health problems, may lead to inadequate consultation (Morgan, 2016). This is reflected in the fact that people with severe mental illness have a higher percentage of 'do not resuscitate' orders than other groups and are less likely to have had discussions about their explicit wishes for end of life care (Warren et al., 2014).

Palliative care

Whilst not all people at the end of life need palliative care an NAO survey (Dixon et al., 2015) confirmed inequities in palliative care provision for both cancer and non-cancer patient populations. In Wales, an estimated 24,000 of the 32,000 people dying would benefit from palliative care but over a quarter do not have access to it (Hughes-Hallet et al., 2011, Dixon et al., 2015). Access to palliative care services for people who die from cancer is 46% compared to 5% from those dying from other conditions (Marie Curie and the Bevan Foundation, 2014), including end-stage lung, heart, renal or liver failure.

People with mental illness and advanced incurable cancer and/or lung, heart, renal or liver failure face inequities and discrimination (Hughes-Hallet et al., 2011) and a lack of integrated care (Ellison, 2008). Some may be excluded from end of life care planning (Woods et al., 2008), and even from hospitals or hospices entirely (McCormack and Sharp, 2006). They may be referred back to mental health services, where staff are largely inadequately prepared to provide appropriate end of life care (Woods

et al., 2008). Difficulties accessing appropriate services mean that for people with mental illness and life-threatening disease palliative care may be the first line of treatment (Ellison, 2008). The specific provision of palliative care for people with severe mental illness is known to be poorer than for the general population. People with severe mental illness are approximately 50% less likely to access appropriate palliative care, including symptom control and pain relief (Woods et al., 2008, Shalev et al., 2017). Palliative care and hospice staff often feel unskilled (Shalev et al., 2017), and lack confidence and training in conducting discussions about end-of-life care with people severe mental illness (Morgan, 2016). Existing evidence also indicates that there is a lack of coordinated end of life care and access to appropriate psychosocial support is often limited (Ellison, 2008, Woods et al., 2008). Medical and nursing staff working in hospices have also been shown to be unprepared for working with people with severe mental illness, basing their assessments on instinct rather than using evidence-based approaches (Hackett and Gaitan, 2007). In a survey of psychological services in hospices in the UK and Republic of Ireland only 30% of hospices had access to a psychiatrist, whilst 41% had access to a clinical psychologist and 45% had neither (Price et al., 2006). Patient experience data underscores these observations, with England's National Cancer Patient Experience Survey showing that people with a long-term mental health condition (2% of those surveyed, n=1184) reporting less positive experiences of cancer care (Department of Health, 2010). In Wales, the most recent National Cancer Patient Experience Survey found that the lowest proportion of respondents reporting positive experiences of their cancer care were those identifying as also having mental health problems (Picker Institute Europe, 2017).

EVIDENCE EXPLAINING WHY THIS RESEARCH IS NEEDED NOW

Research at the interface of physical and mental health is recognised as a UK priority (Department of Health, 2017). Against the background presented above, this project will create generalisable knowledge to improve end of life care and services for an underserved group. Policies from the four UK governments focus on improving end of life care, where diagnosis is immaterial (Department of Health, 2016, Northern Ireland Executive, 2010, Scottish Government, 2008b, Scottish Government, 2008a, Scottish Government, 2009, Welsh Assembly Government, 2005, All Wales Palliative Care Planning Group, 2008, All Wales Palliative Care Planning Group, 2009, Welsh Government, 2013). These require the introduction of palliative and supportive care earlier in the illness trajectory, with patient surveys showing that this is rated very highly by those receiving it (Drakeford, 2014). Marie Curie identified triggers that should initiate palliative care for people with diagnoses other than cancer (Marie Curie, 2015), but apart from dementia they do not mention those with pre-existing severe mental illness. In national policy the particular needs of people with severe mental illness who develop advanced incurable cancer and/or end-stage lung, heart, renal or liver failure are acknowledged poorly, or not at all. This group face the prospect of 'disadvantaged dying' (Exley, 2004, Payne, 2011), at a time when quality of care in the last months of life should be uniformly high for all groups.

Uniquely amongst the cancer, palliative and end of life strategies developed across the four countries of the UK, England's Cancer Strategy 2015-2020 makes the specific recommendation that the NIHR commission research in the area of cancer care for people living with severe mental illness (Independent Cancer Taskforce, 2015). This project responds to this call, and expands it to also cover end of life care for people living with severe mental illness and facing end-stage lung, heart, renal or liver failure. It will answer a question which is both timely and relevant: what evidence is there relating to the organisation, provision and receipt of care for people with severe mental illness who have an additional diagnosis of advanced, incurable, cancer and/or end-stage lung, heart, renal or liver failure and who are likely to die within the next 12 months?

Our rigorously conducted evidence synthesis will bring together reports of approaches to service organisation, processes and interventions shown to both facilitate and hinder the provision of high-quality, accessible, equitable and acceptable end of life care to people with severe mental illness. We will also gather research and other evidence reporting the views and experiences of service users, families and health and social care staff. The project will have an impact on services and practice by presenting its findings in accessible ways to NHS and other managers, practitioners and educators. We anticipate findings will inform future NICE guidelines, and thereby help shape the provision of services. Current, relevant, NICE guidance (National Institute for Clinical Excellence, 2004, National Institute for Health and Care Excellence, 2009, National Institute for Health and Care Excellence, 2011, National Institute for Health and Care Excellence, 2014b, National Institute for Health and Care

Excellence, 2014a) lacks standards or recommendations particularly addressing end of life care for people with severe mental illness, with documents supporting in-progress guideline development (National Institute for Health and Care Excellence, 2018 [anticipated]) also lacking a focus in this area. We anticipate findings from this study will help redress this, informing future guidance and thereby shaping the provision of services.

A search of NIHR databases finds studies which have investigated: the physical health of people with severe mental illness (e.g., HTA 12/28/05); services for this group across organisations (e.g., HS&DR 11/1023/13); and care for people experiencing mental health difficulties after receiving cancer diagnosis (e.g., HTA 09/33/02). As no research has been commissioned in the area we propose here, this project effectively begins a new and important field of work with value to the NHS and its partners. Using a methodical, systematic and transparent approach our team plans to use this study as the starting point for a programme of research which builds on the existing literature, is designed with people who have experience of mental health difficulties and with people who have lived with cancer and have cared for family at the end of life, and which generates new evidence of what works with value to the NHS and other relevant organisations. Further studies planned include data generating investigations into the provision and receipt of end of life care for people with severe mental illness.

AIMS AND OBJECTIVES

The aim of this project is to synthesise relevant research and other appropriate evidence relating to the organisation, provision and receipt of end of life care for people with severe mental illness (including schizophrenia, bipolar disorder and other psychoses, major depression and personality disorder). Specifically, it will answer the question, ‘what evidence is there relating to the organisation, provision and receipt of care for people with severe mental illness who have an additional diagnosis of advanced, incurable, cancer and/or end-stage lung, heart, renal or liver failure and who are likely to die within the next 12 months?

Detailed objectives are to:

1. use internationally recognised, transparent, literature review approaches to locate, appraise and synthesise the relevant research evidence relating to the organisation, provision and receipt of care in the expected last year of life for people with severe mental illness who have additional diagnoses of advanced, incurable, cancer and/or end-stage lung, heart, renal or liver failure and who are likely to die within the next 12 months;
2. locate and synthesise policy, guidance, case reports and other grey and non-research literature relating to the organisation, provision and receipt of care in the expected last year of life for people with severe mental illness who have additional diagnoses of advanced, incurable, cancer and/or end-stage lung, heart, renal or liver failure and who are likely to die within the next 12 months;
3. produce outputs with clear implications for service commissioning, organisation and provision;
4. make recommendations for future research designed to inform service improvements, guidance and policy.

RESEARCH PLAN/METHODS

Quality and standards

The protocol for this evidence synthesis will be registered with the International Prospective Register of Systematic Reviews (PROSPERO) (PROSPERO: International prospective register of systematic reviews, undated). It will be conducted with the involvement of colleagues from the Cardiff Specialist Unit for Review Evidence (SURE). It will follow guidance for undertaking reviews in health care published by the Centre for Reviews and Dissemination (CRD) (2009) and will use methods informed by the Evidence for Policy and Practice Information (EPPI) Centre (Gough et al., 2017). To ensure rigour the review will be reported following the Preferred Reporting Items for Systematic Reviews and Meta-Analyses (PRISMA) statement (Moher et al., 2009). Findings will be accessibly presented, with the help of a stakeholder advisory group and the NIHR Dissemination Centre, to ensure reach. Factors facilitating and hindering high-quality end of life care for people with severe mental illness will

be identified, along with evidence relating to services, processes, interventions, views and experiences.

Search strategy

The search strategy will be comprehensive and designed to ensure that all relevant literature is obtained. While some terminology is equivocal in this area, the search strategy will be designed to identify the relevant evidence relating to end of life care (i.e., in the last year of life) in those with severe mental illness. Reflecting the importance of diagnosis in the framework definition of severe mental illness, diagnostic terms to be included in the search strategy include: schizophrenia, schizophrenia spectrum and other psychotic disorders, schizotypal and delusional disorders, bipolar affective disorder, bipolar and related disorders, major depressive disorder and disorders of adult personality and behaviour. Reflecting prevailing definitions of severe mental illness (for example, as used by the NIHR Dissemination Centre) searches will not be made for studies into mental and behavioural disorders due to psychoactive substance use or for studies into dementia or other neurodegenerative diseases, and items in these areas will not be included in the review except where participants' diagnoses coexist with the disorders included above. To ensure appropriateness of evidence, inclusion/exclusion criteria have been developed (Table 1):

Table 1: Inclusion/exclusion criteria

Inclusion	Exclusion
Relevant evidence specifically relating to adult participants (>18 years of age) with severe mental illness (including schizophrenia, schizophrenia spectrum and other psychotic disorders, schizotypal and delusional disorders, bipolar affective disorder, bipolar and related disorders, major depressive disorder and disorders of adult personality and behaviour) who have an additional diagnosis of advanced, incurable, cancer and/or end-stage lung, heart, renal or liver failure and who are likely to die within the next 12 months.<br><br>Published in English Language only.	Where reporting allows the distinction to be made, mental health problems (e.g. depression) as a consequence of terminal illness (e.g., cancer or chronic organ failure).<br><br>Evidence relating to end of life care for people with mental and behavioural disorders due to psychoactive substance use, except where these coexist with disorders included in the column to the left.<br><br>Evidence relating to end of life care for people with dementia or other neurodegenerative diseases except where these coexist with disorders included in the column to the left.<br><br>Evidence from animal studies.

Comprehensive searches will be conducted across multiple databases from their inception, including: Ovid Medline; Ovid Allied and Complementary Medicine Database (AMED); Proquest Applied Social Sciences Index and Abstracts (ASSIA); Ebsco Cumulative Index of Nursing and Allied Health Literature (CINAHL); Wiley Cochrane Central Register of Controlled Trials; Database of Abstracts of Reviews of Effectiveness; Ovid Embase; Ovid Health Management Information Centre (HMIC); Ovid MEDLINE Epub Ahead of Print and In-Process and Other Non-Indexed Citations; Ovid PsycInfo; Web of Knowledge Science Citation Indexes. A preliminary search will be developed in Ovid Medline using MeSH and text words as piloted in our initial scoping (see 'Background and Rationale' above, and example of search strategy below), then translated to other databases.

Supplementary searches will be undertaken to identify additional papers, information on studies in progress, unpublished research or research reported in the grey literature. Relevant websites and trial registers will be searched (e.g., NIH ClinicalTrials.gov (http://www.clinicaltrials.gov), metaRegister of Controlled Trials (http://www.controlled-trials.com), WHO International Clinical Trials Registry Platform (ICTRP) (http://www.who.int/ictrp/en). To identify published resources that have not yet been catalogued in the electronic databases, recent editions of key journals will be hand-searched.. Reference lists of included studies will be scanned, experts contacted, and forward citation tracking performed using ISI Web of Science.

HS&DR Project 17/100/15: End of life care for people with severe mental illness: an evidence synthesis (the MENLOC study)

Reflecting EPPI Centre (Gough et al., 2017) methods, searches will be conducted for non-research material (e.g., social media, personal accounts, policies) using transparent, clearly described, approaches (Mahood et al., 2014). With advice being taken from members of a project stakeholder advisory group, relevant websites will be searched using search terms and strategies carefully tested and refined as necessary to ensure that all items relevant to the care of people with severe mental illness and (a) advanced, incurable, cancer and/or (b) end-stage lung, heart, renal or liver failure and who are likely to die within the next 12 months are equally included. Candidate websites for searching include: the Care Quality Commission (https://www.cqc.org.uk/), Cancer Research UK (http://www.cancerresearchuk.org/), Cancer Research Wales (https://www.cancerresearchwales.co.uk/), Tenovus Cancer Care (http://www.tenovuscancercare.org.uk/), Mental Health Foundation (https://www.mentalhealth.org.uk/), Centre for Mental Health (https://www.centreformentalhealth.org.uk/), St Mungo's (https://www.mungos.org/), National Kidney Foundation (https://www.kidney.org/), British Liver Trust (https://www.britishlivertrust.org.uk/), British Renal Society (http://britishrenal.org/), The Renal Association (https://renal.org/), British Heart Foundation (https://www.bhf.org.uk/), British Lung Foundation (https://www.blf.org.uk/), National Council for Palliative Care (http://www.ncpc.org.uk/), Hospice UK (http://www.hospiceuk.org/), Marie Curie (https://www.mariecurie.org.uk/), Macmillan Cancer Support (http://www.macmillan.org.uk/) and the Scottish Partnership agency for Palliative Care (https://www.palliativecarescotland.org.uk/).

Preliminary database searching using some of the methods described here has already been carried out, as part of an initial scoping undertaken in preparation of this proposal (see below). The initial search strategy once the project commences will build on this, and it is anticipated that the following MeSH terms and keywords will be used, in various combinations, to inform the systematic search process:

- Palliative care
- Hospice care
- Terminal care
- Terminally ill
- End of life care
- Last year of life
- Neoplasms
- Cancer
- Multiple organ failure
- Mental health
- Depression
- Mental disorders
- Depressive disorder
- Personality disorders
- Bipolar disorder
- Schizophrenia
- Mental illness

In July 2018 we took the opportunity to update and extend our scoping exercise (using terms and keywords reproduced above) using four databases: Ovid MEDLINE; Ovid MEDLINE Epub Ahead of Print, In-Process and Other Non-Indexed Citations and Ovid MEDLINE without Revisions; PsycINFO; and EMBASE. To illustrate this process we reproduce below the search run in MEDLINE, which produced 1,387 citations (before the removal of duplicates) for screening:

Database: Ovid MEDLINE(R) ALL <1946 to July 05, 2018>
Search Strategy:
----
1 exp Palliative care/ (48615)
2 exp Hospice care/ (5807)
3 exp Terminal Care/ (47347)
4 exp Terminally ill/ (6150)
5 ("palliative care" or "hospice care" or "end of life care" or end-of-life).tw. (38005)

6 ((hospice or terminal*) adj3 (care or caring or ill*)).tw. (12527)
7 ("last year of life" or LYOL or "end of life" or "end of their lives").tw. (19101)
8 (end-stage disease* or end stage disease* or end-stage ill* or end stage ill*).tw. (1112)
9 or/1-8 (104642)
10 exp Neoplasms/ (3055654)
11 (cancer* or tumo?r* or neoplas* or malignan* or carcinoma* or adenocarcinoma* or adeno?carcinoma* or choriocrcinoma* or leukemia* or leukaemia* or metastat* or sarcoma* or teratoma* or lymphoma).tw. (3168623)
12 exp Multiple Organ Failure/ (10043)
13 Organ failure.tw. (16597)
14 Pulmonary Disease, Chronic Obstructive/ (32992)
15 (Chronic obstructive pulmonary disease or COPD).tw. (53007)
16 Heart Failure/ (105052)
17 Renal Insufficiency/ (14550)
18 Liver Failure/ (6735)
19 ("cardiac failure" or "liver failure" or "kidney failure" or "renal failure").tw. (114515)
20 Chronic Disease/ (248675)
21 Health service utilization.mp. (1502)
22 lifestyle-related factor*.mp. (337)
23 or/10-22 (4430236)
24 exp Bipolar Disorder/ (37229)
25 exp Schizophrenia/ (97818)
26 (bipolar or mania or Schizophrenia).tw. (148564)
27 exp Depression/ (102372)
28 exp Mental Disorders/ (1125241)
29 ("mental illness" or "mental disorder*" or depression).tw. (333320)
30 mental health condition*.tw. (1794)
31 exp Depressive Disorder/ (99383)
32 exp Mental Health/ (31108)
33 exp Personality Disorders/ (39084)
34 (severe and persistent mental illness).mp. (304)
35 severe mental illness.mp. (3558)
36 or/24-35 (1390277)
37 9 and 23 and 36 (2648)
38 (dementia or Alzheimer).tw. (106344)
39 (Algeria$ or Egypt$ or Liby$ or Morocc$ or Tunisia$ or Western Sahara$ or Angola$ or Benin or Botswana$ or Burkina Faso or Burundi or Cameroon or Cape Verde or Central African Republic or Chad or Comoros or Congo or Djibouti or Eritrea or Ethiopia$ or Gabon or Gambia$ or Ghana or Guinea or Keny$ or Lesotho or Liberia or Madagasca$ or Malawi or Mali or Mauritania or Mauritius or Mayotte or Mozambiq$ or Namibia$ or Niger or Nigeria$ or Reunion or Rwand$ or Saint Helena or Senegal or Seychelles or Sierra Leone or Somalia or South Africa$ or Sudan or Swaziland or Tanzania or Togo or Ugand$ or Zambia$ or Zimbabw$ or China or Chinese or Hong Kong or Macao or Mongolia$ or Taiwan$ or Belarus or Moldov$ or Russia$ or Ukraine or Afghanistan or Armenia$ or Azerbaijan or Bahrain or Cyprus or Cypriot or Georgia$ or Iran$ or Iraq$ or Jordan$ or Kazakhstan or Kuwait or Kyrgyzstan or Leban$ or Oman or Pakistan$ or Palestin$ or Qatar or Saudi Arabia or Syria$ or Tajikistan or Turkmenistan or United Arab Emirates or Uzbekistan or Yemen or Bangladesh$ or Bhutan or British Indian Ocean Territory or Brunei Darussalam or Cambodia$ or India$ or Indonesia$ or Lao or People's Democratic Republic or Malaysia$ or Maldives or Myanmar or Nepal or Philippin$ or Singapore or Sri Lanka or Thai$ or Timor Leste or Vietnam or Albania$ or Andorra or Bosnia$ or Herzegovina$ or Bulgaria$ or Croatia$ or Faroe Islands or Greenland or Liechtenstein or Lithuani$ or Macedonia or Malta or maltese or Romania or Serbia$ or Montenegro or Svalbard or Argentina$ or Belize or Bolivia$ or Brazil$ or Chilean or Colombia$ or Costa Rica$ or Cuba or Ecuador or El Salvador or French Guiana or Guatemala$ or Guyana or Haiti or Honduras or

Jamaica$ or Nicaragua$ or Panama or Paraguay or Peru or Puerto Rico or Suriname or Uruguay or Venezuela or developing countr$ or south America$).ti,sh. (1190707)
40 Academic Dissertations/ (0)
41 thesis.tw. (7038)
42 book.pt. (0)
43 Books/ (3122)
44 or/38-43 (1303105)
45 37 not 44 (2373)
46 limit 45 to (English language and humans and "all adult (19 plus years)") (1387)

Combining these 1,387 citations with citations from the remaining three databases produced a total of 4,754 citations, which reduced to 3,033 once duplicates had been removed with the help of the bibliographic software programme EndNote. Screening based on titles alone reduced this total to 55. With titles and abstracts entered into the software screening programme Covidence, these 55 were reduced to 26 citations potentially meeting our inclusion criteria. Items identified clearly confirm the existence of a literature ready to be synthesised, and include (as illustrative examples) a report into health care use by people with schizophrenia at the end of life (Chochinov et al., 2012), a qualitative study of end of life care perspectives amongst people living with the diagnosis of schizophrenia (Sweers et al., 2013), an analysis of routine health records to examine associations between pre-existing mental illness and service use at the end of life (Lavin et al., 2017) and a whole-population study conducted in Taiwan to explore patterns of end of life care use amongst people with, and without, a diagnosis of schizophrenia (Huang et al., 2018).

Relevant items were also found in July 2018 by following Mahood et al.'s (2014) methods for the searching of grey literature by combining "end of life care" AND "severe mental illness" in the Google UK search engine, with examples including presentations from Canada [http://www.shared-care.ca/files/2C_-_Optimizing_end_of_life_care_(Whitehead).pdf] and New Zealand [http://www.nzcmhn.org.nz/files/file/1105/End%20of%20Life%20care%20for%20people%20with%20SPMI.pdf]. Relevant grey literature documents produced by statutory bodies and charities were also identified via targeted searching of organisational websites. Examples are: a recent review into end of life care produced by the Care Quality Commission (2016a), which contains a report in which carers of people also living with mental illness describe their experiences (Care Quality Commission, 2016b); and a joint report produced by St Mungo's and Marie Curie into end of life care for homeless people, which includes a case study of a woman with schizophrenia and cervical cancer (Kennedy et al., 2013).

Screening

As in our scoping, all citations retrieved will be imported into EndNote, where duplicate references will be removed. All remaining items will then be independently assessed for relevance by two members of the review team using the information provided in the title and abstract. Where any doubt exists the full text will be retrieved. In all cases the full text will be retrieved for all citations that, at this stage, appear to meet the review's inclusion criteria. To achieve a high level of consistency reviewers will screen each retrieved citation for inclusion using a purposely designed form. Disagreements will be resolved through discussion with a third reviewer. Authors of research studies will be contacted by the project team if further information is required. All English language items relating to the provision and receipt of end of life care for people with severe mental illness and an additional diagnosis of advanced, incurable, cancer and/or end-stage lung, heart, renal or liver failure will be included at this stage.

Quality appraisal

Following searching and screening, information from research publications will be independently assessed for methodological quality by two reviewers using design-specific tools developed by the Critical Appraisal Skills Programme (CASP) (2016). Alternative tools, reflecting the specific design and methods used in individual research outputs, will be used as necessary if suitable CASP tools are not available. Any disagreement on quality will be resolved through discussion with a third reviewer.

At this stage all research items will be included other than those which are fatally flawed. Non-research evidence (e.g. policies, reports, expert opinion pieces, case studies etc.) will not be subjected to quality appraisal.

Data extraction

All data will be extracted directly into tables and will follow the format recommended by the CRD (2009). One reviewer will extract the data and a second reviewer will independently check the data extraction forms for accuracy and completeness. Any disagreements will be noted and resolved by consensus within the review team.

Data analysis and synthesis

Researchers informed by the EPPI-Centre approach recognise that different strategies exist for the analysis and synthesis of data (Gough et al., 2017). The synthesis in this review will have both configurative (involving the exploration of potentially heterogeneous materials) and aggregative (involving the pooling of data, where possible) elements (Gough et al., 2012).

For intervention studies meta-analyses of data will be performed where possible. Tests for heterogeneity will be applied. Where statistical pooling is not possible the findings, along with data from non-intervention quantitative studies, will be thematically presented (Thomas and Harden, 2008). The software programme NVivo will be used to help manage this process. Qualitative data, and data from non-research items, will presented in configurative fashion using a thematic approach again assisted by NVivo. Themes will be developed inductively based on close reading of the content of all items included.

Assessing confidence

The strength of findings from the meta-analysis of intervention studies will be assessed using the Grading of Recommendations, Assessment, Development and Evaluation (GRADE) approach (Guyatt et al., 2008). The strength of synthesised qualitative and non-intervention findings will be assessed using the Confidence in the Evidence from Reviews of Qualitative Research (CerQual) approach (Lewin et al., 2015). The original CerQual approach was designed for qualitative findings, but we will use a process previously used by members of this research team in HS&DR 11/1024/08 (Hannigan et al., 2015) and in HS&DR 08/1704/211 (Edwards et al., 2014) in additionally adopting CerQual for the assessment of the confidence of synthesised findings from surveys and other non-intervention quantitative studies.

Overall summary

An overarching summary will bring all elements together. It will present key themes arising from this project as a whole, and do so in accessible manner to ensure reach. Help with this will be sought from the stakeholder advisory group and from the NIHR Dissemination Centre. Factors helping and hindering end of life care for people with severe mental illness will be identified, along with evidence relating to services, processes, interventions, views and experiences. Implications will be stated, and recommendations made for future research.

DISSEMINATION AND PROJECTED OUTPUTS

The main output from this project will be a comprehensive, rigorously conducted, synthesis of research and other evidence relating to services, interventions, processes, views and experiences in the context of end of life care for people with severe mental illness who have an additional diagnosis of advanced, incurable, cancer and/or end-stage lung, heart, renal or liver failure and who are likely to die within the next 12 months.

We will work with the NIHR Dissemination Centre to share findings from our study and to make sure they have maximum benefit, and will follow NIHR guidance by paying close attention to stakeholder

engagement, format, opportunities, context and timing (National Institute for Health Research, undated). Our stakeholders are policymakers, commissioners, managers and practitioners at the interface of the end of life and mental health care fields, along with people using services and carers. We plan the setting up of a stakeholder advisory group (numbering an anticipated 8-12 individuals), building on preliminary discussions held with clinical and managerial leads at relevant, South Wales-based, NHS and charity mental health services, cancer centres and palliative care organisations. Our list of candidate bodies from which stakeholder representation has been secured (or with which we will otherwise engage in order to keep members informed of our project's progress) currently includes: the National End of Life Care Programme Board in England; the End of Life Care Implementation Board in Wales; the National Mental Health Partnership Board in Wales; and the Mental Health and Dementia Programme Board in England. Professional, academic and management stakeholder organisations include: Mental Health Nurse Academics UK (for which chief applicant Ben Hannigan is elected Vice Chair and Chair-elect, and for which co-applicant Michael Coffey is a past Chair); the European Oncology Nursing Society (the immediate past President of which is a colleague of the applicants at Cardiff University); the Palliative Care Research Society UK, and the National Council for Palliative Care. Roger Pratt and Alan Meudell, as service user and carer co-applicants, will be invited to help identify patient and public representatives to also join this stakeholder group. We will work with our advisory group to inform the decisions we take throughout the life of the study, and will particularly work with members to develop a publicity and dissemination strategy.

In our main output (the full and final report for the NIHR 'Health Services and Delivery Research' journal) we will provide a clear statement of the implications of what we have found for services and practice, and offer explicit recommendations for future research where knowledge gaps are uncovered. We also anticipate working with the NIHR Dissemination Centre to promote our findings through NIHR Signals, Highlights and Themed Reviews where opportunities allow. As we have done in other NIHR studies on which members of this team have worked (e.g., HS&DR 11/1024/08) we will produce a high-quality accessible summary for publicising online, via social media and in paper form. Our intended audience will include end of life and mental health care managers, practitioners and educators along with members of the public and patients. We will work with our stakeholder advisory group to make sure this document is understandable. Papers reporting main findings will be published in gold open access form (to maximise reach) in relevant world-leading journals, tailored to audiences; candidate titles include those in the BioMed Central series, including BMC Psychiatry, BMC Health Services Research and BMC Palliative Care. This will allow all parties free access to information to facilitate decisions on service organisation, and to support meaningful decisions on future care commissioning and provision. We will also present findings at key stakeholder conferences in the mental health and end of life fields and take opportunities to provide briefings for key stakeholder organisations such as those identified above. We will create an opportunity to directly engage with stakeholders via an end of project dissemination event, which will be modelled on single-day impact events in which members of the team have previously participated (e.g., in HS&DR 11/2004/12). Throughout the study we will use social media (e.g., using a dedicated Twitter account) and a project website to promote wider interest in our work, and make opportunities to engage with the public via regular fora such as Cardiff PublicUni (https://en-gb.facebook.com/PUBlicengagementcardiff/). As part of our final dissemination strategy we will request a blogpost on the Mental Elf Blog (https://www.nationalelfservice.net/mental-health/), and will continue using our networks to share what we find as widely as possible. We will keep these approaches to dissemination under active review, and will continue to be advised by NIHR Dissemination Centre colleagues and our stakeholder advisory group as appropriate.

In all our outputs, where our findings support this we will say how high-quality end of life care for people with severe mental illness should be organised, providing guidance to commissioners, managers and practitioners concerned with improving services and the user and carer experience. We anticipate that the new knowledge we create will have a significant impact on health services organisation and delivery, informing action (e.g., via future NICE guidance and other initiatives) to tackle the problem of disadvantaged dying and promote parity of esteem. We also intend this project to be the starting point for a larger programme of related research in its field, designed over time to produce an evidence base supporting transformations in how care is organised and provided. Building on this project we therefore anticipate future data-generating studies investigating current services (including the size and spread of provision) and testing innovations and interventions.

HS&DR Project 17/100/15: End of life care for people with severe mental illness: an evidence synthesis (the MENLOC study)

PLAN OF INVESTIGATION AND TIMETABLE

We are able to directly begin work on this project on November 1st 2018 as all members of our team are in place, and as we are not required to secure independent NHS research ethics committee approval, due to the nature of the study.

Our projected timetable is as follows:

Coffey M, *et al. BMJ Open* 2022; 12:e053223. doi: 10.1136/bmjopen-2021-053223

HS&DR Project 17/100/15: End of life care for people with severe mental illness: an evidence synthesis (the MENLOC study)

	-3	-2	-1	1	2	3	4	5	6	7	8	9	10	11	12	13	14	15	+1
	Aug 18	Sep 18	Oct-18	Nov 18	Dec 18	Jan-19	Feb 19	Mar 19	Apr-19	May 19	Jun-19	Jul-19	Aug 19	Sep 19	Oct-19	Nov 19	Dec 19	Jan-20	Feb 20
Notification of award																			
Project set-up (refine protocol, agree subcontracts, comms)																			
Populate stakeholder advisory group (SAG)																			
Project begins																			
SAG meeting																			
Searching and screening for evidence																			
Quality appraisal and data extraction																			
Data analysis, data synthesis and assessing confidence																			
Summary and writing up																			
End of project event																			
Project ends																			
Report to funder																			

PROJECT MANAGEMENT

We are a strong and balanced research team well-placed to complete this project on time and to a high standard. We have expertise in: leading and managing NIHR-funded evidence syntheses to completion (Ben Hannigan and Deborah Edwards); mental health services and research (Ben Hannigan and Michael Coffey); cancer, palliative and end of life services and research (Sally Anstey); organ failure services and research (Paul Gill); systematic reviewing (Mala Mann, Deborah Edwards, Ben Hannigan); and lived experience (Alan Meudell, Roger Pratt). The project manager for this study, Deborah Edwards, is a health services researcher and systematic reviewer with experience of managing complex projects across institutional boundaries. As the researcher with the greatest time attachment to the project her responsibilities will include the day-to-day management of the study. Oversight and overall responsibility for the project will fall to the chief investigator, Ben Hannigan, who is an experienced mental health services researcher and who (with Deborah Edwards and others) has successfully completed two competitively funded systematic reviews in the mental health field in the past (including HS&DR 11/1024/08).

An independently chaired stakeholder advisory group will be populated by representatives drawn from the mental health and end of life fields, and will meet with the project team, in Cardiff, at three strategic time points in the life of the study (see plan of investigation above). As an important part of the work of this group will advising on dissemination, impact and engagement details about membership have been given above (see ‘Dissemination and projected outputs’). A first meeting will be scheduled at the commencement of the project, to refine search terms and strategies for the evidence review. A second meeting will take place at the completion of evidence searching and screening. A final meeting will take place at the commencement of the whole-project synthesis and report writing phase, where progress and plans for dissemination and maximising impact will be discussed. The costs attached to this project include those associated with the convening and running of the advisory group.

Using a model successfully used in HS&DR 11/1024/08 members of the project team will initially maintain weekly contact via email, telephone and/or videoconference to ensure that packages of work are distributed according to team members’ identified responsibilities, and to ensure that work plans proceed according to agreed schedules. Close monitoring of overall progress against milestones will ensure project completion on time and within budget.

APPROVAL BY ETHICS COMMITTEES

No ethics approval is needed, as this is an evidence synthesis.

PATIENT AND PUBLIC INVOLVEMENT

This proposal has been shaped by people with experience of mental health problems, cancer and other long-term conditions, and by people with experience of caring. The original idea for the study arose following sustained and critical discussions between two co-applicants, Roger Pratt and Sally Anstey, who together identified that individuals with severe mental illness are disadvantaged when diagnoses of advanced cancer or end-stage organ failure are made. Roger is a retired mental health social worker, who lives with lymphoma (in long term remission) and heart failure. He cared for his wife who died from advanced peritoneal cancer; she received specialist palliative care in the last year of her life and died in a hospice. This discussion observed that professional perceptions and misperceptions such as stigma and fear impact on management (e.g., pain control, supporting choice and place of care/death) in the case of people with severe mental illness receiving end of life care. We have since involved Alan Meudell in the project team. Alan is a mental health service user consultant and researcher. He has worked on two NIHR studies and one Health and Care Research Wales (HCRW) study, and leads training for HCRW on involving service users in research. He is also interested in the physical health care of people living with mental health difficulties, and on the provision of equitable services.

The roles played by Roger and Alan will be as equal partners in all stages of the research process except the literature search (which will be led, uniquely, by Mala Mann as the team’s information services specialist). Both will participate in initial training which all applicants will join, and will work as part of the team in the critical analysis of the selected articles using CASP (or other appropriate) tools. To support active roles in this activity they will be mentored by other team members as necessary and

will have access to training facilitated by Health and Care Research Wales. They will also contribute to the task of identifying carers and service users to join our stakeholder group. Critically, both will also be invited to advise on the ongoing focus and direction of the study, and to contribute to the accessible writing up of the final report and other outputs. They will be invited to act as ambassadors in the dissemination of the results and to link with existing patient/service user, support and self-help groups to raise awareness and maximise impact.

In preparing this proposal for submission to the HS&DR Programme we also presented our plans to the Patient Experience and Evaluation in Research (PEER) group at Swansea University (http://www.swansea.ac.uk/humanandhealthsciences/research/patientexperienceandevaluationinresearchpeergroup/). The PEER group comprises people with experience of using health care services and of caring, and exists to provide a public and patient view of research proposals before they are submitted for funding. When our proposal was considered by the PEER group members gave it a very positive response, stating that this was a much-needed project in an area that is largely ignored. People were particularly interested in our plans for choosing members of our stakeholder advisory group, and advised us to engage with charities such as Macmillan, with hospices, and with palliative care staff such as nurses. They also recognised the difficulty of actually recruiting stakeholder advisory group members from the target population, but believed we should at least attempt to engage by offering opportunities for people with severe mental illness and end of life diagnoses to participate in any way feasible for them. We have noted this advice, and have approached services in South Wales to seek their agreement in principle to help put us in touch with service users and carers (as well as managers and practitioners) able to advise our project once funded. We will pursue these connections further once confirmation of support has been received from the HS&DR Board.

Our engagement with patients and the public reflects commitments and experiences demonstrated in other studies on which members of this project team have worked. Examples include: HS&DR 11/1024/08 (the RiSC study, an evidence synthesis into ‘risk’ for young people in mental health hospital which actively involved young people as stakeholders in shaping the study’s progress); HS&DR 11/2004/12 and HS&DR 13/10/75 (COCAPP and COCAPP-A which investigated care planning and care coordination in mental health services, in which service users and carers collaborated as members of lived experiences advisory groups and in which people with experience of mental health difficulties worked as researchers conducting qualitative interviews with service user participants); and Health and Care Research Wales SC-12-03 (Plan4Recovery, which involved people with experience of using mental health services as members of a lived experiences advisory group and as qualitative interviewers).

EXPERTISE AND JUSTIFICATION OF SUPPORT REQUIRED

We are a strong and balanced team. Ben Hannigan (BH) is a mental health services researcher who has led a previous NIHR evidence synthesis (HS&DR 11/1024/08) and who has worked on other NIHR (HS&DR 11/2004/12, HS&DR 13/10/75) and Health and Care Research Wales (SC-12-03) mental health studies. He will be chief investigator, contributing across the evidence synthesis, writing up and dissemination. Michael Coffey (MC) is a mental health services researcher and chief investigator on Health and Care Research Wales social care research grant (SC-12-03), and co-investigator on NIHR HS&DR 11/2004/12 and HS&DR 13/10/75. He will contribute to the evidence synthesis, writing up and dissemination. Paul Gill (PG) is an experienced researcher in the field of chronic conditions, and end-stage organ failure particularly. He will contribute to the evidence synthesis, writing up and dissemination. Mala Mann (MM) is an information specialist with expertise in advanced literature searching and the development of systematic review methodologies. She will lead the database searching. Sally Anstey (SA) is an experienced clinician, educationalist and researcher in the end of life and cancer care fields. She will contribute to the to the evidence synthesis, writing up and dissemination. Deborah Edwards (DE) is an experienced health services researcher who has successfully completed multiple systematic reviews (including for the NIHR) using a variety of approaches. She will project manage this study, and contribute to the evidence synthesis, writing up and dissemination. Roger Pratt (RP) is a retired mental health social worker, and a past member of the Velindre Cancer Centre Patient Liaison Group. He will contribute to the to the evidence synthesis, writing up and dissemination. Alan Meudell (AM) is a mental health service user consultant, researcher and trainer, and past trustee of the charity Mind. He has experience in NIHR projects and in training. He will contribute to the to the evidence synthesis, writing up and dissemination.

This division of labour and allocation of time is appropriate for a study of this nature. Additional, essential, non-staff costs included are: travel, including to three project/steering group meetings plus refreshments for the same; a sum to cover the costs of interlibrary loans; attendance for Cardiff staff at two conferences, one of which is anticipated to be the International Mental Health Nursing Research Conference; attendance for Swansea staff at one conference; a sum for the planned end of project impact event, to which key stakeholders from the mental health and end of life fields will be invited; a sum for the production and distribution of our accessible summary; and a sum to cover author processing charges for two gold open access publications. Support for these latter costs is requested to maximise dissemination, reach and impact.

REFERENCES

ADDINGTON-HALL, J. 2000. *Positive partnerships: palliative care for adults with severe mental health problems,* London, National Council for Hospice and Specialist Palliative Care Services.
ALL WALES PALLIATIVE CARE PLANNING GROUP 2008. *Report to Minister for Health and Social Services on palliative care services (the Sugar Report),* Cardiff, Welsh Assembly Government.
ALL WALES PALLIATIVE CARE PLANNING GROUP 2009. *Implementation of palliative care report: palliative care services funding 2008 to 2009 (the Finlay Report),* Cardiff, Welsh Assembly Government.
AMERICAN PSYCHIATRIC ASSOCIATION 2014. *Diagnostic and statistical manual of mental disorders (DSM-V),* Arlington, American Psychiatric Publishing.
BATTY, G. D., WHITLEY, E., GALE, C. R., OSBORN, D., TYNELIUS, P. & RASMUSSEN, F. 2012. Impact of mental health problems on case fatality in male cancer patients. *British Journal of Cancer,* 106**,** 1842-1845.
CARE QUALITY COMMISSION 2016a. *A different ending: end of life care review,* Newcastle upon Tyne, Care Quality Commission.
CARE QUALITY COMMISSION 2016b. *A different ending: end of life care review. People with a mental health condition,* Newcastle upon Tyne, Care Quality Commission.
CARNEY, C. P., JONES, L. & WOOLSON, R. F. 2006. Medical comorbidity in women and men with schizophrenia. *Journal of General Internal Medicine,* 21**,** 1133-1137.
CENTRE FOR MENTAL HEALTH 2010. *The economic and social costs of mental health problems in 2009/10,* London, Centre for Mental Health.
CENTRE FOR REVIEWS AND DISSEMINATION 2009. *Systematic reviews: CRD's guidance for undertaking reviews in health care,* York, University of York.
CHESNEY, E., GOODWIN, G. M. & FAZEL, S. 2014. Risks of all-cause and suicide mortality in mental disorders: a meta-review. *World Psychiatry,* 13**,** 153-160.
CHOCHINOV, H. M., MARTENS, P. J., PRIOR, H. J. & KREDENTSER, M. S. 2012. Comparative health care use patterns of people with schizophrenia near the end of life: A population-based study in Manitoba, Canada. *Schizophrenia Research,* 141**,** 241-246.
CRITICAL APPRAISAL SKILLS PROGRAMME (CASP). 2016. *CASP checklists* [Online]. http://www.casp-uk.net/#!checklists/cb36. [Accessed].
DEPARTMENT OF HEALTH 1995. *Building bridges: a guide to arrangements for interagency working for the care and protection of severely mentally ill people,* London, Department of Health.
DEPARTMENT OF HEALTH 2010. *National cancer patient experience survey programme: 2010 national survey report,* London, Department of Health.
DEPARTMENT OF HEALTH 2016. *Our commitment to you for end of life care,* London, Department of Health.
DEPARTMENT OF HEALTH 2017. *A framework for mental health research,* London, Department of Health.
DESAI, M. M., BRUCE, M. L. & KASL, S. V. 1999. The effects of major depression and phobia on stage at diagnosis of breast cancer. *International Journal of Psychiatry in Medicine,* 29**,** 29-45.
DISABILITY RIGHTS COMMISSION 2006. *Equal treatment: closing the gap. A formal investigation into physical health inequalities experienced by people with learning disabilities and/or mental health problems,* London, Disability Rights Commission.
DIXON, J., KING, D., MATOSEVIC, T., CLARK, M. & KNAPP, M. 2015. *Equity in provision of palliative care in the UK,* London, LSE and Marie Curie.
DRAKEFORD, M. 2014. *Palliative care services in Wales are making an enormous difference to people's quality of life,* Speech given in Ty Gobaith hospice, Conwy, http://www.wired-

gov.net/wg/news.nsf/articles/Palliative+care+services+in+Wales+are+making+an+enormous+difference+to+peoples+quality+of+life+Mark+Drakeford+28102014162040?open.
EDWARDS, D., NOYES, J., HAF SPENCER, L. & GREGORY, J. W. 2014. An ongoing struggle: a mixed-method systematic review of interventions, barriers and facilitators to achieving optimal self-care by children and young people with type 1 diabetes in educational settings. *BMC Paediatrics,* 14.
ELLISON, N. 2008. *Mental health and palliative care. Literature review,* London, Mental Health Foundation.
EXLEY, C. 2004. Review article: the sociology of dying, death and bereavement. *Sociology of Health and Illness,* 26**,** 110-122.
FOTI, M. E. 2012. Palliative care for patients with serious mental Illness. *In:* CHOCHINOV, H. M. & BREITBART, W. (eds.) *Handbook of psychiatry in palliative medicine.* 2nd ed. Oxford: Oxford University Press.
FRIEDLI, L. & PARSONAGE, M. 2009. *Promoting mental health and preventing mental illness: the economic case for investment in Wales,* Cardiff, All Wales Mental Health Promotion Network.
GLOBAL BURDEN OF DISEASE STUDY 2013 COLLABORATORS 2015. Global, regional, and national incidence, prevalence, and years lived with disability for 301 acute and chronic diseases and injuries in 188 countries, 1990-2013: a systematic analysis for the Global Burden of Disease Study 2013. *The Lancet,* 386**,** 743-800.
GOUGH, D., OLIVER, S. & THOMAS, J. (eds.) 2017. *An introduction to systematic reviews,* London: Sage.
GOUGH, D., THOMAS, J. & OLIVER, S. 2012. Clarifying differences between review designs and methods. *Systematic Reviews,* 1.
GUYATT, G. H., OXMAN, A. D., VIST, G. E., KUNZ, R., FALCK-YTTER, Y., ALONSO-COELLO, P. & SCHÜNEMANN, H. J. 2008. GRADE: an emerging consensus on rating quality of evidence and strength of recommendations. *BMJ,* 336**,** 924-926.
HACKETT, A. & GAITAN, A. 2007. A qualitative study assessing mental health issues in two hospices in the UK. *International Journal of Palliative Care Nursing,* 13**,** 273-281.
HANNIGAN, B., EDWARDS, D., EVANS, N., GILLEN, E., LONGO, M., PRYJMACHUK, S. & TRAINOR, G. 2015. An evidence synthesis of risk identification, assessment and management for young people using tier 4 inpatient child and adolescent mental health services. *Health Services and Delivery Research,* 3.
HOWARD, L. M., BARLEY, E. A., DAVIES, E., RIGG, A., LEMPP, H., ROSE, D. & THORNICROFT, G. 2010. Cancer diagnosis in people with severe mental illness: practical and ethical issues. *The Lancet Oncology,* 11**,** 797-804.
HUANG, H. K., WANG, Y.W., HSIEH, J. G. & HSIEH, C. J. 2018. Disparity of end-of-life care in cancer patients with and without schizophrenia: A nationwide population-based cohort study. *Schizophrenia Research,* 195**,** 434-440.
HUGHES-HALLET, T., CRAFT, A. & DAVIES, C. 2011. *Palliative care funding review: funding the right care and support for everyone,* London, Department of Health.
INDEPENDENT CANCER TASKFORCE. 2015. *Achieving world-class cancer outcomes: a strategy for England 2015-2020* [Online]. http://www.cancerresearchuk.org/sites/default/files/achieving_world-class_cancer_outcomes_-_a_strategy_for_england_2015-2020.pdf. [Accessed].
IRWIN, K. E., HENDERSON, D. C., KNIGHT, H. P. & PIRL, W. F. 2014. Cancer care for individuals with schizophrenia. *Cancer,* 120**,** 323-334
ISHIKAWA, H., YASUNAGA, H., MATSUI, H., FUSHIMI, K. & KAWAKAMI, N. 2016. Differences in cancer stage, treatment and in-hospital mortality between patients with and without schizophrenia: retrospective matched-pair cohort study. *British Journal of Psychiatry,* 208**,** 239-244.
KENNEDY, P., SARAFI, C. & GREENISH, W. 2013. *Homelessness and end of life care*, Marie Curie Cancer Care and St Mungo's.
KNAPIK, G. P. & GRAOR, C. H. 2013. Engaging persons with severe persistent mental illness into primary care. *Journal for Nurse Practitioners,* 9**,** 283-287.
KRONENBERG, C., DORAN, T., GODDARD, M., KENDRICK, T., GILBODY, S., DARE, C. R., AYLOTT, L. & JACOBS, R. 2017. Identifying primary care quality indicators for people with serious mental illness: a systematic review. *British Journal of General Practice,* 67**,** e519-e530.
LAVIN, K., DAVYDOW, D. S., DOWNEY, L., ENGELBERG, R. A., DUNLAP, B., SIBLEY, J., LOBER, W. B., OKIMOTO, K., KHANDELWAL, N., LOGGERS, E. T., TENO, J. M. & CURTIS, J. R. 2017. Effect of psychiatric illness on acute care utilization at end of life from serious medical illness. *Journal of Pain and Symptom Management,* 54**,** 176-185.

LEWIN, S., GLENTON, C., MUNTHE-KAAS, H., CARLSEN, B., COLVIN, C. J., GÜLMEZOGLU, M., NOYES, J., BOOTH, A., GARSIDE, R. & RASHIDIAN, A. 2015. Using qualitative evidence in decision making for health and social interventions: an approach to assess confidence in findings from qualitative evidence syntheses (GRADE-CERQual). *PLOS Medicine,* 12**,** e1001895.
MAHOOD, Q., VAN EERD, D. & IRVIN, E. 2014. Searching for grey literature for systematic reviews: challenges and benefits. *Research Synthesis Methods,* 5**,** 221-234.
MARIE CURIE 2015. *Triggers for palliative care. Improving access to care for people with diseases other than cancer: implications for Wales,* London, Marie Curie.
MARIE CURIE AND THE BEVAN FOUNDATION 2014. *Death and dying in Wales,* London and Cardiff, Marie Curie and the Bevan Foundation.
MCCORMACK, P. & SHARP, D. M. M. 2006. Palliative care for people with mental health problems. *European Journal of Palliative Care,,* 13**,** 198-201.
MENTAL HEALTH TASKFORCE 2016. *The five year forward view for mental health*, Mental Health Taskforce.
MILLMAN, J., GALWAY, K., SANTIN, O. & REID, J. 2016. Cancer and serious mental illness – patient, caregiver and professional perspectives: study protocol. *Journal of Advanced Nursing,* 72**,** 217-226.
MOHER, D., LIBERATI, A., TETZLAFF, J., ALTMAN, D. G. & GROUP, T. P. 2009. Preferred reporting items for systematic reviews and meta-analyses: the PRISMA statement. *PLoS Medicine,* 6**,** e1000097.
MORGAN, B. D. 2016. 'No right place to die'. Nursing attitudes and needs in caring for people with serious mental illness at end-of-life. *Journal of the American Psychiatric Nurses Association,* 22**,** 31-42.
NATIONAL INSTITUTE FOR CLINICAL EXCELLENCE 2004. *Improving supportive and palliative care for adults with cancer,* London, National Institute for Clinical Excellence.
NATIONAL INSTITUTE FOR HEALTH AND CARE EXCELLENCE 2009. *Borderline personality disorder: recognition and management,* London, National Institute for Health and Care Excellence.
NATIONAL INSTITUTE FOR HEALTH AND CARE EXCELLENCE 2011. *Service user experience in adult mental health services,* London, National Institute for Health and Care Excellence.
NATIONAL INSTITUTE FOR HEALTH AND CARE EXCELLENCE 2014a. *Bipolar disorder: assessment and management,* London, National Institute for Health and Care Excellence.
NATIONAL INSTITUTE FOR HEALTH AND CARE EXCELLENCE 2014b. *Psychosis and schizophrenia in adults: prevention and management,* London, National Institute for Health and Care Excellence.
NATIONAL INSTITUTE FOR HEALTH AND CARE EXCELLENCE 2018 [anticipated]. *End of life care for adults in the last year of life: service delivery,* London, National Institute for Health and Care Excellence.
NATIONAL INSTITUTE FOR HEALTH RESEARCH 2017. *The Dissemination Centre's upcoming work schedule,* http://www.dc.nihr.ac.uk/about/upcoming-work.htm.
NATIONAL INSTITUTE FOR HEALTH RESEARCH undated. *How to disseminate your research: getting your message heard - and used,* https://www.nihr.ac.uk/funding-and-support/documents/funding-for-research-studies/manage-my-study/How-to-disseminate-your-research/dissemination-guidance.pdf.
NEWTON, J. N., BRIGGS, A. D. M., MURRAY, C. J. L., DICKER, D., FOREMAN, K. J., WANG, H., NAGHAVI, M., FOROUZANFAR, M., OHNO, S. L., BARBER, R. M., VOS, T., STANAWAY, J. D., SCHMIDT, J. C., HUGHES, A. J., FAY, D. F. J., ECOB, R., GRESSER, C., MCKEE, M., RUTTER, H., ABUBAKAR, I., ALI, R., ANDERSON, H. R., BANERJEE, A., BENNETT, D., BERNABÉ, E., BHUI, K. S., BIRYUKOV, S. M., BOURNE, R. R., BRAYNE, C. E. G., BRUCE, N. G., BRUGHA, T. S., BURCH, M., CAPEWELL, S., CASEY, D., CHOWDHURY, R., COATES, M. M., COOPER, C., CRITCHLEY, J. A., DARGAN, P. I., DHERANI, M. K., ELLIOTT, P., EZZATI, M., FENTON, K. A., FRASER, M. S., FÜRST, T., GREAVES, F., GREEN, M. A., GUNNELL, D. J., HANNIGAN, B. M., HAY, R. J., HAY, S. I., HEMINGWAY, H., LARSON, H. J., LOOKER, K. J., LUNEVICIUS, R., LYONS, R. A., MARCENES, W., MASON-JONES, A. J., MATTHEWS, F. E., MOLLER, H., MURDOCH, M. E., NEWTON, C. R., PEARCE, N., PIEL, F. B., POPE, D., RAHIMI, K., RODRIGUEZ, A., SCARBOROUGH, P., SCHUMACHER, A. E., SHIUE, I., SMEETH, L., TEDSTONE, A., VALABHJI, J., WILLIAMS, H. C., WOLFE, C. D. A., WOOLF, A. D. & DAVIS, A. C. J. 2015. Changes in health in England, with analysis by English regions and areas of deprivation, 1990-2013: a systematic analysis for the Global Burden of Disease Study 2013. *The Lancet,* 386**,** 2257-2274.

NORTHERN IRELAND EXECUTIVE 2010. *Living matters, dying matters. A palliative and end of life care strategy for adults in Northern Ireland,* Belfast, Northern Ireland Executive.
PAYNE, S. 2011. From here to there: reaching out for palliative care. *European Journal of Palliative Care,* 18**,** 264-265.
PICKER INSTITUTE EUROPE 2017. *Wales Cancer Patient Experience Survey 2016: National Report,* Oxford, Picker Institute Europe.
PICOT, S. A., GLAETZER, K. M. & MYHILL, K. J. 2015. Coordinating end of life care for individuals with a mental illness: a nurse practitioner collaboration. *Collegian,* 22**,** 143-149.
PRICE, A., HOTOPF, M., HIGGINSON, I., MONROE, B. & HENDERSON, M. 2006. Psychological services in hospices in the UK and Republic of Ireland. *Journal of the Royal Society of Medicine,* 99**,** 637-639.
PROSPERO: INTERNATIONAL PROSPECTIVE REGISTER OF SYSTEMATIC REVIEWS undated. https://www.crd.york.ac.uk/prospero/.
SCOTTISH GOVERNMENT 2008a. *Better cancer care: an action plan,* Edinburgh, Scottish Government.
SCOTTISH GOVERNMENT 2008b. *Living and dying well: a national action plan for palliative and end of life care in Scotland,* Edinburgh, Scottish Government.
SCOTTISH GOVERNMENT 2009. *Improving the health and wellbeing of people with long term conditions in Scotland: a national action plan,* Edinburgh, Scottish Government.
SHALEV, D., BREWSTER, K., ARBUCKLE, M. R. & LEVENSON, J. A. 2017. A staggered edge: end-of-life care in patients with severe mental illness. *General Hospital Psychiatry,* 44**,** 1-3.
SWEERS, K., DIERCKX DE CASTERLÉ, B., DETRAUX, J. & DE HERT, M. 2013. End-of-life (care) perspectives and expectations of patients with schizophrenia. *Archives of Psychiatric Nursing,* 27**,** 246-252.
TAYLOR, J., SWETENHAM, K., MYHILL, K., PICOT, S., GLAETZER, K. & VAN LOON, A. 2012. IMhPaCT: an education strategy for cross-training palliative care and mental health clinicians. *International Journal of Palliative Nursing,* 18**,** 290-294.
THOMAS, J. & HARDEN, A. 2008. Methods for the thematic synthesis of qualitative research in systematic reviews. *BMC Medical Research Methodology,* 8.
THORNICROFT, G. 2013. Premature death among people with mental illness. *BMJ,* 346.
WARREN, M. B., LAPID, M. I., MCKEAN, A. J., CHA, S. S., STEVENS, M. A., BREKKE, F. M. & BURTON, M. C. 2014. Code status discussions in psychiatric and medical inpatients. *Journal of Clinical Psychiatry,* 76**,** 49-53.
WELSH ASSEMBLY GOVERNMENT 2005. *Designed for life: creating world class health and social care for Wales in the 21st Century,* Cardiff, Welsh Assembly Government.
WELSH GOVERNMENT 2013. *Together for health: delivering end of life care. A delivery plan up to 2016 for NHS Wales and its partners,* Cardiff, Welsh Government.
WOODS, A., WILLISON, K., KINGTON, C. & GAVIN, A. 2008. Palliative care for people with severe persistent mental illness: A review of the literature. *The Canadian Journal of Psychiatry,* 53**,** 725-736.
WORLD HEALTH ORGANIZATION 1992. *The 10th revision of the international classification of diseases and related health problems (ICD-10),* Geneva, World Health Organization.
ZABORA, J. R., LOSCALZO, M. & SMITH, E. 2000. Psychosocial rehabilitation. *In:* ABELOFF, M. D., ARMITAGE, J. O., LICHTER, A. S. & NIEDERHUBER, J. E. (eds.) *Clinical oncology.* New York: Churchill Livingstone.

Coffey M, *et al. BMJ Open* 2022; 12:e053223. doi: 10.1136/bmjopen-2021-053223
