# Supplementary material for: End-of-life care for people with severe mental illness: mixed methods systematic review and thematic synthesis of published case studies (the MENLOC study)
Source: BMJ Open. 2022 Feb 22;12(2):e053223. doi: 10.1136/bmjopen-2021-053223 (PMC8867317; doi:10.1136/bmjopen-2021-053223)
Supplement: Supplementary data [file bmjopen-2021-053223supp003.pdf]

Database search strategies

Database: AMED (Allied and Complementary Medicine)

Search Strategy:

----

1 exp Palliative care/ (6092)

2 exp Hospice care/ (2100)

3 exp Terminal Care/ (5265)

4 ("palliative care" or hospice or "end of life care" or end-of-life).tw. (13126)

5 ((hospice or terminal*) adj3 (care or caring or ill*)).tw. (7843)

6 ("Irreversible condition" or "terminal condition" or fatal illness).tw. (27)

7 ("last year of life" or LYOL or "end of life" or "end of their lives" or "last six months of life" or "last 6 months of life").tw. (3749)

8 (end-stage disease* or end stage disease* or end-stage ill* or end stage ill* or end-stage or end stage).tw. (539)

9 (expected adj3 die).tw. (15)

10 (imminent adj3 death).tw. (44)

11 ("Dying soon" or "expected death" or "imminently dying" or Moribund).tw. (40)

12 (conservative adj2 (treatment or management)).tw. (1010)

13 Withholding Treatment/ (66)

14 Treatment Refusal/ (134)

15 (Refus* adj3 (treat* or care or intervention or dialysis)).tw. (272)

16 ((withdrew or withdraw* or withhold*) adj3 (treat* or car* or intervene* or therap* or dialysis or transplant*)).tw. (363)

17 or/1-16 (16993)

18 exp Neoplasms/ (14717)

19 (cancer* or tumo?r* or neoplas* or malignan* or carcinoma* or adenocarcinoma* or adeno?carcinoma* or choriocrcinoma* or leukemia* or leukaemia* or metastat* or sarcoma* or teratoma* or lymphoma).tw. (19919)

20 enzyme disorder.tw. (1)

21 Hereditary disease/ (147)

22 ("duchenne muscular dystrophy" or "amyotrophic lateral sclerosis" or ALS).tw. (1007)

23 amyotrophic lateral sclerosis/ (223)

24 Muscular dystrophy, Duchenne/ (70)

25 ("Genetic disease" or "genetic condition").tw. (18)

26 Cystic Fibrosis/ (451)

27 ("cystis fibrosis" or CF).tw. (314)

28 "Multi* Organ Failure".tw. (20)

29 ("Organ failure" or "chronic organ failure").tw. (49)

30 Pulmonary Disease, Chronic Obstructive/ (449)

31 ("renal insufficiency" or "serious physical illness").tw. (27)

32 (Chronic obstructive pulmonary disease or COPD).tw. (1665)

33 (chronic adj3 (illness or condition or disease*)).tw. (9365)

34 "chronic medical condition".tw. (10)

35 Heart Failure/ (394)

36 (chronic adj2 ("cardiac failure" or "liver failure" or "kidney failure" or "end-stage renal disease" or ESRD or "renal failure" or "heart failure")).tw. (439)

37 Chronic Disease/ (6305)

38 Health service utilization.mp. (25)

39 lifestyle-related factor*.mp. (3)

40 or/18-39 (31309)

41 exp Bipolar Disorder/ (81)

42 exp Schizophrenia/ (931)

43 (schizo* or "mood disorder*" or "personality disorder*" or psychotic* or psychosis or psychoses).tw. (2312)

44 (bipolar or mania or Schizophrenia).tw. (1447)

45 (Depression adj2 (psychosis or psychotic or severe or major)).tw. (390)

46 exp Mental Disorders/ (27114)

47 mental health condition*.tw. (43)

48 exp Personality Disorders/ (323)

49 exp psychotic disorders/ (424)

50 ("severe mental illness" or "persistent mental illness").tw. (362)

51 ((chronic* or sever* or serious or persistent* or enduring or debilitating) adj2 (mental* or psychological*) adj2 (ill* or disorder* or health)).tw. (936)

52 (SPMI or SMI).tw. (79)

53 or/41-52 (27932)

54 (dementia or Alzheimer).ti. (1706)

55 (bipolar electrocoagulation or bipolar radiofrequency or bipolar tumour probe or bipolar diathermy).tw. (6)

56 ("respiratory depression" or "marrow depression" or "hematologic* depression").tw. (42)

57 (child* or adoles* or pediatric or paediatric).tw. (27044)

58 (Algeria$ or Egypt$ or Liby$ or Morocc$ or Tunisia$ or Western Sahara$ or Angola$ or Benin or Botswana$ or Burkina Faso or Burundi or Cameroon or Cape Verde or Central African Republic or Chad or Comoros or Congo or Djibouti or Eritrea or Ethiopia$ or Gabon or Gambia$ or Ghana or Guinea or Keny$ or Lesotho or Liberia or Madagasca$ or Malawi or Mali or Mauritania or Mauritius or Mayotte or Mozambiq$ or Namibia$ or Niger or Nigeria$ or Reunion or Rwand$ or Saint Helena or Senegal or Seychelles or Sierra Leone or Somalia or South Africa$ or Sudan or Swaziland or Tanzania or Togo or Ugand$ or Zambia$ or Zimbabw$ or China or Chinese or Hong Kong or Macao or Mongolia$ or Taiwan$ or Belarus or Moldov$ or Russia$ or Ukraine or Afghanistan or Armenia$ or Azerbaijan or Bahrain or Cyprus or Cypriot or Georgia$ or Iran$ or Iraq$ or Jordan$ or Kazakhstan or Kuwait or Kyrgyzstan or Leban$ or Oman or Pakistan$ or Palestin$ or Qatar or Saudi Arabia or Syria$ or Tajikistan or Turkmenistan or United Arab Emirates or Uzbekistan or Yemen or Bangladesh$ or Bhutan or British Indian Ocean Territory or Brunei Darussalam or Cambodia$ or India$ or Indonesia$ or Lao or People's Democratic Republic or Malaysia$ or Maldives or Myanmar or Nepal or Philippin$ or Singapore or Sri Lanka or Thai$ or Timor Leste or Vietnam or Albania$ or Andorra or Bosnia$ or Herzegovina$ or Bulgaria$ or Croatia$ or Faroe Islands or Greenland or Liechtenstein or Lithuani$ or Macedonia or Malta or maltese or Romania or Serbia$ or Montenegro or Svalbard or Argentina$ or Belize or Bolivia$ or Brazil$ or Chilean or Colombia$ or Costa Rica$ or Cuba or Ecuador or El Salvador or French Guiana or Guatemala$ or Guyana or Haiti or Honduras or Jamaica$ or Nicaragua$ or Panama or Paraguay or Peru or Puerto Rico or Suriname or Uruguay or Venezuela or developing countr$ or south America$).ti,sh. (9674)

59 or/54-58 (37507)

60 17 and 40 and 53 (230)

61 60 not 59 (193)

Proquest ASSIA

(("Dying soon" or "expected death" or "imminently dying" or Moribund) OR ("end-stage disease*" or "end stage disease*" or "end-stage ill*" or "end stage ill*" or end-stage or “end stage”) OR ("last year of life" or LYOL or "end of life" or "end of their lives" or "last six months of life" or "last 6 months of life") OR ("Irreversible condition" or "terminal condition" or "fatal illness") OR ("palliative care" or hospice or "end of life care" or end-of-life or "Terminal care" or "terminal* ill*")) AND ((Neoplasm* or cancer* or tumor* or tumour or neoplas* or malignan* or carcinoma* or

adenocarcinoma* or choriocrcinoma* or leukemia* or leukaemia* or metastat* or sarcoma* or teratoma* or lymphoma) OR ("duchenne muscular dystrophy" or "amyotrophic lateral sclerosis" or ALS) OR ("Genetic disease" or "genetic condition" or "cystis fibrosis" or CF) OR ("Organ failure" or "chronic organ failure" or "mulitple organ failure") OR (Chronic obstructive pulmonary disease or COPD or "renal insufficiency" or "serious physical illness" or "chronic medical condition") OR (chronic NEAR/3 ("cardiac failure" or "liver failure" or "kidney failure" or "end-stage renal disease" or ESRD or "renal failure" or "heart failure")) OR (chronic NEAR/3 (illness or condition or disease*))) AND ((Bipolar or schizo* or "mood disorder*" or "personality disorder*" or psychotic* or psychosis or psychoses) OR (Depression NEAR/2 (psychosis or psychotic or severe or major)) OR ("psychotic disorders" or "schizoaffective disorder" or "severe and persistent mental illness") OR ("psychotic disorders" or "schizoaffective disorder" or "Paranoid Disorders") OR ("severe mental illness" or "persistent mental illness" or SPMI or SMI))

CINAHL Search History

Select / deselect all

Search ID#	Search Terms	Search Options	Actions
S46	S15 AND S32 AND S45	Limiters - English Language; Age Groups: All Adult<br>Search modes - Boolean/Phrase	View Results (356) View Details Edit
S45	S33 OR S34 OR S35 OR S36 OR S37 OR S38 OR S39 OR S40 OR S41 OR S42 OR S43 OR S44	Search modes - Boolean/Phrase	View Results (124,935) View Details Edit
S44	AB SPMI or SMI	Search modes - Boolean/Phrase	View Results (1,211) View Details Edit
S43	AB (chronic* or sever* or serious or persistent* or enduring or debilitating) AND AB (mental* or psychological*) AND AB (ill* or disorder* or health)	Search modes - Boolean/Phrase	View Results (31,921) View Details Edit
S42	AB (chronic* or sever* or serious or persistent* or enduring or debilitating) AND AB (mental* or	Search modes - Boolean/Phrase	View Results (0)

		psychological*) adj2 (ill* or disorder* or health)		View Details Edit
☐	S41	AB (bipolar or mania or Schizophrenia) OR AB (psychotic* or psychosis or psychoses) OR AB (schizo* or "mood disorder*" or "personality disorder*") OR AB "mental health condition*" OR AB "severe mental illness" OR AB "persistent mental illness"	Search modes - Boolean/Phrase	View Results (38,951) View Details Edit
☐	S40	(TX "Mentally Ill person*")	Search modes - Boolean/Phrase	View Results (698) View Details Edit
☐	S39	(MH "Paranoid Disorders")	Search modes - Boolean/Phrase	View Results (617) View Details Edit
☐	S38	(MH "Psychotic Disorders")	Search modes - Boolean/Phrase	View Results (9,609) View Details Edit
☐	S37	(MH "Schizoaffective Disorder")	Search modes - Boolean/Phrase	View Results (223) View Details Edit
☐	S36	(MH "Personality Disorders")	Search modes - Boolean/Phrase	View Results (3,809) View Details Edit
☐	S35	(MH "Mental Disorders")	Search modes - Boolean/Phrase	View Results (48,711) View Details Edit
☐	S34	(MH "Schizophrenia")	Search modes - Boolean/Phrase	View Results (21,058) View Details

	S33	(MH "Bipolar Disorder")	Search modes - Boolean/Phrase	Edit View Results (9,957) View Details Edit
	S32	S16 OR S17 OR S18 OR S19 OR S20 OR S21 OR S22 OR S23 OR S24 OR S25 OR S26 OR S27 OR S28 OR S29 OR S30 OR S31	Search modes - Boolean/Phrase	View Results (444,331) View Details Edit
	S31	TX "lifestyle-related factor*"	Search modes - Boolean/Phrase	View Results (215) View Details Edit
	S30	(MH "Chronic Disease")	Search modes - Boolean/Phrase	View Results (53,902) View Details Edit
	S29	AB chronic N2 "cardiac failure" OR AB chronic N2 "liver failure" OR AB chronic N2 "kidney failure" OR AB chronic N2 "end-stage renal disease" OR AB chronic N2 ESRD OR AB chronic N2 "renal failure" OR AB chronic N2 "heart failure"	Search modes - Boolean/Phrase	View Results (4,975) View Details Edit
	S28	(MH "Liver Failure")	Search modes - Boolean/Phrase	View Results (1,681) View Details Edit
	S27	(MH "Renal Insufficiency")	Search modes - Boolean/Phrase	View Results (5,723) View Details Edit
	S26	(MH "Heart Failure")	Search modes - Boolean/Phrase	View Results (34,187) View Details Edit
	S25	(MH "Pulmonary Disease, Chronic Obstructive") OR	Search modes -	View

		(MH "Lung Diseases, Obstructive") OR (MH "Chronic Disease")	Boolean/Phrase	Results (72,636) View Details Edit
☐	S24	(MH "Multiple Organ Dysfunction Syndrome")	Search modes - Boolean/Phrase	View Results (2,454) View Details Edit
☐	S23	Multiple Organ Failure	Search modes - Boolean/Phrase	View Results (0) View Details Edit
☐	S22	(MH "Cystic Fibrosis")	Search modes - Boolean/Phrase	View Results (6,385) View Details Edit
☐	S21	(MH "Muscular Dystrophy, Duchenne")	Search modes - Boolean/Phrase	View Results (1,331) View Details Edit
☐	S20	(MH "Amyotrophic Lateral Sclerosis")	Search modes - Boolean/Phrase	View Results (3,299) View Details Edit
☐	S19	AB ("renal insufficiency" or "serious physical illness") OR AB (Chronic obstructive pulmonary disease or COPD) OR AB "chronic medical condition"	Search modes - Boolean/Phrase	View Results (14,172) View Details Edit
☐	S18	AB ("Enzymatic disease*" or "enzyme disease*") OR AB ("duchenne muscular dystrophy" or "amyotrophic lateral sclerosis" or ALS) OR AB ("Genetic disease" or "genetic condition") OR AB ("cystis fibrosis" or CF) OR AB ("Organ failure" or "chronic organ failure")	Search modes - Boolean/Phrase	View Results (12,244) View Details Edit
☐	S17	AB cancer* or tumo?r* or neoplas* or malignan* or carcinoma* or adenocarcinoma* or adeno?carcinoma* or choriocrcinoma* or leukemia* or leukaemia* or metastat* or sarcoma* or teratoma* or lymphoma	Search modes - Boolean/Phrase	View Results (274,177) View

				Details Edit
☐	S16	(MH "Neoplasms")	Search modes - Boolean/Phrase	View Results (67,564) View Details Edit
☐	S15	S1 OR S2 OR S3 OR S4 OR S5 OR S6 OR S7 OR S8 OR S9 OR S10 OR S11 OR S12 OR S13 OR S14	Search modes - Boolean/Phrase	View Results (159,654) View Details Edit
☐	S14	AB withdraw* N3 treat* OR AB withdraw* N3 car* OR AB withdraw* N3 intervene* OR AB withdraw* N3 therap* OR AB withdraw* N3 dialysis OR AB withdraw* N3 transplant*	Search modes - Boolean/Phrase	View Results (2,671) View Details Edit
☐	S13	AB withhold* N3 treat* OR AB withhold* N3 car* OR AB withhold* N3 intervene* OR AB withhold* N3 therap* OR AB withhold* N3 dialysis OR AB withhold* N3 transplant*	Search modes - Boolean/Phrase	View Results (561) View Details Edit
☐	S12	AB withdrew N3 treat* OR AB withdrew N3 car* OR AB withdrew N3 intervene* OR AB withdrew N3 dialysis OR AB withdrew N3 transplant* or AB withdrew N3 therap*	Search modes - Boolean/Phrase	View Results (185) View Details Edit
☐	S11	TX Refus* N3 treat* OR TX Refus* N3 care OR TX Refus* N3 intervention OR TX Refus* N3	Search modes - Boolean/Phrase	View Results (13,309) View Details Edit
☐	S10	TX conservative N2 treatment OR TX conservative N2 management OR TX "Withholding Treatment" OR TX "Treatment Refusal"	Search modes - Boolean/Phrase	View Results (20,188) View Details Edit
☐	S9	TX "Dying soon" or "expected death" or "imminently dying" or Moribund	Search modes - Boolean/Phrase	View Results (1,442) View Details Edit

☐	S8	TX ("end-stage disease*" or end stage disease* or end-stage ill* or "end stage ill*" or end-stage or "end stage") OR AB expected N3 die OR AB imminent N3 death	Search modes - Boolean/Phrase	View Results (23,677) View Details Edit
☐	S7	TX ("Irreversible condition" or "terminal condition" or "fatal illness") OR TX ("last year of life" or LYOL or "end of life" or "end of their lives") or TX ("last six months of life" or "last 6 months of life")	Search modes - Boolean/Phrase	View Results (37,619) View Details Edit
☐	S6	AB terminal* N3 care OR AB terminal* N3 caring OR AB terminal* N3 ill*	Search modes - Boolean/Phrase	View Results (3,888) View Details Edit
☐	S5	AB hospice N3 care OR AB hospice N3 caring OR AB hospice N3 ill*	Search modes - Boolean/Phrase	View Results (3,428) View Details Edit
☐	S4	TX "palliative care" or hospice or "end of life care" or end-of-life	Search modes - Boolean/Phrase	View Results (104,201) View Details Edit
☐	S3	(MH "Terminal Care") OR (MH "Terminally Ill Patients")	Search modes - Boolean/Phrase	View Results (23,777) View Details Edit
☐	S2	(MH "Hospice Care")	Search modes - Boolean/Phrase	View Results (7,894) View Details Edit
☐	S1	(MH "Palliative Care")	Search modes - Boolean/Phrase	

Search Name: COCHRANE Menloc final

Last Saved: 13/12/2018 09:55:54

Comment: after editing

ID Search

#1 MeSH descriptor: [Palliative Care] explode all trees

#2 MeSH descriptor: [Hospice Care] explode all trees

#3 MeSH descriptor: [Terminal Care] this term only

#4 MeSH descriptor: [Terminally Ill] this term only

#5 "palliative care" or hospice or "end of life care" or end-of-life

#6 "Irreversible condition" or "terminal condition" or "fatal illness"

#7 "last year of life" or LYOL or "end of life" or "end of their lives" or "last six months of life" or "last 6 months of life"

#8 end-stage disease* or end stage disease* or end-stage ill* or end stage ill* or end-stage or "end stage"

#9 imminent NEAR/3 death

#10 expected NEAR/3 die

#11 MeSH descriptor: [Conservative Treatment] this term only

#12 conservative NEAR/2 management

#13 conservative NEAR/2 treatment

#14 MeSH descriptor: [Withholding Treatment] explode all trees

#15 MeSH descriptor: [Treatment Refusal] explode all trees

#16 withdrew NEAR/3 treat*

#17 Withdrew NEAR/3 car*

#18 Withdrew NEAR/3 intervene*

#19 Withdrew NEAR/3 therap*

#20 Withdrew NEAR/3 dialysis

#21 #1 or #2 #3 or #4 or #5 or #6 or #7 or #8 or #9 or #10 or #11 or #12 or #13 or #14 or #15 or #16 or #17 or #18 or #19 or 20

#22 cancer* or tumo?r* or neoplas* or malignan* or carcinoma* or adenocarcinoma* or adeno?carcinoma* or choriocrcinoma* or leukemia* or leukaemia* or metastat* or sarcoma* or teratoma* or lymphoma

#23 MeSH descriptor: [Neoplasms] explode all trees

#24 MeSH descriptor: [Amyotrophic Lateral Sclerosis] explode all trees

#25 MeSH descriptor: [Genetic Diseases, Inborn] explode all trees

#26 MeSH descriptor: [Muscular Dystrophy, Duchenne] explode all trees

#27 MeSH descriptor: [Cystic Fibrosis] explode all trees

#28 MeSH descriptor: [Multiple Organ Failure] explode all trees

#29 MeSH descriptor: [Pulmonary Disease, Chronic Obstructive] explode all trees

#30 MeSH descriptor: [Heart Failure] explode all trees

#31 MeSH descriptor: [Renal Insufficiency] explode all trees

#32 MeSH descriptor: [Liver Failure] explode all trees

#33 MeSH descriptor: [Chronic Disease] explode all trees

#34 "duchenne muscular dystrophy" or "amyotrophic lateral sclerosis" or ALS

#35 "Genetic disease" or "genetic condition"

#36 chronic NEAR/2 ESRD

#37 "chronic illness" or "chronic condition" or "chronic disease*"

#38 "chronic medical condition"

#39 #22 or #23 or #24 or #25 or #26 or #27 or #28 or #29 or #30 or #31 or #32 or #33 or #34 or #35 or #36 or #37 or #38

#40 "severe mental illness" or "persistent mental illness"

#41 schizo* or "mood disorder*" or "personality disorder*" or psychotic* or psychosis or psychoses or SPMI or SMI

#42 bipolar or mania or Schizophrenia or psychosis

#43 Depression NEAR/2 psychosis

#44 Depression NEAR/2 psychotic

#45 Depression NEAR/2 severe

#46 Bipolar or "Mental Disorders" or "Personality Disorders"

#47 "psychotic disorders" or "schizoaffective disorder"

#48 "severe and persistent mental illness".

#49 #40 or #41 or #42 or #43 or #44 or #45 or #46 or #47 or #48

#50 #21 and #39 and #49

DARE 1998-2018

DARE and NHS EED archives secure on CRD website until at least 2021

("palliative care" or hospice or "end of life care" or end-of-life) and "severe mental illness" or " persistent mental illness".

Database: EMBASE <1947-Present>

Search Strategy:

----

1 exp palliative therapy/ (102069)

2 exp hospice care/ (9228)

3 exp terminal care/ (63131)

4 exp terminally ill patient/ (8148)

5 ("palliative care" or hospice or "end of life care" or end-of-life).tw. (65572)

6 ((hospice or terminal*) adj3 (care or caring or ill*)).tw. (17540)

7 ("Irreversible condition" or "terminal condition" or fatal illness).tw. (1173)

8 ("last year of life" or LYOL or "end of life" or "end of their lives" or "last six months of life" or "last 6 months of life").tw. (28590)

9 (end-stage disease* or end stage disease* or end-stage ill* or end stage ill* or end-stage or end stage).tw. (88414)

10 (expected adj3 die).tw. (306)

11 (imminent adj3 death).tw. (870)

12 ("Dying soon" or "expected death" or "imminently dying" or Moribund).tw. (3793)

13 conservative treatment/ (78244)

14 (conservative adj2 (treatment or management)).tw. (64421)

15 treatment withdrawal/ (17413)

16 treatment refusal/ (17138)

17 (Refus* adj3 (treat* or care or intervention or dialysis)).tw. (7265)

18 ((withdrew or withdraw* or withhold*) adj3 (treat* or car* or intervene* or therap* or dialysis or transplant*)).tw. (23578)

19 or/1-18 (420975)

20 exp neoplasm/ (4447791)

21 (cancer* or tumo?r* or neoplas* or malignan* or carcinoma* or adenocarcinoma* or adeno?carcinoma* or choriocrcinoma* or leukemia* or leukaemia* or metastat* or sarcoma* or teratoma* or lymphoma).tw. (4510708)

22 ("Enzymatic disease*" or "enzyme disease*").tw. (45)

23 genetic disorder/ (55815)

24 ("duchenne muscular dystrophy" or "amyotrophic lateral sclerosis" or ALS).tw. (55394)

25 amyotrophic lateral sclerosis/ (34903)

26 Duchenne muscular dystrophy/ (14824)

27 ("Genetic disease" or "genetic condition").tw. (10518)

28 cystic fibrosis/ (68731)

29 ("cystis fibrosis" or CF).tw. (62550)

30 exp multiple organ failure/ (35190)

31 ("Organ failure" or "chronic organ failure").tw. (27179)

32 chronic obstructive lung disease/ (115273)

33 ("renal insufficiency" or "serious physical illness").tw. (32224)

34 (Chronic obstructive pulmonary disease or COPD).tw. (93906)

35 (chronic adj3 (illness or condition or disease*)).tw. (374959)

36 "chronic medical condition".tw. (563)

37 exp kidney failure/ (343470)

38 exp heart failure/ (474239)

39 exp liver failure/ (70302)

40 (chronic adj2 ("cardiac failure" or "liver failure" or "kidney failure" or "end-stage renal disease" or ESRD or "renal failure" or "heart failure")).tw. (67182)

41 exp chronic disease/ (187803)

42 lifestyle-related factor*.mp. (445)

43 or/20-42 (6880626)

44 exp bipolar disorder/ (58962)

45 exp schizophrenia/ (184860)

46 (schizo* or "mood disorder*" or "personality disorder*" or psychotic* or psychosis or psychoses).tw. (279001)

47 (bipolar or mania or Schizophrenia).tw. (216783)

48 (Depression adj2 (psychosis or psychotic or severe or major)).tw. (39961)

49 exp mental disease/ (2123169)

50 mental health condition*.tw. (2502)

51 exp personality disorder/ (60988)

52 exp psychosis/ (290561)

53 exp schizoaffective psychosis/ (9378)

54 exp paranoid psychosis/ (18067)

55 "severe mental illness".tw. (4748)

56 " persistent mental illness".tw. (514)

57 ((chronic* or sever* or serious or persistent* or enduring or debilitating) adj2 (mental* or psychological*) adj2 (ill* or disorder* or health)).mp. (16645)

58 mental patient/ (27553)

59 (SPMI or SMI).tw. (5581)

60 or/44-59 (2207382)

61 (dementia or Alzheimer).ti. (70245)

62 (bipolar electrocoagulation or bipolar radiofrequency or bipolar tumour probe or bipolar diathermy).tw. (1641)

63 ("respiratory depression" or "marrow depression" or "hematologic* depression").tw. (10545)

64 (child* or adoles* or pediatric or paediatric).tw. (2085814)

65 (Algeria$ or Egypt$ or Liby$ or Morocc$ or Tunisia$ or Western Sahara$ or Angola$ or Benin or Botswana$ or Burkina Faso or Burundi or Cameroon or Cape Verde or Central African Republic or Chad or Comoros or Congo or Djibouti or Eritrea or Ethiopia$ or Gabon or Gambia$ or Ghana or Guinea or Keny$ or Lesotho or Liberia or Madagasca$ or Malawi or Mali or Mauritania or Mauritius or Mayotte or Mozambiq$ or Namibia$ or Niger or Nigeria$ or Reunion or Rwand$ or Saint Helena or Senegal or Seychelles or Sierra Leone or Somalia or South Africa$ or Sudan or Swaziland or Tanzania or Togo or Ugand$ or Zambia$ or Zimbabw$ or China or Chinese or Hong Kong or Macao or Mongolia$ or Taiwan$ or Belarus or Moldov$ or Russia$ or Ukraine or Afghanistan or Armenia$ or Azerbaijan or Bahrain or Cyprus or Cypriot or Georgia$ or Iran$ or Iraq$ or Jordan$ or Kazakhstan or Kuwait or Kyrgyzstan or Leban$ or Oman or Pakistan$ or Palestin$ or Qatar or Saudi Arabia or Syria$ or Tajikistan or Turkmenistan or United Arab Emirates or Uzbekistan or Yemen or Bangladesh$ or Bhutan or British Indian Ocean Territory or Brunei Darussalam or Cambodia$ or India$ or Indonesia$ or Lao or People's Democratic Republic or Malaysia$ or Maldives or Myanmar or Nepal or Philippin$ or Singapore or Sri Lanka or Thai$ or Timor Leste or Vietnam or Albania$ or Andorra or Bosnia$ or Herzegovina$ or Bulgaria$ or Croatia$ or Faroe Islands or Greenland or Liechtenstein or Lithuani$ or Macedonia or Malta or maltese or Romania or Serbia$ or Montenegro or Svalbard or Argentina$ or Belize or Bolivia$ or Brazil$ or Chilean or Colombia$ or Costa Rica$ or Cuba or Ecuador or El Salvador or French Guiana or Guatemala$ or Guyana or Haiti or Honduras or Jamaica$ or Nicaragua$ or

Panama or Paraguay or Peru or Puerto Rico or Suriname or Uruguay or Venezuela or developing countr$ or south America$).ti,sh. (1653512)

66 scientific literature/ (30246)

67 thesis.tw. (11296)

68 book.pt. (1180)

69 book/ (41676)

70 or/61-69 (3683087)

71 19 and 43 and 60 (13644)

72 71 not 70 (11727)

73 limit 72 to (human and english language and (adult <18 to 64 years> or aged <65+ years>)) (5427)

Database: Ovid MEDLINE(R) ALL <1946 to December 06, 2018>

Search Strategy:

----

1 exp Palliative care/ (49655)

2 exp Hospice care/ (5949)

3 exp Terminal Care/ (48040)

4 exp Terminally ill/ (6227)

5 ("palliative care" or hospice or "end of life care" or end-of-life).tw. (43896)

6 ((hospice or terminal*) adj3 (care or caring or ill*)).tw. (12766)

7 ("Irreversible condition" or "terminal condition" or fatal illness).tw. (791)

8 ("last year of life" or LYOL or "end of life" or "end of their lives" or "last six months of life" or "last 6 months of life").tw. (19924)

9 (end-stage disease* or end stage disease* or end-stage ill* or end stage ill* or end-stage or end stage).tw. (60633)

10 (expected adj3 die).tw. (215)

11 (imminent adj3 death).tw. (561)

12 ("Dying soon" or "expected death" or "imminently dying" or Moribund).tw. (2493)

13 conservative treatment/ (1401)

14 (conservative adj2 (treatment or management)).tw. (42831)

15 Withholding Treatment/ (10909)

16 Treatment Refusal/ (11477)

17 (Refus* adj3 (treat* or care or intervention or dialysis)).tw. (4855)

18 ((withdrew or withdraw* or withhold*) adj3 (treat* or car* or intervene* or therap* or dialysis or transplant*)).tw. (15455)

19 or/1-18 (244771)

20 exp Neoplasms/ (3107101)

21 (cancer* or tumo?r* or neoplas* or malignan* or carcinoma* or adenocarcinoma* or adeno?carcinoma* or choriocrcinoma* or leukemia* or leukaemia* or metastat* or sarcoma* or teratoma* or lymphoma).tw. (3243976)

22 ("Enzymatic disease*" or "enzyme disease*").tw. (25)

23 Genetic Disease, Inborn/ (13207)

24 ("duchenne muscular dystrophy" or "amyotrophic lateral sclerosis" or ALS).tw. (40207)

25 amyotrophic lateral sclerosis/ (16921)

26 Muscular dystrophy, Duchenne/ (4706)

27 ("Genetic disease" or "genetic condition").tw. (7305)

28 Cystic Fibrosis/ (33150)

29 ("cystis fibrosis" or CF).tw. (36968)

30 exp Multiple Organ Failure/ (10209)

31 ("Organ failure" or "chronic organ failure").tw. (17137)

32 Pulmonary Disease, Chronic Obstructive/ (34379)

33 ("renal insufficiency" or "serious physical illness").tw. (21134)

34 (Chronic obstructive pulmonary disease or COPD).tw. (54881)

35 (chronic adj3 (illness or condition or disease*)).tw. (250692)

36 "chronic medical condition".tw. (382)

37 Heart Failure/ (107446)

38 Renal Insufficiency/ (14803)

39 Liver Failure/ (6828)

40 (chronic adj2 ("cardiac failure" or "liver failure" or "kidney failure" or "end-stage renal disease" or ESRD or "renal failure" or "heart failure")).tw. (44143)

41 Chronic Disease/ (251517)

42 Health service utilization.mp. (1569)

43 lifestyle-related factor*.mp. (354)

44 or/20-43 (4717135)

45 exp Bipolar Disorder/ (37764)

46 exp Schizophrenia/ (99011)

47 (schizo* or "mood disorder*" or "personality disorder*" or psychotic* or psychosis or psychoses).tw. (197049)

48 (bipolar or mania or Schizophrenia).tw. (151754)

49 (Depression adj2 (psychosis or psychotic or severe or major)).tw. (28840)

50 exp Mental Disorders/ (1143274)

51 mental health condition*.tw. (1954)

52 exp Personality Disorders/ (39433)

53 exp psychotic disorders/ (48805)

54 exp schizoaffective disorder/ (48805)

55 exp psychosis/ (48805)

56 Paranoid Disorders/ (3987)

57 ("severe mental illness" or "persistent mental illness").tw. (3969)

58 ((chronic* or sever* or serious or persistent* or enduring or debilitating) adj2 (mental* or psychological*) adj2 (ill* or disorder* or health)).tw. (12556)

59 exp Mentally Ill Persons/ (5926)

60 (SPMI or SMI).tw. (3830)

61 or/45-60 (1239759)

62 (dementia or Alzheimer).ti. (52380)

63 (bipolar electrocoagulation or bipolar radiofrequency or bipolar tumour probe or bipolar diathermy).tw. (1123)

64 ("respiratory depression" or "marrow depression" or "hematologic* depression").tw. (6399)

65 (child* or adoles* or pediatric or paediatric).tw. (1520041)

66 (Algeria$ or Egypt$ or Liby$ or Morocc$ or Tunisia$ or Western Sahara$ or Angola$ or Benin or Botswana$ or Burkina Faso or Burundi or Cameroon or Cape Verde or Central African Republic or Chad or Comoros or Congo or Djibouti or Eritrea or Ethiopia$ or Gabon or Gambia$ or Ghana or Guinea or Keny$ or Lesotho or Liberia or Madagasca$ or Malawi or Mali or Mauritania or Mauritius or Mayotte or Mozambiq$ or Namibia$ or Niger or Nigeria$ or Reunion or Rwand$ or Saint Helena or Senegal or Seychelles or Sierra Leone or Somalia or South Africa$ or Sudan or Swaziland or Tanzania or Togo or Ugand$ or Zambia$ or Zimbabw$ or China or Chinese or Hong Kong or Macao or

Mongolia$ or Taiwan$ or Belarus or Moldov$ or Russia$ or Ukraine or Afghanistan or Armenia$ or Azerbaijan or Bahrain or Cyprus or Cypriot or Georgia$ or Iran$ or Iraq$ or Jordan$ or Kazakhstan or Kuwait or Kyrgyzstan or Leban$ or Oman or Pakistan$ or Palestin$ or Qatar or Saudi Arabia or Syria$ or Tajikistan or Turkmenistan or United Arab Emirates or Uzbekistan or Yemen or Bangladesh$ or Bhutan or British Indian Ocean Territory or Brunei Darussalam or Cambodia$ or India$ or Indonesia$ or Lao or People's Democratic Republic or Malaysia$ or Maldives or Myanmar or Nepal or Philippin$ or Singapore or Sri Lanka or Thai$ or Timor Leste or Vietnam or Albania$ or Andorra or Bosnia$ or Herzegovina$ or Bulgaria$ or Croatia$ or Faroe Islands or Greenland or Liechtenstein or Lithuani$ or Macedonia or Malta or maltese or Romania or Serbia$ or Montenegro or Svalbard or Argentina$ or Belize or Bolivia$ or Brazil$ or Chilean or Colombia$ or Costa Rica$ or Cuba or Ecuador or El Salvador or French Guiana or Guatemala$ or Guyana or Haiti or Honduras or Jamaica$ or Nicaragua$ or Panama or Paraguay or Peru or Puerto Rico or Suriname or Uruguay or Venezuela or developing countr$ or south America$).ti,sh. (1225437)

67 Academic Dissertations/ (0)

68 thesis.tw. (7167)

69 book.pt. (0)

70 Books/ (3174)

71 or/62-70 (2653676)

72 19 and 44 and 61 (2434)

73 72 not 71 (2126)

74 limit 73 to (english language and humans and "all adult (19 plus years)") (1217)

Database: PsycINFO <1806 to December Week 1 2018>

Search Strategy:

----

1 exp Palliative Care/ (11016)

2 Hospice/ (3062)

3 exp Terminally Ill Patients/ (4562)

4 exp HOSPICE/ (3062)

5 ("palliative care" or "hospice care" or "end of life care" or end-of-life).tw. (15589)

6 ((hospice or terminal*) adj3 (care or caring or ill*)).tw. (6333)

7 ("Irreversible condition" or "terminal condition" or fatal illness).tw. (216)

8 ("last year of life" or LYOL or "end of life" or "end of their lives" or "last six months of life" or "last 6 months of life").tw. (9038)

9 (end-stage disease* or end stage disease* or end-stage ill* or end stage ill* or end-stage or end stage).tw. (2002)

10 (expected adj3 die).tw. (44)

11 (imminent adj3 death).tw. (272)

12 ("Dying soon" or "expected death" or "imminently dying" or Moribund).tw. (235)

13 (conservative adj2 (treatment or management)).tw. (460)

14 Treatment Withholding/ (462)

15 Treatment Refusal/ (730)

16 (Refus* adj3 (treat* or care or intervention or dialysis)).tw. (1811)

17 ((withdrew or withdraw* or withhold*) adj3 (treat* or car* or intervene* or therap* or dialysis or transplant*)).tw. (3579)

18 or/1-17 (28620)

19 exp NEOPLASMS/ (47829)

20 (cancer* or tumo?r* or neoplas* or malignan* or carcinoma* or adenocarcinoma* or adeno?carcinoma* or choriocrcinoma* or leukemia* or leukaemia* or metastat* or sarcoma* or teratoma* or lymphoma).tw. (76722)

21 ("Enzymatic disease*" or "enzyme disease*").tw. (0)

22 Genetic Disorders/ (4048)

23 ("duchenne muscular dystrophy" or "amyotrophic lateral sclerosis" or ALS).tw. (13477)

24 Amyotrophic Lateral Sclerosis/ (3551)

25 Muscular Dystrophy/ (1293)

26 ("Genetic disease" or "genetic condition").tw. (611)

27 Cystic Fibrosis/ (854)

28 ("cystis fibrosis" or CF).tw. (3217)

29 "Multi* Organ Failure*".tw. (71)

30 ("Organ failure" or "chronic organ failure").tw. (203)

31 exp Chronic Obstructive Pulmonary Disease/ (1272)

32 ("renal insufficiency" or "serious physical illness").tw. (319)

33 (Chronic obstructive pulmonary disease or COPD).tw. (2277)

34 (chronic adj3 (illness or condition or disease*)).tw. (28394)

35 "chronic medical condition".tw. (196)

36 Heart Disorders/ (8989)

37 Kidney Diseases/ (2001)

38 Liver Disorders/ (1145)

39 (chronic adj2 ("cardiac failure" or "liver failure" or "kidney failure" or "end-stage renal disease" or ESRD or "renal failure" or "heart failure")).tw. (866)

40 Chronic Illness/ (10804)

41 lifestyle-related factor*.mp. (51)

42 or/19-41 (139563)

43 exp Bipolar Disorder/ (25413)

44 exp SCHIZOPHRENIA/ (85758)

45 (schizo* or "mood disorder*" or "personality disorder*" or psychotic* or psychosis or psychoses).tw. (208100)

46 (bipolar or mania or Schizophrenia).tw. (137089)

47 (Depression adj2 (psychosis or psychotic or severe or major)).tw. (34922)

48 exp Mental Disorders/ (566262)

49 mental health condition*.tw. (1698)

50 exp Personality Disorders/ (33638)

51 exp Schizoaffective Disorder/ (2961)

52 exp Psychosis/ (109879)

53 "Paranoia (Psychosis)"/ (1215)

54 "Severity (Disorders)"/ (15885)

55 "severe mental illness".tw. (4681)

56 "persistent mental illness".tw. (799)

57 ((chronic* or sever* or serious or persistent* or enduring or debilitating) adj2 (mental* or psychological*) adj2 (ill* or disorder* or health)).tw. (16725)

58 Mentally Ill Persons.mp. (805)

59 (SPMI or SMI).tw. (1991)

60 or/43-59 (637058)

61 (dementia or Alzheimer).ti. (30336)

62 (bipolar electrocoagulation or bipolar radiofrequency or bipolar tumour probe or bipolar diathermy).tw. (5)

63 ("respiratory depression" or "marrow depression" or "hematologic* depression").tw. (407)

64 (child* or adoles* or pediatric or paediatric).tw. (811926)

65 (Algeria$ or Egypt$ or Liby$ or Morocc$ or Tunisia$ or Western Sahara$ or Angola$ or Benin or Botswana$ or Burkina Faso or Burundi or Cameroon or Cape Verde or Central African Republic or Chad or Comoros or Congo or Djibouti or Eritrea or Ethiopia$ or Gabon or Gambia$ or Ghana or Guinea or Keny$ or Lesotho or Liberia or Madagasca$ or Malawi or Mali or Mauritania or Mauritius or Mayotte or Mozambiq$ or Namibia$ or Niger or Nigeria$ or Reunion or Rwand$ or Saint Helena or Senegal or Seychelles or Sierra Leone or Somalia or South Africa$ or Sudan or Swaziland or Tanzania or Togo or Ugand$ or Zambia$ or Zimbabw$ or China or Chinese or Hong Kong or Macao or Mongolia$ or Taiwan$ or Belarus or Moldov$ or Russia$ or Ukraine or Afghanistan or Armenia$ or Azerbaijan or Bahrain or Cyprus or Cypriot or Georgia$ or Iran$ or Iraq$ or Jordan$ or Kazakhstan or Kuwait or Kyrgyzstan or Leban$ or Oman or Pakistan$ or Palestin$ or Qatar or Saudi Arabia or Syria$ or Tajikistan or Turkmenistan or United Arab Emirates or Uzbekistan or Yemen or Bangladesh$ or Bhutan or British Indian Ocean Territory or Brunei Darussalam or Cambodia$ or India$ or Indonesia$ or Lao or People's Democratic Republic or Malaysia$ or Maldives or Myanmar or Nepal or Philippin$ or Singapore or Sri Lanka or Thai$ or Timor Leste or Vietnam or Albania$ or Andorra or Bosnia$ or Herzegovina$ or Bulgaria$ or Croatia$ or Faroe Islands or Greenland or Liechtenstein or Lithuani$ or Macedonia or Malta or maltese or Romania or Serbia$ or Montenegro or Svalbard or Argentina$ or Belize or Bolivia$ or Brazil$ or Chilean or Colombia$ or Costa Rica$ or Cuba or Ecuador or El Salvador or French Guiana or Guatemala$ or Guyana or Haiti or Honduras or Jamaica$ or Nicaragua$ or Panama or Paraguay or Peru or Puerto Rico or Suriname or Uruguay or Venezuela or developing countr$ or south America$).ti,sh. (128851)

66 thesis.tw. (24537)

67 book.pt. (476469)

68 exp BOOKS/ (6110)

69 or/61-68 (1332511)

70 18 and 42 and 60 (838)

71 70 not 69 (609)

# 11	105	#10 AND #7 AND #6<br>Indexes=SCI-EXPANDED, SSCI, CPCI-S, CPCI-SSH<br>Timespan=All years
# 10	394,440	#9 OR #8<br>Indexes=SCI-EXPANDED, SSCI, CPCI-S, CPCI-SSH<br>Timespan=All years
# 9	388,426	TS=(Bipolar or schizo* or "mood disorder*" or "personality disorder*" or psychotic* or psychosis or psychoses) OR TS=("psychotic disorders" or "schizoaffective disorder" or "severe and persistent mental illness")<br>Indexes=SCI-EXPANDED, SSCI, CPCI-S, CPCI-SSH<br>Timespan=All years
# 8	22,785	**TOPIC:** ("schizoaffective disorder" or "severe and persistent mental illness" OR "psychotic disorders" or "schizoaffective disorder" or "Paranoid Disorders" OR "severe mental illness" or "persistent mental illness" or SPMI or SMI)

		Indexes=SCI-EXPANDED, SSCI, CPCI-S, CPCI-SSH Timespan=All years
# 7	4,104,078	**TOPIC:** (Neoplasm* or cancer* or tumor* or tumour or neoplas* or malignan* or carcinoma* or adenocarcinoma* or choriocrcinoma* or leukemia* or leukaemia* or metastat* or sarcoma* or teratoma* or lymphoma) *OR* **TOPIC:** ("duchenne muscular dystrophy" or "amyotrophic lateral sclerosis" or ALS) *OR* **TOPIC:** ("Genetic disease" or "genetic condition" or "cystis fibrosis" or CF) *OR* **TOPIC:** ("Organ failure" or "chronic organ failure" or "mulitple organ failure") *OR* **TOPIC:** (Chronic obstructive pulmonary disease or COPD or "renal insufficiency" or "serious physical illness" or "chronic medical condition") Indexes=SCI-EXPANDED, SSCI, CPCI-S, CPCI-SSH Timespan=All years
# 6	109,890	#5 OR #4 OR #3 OR #2 OR #1 Indexes=SCI-EXPANDED, SSCI, CPCI-S, CPCI-SSH Timespan=All years
# 5	2,468	**TOPIC:** ("Dying soon" or "expected death" or "imminently dying" or Moribund) Indexes=SCI-EXPANDED, SSCI, CPCI-S, CPCI-SSH Timespan=All years
# 4	56,678	**TOPIC:** ("end-stage disease*" or "end stage disease*" or "end-stage ill*" or "end stage ill*" or end-stage or “end stage”) Indexes=SCI-EXPANDED, SSCI, CPCI-S, CPCI-SSH Timespan=All years
# 3	24,428	**TOPIC:** ("last year of life" or LYOL or "end of life" or "end of their lives" or "last six months of life" or "last 6 months of life") Indexes=SCI-EXPANDED, SSCI, CPCI-S, CPCI-SSH Timespan=All years
# 2	773	**TOPIC:** ("Irreversible condition" or "terminal condition" or "fatal illness") Indexes=SCI-EXPANDED, SSCI, CPCI-S, CPCI-SSH Timespan=All years
# 1	54,014	**TOPIC:** ("palliative care" or hospice or "end of life care" or end-of-life or "terminal care") Indexes=SCI-EXPANDED, SSCI, A&HCI, CPCI-S, CPCI-SSH, ESCI Timespan=All years

Other sources

Trial Registers	Search terms
ClinicalTrials.gov	"end of life" or palliative and mental
metaRegister of Controlled Trials (mRCT)	Not Applicable
UK Clinical Trials Gateway (UKCTG)	for "last year of life" or LYOL or "end of life" or "end of their lives" in mental
WHO ICTRP Search Portal International	"end of life" or "end of their lives" and

	mental

Charity websites	Search terms
Cancer Research UK	**"end of life" and "mental illness"**
Cancer Research Wales	"end of life" and "mental illness"
Tenovus Cancer Care	"end of life" and "mental illness"
Mental Health Foundation	Reports identified browsing publications page
Centre for Mental Health	Searched publications – Mental health Policy https://www.centreformentalhealth.org.uk/publications
National Kidney Foundation	**"end of life" and "mental illness"**
British Liver Trust	**"end of life" and "mental illness"**
British Renal Society	Scanning BRS conference abstract page
The Renal Association	**"end of life" and "mental illness"**
British Heart Foundation	**"last year of life" or LYOL or "end of life" or "end of their lives" and "severe mental" and Healthcare professionals**
British Lung Foundation	**"end of life" and "mental illness"**
National Council for Palliative Care	"end of life care" and mental
Hospice UK	"end of life care" and mental
Marie Curie	"end of life care" and "mental illness" and evaluation
Macmillan Cancer Support	**"last year of life" or LYOL or "end of life" or "end of their lives" and "severe mental"**
Scottish Partnership agency for Palliative Care	"end of life care" and "mental illness" and evaluation
Bipolar UK	"end of life care" and "mental illness" and evaluation
MIND	"end of life care" and "mental illness"
Rethink Mental Illness	"end of life care" and "mental illness"
Sova	"end of life care" and mental
Mental Health UK	No search facility or publications page
Heads Together	No search facility or publications page

Organisation Websites	Search terms
NHS England	"end of life care" and "mental illness" and evaluation
NHS Wales	"end of life care" and "severe mental" and evaluation
Care Inspectorate Wales	"end of life care" and "severe mental"
English CQC	**"end of life" and "severe mental" in Publications**
Ministry of Justice	"end of life care" and "mental illness" and evaluation

Journals	Search terms

Journal of Pain and Symptom Management	**"end of life care"** in *Title/Abs/Keywords* OR **"palliative care"** in *Title/Abs/Keywords* AND **Mental** in *Title/Abs/Keywords*<br>2017-2019
Cancer	"end of life" and mental or palliative and mental 2017-2019
Psycho-Oncology	"end of life" and mental (2017-2019) or palliative and mental (2017-2018)
BMJ Supportive & Palliative Care	""mental illness"" and published between "01 Jan, 2017 and 21 Feb, 2019""mental" and published between "01 Jan, 2017 and 21 Feb, 2019
