# Supplementary material for: End-of-life care for people with severe mental illness: mixed methods systematic review and thematic synthesis of published case studies (the MENLOC study)
Source: BMJ Open. 2022 Feb 22;12(2):e053223. doi: 10.1136/bmjopen-2021-053223 (PMC8867317; doi:10.1136/bmjopen-2021-053223)
Supplement: Supplementary data [file bmjopen-2021-053223supp004.pdf]

Supplementary searches

Trial Registers	Search terms
ClinicalTrials.gov	"end of life" or palliative and mental
metaRegister of Controlled Trials (mRCT)	Not Applicable
UK Clinical Trials Gateway (UKCTG)	"last year of life" or LYOL or "end of life" or "end of their lives" in mental
WHO ICTRP Search Portal International<br>https://www.who.int/ictrp/en/	"end of life" or "end of their lives" and mental

Charity websites	Search type	Search terms
1. Cancer Research UK<br>http://www.cancerresearchuk.org/	Electronic search: Main website	"end of life" and "mental illness/health"<br>"palliative care" and mental illness/health"
2. Cancer Research Wales<br>http://www.cancerresearchuk.org/	Electronic search: Main website	"end of life" and "mental illness/health"<br>"palliative care" and mental illness/health"
3. Tenovus Cancer Care<br>http://www.tenovuscancercare.org.uk/	Electronic search: Main website	"end of life" and "mental illness"
4. Mental Health Foundation<br>https://www.mentalhealth.org.uk/	Electronic search: Main website<br><br>Manual search: Policy publications	"end of life" and "mental illness/health"<br>"palliative care" and mental illness/health"<br>As per reviews inclusion criteria
5. Centre for Mental Health<br>https://www.centreformentalhealth.org.uk/	Electronic search: Policy publications	"end of life" and "mental illness/health"

		"palliative care" and mental illness/health"
6. National Kidney Foundation<br>https://www.kidney.org/	Electronic search: Main website<br><br>Manual search: Policy publications	"end of life" and "mental illness/health"<br>"palliative care" and mental illness/health"<br>As per reviews inclusion criteria
7. British Liver Trust<br>https://www.britishlivertrust.org.uk/	Electronic search: Publications	"end of life" and "mental illness"<br>"palliative care" and mental illness"<br>(mental health too broad a term)
8. British Renal Society<br>http://britishrenal.org/	Electronic search: Conference abstracts	"end of life" and "mental illness/health"<br>"palliative care" and mental illness/health"
9. The Renal Association<br>http://britishrenal.org/	Electronic search: Main website	"end of life" and "mental illness/health"<br>"palliative care" and mental illness/health"
10. British Heart Foundation<br>http://britishrenal.org/	Electronic search: Main website	“last year of life" or LYOL or "end of life" or "end of their lives" and “severe mental” and Healthcare professionals<br>"palliative care" and mental illness"<br>(mental health too broad)
11. British Lung Foundation<br>https://www.blf.org.uk/	Electronic search: Main website	"end of life" and "mental illness/health"<br>"palliative care" and mental illness/health"
12. Hospice UK<br>http://www.hospiceuk.org/	Electronic search: Main website	"end of life" and "mental illness/health"<br>"palliative care" and mental illness/health"
13. Marie Curie<br>https://www.mariecurie.org.uk/	Electronic search: Main website	"end of life" and "mental illness/health"<br>"palliative care" and mental illness/health"

	Manual search: Briefings	As per reviews inclusion criteria
14. Macmillan Cancer Support https://www.macmillan.org.uk/	Electronic search: Main website	“last year of life" or LYOL or "end of life" or "end of their lives" and “severe mental” "palliative care" and mental illness/health"
15. Bipolar UK https://www.bipolaruk.org/	Electronic search: Main website	"end of life" and "mental illness/health" Palliative
16. MIND https://www.mind.org.uk/	Electronic search: Main website	"end of life care" and "mental illness/health" Palliative
17. Rethink Mental Illness https://www.rethink.org/	Electronic search: Main website	"end of life care" and "mental illness/health" Palliative
18. Sova https://www.sova.org.uk/	Electronic search: Main website	"end of life care" and mental Palliative
19. Hafal: for recovery for serious mental illness www.hafal.org	Electronic search: Main website	"end of life care" and mental Palliative and mental
20. Age Concern https://www.ageuk.org.uk	Electronic search: Main website	"end of life care " and mental Palliative and mental
21. Salvation Army https://salvationarmy.org.uk	Electronic search: Main website	"end of life care" and mental Palliative and mental
22. Dying Matters https://www.dyingmatters.org/	Electronic search: Main website	"end of life " and mental "palliative care" and mental illness/health"
23. Care Not Killing https://www.carenotkilling.org.uk/	Electronic search: Main website	"end of life care" and then searched within results for the term mental

		"palliative care" and then searched within results for the term mental
24. Northern Ireland Hospice https://www.nihospice.org/	Electronic search: Main website	"end of life care" and mental<br>Palliative
25. Mental Health UK https://www.mentalhealth-uk.org/	No search facility or publications page	
26. Heads Together https://www.headstogether.org.uk/	No search facility or publications page	
27. Kidney Research UK https://www.kidneyresearchuk.org/	Electronic search: Main website	"end of life care" and mental<br>Palliative
28. National Kidney Federation https://www.kidney.org.uk/	Electronic search: Main website	"end of life care" and mental<br>Palliative
29. St Mungos https://www.mungos.org/	Electronic search: Main website	"end of life "<br>Palliative<br>“mental illness”
30. Samaritans https://www.samaritans.org/	Electronic search: Main website	"end of life" and “severe mental”<br>Palliative and “severe mental”
31. Llanmau https://www.llamau.org.uk/	Electronic search: Main website	
32. SSAFA https://www.ssafa.org.uk/	Electronic search: Main website	
33. Community Housing Cymru: https://chcymru.org.uk/	Electronic search: Main website	
34. National Housing Federation: https://www.housing.org.uk/	Electronic search: Main website	
35. Shelter Cymru https://sheltercymru.org.uk/	Electronic search: Main website	

36. Shelter<br>https://www.shelter.org.uk/	Electronic search: Main website	
37. Gofal<br>http://www.gofal.org.uk/	No search function checked projects & services page but no relevant reports.	As per reviews inclusion criteria
38. Compassionate communities<br>https://www.compassionate-communitiesuk.co.uk/	No search function checked projects but no relevant reports	As per reviews inclusion criteria
39. Byw Nawr<br>https://www.dyingmatters.org/wales	Electronic search: Main website	
40. Combat Stress<br>https://www.combatstress.org.uk/	Electronic search: Main website	
41. Royal British Legion<br>https://www.britishlegion.org.uk/	Electronic search: Main website	

Organisation Websites	Search type	Search terms
1. NHS England<br>https://www.england.nhs.uk/cancer/strategy/<br>https://www.england.nhs.uk/eolc/resources/ -<br>http://endoflifecareambitions.org.uk/	Electronic search: Main website	"mental illness"
2. NHS Wales<br>http://www.wales.nhs.uk/	Electronic search: Main website<br>Advanced search: title field only	"end of life" and "mental illness/health"<br>"palliative care" and mental illness/health"
3. Welsh Government<br>https://gov.wales/	Electronic search: Main website<br>Advanced search<br>Limited to Health and social services	"end of life" and "mental illness/health"<br>"palliative care" and mental illness/health"
4. Department of Health and Social Care<br>https://www.gov.uk/	Electronic search: Publications<br>Limited by:	"end of life" and "mental illness/health"<br>"palliative care" and mental illness/health"

	Department of Health and Social Care<br>Topic: Health and Social Care<br>Sub topic: End of Life	
5. Department of Health: Northern Ireland		"end of life" and "mental illness/health"<br>"palliative care" and mental illness/health"
6. Department of Health, Social Services and Public Safety: Northern Ireland		"end of life" and "mental illness/health"<br>"palliative care" and mental illness/health"
7. Ministry of Justice<br>https://www.gov.uk/	Electronic search: Publications<br>Limited by: Ministry of Justice<br>Topic: Health and Social Care<br>Sub Topic: End of Life	"end of life" and "mental illness/health"<br>"palliative care" and mental illness/health"
8. Care Inspectorate Wales<br>https://www.qcs.co.uk/wales/	Searching only available with practitioner registration<br>Emailed – no relevant publications	
9. Care Quality Commission<br>https://www.cqc.org.uk/	Electronic search: Publications	"end of life" and “severe mental”<br>"palliative care" and "mental illness"
10. Prisons and Probation Ombudsman<br>https://www.ppo.gov.uk	Electronic search: Main website<br>Manual search: Fatal incident reports over a one year period between June 2017-18.<br>Limited by: Deaths of natural causes	"end of life"<br>As per reviews inclusion criteria
11. Royal College of Psychiatrists<br>https://www.rcpsych.ac.uk/	Manual search: College Reports	As per reviews inclusion criteria
12. Royal College of Physicians<br>https://www.rcplondon.ac.uk/	Electronic search: Main website	"end of life" and "mental illness/health"<br>"palliative care" and mental illness/health"
13. The Worldwide Hospice and Palliative Care Alliance	Manual search: Resources	As per reviews inclusion criteria

http://www.thewhpca.org/		
14. European Association of Palliative Care<br>https://www.eapcnet.eu/	Manual search: National Guidelines<br>(UK Countries only)	As per reviews inclusion criteria
15. Scottish Partnership agency for Palliative Care<br>https://www.palliativecarescotland.org.uk/	Electronic search: Main website	"end of life care" and "mental illness" and evaluation<br>"palliative care" and mental illness/health"
16. National Council for Palliative Care<br>http://www.ncpc.org.uk/	Electronic search: Main website	"end of life" and "mental illness/health"<br>"palliative care" and mental illness/health""
17. Social Care Institute for Excellence<br>https://www.scie.org.uk/	Electronic search: Main website	"end of life" and "mental illness"<br>"palliative care" and mental illness"
18. Health Improvement Scotland<br>http://www.healthcareimprovementscotland.org/<br>Covers publications by NHS Scotland, Scottish Government and Health Improvement Scotland	Electronic search: Main website	"end of life" and "mental illness"<br>"palliative care" and mental illness"
19. National Institute of Clinical Excellence.<br>https://www.nice.org.uk/guidance	Electronic search: Main website	"end of life" or "palliative care"<br>“mental illness"
20. Royal College of Nursing<br>https://www.rcn.org.uk/clinical-topics/end-of-life-care	Manual search through section on Clinical Topics End of Life	As per reviews inclusion criteria
21. Gold Standards Framework<br>http://www.goldstandardsframework.org.uk/	Manual search through library resources	As per reviews inclusion criteria

**Hand searching journals**	**Search terms**
Journal of Pain and Symptom Management	"end of life care" in *Title/Abs/Keywords* OR "palliative care" in *Title/Abs/Keywords* AND Mental in *Title/Abs/Keywords* 2017-2019
Cancer	"end of life" and mental or palliative and mental 2017-2019
Psycho-Oncology	"end of life" and mental (2017-2019) or palliative and mental (2017-2018)
BMJ Supportive & Palliative Care	""mental illness"" and published between "01 Jan, 2017 and 21 Feb, 2019""mental" and published between "01 Jan, 2017 and 21 Feb, 2019

Google search using the terms "palliative care" and "mental illness" (searching first 10 pages of output)

Output	Action	
Naylor et al 2016. Bringing together physical and mental health: A new frontier for integrated care. Available from: https://www.kingsfund.org.uk/publications/physical-and-mental-health	Searched and added to table	Relevant report
Help the Hospices. 2016 Hospice and palliative care – Access for All. Available from: http://nican.hscni.net/files/hospice_and_palliative_care_access_for_all.pdf Accessed 31 January 2019	Searched and added to table	Relevant report
Ulster University Repository<br>Walsh, S. Sheridan, A. Leavey, G. Coughlan, B. Frazer, K. O'Toole, S. Kemple, M. Crawley, L. (2014) Identifying and addressing the palliative care needs of people with serious mental illness in Ireland. Irish College of General Practitioners, 31(7). p.1<br><br>Sheridan, A. Couglan, B. Frazer, K. Walsh, S. Bergin, J. A. Crawley, L. Kemple, M. O'Toole, S. (n.d.)The palliative care needs of people with serious mental illness in Ireland. http://www.professionalpalliativehub.com/sites/default/files/Paliative%20Care%20need%20of%20People%20with%20SMI%20June%2015%202017%20Report%20Draft.pdf	Screened<br><br>Email sent to author 20-02-2019 via researchers gate for date of publication	Relevant paper

NHS Lothian. 2010. Living and dying well in Lothian. Available from: https://www.nhslothian.scot.nhs.uk/OurOrganisation/Strategies/ladwinlothian/Documents/Palliative%20Care%20Strategy%202010%20-15%20VER%2023%20FINAL.pdf	Searched and added to table	Relevant Report
alex.mathew@wales.nhs.uk. End of life care for older people with schizophrenia and bipolar affective disorder along with long term physical conditions: a phenomenological enquiry into the experiences of bereaved family carers, mental health and social care providers in South Wales-UK	Email sent 20th Feb 2019	NOT RELEVANT
Queens University. Belfast Repository Millman, J. Galway, K. Santin, O. Reid J. (2016) Cancer and serious mental illness – patient, caregiver and professional perspectives: study protocol. Journal of Advanced Nursing 72(1), 217–226	Author search conducted on PubMed and full study findings not yet published	NOT RELEVANT
Bloomer, M. J. O'Brien, A. P. (2013) Palliative care for the person with a serious mental illness: The need for a partnership approach to care in Australia, Progress in Palliative Care, 21:1, 27-31	Screened	Not relevant

Google search using the terms “end of life” and “mental illness” (searching first 10 pages of output)

Output	Action	
Blog post Paul Gionfriddo and Nathaniel Counts “Providing end-of-life support for elders with serious mental illnesses" Health Affairs Blog, November 16, 2017.DOI: 10.1377/hblog20171114.475106	Screened	1. Not Relevant
Doering, P. End of life care and serious persistent mental illness. Regional Palliative Care Services. June 2018, Issue 42, p. 1-3 https://www.northernhealth.ca/sites/northern_health/files/health-professionals/palliative-care/documents/serious-persistent-mental-illness-issue-42.pdf	Screened	2. Not relevant
Ahearn 2015. The loneliness of mental illness at the end of life.	Screened	3. Not relevant

https://www.wisconsinmedicalsociety.org/_WMS/publications/wmj/pdf/117/3/101.pdf		
WA Cancer and Palliative Care Network 2018. WA End-of-Life and Palliative Care Strategy 2018–2028. Available from: https://ww2.health.wa.gov.au/~/media/Files/Corporate/general%20documents/Health%20Networks/Palliative%20care/WA%20End-of-life%20and%20Palliative%20Care%20Strategy%202018-2028.pdf	Screened	Not relevant
National Association of Social workers 2004. NASW Standards for Palliative & End of Life Care. Available from: https://www.socialworkers.org/LinkClick.aspx?fileticket=xBMd58VwEhk%3D&portalid=0	Screened	Not relevant
NSW Ministry of Health 2015. Dignity, Respect and Choice: Advance Care Planning for End of Life for People with Mental Illness - A Comprehensive Guide. Available at: https://www.health.nsw.gov.au/patients/acp/Pages/comprehensive-guide.aspx. NSW Government	Screened	Not relevant

Google search using the terms using the terms “end of life” and schizophrenia (searching first 5 pages of output)

Output	Action	
Steves and Williams 2016<br>Enhancing end-of-life care for terminally ill psychiatric patients<br>Nursing, 46(8): 54-8	screened	8. Relevant

Google search using the terms “end of life” and bipolar (searching first 5 pages of output)

Output	Action	
Maloney, K. Opinion: My husband refused to believe he had bipolar disorder, and denial destroyed him. May 15th 2014 https://www.thejournal.ie/readme/bipolar-ii-disorder-denial-mental-health-1467162-May2014/	Screened	Relevant

Journal articles and reports suggested by stakeholder advisory members

Output	Action	
Kurella, M. Kimmel, P. L. Young, B.S. Chertow, G. M.<br>Suicide in the United States end-stage renal disease program. Journal of the American Society of Nephrology. 2005: 16(3): 774-81 https://jasn.asnjournals.org/content/16/3/774.full	Screened	1. Not relevant
Liu, C-H. Yeh, M-K. Weng, S-C. Bai, M-Y. Chang, J-C.<br>Suicide and chronic kidney disease: a case-control study. Nephrology Dialysis Transplantation. 2017: 2(9): 1524–29	Screened	2. Not relevant
Prison and Probation Ombudsman for England and Wales 2013<br>Learning from PPO Investigations. End of Life Care	Searched and added to table	3. Not relevant
Prison and Probation Ombudsman for England and Wales 2019<br>Fatal Incident Reports. https://www.ppo.gov.uk/document/fii-report/	Searched through 2 years' worth of reports where death was from natural causes 4th February 2019	4. UNSURE
All Wales Palliative Care Planning Group 2008<br>Report to Minister for Health and Social services on Palliative care Services – Sugar Report<br>www,wales.nhs.uk/documents/palliativecarereport.pdf	Searched and added to table	5. Not relevant
Secretariat of the Commission of the Bishops' Conferences of the European Community 2016<br>Opinion of the Working Group on Ethics in Research and Medicine On Palliative Care in the European Union<br>http://www.comece.eu/dl/psqlJKJKooNNJqx4KJK/PalliativeCARE_EN.pdf	Searched and added to table	6. Not relevant
Addicott, R. Ashton, R. 2010<br>Delivering Better Care at the End of Life: The next steps. The King's Fund	Searched and added to table	7. Not relevant

https://www.kingsfund.org.uk/sites/default/files/Delivering-better-care-end-of-life-Kings-Fund-January-2010-Leeds-Castle-EOLC.pdf		
Addicott, R. Hiley, J. 2011<br>Issues Facing Commissioners in End-of-Life Care. London: The King's Fund<br>https://www.kingsfund.org.uk/sites/default/files/issues-facing-commissioners-end-of-life-care-report-september2011.pdf	Searched and added to table	8. Not relevant
Addicott R, Ross S. 2010<br>Implementing the End of Life Care Strategy. Lessons for Good Practice. The Kings Fund<br>https://www.kingsfund.org.uk/sites/default/files/field/field_publication_file/Implementing-end-of-life-care-Rachael-Addicott-Shilpa-Ross-Kings-Fund-October2010_0.pdf	Searched and added to table	9. Not relevant
General Medical Council 2010<br>Treatment and Care Towards the End of Life: Good Practice in Decision Making<br>https://www.gmc-uk.org/-/media/documents/treatment-and-care-towards-the-end-of-life---english-1015_pdf-48902105.pdf	Searched and added to table	10. Not relevant
Hall, S. Petkova, H. Tsouros, A. D. Costantini, M. Higginson, I. G. 2011<br>Palliative Care for Older People: Better Practices. Copenhagen WHO: Regional Office for Europe.<br>http://www.euro.who.int/__data/assets/pdf_file/0017/143153/e95052.pdf	Searched and added to table	11. Not relevant
