# Supplementary material for: End-of-life care for people with severe mental illness: mixed methods systematic review and thematic synthesis of published case studies (the MENLOC study)
Source: BMJ Open. 2022 Feb 22;12(2):e053223. doi: 10.1136/bmjopen-2021-053223 (PMC8867317; doi:10.1136/bmjopen-2021-053223)
Supplement: Supplementary data [file bmjopen-2021-053223supp005.pdf]

THE JOANNA BRIGGS INSTITUTE

# JBI Critical Appraisal Checklist for Case Reports

| | |
|---|---|
| Reviewer | |
| Date reviewed | |
| Author | |
| Year | |

| | Yes | No | Unclear | Not applicable |
|---|---|---|---|---|
| 1. Were patient's demographic characteristics clearly described? | ☐ | ☐ | ☐ | ☐ |
| 2. Was the patient's history clearly described and presented as a timeline? | ☐ | ☐ | ☐ | ☐ |
| 3. Was the current clinical condition of the patient on presentation clearly described? | ☐ | ☐ | ☐ | ☐ |
| 4. Were diagnostic tests or assessment methods and the results clearly described? | ☐ | ☐ | ☐ | ☐ |
| 5. Was the intervention(s) or treatment procedure(s) clearly described? | ☐ | ☐ | ☐ | ☐ |
| 6. Was the post-intervention clinical condition clearly described? | ☐ | ☐ | ☐ | ☐ |
| 7. Were adverse events (harms) or unanticipated events identified and described? | ☐ | ☐ | ☐ | ☐ |
| 8. Does the case report provide takeaway lessons? | ☐ | ☐ | ☐ | ☐ |

Overall appraisal: Include ☐ Exclude ☐ Seek further info ☐

| Comments (including reason for exclusion): |
|---|
| |
