# Supplementary material for: End-of-life care for people with severe mental illness: mixed methods systematic review and thematic synthesis of published case studies (the MENLOC study)
Source: BMJ Open. 2022 Feb 22;12(2):e053223. doi: 10.1136/bmjopen-2021-053223 (PMC8867317; doi:10.1136/bmjopen-2021-053223)
Supplement: Supplementary data [file bmjopen-2021-053223supp006.pdf]

Table 1 Characteristics of case studies included in the review

**Author, Year**<br><br>**Country**<br><br>**Purpose of article**	**Setting**<br><br>**Demographic characteristics**	**Focus of case study**	**Discussion**
1 Ang et al 2009[148]<br><br>Singapore<br><br>Addresses the challenge of what should be done in the case of people with chronic conditions which impair their capacity	Emergency hospital care of a woman in her 40s with a diagnosis of schizoaffective disorder since her early 20s and with recent end stage renal failure	Ethical issues in EoLC for people lacking capacity to make decisions	Autonomy of a patient with regard to medical treatment, assessment of the patient's decisional capacity, and the process of deciding on the appropriate course to take in a patient without the mental capacity to give consent for required treatment in a potentially life-threatening situation<br><br>Legal frameworks, tools to assess capacity and the use of advanced ('Ulysses') contracts are all addressed
2 Badger and Ekham 2011[116]<br><br>USA<br><br>Ethical conflicts at the end of life for patients with personality disorder, substance misuse and self-harming behaviour	A case of a man in his 40s with long-term MH problems whose physical health declines (liver and respiratory failure) and whose care presents an ethical challenge to practitioners	Ethical issues in EoLC for people lacking capacity to make decisions	Decision-making for people lacking capacity is difficult in the absence of ADs and in the case of people with complex MH and substance misuse problems
3 Bakker 2000[109]	A residential service using DSA for older people with complex mental and	The use of the DSA approach, including in the example of people	The DSA approach is offered as a psychosocial counterbalance to more

Netherlands<br><br>Focuses on the PC of patients in a 'reactivation program', this being a service for older people with complex health problems	physical health problems, in which the case is a woman in her 90s	who end their lives in the DSA service, illustrating the phases of the approach in action	exclusively biomedically oriented approaches to care
4 Boyd et al 1997[117]<br><br>USA<br><br>The process of care for a hospice patient described as manipulating family, friends, and hospice staff during her EoLC	A hospice service for people at the EoL, with the case being a woman in her 50s with breast cancer who is described as having a pre-existing (but not clearly described) severe MH problem characterised by 'manipulative' behaviour	The challenges faced by hospice staff in providing care to someone perceived as being manipulative, and how these can be addressed using practices informed by psychodynamic thinking	Hospice staff can provide care for people with 'troubled personality' using psychodynamic approaches, including supporting ego functioning; supporting autonomy; setting limits; and assessing and managing staff emotional responses
5 Cabaret et al 2002[146]<br><br>France<br><br>The provision of EoLC in an inpatient psychiatric hospital setting	An inpatient psychiatric hospital, with the case being a man in his 40s with a diagnosis of schizophrenia and lung cancer	The provision of humane, palliative, care in a MH hospital	Inpatient MH staff are able to provide humane, dignified, care until the EoL for people with SMI
6 Candilis and Foti 1999[118]<br>USA	A case study beginning in an ED and moving to a MH unit, focused on the care of a woman in her 70s with cancer and psychosis	Decision-making in the context of EoLC and psychosis, where the patient's understanding and preferences are not clearly known	Differences in view can exist over the best course of action in cases where patients refuse treatment but do not elaborate supportive, palliative, care or more aggressive intervention

EoLC to someone with psychosis and cancer, where the patient's preferences are not clearly known			
7 Clements-Cortes 2004[143]<br><br>Canada<br><br>The use of music therapy in the emotional care of people who are at the EoL	A discussion on the value of music therapy, followed by three case studies all of people using an older people's PC inpatient service, of which two (women in their 70s) have pre-existing MH problems	The value of music in providing emotional support to people with terminal illnesses	Music therapy can facilitate emotional expression, including in people with SMI
8 Doron and Mendlovic 2008[147]<br><br>Israel<br><br>EoLC in inpatient MH wards	Two case studies of people, one in her 40s at her death and the other a woman whose age at death is not stated, cared for near to the EoL in inpatient MH settings, where both had a diagnosis of SMI and cancer	The challenges for both patients and staff of being cared for during a terminal illness in a MH inpatient setting	Information on the patient's condition and prognosis should be given in the context of their capacity to understand, decisions should be made by patients where possible or otherwise by clinicians and home is where people should die meaning that if this is a psychiatric ward staff should be supported to provide care for as long as possible
9 Feely et al 2013[119]<br><br>USA<br><br>EoLC in a hospice for people with borderline personality disorder	A case study of a woman in her 40s with a diagnosis of borderline personality disorder and terminal metastatic pancreatic cancer, being cared for in a hospice	The difficulties for staff in hospices of providing care for people with complex MH problems characterised by impulsive behaviour and difficulties in relationships	There is a lack of evidence to support interventions with people with borderline personality disorder at the EoL, but behavioural interventions should be tailored to the person based on the creation of genuine, respectful, relationships

10 Feldman and Petriyakoil 2006[120]<br><br>USA<br><br>PTSD at EoL	A case study of a male veteran in his 60s with heart failure and acute onset of PTSD symptoms.	Challenges of recurrence of latent PTSD symptoms seemingly triggered by the threat to life of terminal illness mimicking previous experiences.	Specific challenges in EoLC for people with PTSD may mean they experience psychological symptoms that have been dormant, become anxious and angry, avoid healthcare, distrust medical recommendations leading to nonadherence and refusal of treatment
11 Feldman et al 2014[110]<br><br>USA<br><br>The provision of PC to people with PTSD at the EoL	A re-presentation of a new model of care to people with a diagnosis of PTSD, either pre-existing or post-EoL diagnosis, illustrated by three case studies (men in their 60s) in diverse PC settings	An illustrated model of care for people with PTSD at the EoL, involving therapy over three distinct stages: Palliate Immediate Discomfort and Provide Social Supports; Provide Psychoeducation and Enhance Coping Skills; and Treat Specific Trauma Issues	The SPPC model can be used to support terminally ill patients with PTSD where conventional PTSD interventions are challenged by limited life expectancy, fatigue and other concerns
12 Feldman et al 2017[111]<br><br>USA<br><br>The provision of PC to people with PTSD at the EoL	A case of a male veteran in his 70s with a diagnosis of PTSD and multiple myeloma, admitted for inpatient hospice care at the EoL	How a staged approach to PTSD care is helpful for people at the EoL	A further illustration of how the SPPC model can be used to support terminally ill patients with PTSD where conventional PTSD interventions are challenged by limited life expectancy, fatigue and other concerns
13 Geppert et al 2011[121]<br><br>USA	A detailed case study of a man in his 50s with a pre-existing a diagnosis of schizoaffective disorder who has	The ethical issues around treatment when people have psychosis and lack decisional capacity	Psychosis raises ethical issues for EoLC as it undermines decisional capacity, raises questions about the use of proxies and additional questions about where care ought to

PC and capacity in the context of people with psychosis and terminal cancer	terminal laryngeal cancer and who is admitted to hospital		be provided (MH services, or ned of life services)
14 Gonzalez et al 2009[122]<br><br>USA<br><br>Hospice care of a man with breast cancer and bipolar disorder	A medical case study of a man in his 80s with complex health needs who receives inpatient care, and who has a diagnosis of bipolar disorder, dementia and breast cancer first treated 20 years previously	The complexity of providing care for people with multiple needs, in the context of family members also having significant health needs	Curative care is difficult to provide if treatment is not consistently provided and accepted, and PC is appropriate particularly when caregivers have health needs of their own
15 Griffith 2007[123]<br><br>USA<br><br>Palliative and EoLC for a man with heart and lung failure and schizophrenia [pt 1 of 2]	An extended case study of a man in his 60s, focusing on his schizophrenia diagnosis and his lung and heart disease and his care at home until death	The role of the psychiatrist in the provision of integrated care, including at the EoL, to people with SMI and multi-organ failure	Psychiatrists have an important part to play in treating people who have both severe MH and terminal physical health problems
16 Griffith 2007[124]<br><br>USA<br><br>Palliative and EoLC for a man with heart and lung failure and schizophrenia [pt 2 of 2]	An extended case study of a man in his 60s, focusing on his schizophrenia diagnosis and his lung and heart disease and his care at home until death	The role of the psychiatrist in the provision of integrated care, including at the EoL, to people with SMI and multi-organ failure	Psychiatrists should have cultural and religious competence and be able to work with families
17 Hill 2005[125]<br><br>USA	A case of a woman in her 80s described as having borderline personality disorder and hospice care at home	EoLC for people with a diagnosis of borderline personality disorder and complex physical health problems and family histories	Nurses should develop therapeutic relationships, have good supervision, be consistent in caregiving, and have

Hospice at home care for a person with borderline personality disorder			good working relationships with families and psychiatrists
18 Irwin et al 2014[26]<br><br>USA<br><br>Cancer care, including to EoL, for people with schizophrenia	A detailed review of literature followed by a case study of a woman in her 60s with a diagnosis of paranoid schizophrenia and terminal cancer, whose care moves from home to inpatient hospice	Health inequalities for people with a diagnosis of schizophrenia and cancer	Practitioners need improved understanding of health disparities experienced by people with a diagnosis of schizophrenia and cancer, research is needed to examine factors influencing survival and quality of life and better integration is required between oncology and psychiatric services
19 Kadri et al 2014[144]<br><br>Canada<br><br>Care for a person with schizophrenia and advanced kidney disease who lacks capacity and resists treatment	A case of a woman in her 50s with a diagnosis of schizophrenia and advanced chronic kidney disease admitted to hospital as an emergency and who refuses dialysis, then receives supportive care	Ethical issues in the case of people with SMI and who are unable to make decisions, but who actively resist treatment	Two ethical issues are highlighted: the lack of acceptance of treatment, and the risk of doing immediate harm if treatment is continued nonetheless
20. Kennedy et al 2013[114]<br><br>UK<br><br>Homelessness and EoLC	A case study of a woman in her 50s with diagnosis of schizophrenia and cervical cancer living semi-independently in a one bedroomed flat.	Treatment refusal in someone with severe mental illness who has the capacity to make decisions and her determination to return home to live.	Providing care for a person in their own home involving multiple professional teams in support of her autonomous decision to remain out of hospital and considering her refusal of active treatment
21 Kunkel et al 1997[126]<br><br>USA	A discussion of denial of cancer followed by five illustrative cases:<br><br>a woman in her 30s with a diagnosis of psychosis and breast cancer, who	A review of factors associated with non-compliance in people with cancer, specifically psychoses and cognitive impairment Both are shown to play a role in delayed help-seeking and non-	Key issues affecting cancer treatment are competence, the place for legal intervention, ambivalence towards health care providers and the

Denial of cancer and non-compliance in treatment	misses treatment and dies following an emergency admission;<br><br>a woman in her 50s with presumed 'organic personality syndrome' and squamous cell carcinoma who was psychotic and who died in hospital;<br><br>-a man in his 70s with presumptive pancreatic cancer and pre-existing organic delusional disorder and a diagnosis of major depression with psychosis, who had home hospice care;<br><br>a woman in her 70s with a diagnosis of dementia and breast cancer;<br><br>a woman in her 70s with a diagnosis of schizophrenia, superimposed dementia and lung cancer	compliance with cancer, and EoL, care	importance of early psychiatric intervention
22 Levin and Feldman 1983[127]<br><br>USA (the authors are based in South Africa)<br><br>Ethical issues surrounding the care of people with psychosis and terminal cancer	A case of a woman in her 30s with a diagnosis of schizophrenia and terminal breast cancer who is described as wanting to leave hospital without medical care following the treatment of an acute episode of her mental illness	A discussion of ethical issues associated with the care of people with SMI and terminal illnesses when treatment is refused	People have a right to refuse high-powered medicine at the EoL
23 Lopez et al 2010[128]	The case of a woman in her 30s with a diagnosis of chronic anorexia	Discussion of ethical and legal issues associated with caring for a person with a psychiatric condition who is not	Refusal of treatment can be irrational but must be weighed against workers obligations and duty of care and the

USA<br><br>Explores how medical futility and principles of palliation may contribute to the management of treatment refractory anorexia nervosa	nervosa, treated unsuccessfully for several years	helped by treatment where the condition ultimately leads to her death	autonomy of the individual. Two broad options are considered, 1) involuntary treatment, or 2) attempting to motivate the person until such time as they are willing to engage in treatment. The first option may have kept her alive in the short term but seemed unlikely to reverse the underlying condition
24 Maloney 2014[115]<br><br>USA [blog]<br><br>https://www.thejournal.ie/readme/bipolar-ii-disorder-denial-mental-health-1467162-May2014<br><br>A wife's experiences of her husband's bipolar disorder and terminal cancer	A personal blogpost recording a woman's experiences of her husband's diagnosis with a diagnosis of bipolar disorder, his subsequent terminal cancer and his death at home	A first-hand, narrative, account of long-term experiences ending in the death of a loved one	SMI changes people's lives, and can cause people to lose touch with reality People affected (patients and families) need support
25 Mason and Bowman 2018[112]<br><br>Country unknown (conference abstract)<br><br>The challenges of providing cancer care at the EoL to people with SMI and fluctuating capacity	A conference proceedings abstract describing the care of a man in his 70s with a diagnosis of schizophrenia and fluctuating capacity admitted to hospital for investigations and care into prostate cancer and metastases	An account of the challenges for people with SMI and fluctuating capacity in the context of EoL cancer care	Determining capacity in the context of SMI and acute physical illness is difficult, where standardisation in capacity assessment may help
26 McCasland 2007[129]<br><br>USA	A case of a woman in her 50s with a diagnosis of schizoaffective disorder, alcohol problems, liver damage and	Hospice nurses need better preparation to look after people at the EoL who also have SMI	Communication skills are important for hospice nurses caring for people with SMI at the EoL, and hospice nurses

The care and treatment of people with SMI and cancer, including at the EoL	breast cancer who is cared for in the community until dying in a hospice		needs more training to care for this group of people
27 McKenna et al 1994[130]<br><br>USA<br><br>The treatment with clozapine of people with SMI and cancer, where treatment also includes antineoplastic medication	A letter to the editor describing a man in his 40s with a diagnosis of schizophrenia and lung cancer with a poor prognosis, treated with clozapine, whose care was transferred from a MH to a medical care institution	The combination of clozapine with anti-tumour drugs	Guidance is needed on the combination of clozapine (an anti-psychotic) with anti-cancer drugs
28 Mogg and Bartlett 2005[140]<br><br>UK<br><br>The care and treatment of a man with treatment-resistant psychosis and life-threatening renal failure	The case is a man in his forties with a 30-year history of psychosis and multiple admissions to MH hospital, now discovered to have life-threatening renal disease requiring regular dialysis	Different approaches to the assessment of capacity (outcome, status, functional), set in the context of the legal framework	Capacity can fluctuate, and 'best interests' need to be considered which can mean guidance needing to be sought from the courts
29 Moini and Levinson 2009[131]<br><br>USA<br><br>The care and treatment of people with simple schizophrenia and medical conditions	A case of a woman in her 40s with a diagnosis of simple schizophrenia who is lost to services following diagnosis of breast cancer with metastases, and who is located at a later point at the terminal stage of her illness	Approaches to working with people with a diagnosis of simple schizophrenia and medical problems	Proactive care is needed so that people stay in touch with services, though people with a diagnosis of simple schizophrenia are also likely to have capacity to decide on their courses of treatment
30 Monga et al 2015[132]<br><br>USA	The case is of a man in his 70s with a diagnosis of schizophrenia, treated with clozapine, who continues his antipsychotic medication whilst having	The use of clozapine as an atypical antipsychotic alongside the use of chemotherapy	There is limited information to guide management of clozapine treatment during chemotherapy, and close

The use of clozapine in people with psychosis who are also having chemotherapy	hospice care and treatment for oesophageal cancer		cooperation between psychiatrists and oncologists is needed
31 Muhtaseb et al 2001[141]<br><br>UK<br><br>The treatment of people with SMI and advanced, terminal, cancer	The case is of a man in his 70s with a diagnosis of schizophrenia and advanced basal cell carcinoma, who refuses active intervention	Treatment of advanced, life-threatening, cancer in people with SMI who refuse active intervention	Consent must be freely given, and people (including people with SMI) should be considered able to decide for themselves
32 O'Neill et al 1994[142]<br><br>UK<br><br>Hospice care for woman with anorexia nervosa	The case of a woman in her 20s with a diagnosis of anorexia admitted to a hospice in poor physical condition after 7 years of anorexia nervosa that was not helped by treatment.	Discusses the treatment and rapid decline to eventual death of a young woman from the complications associated with anorexia nervosa.	Recognises that psychiatric units do not have relevant expertise for providing complex physical healthcare. Says that her condition was identified as incurable by the psychiatric team and when the patient and her family accepted this she was then able to access appropriate care. Says hospice staff are familiar with other EoLC and that this largely worked for this woman helped by an excellent referral letter (no clue how this is judged) and ongoing support from the psychiatrist. They conclude that there is a role for carefully selected non-cancer patients to be cared for in SPC centres
33 Picot et al 2015[15]<br><br>Australia	The case is of a woman in her 40s who has a diagnosis of bipolar disorder and metastatic breast cancer,	An illustrative case showcasing the work of nurse practitioners involved in the IMhPaCT programme, which is	People with SMI at the EoL face additional problems of isolation, declining physical abilities, pain, and disintegrating selfhood Collaboration

The IMhPaCT, designed to improve care for people with SMI and life-limiting illnesses	who receives integrated MH and PC at home before dying in hospital	designed to bring together MH and PC for people with SMI	across specialities can enhance outcomes, and MH nurses have a role to play
34 Rice et al 2012[133]<br><br>USA<br><br>Patient-provider communication in EoLC	The case of a male veteran in his 50s with renal disease and a diagnosis of schizophrenia who refuses dialysis treatment.	The case provides an example of man refusing treatment in line with previous history and is found to lack capacity to decide in his best interests. Involvement of his sister who is ill-prepared to take on proxy decisions	Discussion hinges on challenges of enforcing physical treatments on a person who is refusing, handling autonomy versus coercion, recognition of need for building therapeutic alliances with the person and their surrogates
35 Rodriguez-Mayoral 2018[113]<br><br>Mexico<br><br>Integrating MH and PC for people with SMI and cancer	A short conference abstract describing a woman in her 70s with a diagnosis of bipolar disorder and advanced, untreated, colon cancer from which she dies	The case briefly describes how the woman was cared for until her death, free form symptoms of her mental illness	Cancer care regardless of disease stage should be integrated with palliative and MH care to control symptoms and quality of life and death
36 Romm et al 2009[134]<br><br>USA<br><br>Managing cancer care for people using inpatient MH services	The case is a man in his 40s with a diagnosis of schizophrenia and osteosarcoma for which amputation was indicated, and who refused surgical treatment but who was judged to lack capacity His mother assumed decision-making responsibility and concurred with her son, and PC was initiated	Treatment of people with acute MH problems and life-threatening cancer, and patients' capacity to understand and made decisions	Psychiatrists have responsibilities to represent the interests of people who are 'mentally compromised', including those who are also irreversibly medically compromised Collaboration with PC practitioners is important, and whilst SMI can impair capacity this is not necessarily so
37 Shah et al 2008[135]<br><br>USA	The case is a homeless man in his 60s with a diagnosis of schizophrenia and neglected basal cell carcinoma, and who refuses treatment in the	Informed consent is key to the provision of cancer treatment, and in	In the absence of being able to give informed consent interprofessional

The treatment of people with SMI and advanced, terminal, cancer	context of a lack of capacity and is then treated for his mental illness	cases where people lack capacity additional care needs to take	teams need to be involved, along with independent ethics committees
38 Stecker 1993[136]<br><br>USA<br><br>Caring for young people with life-threatening cancer on an acute psychiatric ward	The case is of a man in his 20s with presumed bipolar disorder and sarcoma who is admitted to an inpatient psychiatric unit and who causes staff to struggle with the acceptance of death	Staff in MH wards do not routinely care for young people at the EoL, and this case challenged staff	MH nurses need to be able to identify feelings preventing them from working therapeutically with patients
39 Steves and Williams 2016[137]<br><br>USA<br><br>EoLC for people with terminal illnesses and SMI	A very brief case of a man, of unknown age, who has a diagnosis of schizophrenia and terminal lung cancer but who has capacity and refuses treatment prior to his death	Services for people with SMI at the EoL are in short supply, MH nurses need training in EoLC, attention needs to be paid to the environment, communication needs to be effective, caregivers needs to be supported, family members may be resistant and other inpatients need to be supported following a death	Investments are needed in services and staff to care for increasing numbers of people with SMI who also have terminal medical conditions
40 Terpstra et al 2014[138]<br><br>USA<br><br>EoL hospice care for people with SMI	The case is of a man in his 60s with a diagnosis of schizophrenia living in adult foster care who reports physical symptoms and is found to have bladder cancer and brain masses, moves to an open hospice and whose care and treatment is then described	The complexity of providing hospice care for people with SMI and terminal cancer, and the role of nurses	People with schizophrenia diagnoses are often medically undertreated, have shortened life expectancies, and have care which is challenged by their placements, poor communication and symptom management

41 Thomson and Henry 2012[70]<br><br>USA<br><br>The challenge for oncology nurses of caring for people with SMI and cancer	A paper on oncology nursing for people with pre-existing SMI, including a series of brief case studies: a woman in her 30s with a diagnosis of major depression and then breast cancer; a man in his 40s with a diagnosis of bipolar disorder and advanced pancreatic cancer and a woman in her 20s with a diagnosis of schizophrenia and breast cancer	Care and treatment of people with pre-existing SMI at the EoL, with a particular emphasis on understanding mental illnesses and its treatment	Medical professionals need to be aware of premature mortality in people with SMI, and need to develop skills and knowledge in caring for people with SMI as an underserved group Practice is advancing in the areas of drug treatments, case management, family work and social support
42 Webber 2012[145]<br><br>Canada<br><br>Caring for a man with SMI in his supported home prior to hospice care	A brief case study of a man of unknown age who has a diagnosis of schizophrenia and metastatic gastric cancer and who was cared for in his supported home for as long as possible until being transferred to a hospice, where he died	Pain control, control over of levels of intervention, control over maintaining meaningful, relationships, outcomes of lost autonomy, lack of resources, restoring justice for patients with SMI and terminal illness, and innovation are all discussed	Autonomy has been diminished for people with SMI and palliative illness, yet people with SMI at the life want the same things as everyone else Services and care need to improve,

Key: DSA: dynamic system analysis; EoL: end of life; EoLC: end of life care: IMhPaCT: Integrated Mental Health and Palliative Care Task; PTSD: post-traumatic stress disorder; SMI: severe mental illness; SPC: specialist palliative care: SPPC: stepwise psychosocial palliative care

Table 2 Critical appraisal scores

Citation	Q1	Q2	Q3	Q4	Q5	Q6	Q7	Q8	Score
1. Ang et al 2009[148]	Y	Y	Y	N	Y	Y	Y	Y	7/8
2. Badger and Ekham 2011[116]	Y	Y	Y	N	Y	Y	Y	Y	7/8
3. Bakker 2000[109]	Y	Y	Y	N	Y	Y	Y	Y	7/8
4. Boyd et al 1997[117]	Y	Y	Y	N	Y	Y	Y	Y	7/8
5. Cabaret et al 2002[146]	Y	Y	Y	N	Y	Y	Y	Y	7/8
6. Candilis and Foti 1999[118]	Y	Y	Y	N	Y	Y	Y	Y	7/8
7. Clements-Cortes 2004[143]	Y	Y	Y	N	Y	Y	N	Y	6/8
8. Doron and Mendlovic 2008[147]	Y	Y	Y	Y	Y	Y	Y	Y	8/8
9. Feely et al 2013[119]	Y	Y	Y	N	Y	Y	Y	Y	7/8
10. Feldman and Petriyakoil 2006[120]	Y	UC	Y	N	N	Y	Y	N	4/8
11. Feldman et al 2014[110]	Y	Y	Y	N	Y	Y	Y	Y	7/8
12. Feldman et al 2017[111]	Y	Y	Y	N	Y	Y	Y	Y	7/8
13. Geppert et al 2011[121]	Y	Y	Y	Y	Y	Y	Y	Y	8/8
14. Gonzelez et al 2009[122]	Y	Y	Y	Y	Y	Y	Y	Y	8/8
15. Griffith 2007[123]	Y	Y	N	Y	Y	Y	Y	Y	7/8
16. Griffith 2007[124]	Y	Y	N	Y	Y	Y	Y	Y	7/8
17. Hill 2005[125]	Y	Y	Y	Y	Y	Y	Y	Y	8/8
18. Irwin et al 2014[26]	Y	Y	Y	Y	Y	Y	Y	Y	8/8
19. Kadri et al 2018[144]	Y	N	Y	N	Y	N	Y	Y	5/8
20. Kennedy et al 2013[114]	Y	UC	Y	N	Y	Y	Y	Y	6/8
21. Kunkel et al 1997[126]	Y	Y	Y	Y	Y	Y	Y	Y	8/8
22. Levin and Feldman 1983[127]	Y	Y	Y	N	Y	N	Y	Y	6/8
23. Lopez et al 2010[128]	Y	Y	Y	N	Y	Y	N	Y	6/8
24. Maloney et al 2014[115]	Y	Y	Y	N	Y	Y	Y	Y	7/8
25. Mason and Bowman 2018[112]	Y	Y	Y	Y	Y	Y	Y	Y	8/8

26. McCasland 2007[129]	Y	Y	Y	Y	Y	Y	Y	Y	8/8
27. McKenna et al 1994[130]	Y	N	UC	N	Y	N	Y	Y	4/8
28. Mogg and Bartlett 2005[140]	Y	N	N	N	Y	N	N	Y	2/8
29. Moini and Levenson 2009[131]	Y	Y	Y	N	N	N	Y	Y	5/8
30. Monga et al 2015[132]	Y	Y	Y	Y	Y	Y	Y	Y	8/8
31. Muhtaseb et al 2001[141]	Y	Y	Y	Y	Y	Y	Y	Y	8/8
32. O'Neill et al 1994[142]	Y	N	Y	N	Y	N	N	N	2/8
33. Picot et al 2015[15]	Y	Y	Y	Y	Y	Y	Y	Y	8/8
34. Rice et al 2012[133]	Y	N	Y	N	Y	Y	Y	Y	6/8
35. Rodriguez-Mayoral 2018[113]	Y	N	N	N	N	Y	Y	Y	5/8
36. Romm et al 2009[134]	Y	N	Y	N	N	N	Y	Y	3/8
37. Shah et al 2008[135]	Y	N	Y	Y	Y	Y	Y	Y	7/8
38. Stecker 1993[136]	Y	N	Y	N	N	N	Y	Y	4/8
39. Steves and Willliams 2016[137]	Y	N	N	N	Y	Y	Y	Y	4/8
40. Terpstra et al 2014[138]	Y	Y	Y	Y	Y	Y	Y	Y	8/8
41.Thomson and Henry 2012[139]	Y	Y	N	N	N	N	N	Y	2/8
42. Webber 2012[145]	Y	N	N	N	N	Y	Y	Y	3/8

Key: Y=Yes, N=No, UC=unclear
