# Supplementary material for: End-of-life care for people with severe mental illness: mixed methods systematic review and thematic synthesis of published case studies (the MENLOC study)
Source: BMJ Open. 2022 Feb 22;12(2):e053223. doi: 10.1136/bmjopen-2021-053223 (PMC8867317; doi:10.1136/bmjopen-2021-053223)
Supplement: Author's manuscript [file bmjopen-2021-053223.draft_revisions.pdf]

BMJ Open is committed to open peer review. As part of this commitment we make the peer review history of every article we publish publicly available.

When an article is published we post the peer reviewers' comments and the authors' responses online. We also post the versions of the paper that were used during peer review. These are the versions that the peer review comments apply to.

The versions of the paper that follow are the versions that were submitted during the peer review process. They are not the versions of record or the final published versions. They should not be cited or distributed as the published version of this manuscript.

BMJ Open is an open access journal and the full, final, typeset and author-corrected version of record of the manuscript is available on our site with no access controls, subscription charges or pay-per-view fees (http://bmjopen.bmj.com).

If you have any questions on BMJ Open's open peer review process please email info.bmjopen@bmj.com

BMJ Open

End of life care for people with severe mental illness: mixed methods systematic review and thematic synthesis of published case studies (the MENLOC study)

Journal:	*BMJ Open*
Manuscript ID	bmjopen-2021-053223
Article Type:	Original research
Date Submitted by the Author:	18-May-2021
Complete List of Authors:	Coffey, Michael; Swansea University, Public Health, Policy and Social Sciences<br>Edwards, Deborah; Cardiff University, School of Healthcare Sciences<br>Anstey, Sally; Cardiff University, School of Healthcare Sciences<br>Gill, Paul; Cardiff University<br>Mann, Mala; Cardiff University, University Library Services<br>Meudell, Alan<br>Hannigan, Ben; Cardiff Univ, School of Healthcare Sciences
Keywords:	PALLIATIVE CARE, MENTAL HEALTH, ONCOLOGY, Organisation of health services < HEALTH SERVICES ADMINISTRATION & MANAGEMENT

SCHOLARONE™
Manuscripts

I, the Submitting Author has the right to grant and does grant on behalf of all authors of the Work (as defined in the below author licence), an exclusive licence and/or a non-exclusive licence for contributions from authors who are: i) UK Crown employees; ii) where BMJ has agreed a CC-BY licence shall apply, and/or iii) in accordance with the terms applicable for US Federal Government officers or employees acting as part of their official duties; on a worldwide, perpetual, irrevocable, royalty-free basis to BMJ Publishing Group Ltd ("BMJ") its licensees and where the relevant Journal is co-owned by BMJ to the co-owners of the Journal, to publish the Work in this journal and any other BMJ products and to exploit all rights, as set out in our licence.

The Submitting Author accepts and understands that any supply made under these terms is made by BMJ to the Submitting Author unless you are acting as an employee on behalf of your employer or a postgraduate student of an affiliated institution which is paying any applicable article publishing charge ("APC") for Open Access articles. Where the Submitting Author wishes to make the Work available on an Open Access basis (and intends to pay the relevant APC), the terms of reuse of such Open Access shall be governed by a Creative Commons licence – details of these licences and which Creative Commons licence will apply to this Work are set out in our licence referred to above.

Other than as permitted in any relevant BMJ Author's Self Archiving Policies, I confirm this Work has not been accepted for publication elsewhere, is not being considered for publication elsewhere and does not duplicate material already published. I confirm all authors consent to publication of this Work and authorise the granting of this licence.

For peer review only

End of life care for people with severe mental illness: mixed methods systematic review and thematic synthesis of published case studies (the MENLOC study)

Michael Coffey[a] ORCID 0000-0002-0380-4704

Deborah Edwards[b] https://orcid.org/0000-0003-1885-9297

Sally Anstey[b] https://orcid.org/0000-0003-2295-3761

Paul Gill[b] https://orcid.org/0000-0003-4056-3230

Mala Mann[c] http://orcid.org/0000-0002-2554-9265

Alan Meudell[d] https://orcid.org/0000-0001-8138-4744

Ben Hannigan[b] https://orcid.org/0000-0002-2512-6721

Corresponding author: Michael Coffey, Department of Public Health, Policy and Social Sciences, College of Human and Health Sciences, Swansea University, UK, email: m.j.coffey@swansea.ac.uk

a Department of Public Health, Policy and Social Sciences, College of Human and Health Sciences, Swansea University, UK

b School of Healthcare Sciences, College of Biomedical and Life Sciences, Cardiff University, UK

c Specialist Unit for Review Evidence, University Library Services, Cardiff University, UK

d Independent Service User Researcher, UK

For peer review only - http://bmjopen.bmj.com/site/about/guidelines.xhtml

Abstract:

Objectives: People with severe mental illness have significant co-morbidities and reduced life expectancy. The objective for the review reported in this paper was to synthesise material from case studies relating to the organisation, provision and receipt of care for people with severe mental illness who have an end-of-life diagnosis.

Design: Systematic review and thematic synthesis.

Data sources: MEDLINE, PsycINFO, EMBASE, HMIC, AMED, CINAHL, CENTRAL, ASSIA, DARE and Web of Science from inception to December 2019. Supplementary searching for additional material including grey literature along with 62 organisational websites.

Results: Of the 11,904 citations retrieved, 34 research publications were included plus 28 non-research items. An additional 42 case study papers reporting 51 case studies were identified and are reported here. Twenty five of the 42 case study papers met seven, or more quality criteria with eight meeting half or less. Attributes of case study subjects included that just over half were men, had a mean age of 55 years, psychotic illnesses dominated, and the end-of-life condition was in most cases a cancer. Analysis generated themes as follows Diagnostic delay and overshadowing, Decision Capacity and Dilemmas, Medical Futility, Individuals and their networks, Care Provision.

Conclusions: In the absence of high-quality intervention studies, this evidence synthesis indicates that cross disciplinary care is supported within the context of established therapeutic relationships. Attention to potential delay and diagnostic overshadowing is required and care provision the values the preferences of individuals with severe mental illness experiencing an end-of-life condition is indicated.

Keywords: Palliative care**,** mental health, oncology, organisation of health services

PROSPERO registration number: CRD42018108988

For peer review only - http://bmjopen.bmj.com/site/about/guidelines.xhtml

Article Summary

Strengths and limitations of this study

- This systematic review represents, to our knowledge, the first synthesis of case studies in end-of-life care for people with prior severe mental illness.
- Case studies have a long history in medicine, provide useful insights, and fill an important gap in the absence of intervention research.
- We identified 51 case studies across 42 articles with 25 meeting seven or more of eight quality criteria.
- Most papers were from high income countries thus limiting transferability.
- Future research should focus on generating high quality intervention studies in this field.

For peer review only

For peer review only - http://bmjopen.bmj.com/site/about/guidelines.xhtml

Introduction

Mental ill-health is the leading cause of years lived with disability in 56 countries and the second leading cause in a further 56.[1] The wider economic costs of mental illness in England have been estimated at £105.2 billion each year[7] and £7.2 billion in Wales.[2]

The term 'severe' mental illness (SMI) has longstanding currency within the fields of mental health policy, services and practice.[3] It continues to be used in research,[4] and by the NIHR Dissemination Centre.[5] People with SMI have high co-morbidities,[6] and higher mortality rates and reduced life expectancy compared to the general population[7] across all age groups,[8] with a 10–20-year reduction in life expectancy.[9] Inequities, not limited to care at the end of life specifically, can be explained with reference to individual and system-level factors. People with SMI are less likely to attend health screenings and may respond to symptoms differently.[10] They may delay or avoid help, and are more likely to exhibit disruptive behaviours or miss contacts with health professionals[11,12] putting them at risk of delayed disease detection.[6] Inadequate support systems are also common among those with SMI, affecting their ability to access appropriate clinical care and navigate complex health systems.[13] Other factors influencing variations in mortality and morbidity for people living with SMI include poor previous experiences of seeking help from health care professionals, incorrect attribution of physical symptoms to psychiatric disorder by care staff, and lack of experience by mental health professionals in determining how and when to refer onwards to other appropriate services.[11,14]

End-of-life care refers to the care of people with diagnoses of advanced, incurable, cancer and/or end-stage lung, heart, renal or liver failure and who are likely to die within the next 12 months[15]. It includes care provided in hospitals, hospices, and other institutional settings (such as care homes, prisons, and hostels) and care provided at home and via outreach to people who may also be homeless.

Although cancer incidence amongst people living with SMI is similar to that of the general population, mortality rates are double.[16,17] This disparity may be related in part to late presentation and reduced use of interventions such as surgery, systemic anti-cancer therapies or radiotherapy.[18,19] The experience of SMI can delay access, detection and

For peer review only - http://bmjopen.bmj.com/site/about/guidelines.xhtml

treatment of life-threatening physical disorders and their symptoms, specifically pain.[20] Consequently, this patient cohort is more likely to present advanced cancers that are more complex and costly to treat, being less likely to undergo invasive treatments and more likely to die.[21]

Research at the interface of physical and mental health care is recognised as a priority.[22] Policies focus on improving end-of-life care, where diagnosis is immaterial.[23-31] These policies require the introduction of palliative and supportive care earlier in the illness trajectory. In national policy the needs of people with SMI who develop advanced incurable cancer and/or end-stage lung, heart, renal or liver failure are acknowledged poorly, or not at all. This group face the prospect of 'disadvantaged dying',[32 33] at a time when quality of care in the last months of life should be uniformly high for all groups.

In this paper we report one part of our NIHR funded systematic review focused on published evidence for end-of-life care for people with severe mental illness.[34] We provide a synthesis of published case studies. Case studies have a long tradition in healthcare[35] aiming to provide insights from the specific to illustrate broader lessons[36]. Given the limited research into end-of-life care for people experiencing severe mental illness, a synthesis of case studies can contribute new understandings and direct us towards patterns of care that might otherwise remain hidden.

Methods

The protocol[37] is registered in PROSPERO (CRD42018108988) (see supplementary file 1)and followed the Preferred Reporting Items for Systematic Review and Meta-Analysis (PRISMA) guidelines (see supplementary file 2). The methods are reported in detail in the full project report[34] and summarised here.

Ethics Statement

This is a systematic review, no patient data was collected for this review and research ethics approval was not required.

For peer review only - http://bmjopen.bmj.com/site/about/guidelines.xhtml

Inclusion criteria

Population

Adult participants (>18 years of age) with SMI who have an additional diagnosis of advanced, incurable, cancer and/or end-stage lung, heart, renal or liver failure and who are likely to die within the next 12 months was considered. SMI was defined as including those with but not limited to schizophrenia, schizophrenia spectrum and other psychotic disorders, schizotypal and delusional disorders, bipolar affective disorder, bipolar and related disorders, major depressive disorder and disorders of adult personality and behaviour.

Types of intervention and phenomena of interest

End-of-life care.

Context

End of life care provided in hospitals, hospices, and other institutional settings (such as care homes, prisons, and hostels), and care provided in the home and via outreach to people who may also be homeless.

Types of evidence

Case studies.

Exclusion criteria

Evidence relating to:

- mental health problems (e.g., depression) as a consequence of terminal illness (e.g., cancer or chronic organ failure).
- End-of-life care for people with mental and behavioural disorders due to psychoactive substance use, except where these coexisted with SMI as specified above.
- End-of-life care for people with dementia or other neurodegenerative diseases except where these coexisted with SMI as specified above.

For peer review only - http://bmjopen.bmj.com/site/about/guidelines.xhtml

The search strategy was developed for Ovid Medline and adapted for the other databases (see supplementary file 3). Searches were run on the following databases from inception for studies published in the English language.

- MEDLINE ALL; EMBASE; HMIC, PsycINFO; AMED;
- CINAHL; CENTRAL;
- ASSIA;
- DARE; Web of Science (WoS).

The keywords that were used to inform these searches included the following:

Palliative care OR Hospice care OR Terminal care OR Terminally ill OR End of life care OR Last year of life

AND

Neoplasms OR Cancer OR heart failure, lung failure, liver failure or lung failure

AND

Mental health OR Depression OR Mental disorders OR Depressive disorder OR Personality disorders OR Bipolar disorder OR Schizophrenia OR Mental illness.

Supplementary searches were conducted for additional papers, information on studies in progress, unpublished research, research reported in the grey literature and personal blogs (see supplementary file 4).

Searches were also conducted using Google as described by Mahood et al.[38 39] The first 10 pages of each Google output were screened using the terms:

- "palliative care" and "mental illness"
- "end of life" and "mental illness"
- "end of life" and schizophrenia (searching first 5 pages of output)
- "end of life" and bipolar (searching first 5 pages of output)

The contents of the last 2 years of the *Journal of Pain and Symptom Management*, *Cancer*, *Psycho-Oncology* and *BMJ Supportive & Palliative Care* were hand-searched. These journals were selected due to the large number of outputs identified in database searches being

For peer review only - http://bmjopen.bmj.com/site/about/guidelines.xhtml

published within them. Reference lists of included studies were scanned, and forward citation tracking performed using WoS.

Screening

References were collected and deduplicated using Covidence™ systematic review software (Veritas Health Innovation, Melbourne, Australia).

Data extraction

Demographic data were extracted into tables and followed the format recommended by the CRD.[39] These data were checked by a second reviewer independently for accuracy and completeness. Where multiple publications from the same study were identified, data were extracted and reported as a single study.

Data synthesis

All case studies were available in full text form, were read and re-read, uploaded to NVivo, inductively coded by one reviewer and checked by a second, and then synthesised into five themes[40].

Assessment of methodological quality

The JBI critical appraisal checklist for case reports was used.[41] This is an eight-item checklist ('yes', 'no', 'unclear', 'not applicable') from which an overall score is generated reflecting the number of items answered 'Yes' (see supplementary file 5).

Patient and public involvement

This study included public and patient involvement perspectives commencing with original concept, the definition of search terms and parameters, study steering group, and onwards to impact and dissemination (e.g., co-author on report and papers).

Results

Search results

For peer review only - http://bmjopen.bmj.com/site/about/guidelines.xhtml

Of the 11,904 citations retrieved, 34 research publications were included[34] plus 28 non-research items and 42 case study papers which are reported in this current paper. The PRISMA flow diagram is shown in figure 1.

Figure 1 PRISMA flow chart here

Description of case studies

There were 42 publications containing 51 case studies of individuals with an existing mental illness diagnosis who went on to develop an end-of-life condition (see Table 1, supplementary file 6). In four case studies the purpose of the paper was to show the application of a particular model of care e.g. Dynamic System Analysis (DSA),[42] or Stepwise Psychosocial Palliative Care (SPPC).[43 44] Case studies were mostly published in peer reviewed research journals (n=38) with two conference abstracts,[45 46] one appearing in a report[47] and one a first-person account blog.[48] The case studies ranged in depth from discursive papers with little direct detail about the individuals involved, to those that focused mainly on the patients' physical illness. Case studies reported on the health and/or social care provided to individuals, and as such provided insights into everyday practice and its associated opportunities, challenges, and dilemmas.

Country of research

Papers were published in the US (n=28);[19,43,44,48-71] UK (n=4);[47 72-74] Canada (n=3);[75-77] Australia (n=1);[8] France (n=1);[78] Israel (n=1);[79] Mexico (n=1);[46] Netherlands (n=1);[42] Singapore (n=1);[80] with one conference abstract not stating a country.[45]

TABLE 1 HERE Studies and Cases SUPPLEMENTARY FILE 6

Year of publication

The earliest case study was published in 1983[59] and outputs appeared regularly up until the end of the search period in 2019. Figure 2 displays the year of publication of the 42 case study articles.

For peer review only - http://bmjopen.bmj.com/site/about/guidelines.xhtml

Figure 2 HERE

Attributes of service users

The age range across the case studies was 20-91 years (mean 55 years) and was provided for all except two.[48 69] Women were the focus of 24 case studies. Diagnosis was reported for all case study individuals and included psychotic-type diagnoses (schizophrenia, psychosis, schizoaffective and bipolar conditions) (31 publications reporting 35 case studies),[8,19,42,45-48,51,54-56 81,58,59,61-73,75-80] personality disorder (5 publications reporting 5 case studies),[49,50,52,57 58] Post Traumatic Stress Disorder (PTSD) (3 publications reporting 3 case studies)[43,44,53] and anorexia nervosa (n=2).[60,74] The anorexia case studies are two outliers in that the end-of-life condition was a direct result of the mental health issue for those individuals leading to chronic fractures and organ failure.[60,74] The vast majority of end-of-life conditions presented in the case study papers were cancer related diagnoses, and organ failure (heart (n=3 (across four publications),[42,53,56,81] liver (n=1)[49] and renal (n=5)[57,65,72,76,80] made up the remainder.

Critical appraisal scores

The quality of the studies varied overall (see Table 2, supplementary file 7). Twenty five of the 42 papers met seven or more criteria. In most cases the single criterion missing related to the description of diagnostic tests or assessment methods and their results. Eight of the 42 papers met half or fewer of the eight quality criteria, but for the purposes of inclusivity all 42 papers were included in the subsequent narrative synthesis.

TABLE 2 HERE CRITICAL APPRAISAL SCORES SUPPLEMENTARY FILE 7

Thematic synthesis

These case studies report on key episodes, or critical junctures,[82] in the lives of people with SMI at the end-of-life. Five themes were identified centring on people with SMI presenting to health services and then disappearing again, or pivotal decisions with profound impact for patients and others. These themes are *Diagnostic delay and overshadowing*; *Decisional capacity and dilemmas*; *Medical futility; Individuals and their networks*; and *Care provision*.

For peer review only - http://bmjopen.bmj.com/site/about/guidelines.xhtml

Diagnostic delay and overshadowing

A consistent picture of delayed or late diagnosis and diagnostic overshadowing appears in case studies. Complex and enduring mental health problems may contribute to these issues. The problem of delay is signalled as arising from the mental illness of the individual who fails to recognise the seriousness of their plight, and presents to services only when their condition is advanced and treatment options limited.[51,58,59,63,70] For example, Ms A, African American woman in her thirties with an unspecified psychosis presented with a 20cm mass in her right breast adhering to the chest wall.[58] Investigations revealed a significant cancer with lymph node involvement with no metastases. Ms A was treated with chemotherapy and surgery, failed to attend for radiation treatment and considered new lesions as nothing more than 'haematoma'. She later presented in respiratory distress with large, presumably malignant effusions, asked not to be resuscitated and died soon after. The case study paper labels Ms A's difficulty with accepting her situation and similar behaviours as 'maladaptive denial'.

The tendency to impute the mental conditions of individuals as a source of late presentation, or indeed for deciding not to continue with treatment, is a recurring feature of case studies. Case studies indicate how diagnosis and treatment of serious life-threatening or life-limiting physical conditions are often complicated by untreated mental illness and the beliefs and behaviours arising from this.[58,59,63] Some case studies appear to elide behavioural aspects of presentations with mental ill-health and position these as problematic in the delivery of care and treatment with terms such as 'malingering', 'manipulative', 'dominance and aggression', 'demanding',[42,49,50,52] or problems for staff who 'struggled with how best to provide care'.[53]

Denial or a lack of insight, is a possible complicating factor. It is not unknown for persons diagnosed with other serious conditions to deny the gravity of their situation, and for people with pre-existing mental health conditions it appears that this is no different.[58] Specific issues arise in cases of PTSD where 'the threat to life inherent in terminal illness may mimic the original trauma' leading to exacerbation of psychological symptoms

For peer review only - http://bmjopen.bmj.com/site/about/guidelines.xhtml

associated with the condition including anxiety, anger, denial, avoidance and distrust of authority impeding medical adherence resulting in refusal of treatment.[53]

Individuals with long histories of pre-existing mental illness with regular access to healthcare professionals nevertheless experience delayed or late diagnosis of conditions that place them on the end-of-life trajectory.[19,46,54,64,77-79] For example, a man in his sixties with a longstanding diagnosis of schizophrenia, living in an adult foster home and under legal guardianship,[70] was seen fortnightly at a mental health clinic and attended a primary care provider to report a new onset cough. His examination was documented as benign but one week later he complained of hypotension and left-sided weakness. A detailed examination revealed advanced bladder cancer and multiple brain metastases not previously noted. The authors locate the problem in the mental condition suggesting people with conditions such as schizophrenia do not willingly verbalise pain or related symptoms. It is however difficult to escape the conclusion that extended contact with healthcare services had failed to identify his condition.

Decisional capacity and dilemmas

Decisional capacity of individuals to consent to treatment and/or to refuse treatments are reported and implicate professional dilemmas of determining the value of attempting curative treatments versus palliation.[45,47,49,54,58,59,72,73,80] It is argued[66] that clinicians have a particular duty to ensure that the interests of people with SMI are defended by offering medical treatment. While SMI can impair decisional capacity, this should not be assumed but instead thoroughly assessed.[66] In some examples the conclusion is that the person retained capacity to determine their treatment choices, and these choices were then respected.[47]

Treatment refusal is highlighted numerous times.[47,51,54,55,59,62,65,66,69,71-73] Past refusals prompt treatment teams to impute future problems.[65] For example, a patient in their eighties with a 20-year history of a mastectomy for breast cancer and refusal of medical care, re-presents with a bleeding and ulcerating mass on the chest wall.[55] The case study reports that the patient was not a case for curative treatment despite no evidence of metastases.

For peer review only - http://bmjopen.bmj.com/site/about/guidelines.xhtml

Treatment refusal is reported as arising from psychiatric symptomatology such as fixed beliefs about damnation or that thoughts could be read by physicians, rather than due to capacity to understand and make decisions based on available information.[19]

Fluctuating mental capacity requiring multiple assessments[45,72] is reported. This can mean resort to the courts in treatment refusal[72] for declaration of lawfulness, being in the best interests of the patient, or of not imposing treatment notwithstanding the patient's inability to accept or refuse treatment. In one case of treatment refusal in a man with decisional incapacity, the medical ethics committee concluded that even with full decision-making capacity a person might reasonably refuse radical procedures due to risks involved and the deforming nature of surgery.[67]

The absence of previously declared wishes on life-saving treatments is a recurring issue and suggests one area for future intervention testing.[76] In these circumstances teams seek agreement of a substitute decision maker, such as a family member[65,66] sometimes with power of attorney. The patient may however indicate by their actions their refusal e.g., repeatedly removing lifesaving treatments such as catheters in renal dialysis[76] or not agreeing to take medication.[58] Enforcing medical treatment when it is actively refused is not supported in this literature and may complicate future alliance building.[65]

Medical futility

The concept of medical futility is invoked in case studies of people with anorexia nervosa indicting how experience of this condition can exhaust the optimism of those doing the caring.[60] The language used, e.g. 'refractory' and 'incurable' reflects this.[74] Ms A, a woman in her early thirties with a diagnosis of anorexia nervosa where there is use of palliation and referral to hospice care for the physical consequences of the psychiatric condition.[60] In this case it is also reported that the option of an eating disorder treatment programme was eventually rejected on cost grounds, implying that perhaps the situation was less futile than indicated. Nevertheless, the treatment team and the ethics committee concluded, 'that her physical and psychiatric impairments were likely to lead to her death, despite any plausible attempts at aggressive intervention'.[60, p.373]

For peer review only - http://bmjopen.bmj.com/site/about/guidelines.xhtml

Individuals and their networks

Support networks for people with SMI are crucial, and the absence of these is implicated in delayed treatment-seeking. Case studies refer to family involvement,[42,56,81,61,71,80] whilst others indicate the absence of such support.[49,51] The end-of-life condition in some circumstances appears to have led to the re-emergence of family support,[78] or that teams actively support the person to reconnect with distant family.[47] For some, tensions in family involvement are reported[50,57] and in one example family dynamics are situated as the source of subsequent mental health-related troubles.[74] Case studies also identify family concerns for the individual with the end-of-life condition.[56,81] End of life care places additional demands on families, such as learning to manage symptoms[43] and lead to exhausted or burned-out family members.[60] One such demand arises from the absence of advance decisions, or where decisional capacity is in question. Families are then drawn into discussions on treatment and do not resuscitate decisions for which they are ill-prepared.[54,58,65,66,76] Families also express concern that person's mental health problems are overlooked by the palliative care team.[71]

Care Provision

Case studies reveal issues in the provision of care for treatment teams, such as how to handle psychiatric presentations.[42,50,65,71] Case studies also report examples of what has worked in supporting people with mental health problems at the end-of-life, including: the building of rapport and trust especially in people with PTSD;[43] the use of music therapy at end-of-life;[75] having conversations about death;[19,56,81,78] initiating hospice at home;[19,56,81] and multidisciplinary mental health care and palliative care being provided at home.[47] Challenges reported include mental health staff being emotionally unprepared for caring for people who are terminally ill.[68] Where people with SMI receive end-of-life care in hospices palliative care staff experience strong emotions, such as anger and frustration.[43] Case study papers occasionally offer psychodynamic interpretations of staff/patient interactions and care.[42,57,68]

Case studies reporting the ongoing delivery of care to individuals with pre-existing SMI and subsequent end-of-life advocate the benefits of a transdisciplinary approach involving

For peer review only - http://bmjopen.bmj.com/site/about/guidelines.xhtml

palliative care specialists, psychiatric specialists (preferably the team with a pre-existing relationship) and wider community members (e.g., religious ministers).[8,19,46,47,60,66,74]

Liaison consultation and collaboration is reported as beneficial for hospice staff. Examples include psychiatric consultation,[52] psychologists advising on reducing environmental stress[43] and a hospice nurse being supported by a psychiatric nurse.[57] Mutual benefits of hospice and mental health nurses working together in end-of-life care, and the similarities in their work are reported. Hospice staff[71] and mental health staff[78] express interest in learning from each other when providing end of life care for people with mental health problems.

Where models of care are reported these are small scale and do not indicate transferability or generalisability. Examples include treatment models e.g., DSA,[42] SPPC[43,44] and patient, provider, systems model.[19]

In almost every example the place of care is positioned as one arising from the preferences of the individual. These include palliative care in mental health institutions;[54,78,79] hospice care for mental health patients;[52,61,70,74] acute hospital;[8] and home care.[47] Repeated transfer between settings related to psychiatric and/or physical symptomatology is also evident[65] suggesting challenges to continuity of care.

Treatment challenges are reported in managing mental health-related medication alongside the provision of chemotherapy. Two case study papers report issues with the prescription of clozapine, as this is implicated in depressing white blood cell counts and could precipitate a life-threatening infection compounding the neutropenia associated with cytotoxic chemotherapy.[62,64] Treatment challenges also arise from people with SMI at the end-of-life absconding or being lost to follow-up.[63,73] A further challenge is in providing palliative care to patients who, despite careful explanation, continue to believe that they are not going to die.[60]

Discussion

Case studies demonstrate the complexities and the ethical dilemmas associated with the provision of care to people with SMI at the end-of-life. They reveal stark challenges

For peer review only - http://bmjopen.bmj.com/site/about/guidelines.xhtml

presented by delayed diagnosis, patients' fluctuating capacity to make decisions and a concern about the futility of treatment. Case studies also demonstrate, the lack of preparedness of professionals to meet patients' multiple needs. For example, palliative care staff lack mental health knowledge and mental health staff have little or no experience of palliative care. Despite this, professionals want to learn from each other across specialities to provide better care. Synthesised case study findings also show the dangers of ascribing delays in mobilising (or continuing with) palliative care services in response to patients' challenging presentations, or difficult behaviour.

Research on end-of-life care for people with mental health problems is limited[34] and in the absence of high quality intervention research case studies provide an important source of knowledge. While there is much to learn from these case studies, reporting of clinical details is variable, thus limiting generalisation. A further challenge in synthesis arises from system differences across multiple countries. Indeed, almost all case studies come from high income countries limiting transferability to other settings.

Practitioners sometimes struggle with engaging individuals in difficult discussions about their care. However, it is clear that people with severe mental illness want these conversations. Care provided across specialist boundaries and with the continuing support of already established therapeutic relationships is well supported. In providing care at the end-of-life for people with severe mental illness practitioners must work to sustain these relationships, display compassion and sensitivity in difficult but necessary conversations, engage across disciplinary barriers to improve their understanding, and establish preferences of the person in relation to end-of-life decisions.

The cases studies synthesised here provide a range of clinical and non-clinical experiences of care for people with end-of-life conditions in the context of severe mental illness. These experiences point to a range of possible approaches for improving the prompt diagnosis and care of these individuals. In the absence of intervention studies, it remains unclear how robust these indications are for future clinical care.

Conclusion

For peer review only - http://bmjopen.bmj.com/site/about/guidelines.xhtml

For peer review only

This synthesis of case studies indicates that cross disciplinary care provided in the context of established therapeutic relationships and which values the preferences of individuals with severe mental illness experiencing an end-of-life condition is supported in the absence of high-quality intervention research.

Figure 1 PRISMA flow chart

Figure 2: Histogram of year of publication of case studies

The original protocol for the study (supplementary file 1).

Funding statement: This work was supported by the National Institute for Health Research (NIHR) Health Services and Delivery Research programme, grant number HS&DR 17/100/15 and will be published in full in the Health Services and Delivery Research Journal; Vol. XX, No. XX.

A competing interests statement. None declared.

Author Contributions: Conceived and designed the systematic review: MC, DE, SA, PG, MM, AM, BH. Conducted the literature search: MM, DE. Analysed the data: MC, DE, SA, PG, AM, BH. Wrote the paper: MC, DE, SA, PG, MM, AM, BH. Data interpretation and critical revision of manuscript: MC, DE, SA, PG, MM, AM, BH. All authors reviewed and approved the manuscript.

Any checklist and flow diagram for the appropriate reporting statement, (supplementary file 2 for completed PRISMA checklist, supplementary file 5 for JBI checklist,)

Patient consent for publication: Not required.

Data availability statement All data of the current study is present in the main manuscript, figures, tables, and online supplemental material.

For peer review only - http://bmjopen.bmj.com/site/about/guidelines.xhtml

References

1. Vos T, Barber RM, Bell B, et al. Global, regional, and national incidence, prevalence, and years lived with disability for 301 acute and chronic diseases and injuries in 188 countries, 1990–2013: a systematic analysis for the Global Burden of Disease Study 2013. *The Lancet* 2015;386(9995):743-800. doi: https://doi.org/10.1016/S0140-6736(15)60692-4
2. Friedli L, Parsonage M. Promoting mental health and preventing mental illness: the economic case for investment in Wales. Cardiff: All Wales Mental Health Promotion Network, 2009.
3. Department of Health. Building bridges: A guide to arrangements for interagency working for the care and protection of severely mentally ill people. London: Department of Health, 1995.
4. Kronenberg C, Doran T, Goddard M, et al. Identifying primary care quality indicators for people with serious mental illness: a systematic review. *Br J Gen Pract* 2017;67(661):e519-e30. doi: 10.3399/bjgp17X691721 [published Online First: 2017/07/05]
5. National Institute for Health Research. Forward thinking: NIHR research on support for people with severe mental illness. Southampton: National Institute for Health Research, 2018.
6. Ellison N. Mental health and palliative care. London: Mental Health Foundation 2008.
7. Thornicroft G. Premature death among people with mental illness. *BMJ : British Medical Journal* 2013;346:f2969. doi: 10.1136/bmj.f2969
8. Picot SA, Glaetzer KM, Myhill KJ. Coordinating end of life care for individuals with a mental illness. A nurse practitioner collaboration. *Collegian: Journal of the Royal College of Nursing, Australia* 2015;22(1):143-9.
9. Chesney E, Goodwin GM, Fazel S. Risks of all-cause and suicide mortality in mental disorders: a meta-review. *World Psychiatry* 2014;13(2):153-60. doi: 10.1002/wps.20128 [published Online First: 2014/06/04]
10. Carney CP, Jones L, Woolson RF. Medical comorbidity in women and men with schizophrenia: a population-based controlled study. *J Gen Intern Med* 2006;21(11):1133-7. doi: 10.1111/j.1525-1497.2006.00563.x [published Online First: 2006/10/10]
11. Woods A, Willison K, Kington C, et al. Palliative care for people with severe persistent mental illness: A review of the literature. *Canadian Journal of Psychiatry* 2008;53(11):725-36.
12. Millman J, Galway K, Santin O, et al. Cancer and serious mental illness – patient, caregiver and professional perspectives: Study protocol. *Journal of Advanced Nursing* 2016;72(1):217-26.
13. Knapik GP, Graor CH. Engaging Persons with Severe Persistent Mental Illness into Primary Care. *The Journal for Nurse Practitioners* 2013;9(5):283-87. doi: https://doi.org/10.1016/j.nurpra.2013.02.019
14. Shalev D, Brewster K, Arbuckle MR, et al. A staggered edge: End-of-life care in patients with severe mental illness. *General Hospital Psychiatry* 2017;44:1-3.
15. General Medical Council. Treatment and care towards the end of life: Good practice in decision making. Manchester: General Medical Council, 2010.
16. Howard LM, Barley EA, Davies E, et al. Cancer diagnosis in people with severe mental illness: Practical and ethical issues. *The Lancet Oncology* 2010;11(8):797-804. doi: 10.1016/s1470-2045(10)70085-1
17. Batty GD, Whitley E, Gale CR, et al. Impact of mental health problems on case fatality in male cancer patients. *Br J Cancer* 2012;106(11):1842-5. doi: 10.1038/bjc.2012.150 [published Online First: 2012/04/21]
18. Desai MM, Bruce ML, Kasl SV. The effects of major depression and phobia on stage at diagnosis of breast cancer. *Int J Psychiatry Med* 1999;29(1):29-45. doi: 10.2190/0c63-u15v-5nur-tvxe [published Online First: 1999/06/22]
19. Irwin KE, Henderson DC, Knight HP, et al. Cancer care for individuals with schizophrenia. *Cancer* 2014;120(3):323-34.

For peer review only - http://bmjopen.bmj.com/site/about/guidelines.xhtml

For peer review only

20. Foti ME. Palliative care for patients with serious mental Illness. In: Chochinov HM, Breitbart W, eds. Handbook of Psychiatry in Palliative Medicine. 2nd ed. Oxford: Oxford University Press 2012.
21. Ishikawa H, Yasunaga H, Matsui H, et al. Differences in cancer stage, treatment and in-hospital mortality between patients with and without schizophrenia: Retrospective matched-pair cohort study. *British Journal of Psychiatry* 2016;208(3):239-44. doi: 10.1192/bjp.bp.114.156265
22. Department of Health. A framework for mental health research. London: Department of Health, 2017.
23. Department of Health. Our commitment to you for end of life care the government response to the review of choice in end of life care, 2016.
24. Department of Health SSaPS. Living matters dying matters. A palliative and end of life care strategy for adults in Northern Ireland 2010.
25. Scottish Government. Better cancer care: an action plan. Edinburgh: Scottish Government, 2008.
26. NHS Scotland. Living and dying well. A national action plan for palliative and end of life care in Scotland, 2008.
27. Scottish Government. Improving the health and wellbeing of people with long term conditions in Scotland: A national action plan. Edinburgh: Scottish Government, 2009.
28. Welsh Assembly Government. Designed for life: creating world class health and social care for Wales in the 21st Century. Cardiff: Welsh Assembly Government, 2005.
29. All Wales Palliative Care Planning Group. Report to Minister for health and social services on palliative care services – Sugar Report. Cardiff: Welsh Assembly Government, 2008.
30. All Wales Palliative Care Planning Group. Implementation of palliative care report: Palliative care services funding 2008 to 2009 - the Finlay Report. Cardiff: Welsh Assembly Government, 2009.
31. Welsh Government. Together for health: Delivering end of life care. Cardiff: Welsh Government, 2013.
32. Exley C. Review article: The sociology of dying, death and bereavement. *Sociology of Health and Illness* 2004;26(1):10–22.
33. Payne S. From here to there: reaching out for palliative care. *European Journal of Palliative Care* 2011;18(6):264–5.
34. Hannigan B, Edwards D, Anstey S, et al. End of life care for people with severe mental illness: an evidence synthesis (the MENLOC study) *Health Services and Delivery Research journal* 2021 in press
35. McWhinney IR. The value of case studies,. *The European Journal of General Practice* 2001; 7(3):88-89. doi: 10.3109/13814780109080866
36. Crowe S, Cresswell K, Robertson A, et al. The case study approach. *BMC Medical Research Methodology* 2011;11(1):100. doi: 10.1186/1471-2288-11-100
37. Moher D, Liberati A, Tetzlaff J, et al. Preferred reporting items for systematic reviews and meta-analyses: the PRISMA statement. *Ann Intern Med* 2009;151(4):264-9, w64. doi: 10.7326/0003-4819-151-4-200908180-00135 [published Online First: 2009/07/23]
38. Mahood Q, Van Eerd D, Irvin E. Searching for grey literature for systematic reviews: challenges and benefits. *Res Synth Methods* 2014;5(3):221-34. doi: 10.1002/jrsm.1106 [published Online First: 2015/06/09]
39. Centre for Research Dissemination. CRD's guidance on undertaking reviews in health care. York: University of York CRD, 2009.
40. Thomas J, Harden A. Methods for the thematic synthesis of qualitative research in systematic reviews. *BMC Medical Research Methodology* 2008;8(1):45. doi: 10.1186/1471-2288-8-45
41. Moola S, Munn Z, Tufanaru C, et al. Chapter 7: Systematic reviews of etiology and risk. In: Aromataris E, Munn Z, eds. JBI Manual for Evidence Synthesis. Adelaide: The Joanna Briggs Institute 2020.

For peer review only - http://bmjopen.bmj.com/site/about/guidelines.xhtml

For peer review only

42. Bakker TJ. Palliative care in chronic psycho-geriatrics: A case-study. *Patient Education and Counselling* 2000;41(1):107-13.
43. Feldman DB, Sorocco KH, Bratkovich KL. Treatment of posttraumatic stress disorder at the end-of-life: Application of the Stepwise Psychosocial Palliative Care model. *Palliative & Supportive Care* 2014;12(3):233-43.
44. Feldman DB. Stepwise psychosocial palliative care: A new approach to the treatment of posttraumatic stress disorder at the end of life. *Journal of Social Work in End-of-Life & Palliative Care* 2017;13(2-3):113-33.
45. Mason EN, Bowman EH. Determination of capacity in a critically ill patient with long standing mental illness. 2018 Annual Scientific Meeting: Journal of the American Geriatrics Society, 2018:S97.
46. Rodriguez-Mayoral O. Bipolar disorder and cancer. Management at end of life. Case report. *Bipolar Disorders* 2018;Conference(Supplement 1):79.
47. Kennedy P, Sarafi C, Greenish W. Homelessness and end of life care – Practical information and tools to support the needs of homeless people who are approaching the end of life, and those who are bereaved. London: St Mungos and Marie Curie, 2013.
48. Maloney K. Opinion: My husband refused to believe he had bipolar disorder, and denial destroyed him. TheJournalie. Ireland, 2014.
49. Badger JM, Ekham LR. Conflicting voices: Withhold treatment or not for a patient with chronic self-destructive behavior? *JONA'S Healthcare Law, Ethics and Regulation* 2011;13(3):79-83.
50. Boyd C, Cantone J, Gambrino P, et al. When the hospice patient is manipulative. *Hospice Journal* 1997;12(1):1-16.
51. Candilis PJ, Foti ME. Case presentation: End-of-life care and mental illness: The case of Ms. W. *Journal of Pain and Symptom Management* 1999;18(6):447-8.
52. Feely MA, Havyer RDA, Lapid MI, et al. Management of end-of-life care and of difficult behaviors associated with borderline personality disorder. *Journal of Pain and Symptom Management* 2013;45(5):934-8.
53. Feldman D, Periyakoil V. Posttraumatic stress disorder at the end of life. *Journal of palliative medicine* 2006;9 1:213-8.
54. Geppert CMA, Rabjohn P, Vlaskovits J. To treat or not to treat: Psychosis, palliative care, and ethics at the end-of-life: A case analysis. *Psychosomatics* 2011;52(2):178-84.
55. Gonzalez MR, Marcelo CD, Stringer M. Hospice care for 86 year old male with recurrent breast cancer: A case report. *Cases Journal* 2009;2(6):8357.
56. Griffith LJ. Good palliative care for a patient with schizophrenia dying of emphysema and heart failure. *Psychiatry* 2007;4(8):61-5.
57. Hill R. End-of-life care for the patient with borderline personality disorder. *Journal of Hospital and Palliative Nursing* 2005;7(3):150-63.
58. Kunkel EJ, Woods CM, Rodgers C, et al. Consultations for 'maladaptive denial of illness' in patients with cancer: psychiatric disorders that result in noncompliance. *Psycho-Oncology* 1997;6(2):139-49.
59. Levin SM, Feldman MB. Terminal illness in a psychiatric patient--issues and ethics. *South African Medical Journal* 1983;63(13):492-4.
60. Lopez A, Yager J, Feinstein RE. Medical futility and psychiatry: Palliative care and hospice care as a last resort in the treatment of refractory anorexia nervosa. *International Journal of Eating Disorders* 2010;43(4):372-7.
61. McCasland LA. Providing hospice and palliative care to the seriously and persistently mentally ill. *Journal of Hospice and Palliative Nursing* 2007;9(6):305-13.
62. McKenna RC, Bailey L, Haake J, et al. Clozapine and chemotherapy. *Hospital & Community Psychiatry* 1994;45(8):831.
63. Moini B, Levenson JL. A forgotten diagnosis: simple schizophrenia in a patient with breast cancer. *Psychosomatics* 2009;50(1):87-9.

For peer review only - http://bmjopen.bmj.com/site/about/guidelines.xhtml

64. Monga V. Clozapine and concomitant chemotherapy in a patient with schizophrenia and new onset esophageal cancer. *Psycho-Oncology* 2015;24(8):971-2.
65. Rice TR, Dobry Y, Novakovic V, et al. The importance of patient-provider communication in end-of-life care. *Journal of Bioethical Inquiry* 2012;9(4):439-41. doi: 10.1007/s11673-012-9397-4
66. Romm S, Combs H, Poeschla B. Psychiatry and palliative care: Thoughts on collaboration. *Journal of Hospital and Palliative Nursing* 2009;11(2):79-81.
67. Shah HA, Lee HBH, Nunery WR. Neglected basal cell carcinoma in a schizophrenic patient. *Ophthalmic Plastic & Reconstructive Surgery* 2008;24(6):495-7.
68. Stecker E. The terminally ill patient on an acute psychiatric unit. A case study. *Journal of Psychosocial Nursing and Mental Health Services* 1993;31(5):13-5.
69. Steves F, Williams T. Enhancing end-of-life care for terminally ill psychiatric patients. *Nursing* 2016;46(8):54-8.
70. Terpstra TL, Williamson S, ., Terpstra T. Palliative care for terminally ill individuals with schizophrenia. *Journal of Psychosocial Nursing and Mental Health Services* 2014;52(8):32-8.
71. Thomson K, Henry B. Oncology clinical challenges: Caring for patients with preexisting psychiatric illness. *Clinical Journal of Oncology Nursing* 2012;16(5):471-80.
72. Mogg A, Bartlett A. Refusal of treatment in a patient with fluctuating capacity - Theory and practice. *Journal of Forensic Psychiatry and Psychology* 2005;16(1):60-9.
73. Muhtaseb M, Olver JM, Constantine S. Massive basal cell carcinoma in a schizophrenic patient: Treatment options and constraints. *British Journal of Ophthalmology* 2001;85(1):117-8.
74. O'Neill J, Crowther T, Sampson G. A case study: anorexia nervosa. Palliative care of terminal psychiatric disease. *Am J Hosp Palliat Care* 1994;11(6):36-8. doi: 10.1177/104990919401100609 [published Online First: 1994/11/01]
75. Clements-Cortes A. The use of music in facilitating emotional expression in the terminally ill. *American Journal of Hospice & Palliative Medicine* 2004;21(4):255-60.
76. Kadri A, Blackmer J, Ibrahim M. Obtaining consent to a life-sustaining treatment for a patient with a major psychiatric illness. *CMAJ* 2014;186(14):1087-9.
77. Webber T. End of life care for people with mental illness. *Journal of Ethics in Mental Health* 2012;7:1-4.
78. Cabaret W, Krerbi Y, Saravane D. Palliative care in psychiatric institutions. *European Journal of Palliative Care* 2002;9(4):150-2.
79. Doron A, Mendlovic S. Dying in the psychiatric ward. *Israel Journal of Psychiatry and Related Sciences* 2008;45(2):141-4.
80. Ang A, Loke PCW, Campbell AV, et al. Live or let die: Ethical issues in a psychiatric patient with end-stage renal failure. *Annals of the Academy of Medicine Singapore* 2009;38(4):370-73.
81. Griffith LJ. Brief supportive psychotherapy for a patient with chronic schizophrenia who is dying. *Psychiatry* 2007;4(12):49-54.
82. Hannigan B, Evans N. Critical junctures in health and social care: Service user experiences, work and system connections. *Social Theory & Health* 2013;11(4):428-44. doi: 10.1057/sth.2013.16

For peer review only - http://bmjopen.bmj.com/site/about/guidelines.xhtml

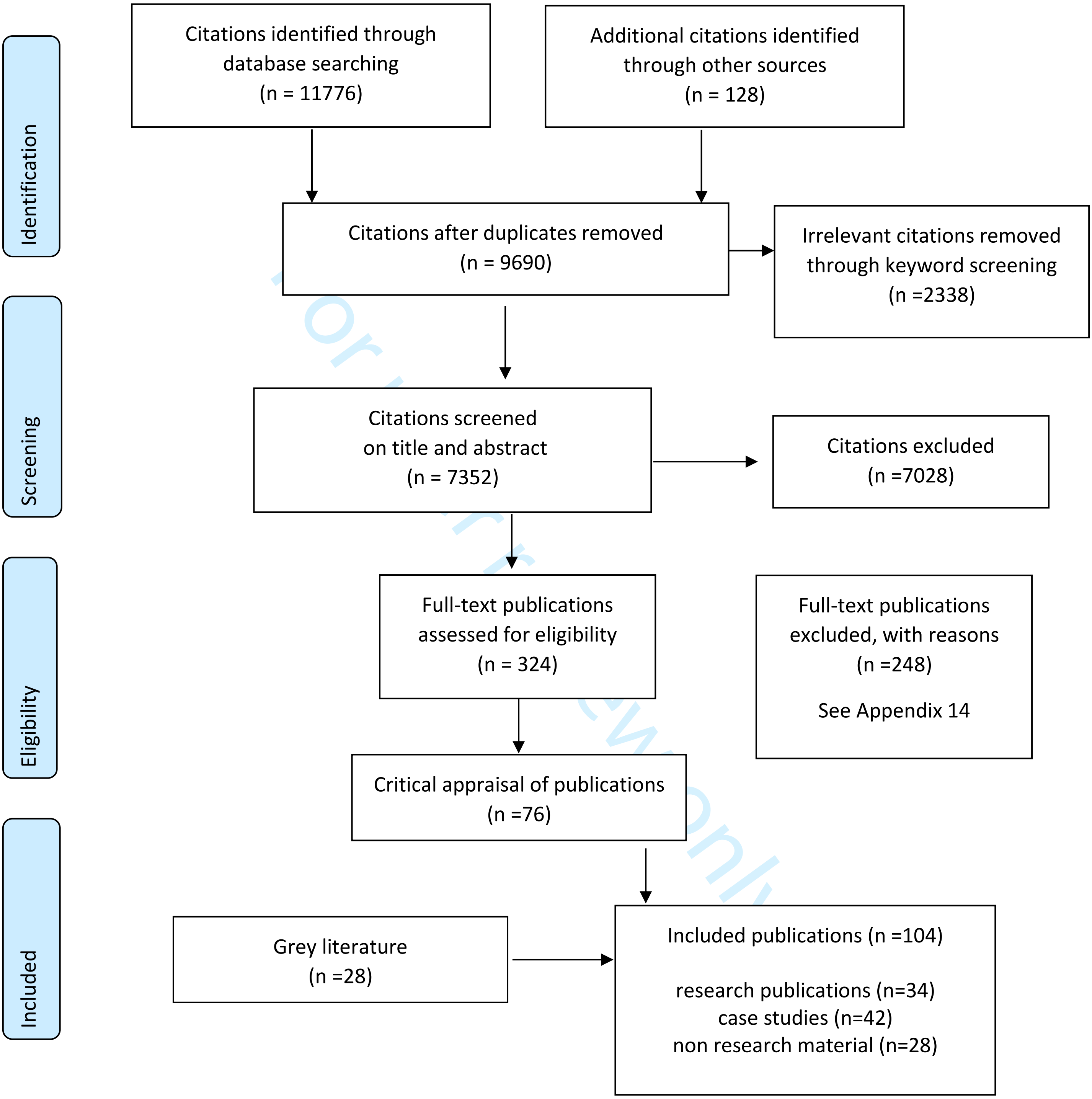

Figure 1: PRISMA Flow chart

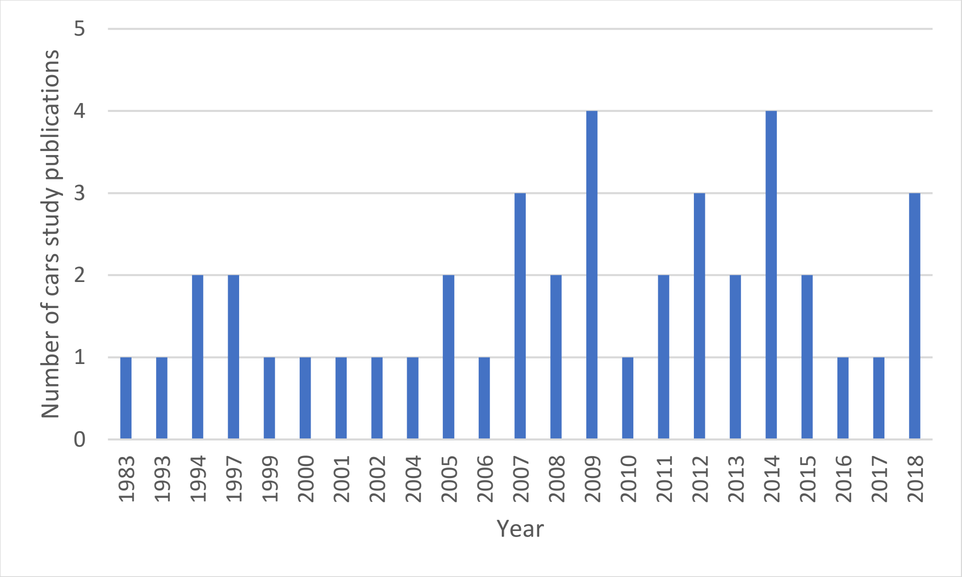

Figure 2: Histogram of year of publication of case studies

For peer review only

National Institute for Health Research (NIHR)
Health Services and Delivery Research (HS&DR) Programme

Protocol

Project title:	End of life care for people with severe mental illness: an evidence synthesis (the MENLOC study)
Project reference:	HS&DR Project 17/100/15
Version:	1.0
Date:	24 September 2018
Chief investigator:	Professor Ben Hannigan
Research team:	Dr Sally Anstey, Dr Paul Gill, Deborah Edwards, Mala Mann, Professor Michael Coffey, Alan Meudell, Roger Pratt
REC approval:	Not applicable
Start date:	1 November 2018
End date:	31 January 2020
Funding acknowledgement:	This project is funded by the NIHR HS&DR Programme (ref 17/100/15)
Department of Health and Social Care disclaimer:	The views expressed are those of the authors and not necessarily those of the NHS, the NIHR or the Department of Health and Social Care

Protocol version control:

Version	Date	Details
1.0	24.09.2018	Protocol reflecting full project description approved by HS&DR Board

For peer review only - http://bmjopen.bmj.com/site/about/guidelines.xhtml

HS&DR Project 17/100/15: End of life care for people with severe mental illness: an evidence synthesis (the MENLOC study)

FULL TITLE OF PROJECT

End of life care for people with severe mental illness: an evidence synthesis (the MENLOC study)

SUMMARY OF RESEARCH

The aim of this project is to synthesise relevant research and other appropriate evidence relating to the organisation, provision and receipt of end of life care for people with severe mental illness (including schizophrenia, bipolar disorder and other psychoses, major depression and personality disorder) who have an additional diagnosis of advanced, incurable, cancer and/or end-stage lung, heart, renal or liver failure and who are likely to die within the next 12 months. Outputs from the project will be tailored to stakeholders, and clear implications will be drawn for the future commissioning, organisation, management and provision of clinical care. Recommendations will be made for future data-generating studies designed to inform service and practice improvements, guidance and policy.

In this context, summary objectives are to:

1. locate, appraise and synthesise relevant research;
2. locate and synthesise policy, guidance, case reports and other grey and non-research literature;
3. produce outputs with clear implications for service commissioning, organisation and provision;
4. make recommendations for future research designed to inform service improvements, guidance and policy.

This review will be conducted according to the guidance developed by the Centre for Reviews and Dissemination (CRD) and will be reported following the Preferred Reporting Items for Systematic Reviews and Meta-Analyses (PRISMA) statement recommendations. Reflecting Evidence for Policy and Practice Information (EPPI) Centre principles, opportunities will also be embedded into the project to maximise stakeholder engagement for the purposes of both shaping its focus and maximising its reach and impact.

Searches will be developed initially using Medical Subject Headings (MeSH) and text words across health, social care and psychology databases from their inception. In consultation with a stakeholder advisory group, supplementary methods will be developed to identify additional material including policies, reports, expert opinion pieces and case studies. All English language items relating to the provision and receipt of end of life care for people with severe mental illness and an additional diagnosis of advanced, incurable, cancer and/or end-stage lung, heart, renal or liver failure will be included. All included citations will be assessed for quality using tools developed by the Critical Appraisal Skills Programme (CASP), or alternatives as necessary if suitable CASP tools are not available. Data will be extracted into tables, and subjected to meta-analyses where possible or thematic synthesis with help from NVivo. Strength of synthesised findings will be reported where possible using GRADE and CerQual.

Information derived from the processes described above will be drawn on in an accessibly written summary. Uniquely, this synthesis will comprehensively bring together evidence on factors facilitating and hindering high-quality end of life care for people with severe mental illness, who have an additional diagnosis of advanced, incurable, cancer and/or end-stage lung, heart, renal or liver failure, and evidence relating to services, processes, interventions, views and experiences. Implications will be stated for the improvement of relevant NHS and third sector care and recommendations will be made for future research.

BACKGROUND AND RATIONALE

We plan a rigorous synthesis of research and other evidence conducted according to internationally agreed quality standards. The project is within the HS&DR Programme's remit in addressing quality, organisation and access in health services. In the context of calls for parity of esteem between mental health and physical health care (Mental Health Taskforce, 2016) the health problem this project addresses is a highly relevant, but also neglected, one.

For peer review only - http://bmjopen.bmj.com/site/about/guidelines.xhtml

HS&DR Project 17/100/15: End of life care for people with severe mental illness: an evidence synthesis (the MENLOC study)

In preparing this proposal an initial scoping review was undertaken (with updated searches run in July 2018), and a targeted search was made for relevant policy documents across the four UK nations. A search of the database of NIHR projects was conducted to check for overlapping or related studies. Searches of four databases (combining 'palliative care', 'mental health' and 'service provision' terms: see 'Search strategy' below) produced 4,754 citations, contained within which are numbers of relevant papers including two previous literature reviews (from the UK and Canada respectively), both now out-of-date having been published a decade ago (Ellison, 2008, Woods et al., 2008). Items discovered in this scoping search confirm the timeliness and feasibility of a new, rigorous, evidence synthesis: and particularly an EPPI-Centre style review which is sensitive to the needs of stakeholders and which includes grey and non-research materials (Gough et al., 2017). Items from this initial search, combined with general material addressing what is known about the burden of disease and the physical health of people living with mental health difficulties, have been used to inform this background and rationale (and subsequent) sections of this project proposal.

Burden of disease and costs

The overarching background for this project includes what is already known about the burden of severe mental illness, cancer and end-stage lung, heart, renal or liver failure. Mental ill-health is a leading cause of years lived with disability (YLD) around the world, with major depressive disorder the leading cause of YLD in 56 countries and the second leading cause in a further 56 (Global Burden of Disease Study 2013 Collaborators, 2015). Specific cancers, along with mental health, neurological and drug use disorders and specific organ diseases all feature in the leading 20 causes of disability adjusted life years (DALYs) in England for 2013 (Newton et al., 2015). The wider economic costs of mental illness in England were recently estimated at £105.2 billion each year (Centre for Mental Health, 2010). This figure combines the direct costs of services, lost productivity at work and reduced quality of life with the annual costs of the same in Wales estimated at £7.2 billion (Friedli and Parsonage, 2009).

Meeting the physical health needs of people living with severe mental illness

The term 'severe [or, often used interchangeably, 'serious' or 'serious and enduring'] mental illness' (SMI) as used throughout this project proposal has longstanding currency within the fields of mental health policy, services and practice dating back at least as far as the publication of *Building Bridges* (Department of Health, 1995). It continues to be used in research (see for example: Kronenberg et al., 2017), and has currency with the NIHR Dissemination Centre which published a Themed Review into Severe Mental Illness in 2018 (National Institute for Health Research, 2017). *Building Bridges* recognised the imprecision of the term 'severe mental illness', and endorsed a multidimensional framework definition encompassing five areas: safety; need for informal or formal care; disability; diagnosis; and duration. Diagnosis is therefore an important, but not the only, dimension used in the identification of people with severe mental illness and includes ICD-10 diagnoses of schizophrenia, schizotypal and delusional disorders, bipolar affective disorder, and disorders of adult personality and behaviour (World Health Organization, 1992) along with similar DSM-5 diagnoses including schizophrenia spectrum and other psychotic disorders, bipolar and related disorders, and major depressive disorder (American Psychiatric Association, 2014).

People with severe mental illness have high co-morbidities (Ellison, 2008), and experience higher mortality rates and reduced life expectancy compared to the general population (Taylor et al., 2012, Thornicroft, 2013). Higher mortality and morbidity rates for this group have been found across all age groups (Picot et al., 2015), with a 10-20 year reduction in life expectancy reported (Chesney et al., 2014). Inequities can be explained with reference to individual and system-level factors. People with severe mental illness are less likely to attend health screenings and may respond to symptoms differently (Disability Rights Commission, 2006, Carney et al., 2006). They may delay or avoid help, and are more likely to exhibit noncompliant and disruptive behaviours (Woods et al., 2008, Millman et al., 2016) putting them at risk of delayed disease detection (Ellison, 2008). Inadequate support systems are also common among those with severe mental illness, affecting their ability to access appropriate clinical care and navigate complex health systems (Knapik and Graor, 2013). Other factors influencing variations in mortality and morbidity for people living with severe mental illness include poor previous experiences of seeking help from health care professionals, the incorrect

For peer review only - http://bmjopen.bmj.com/site/about/guidelines.xhtml

attribution of physical symptoms to psychiatric disorder by workers and lack of experience by mental health professionals in determining how and when to refer onwards to other appropriate services (Woods et al., 2008, Shalev et al., 2017).

End of life care for people with severe mental illness

In this study end of life care is used to refer to the care of people with diagnoses of advanced, incurable, cancer and/or end-stage lung, heart, renal or liver failure and who are likely to die within the next 12 months. It includes care provided in hospitals, hospices and other institutional settings (such as prisons and hostels) and care provided in the home and via outreach to people who may also be homeless.

Beyond the inequities identified above, commitments to parity of esteem demand that end of life care for people with severe illness should be as timely and as high-quality as it is for others. However, evidence suggests that this group is poorly served, with England's Cancer Strategy 2015-2020 (Independent Cancer Taskforce, 2015) recognising people with severe mental illness as needing a targeted focus to improve diagnosis and care. Although cancer incidence amongst people living with chronic mental health difficulties is similar to that of the general population, mortality rates are double (Howard et al., 2010, Batty et al., 2012). This disparity may be related in part to late presentation and reduced use of interventions such as surgery, chemotherapy or radiotherapy (Desai et al., 1999, Irwin et al., 2014). The experience of severe mental illness can delay detection and treatment of life-threatening physical disorders as people are less likely to seek treatment, to verbalise pain and to access timely healthcare (Foti, 2012). Consequently, this patient cohort is more likely to present with more advanced cancers which are invariably more complex and costly to treat, with patients less likely to undergo invasive treatments and more likely to die (Ishikawa et al., 2016). Some cancers, other terminal conditions and/or related treatments may also compound mental illness and precipitate potential 'problematic' behaviours (McCormack and Sharp, 2006). For many patients, palliative care is therefore often the only meaningful treatment option available.

Once in touch with end of life services the symptoms of people with severe mental illness may be poorly recognised and undertreated, with staff working in end of life services lacking knowledge, training and experience in this area (Addington-Hall, 2000). Undetected and hence untreated mental illness can jeopardise treatment outcomes, reduce patient satisfaction and increase health care costs (Zabora et al., 2000). Variable adherence can be a complicating factor (Knapik and Graor, 2013), compounded by comorbid disorders such as substance misuse and social factors such as homelessness, isolation or lack of transportation all of which can exert an impact on care planning for end of life care and treatment (Woods et al., 2008, Picot et al., 2015). Assumptions about the capacity of people with severe mental illness to make end of life decisions, and concerns that end of life discussions would be too distressing or exacerbate mental health problems, may lead to inadequate consultation (Morgan, 2016). This is reflected in the fact that people with severe mental illness have a higher percentage of 'do not resuscitate' orders than other groups and are less likely to have had discussions about their explicit wishes for end of life care (Warren et al., 2014).

Palliative care

Whilst not all people at the end of life need palliative care an NAO survey (Dixon et al., 2015) confirmed inequities in palliative care provision for both cancer and non-cancer patient populations. In Wales, an estimated 24,000 of the 32,000 people dying would benefit from palliative care but over a quarter do not have access to it (Hughes-Hallet et al., 2011, Dixon et al., 2015). Access to palliative care services for people who die from cancer is 46% compared to 5% from those dying from other conditions (Marie Curie and the Bevan Foundation, 2014), including end-stage lung, heart, renal or liver failure.

People with mental illness and advanced incurable cancer and/or lung, heart, renal or liver failure face inequities and discrimination (Hughes-Hallet et al., 2011) and a lack of integrated care (Ellison, 2008). Some may be excluded from end of life care planning (Woods et al., 2008), and even from hospitals or hospices entirely (McCormack and Sharp, 2006). They may be referred back to mental health services, where staff are largely inadequately prepared to provide appropriate end of life care (Woods

For peer review only - http://bmjopen.bmj.com/site/about/guidelines.xhtml

et al., 2008). Difficulties accessing appropriate services mean that for people with mental illness and life-threatening disease palliative care may be the first line of treatment (Ellison, 2008). The specific provision of palliative care for people with severe mental illness is known to be poorer than for the general population. People with severe mental illness are approximately 50% less likely to access appropriate palliative care, including symptom control and pain relief (Woods et al., 2008, Shalev et al., 2017). Palliative care and hospice staff often feel unskilled (Shalev et al., 2017), and lack confidence and training in conducting discussions about end-of-life care with people severe mental illness (Morgan, 2016). Existing evidence also indicates that there is a lack of coordinated end of life care and access to appropriate psychosocial support is often limited (Ellison, 2008, Woods et al., 2008). Medical and nursing staff working in hospices have also been shown to be unprepared for working with people with severe mental illness, basing their assessments on instinct rather than using evidence-based approaches (Hackett and Gaitan, 2007). In a survey of psychological services in hospices in the UK and Republic of Ireland only 30% of hospices had access to a psychiatrist, whilst 41% had access to a clinical psychologist and 45% had neither (Price et al., 2006). Patient experience data underscores these observations, with England's National Cancer Patient Experience Survey showing that people with a long-term mental health condition (2% of those surveyed, n=1184) reporting less positive experiences of cancer care (Department of Health, 2010). In Wales, the most recent National Cancer Patient Experience Survey found that the lowest proportion of respondents reporting positive experiences of their cancer care were those identifying as also having mental health problems (Picker Institute Europe, 2017).

EVIDENCE EXPLAINING WHY THIS RESEARCH IS NEEDED NOW

Research at the interface of physical and mental health is recognised as a UK priority (Department of Health, 2017). Against the background presented above, this project will create generalisable knowledge to improve end of life care and services for an underserved group. Policies from the four UK governments focus on improving end of life care, where diagnosis is immaterial (Department of Health, 2016, Northern Ireland Executive, 2010, Scottish Government, 2008b, Scottish Government, 2008a, Scottish Government, 2009, Welsh Assembly Government, 2005, All Wales Palliative Care Planning Group, 2008, All Wales Palliative Care Planning Group, 2009, Welsh Government, 2013). These require the introduction of palliative and supportive care earlier in the illness trajectory, with patient surveys showing that this is rated very highly by those receiving it (Drakeford, 2014). Marie Curie identified triggers that should initiate palliative care for people with diagnoses other than cancer (Marie Curie, 2015), but apart from dementia they do not mention those with pre-existing severe mental illness. In national policy the particular needs of people with severe mental illness who develop advanced incurable cancer and/or end-stage lung, heart, renal or liver failure are acknowledged poorly, or not at all. This group face the prospect of 'disadvantaged dying' (Exley, 2004, Payne, 2011), at a time when quality of care in the last months of life should be uniformly high for all groups.

Uniquely amongst the cancer, palliative and end of life strategies developed across the four countries of the UK, England's Cancer Strategy 2015-2020 makes the specific recommendation that the NIHR commission research in the area of cancer care for people living with severe mental illness (Independent Cancer Taskforce, 2015). This project responds to this call, and expands it to also cover end of life care for people living with severe mental illness and facing end-stage lung, heart, renal or liver failure. It will answer a question which is both timely and relevant: what evidence is there relating to the organisation, provision and receipt of care for people with severe mental illness who have an additional diagnosis of advanced, incurable, cancer and/or end-stage lung, heart, renal or liver failure and who are likely to die within the next 12 months?

Our rigorously conducted evidence synthesis will bring together reports of approaches to service organisation, processes and interventions shown to both facilitate and hinder the provision of high-quality, accessible, equitable and acceptable end of life care to people with severe mental illness. We will also gather research and other evidence reporting the views and experiences of service users, families and health and social care staff. The project will have an impact on services and practice by presenting its findings in accessible ways to NHS and other managers, practitioners and educators. We anticipate findings will inform future NICE guidelines, and thereby help shape the provision of services. Current, relevant, NICE guidance (National Institute for Clinical Excellence, 2004, National Institute for Health and Care Excellence, 2009, National Institute for Health and Care Excellence, 2011, National Institute for Health and Care Excellence, 2014b, National Institute for Health and Care

For peer review only - http://bmjopen.bmj.com/site/about/guidelines.xhtml

Excellence, 2014a) lacks standards or recommendations particularly addressing end of life care for people with severe mental illness, with documents supporting in-progress guideline development (National Institute for Health and Care Excellence, 2018 [anticipated]) also lacking a focus in this area. We anticipate findings from this study will help redress this, informing future guidance and thereby shaping the provision of services.

A search of NIHR databases finds studies which have investigated: the physical health of people with severe mental illness (e.g., HTA 12/28/05); services for this group across organisations (e.g., HS&DR 11/1023/13); and care for people experiencing mental health difficulties after receiving cancer diagnosis (e.g., HTA 09/33/02). As no research has been commissioned in the area we propose here, this project effectively begins a new and important field of work with value to the NHS and its partners. Using a methodical, systematic and transparent approach our team plans to use this study as the starting point for a programme of research which builds on the existing literature, is designed with people who have experience of mental health difficulties and with people who have lived with cancer and have cared for family at the end of life, and which generates new evidence of what works with value to the NHS and other relevant organisations. Further studies planned include data generating investigations into the provision and receipt of end of life care for people with severe mental illness.

AIMS AND OBJECTIVES

The aim of this project is to synthesise relevant research and other appropriate evidence relating to the organisation, provision and receipt of end of life care for people with severe mental illness (including schizophrenia, bipolar disorder and other psychoses, major depression and personality disorder). Specifically, it will answer the question, ‘what evidence is there relating to the organisation, provision and receipt of care for people with severe mental illness who have an additional diagnosis of advanced, incurable, cancer and/or end-stage lung, heart, renal or liver failure and who are likely to die within the next 12 months?

Detailed objectives are to:

1. use internationally recognised, transparent, literature review approaches to locate, appraise and synthesise the relevant research evidence relating to the organisation, provision and receipt of care in the expected last year of life for people with severe mental illness who have additional diagnoses of advanced, incurable, cancer and/or end-stage lung, heart, renal or liver failure and who are likely to die within the next 12 months;
2. locate and synthesise policy, guidance, case reports and other grey and non-research literature relating to the organisation, provision and receipt of care in the expected last year of life for people with severe mental illness who have additional diagnoses of advanced, incurable, cancer and/or end-stage lung, heart, renal or liver failure and who are likely to die within the next 12 months;
3. produce outputs with clear implications for service commissioning, organisation and provision;
4. make recommendations for future research designed to inform service improvements, guidance and policy.

RESEARCH PLAN/METHODS

Quality and standards

The protocol for this evidence synthesis will be registered with the International Prospective Register of Systematic Reviews (PROSPERO) (PROSPERO: International prospective register of systematic reviews, undated). It will be conducted with the involvement of colleagues from the Cardiff Specialist Unit for Review Evidence (SURE). It will follow guidance for undertaking reviews in health care published by the Centre for Reviews and Dissemination (CRD) (2009) and will use methods informed by the Evidence for Policy and Practice Information (EPPI) Centre (Gough et al., 2017). To ensure rigour the review will be reported following the Preferred Reporting Items for Systematic Reviews and Meta-Analyses (PRISMA) statement (Moher et al., 2009). Findings will be accessibly presented, with the help of a stakeholder advisory group and the NIHR Dissemination Centre, to ensure reach. Factors facilitating and hindering high-quality end of life care for people with severe mental illness will

For peer review only - http://bmjopen.bmj.com/site/about/guidelines.xhtml

be identified, along with evidence relating to services, processes, interventions, views and experiences.

Search strategy

The search strategy will be comprehensive and designed to ensure that all relevant literature is obtained. While some terminology is equivocal in this area, the search strategy will be designed to identify the relevant evidence relating to end of life care (i.e., in the last year of life) in those with severe mental illness. Reflecting the importance of diagnosis in the framework definition of severe mental illness, diagnostic terms to be included in the search strategy include: schizophrenia, schizophrenia spectrum and other psychotic disorders, schizotypal and delusional disorders, bipolar affective disorder, bipolar and related disorders, major depressive disorder and disorders of adult personality and behaviour. Reflecting prevailing definitions of severe mental illness (for example, as used by the NIHR Dissemination Centre) searches will not be made for studies into mental and behavioural disorders due to psychoactive substance use or for studies into dementia or other neurodegenerative diseases, and items in these areas will not be included in the review except where participants' diagnoses coexist with the disorders included above. To ensure appropriateness of evidence, inclusion/exclusion criteria have been developed (Table 1):

Table 1: Inclusion/exclusion criteria

Inclusion	Exclusion
Relevant evidence specifically relating to adult participants (>18 years of age) with severe mental illness (including schizophrenia, schizophrenia spectrum and other psychotic disorders, schizotypal and delusional disorders, bipolar affective disorder, bipolar and related disorders, major depressive disorder and disorders of adult personality and behaviour) who have an additional diagnosis of advanced, incurable, cancer and/or end-stage lung, heart, renal or liver failure and who are likely to die within the next 12 months.<br><br>Published in English Language only.	Where reporting allows the distinction to be made, mental health problems (e.g. depression) as a consequence of terminal illness (e.g., cancer or chronic organ failure).<br><br>Evidence relating to end of life care for people with mental and behavioural disorders due to psychoactive substance use, except where these coexist with disorders included in the column to the left.<br><br>Evidence relating to end of life care for people with dementia or other neurodegenerative diseases except where these coexist with disorders included in the column to the left.<br><br>Evidence from animal studies.

Comprehensive searches will be conducted across multiple databases from their inception, including: Ovid Medline; Ovid Allied and Complementary Medicine Database (AMED); Proquest Applied Social Sciences Index and Abstracts (ASSIA); Ebsco Cumulative Index of Nursing and Allied Health Literature (CINAHL); Wiley Cochrane Central Register of Controlled Trials; Database of Abstracts of Reviews of Effectiveness; Ovid Embase; Ovid Health Management Information Centre (HMIC); Ovid MEDLINE Epub Ahead of Print and In-Process and Other Non-Indexed Citations; Ovid PsycInfo; Web of Knowledge Science Citation Indexes. A preliminary search will be developed in Ovid Medline using MeSH and text words as piloted in our initial scoping (see 'Background and Rationale' above, and example of search strategy below), then translated to other databases.

Supplementary searches will be undertaken to identify additional papers, information on studies in progress, unpublished research or research reported in the grey literature. Relevant websites and trial registers will be searched (e.g., NIH ClinicalTrials.gov (http://www.clinicaltrials.gov), metaRegister of Controlled Trials (http://www.controlled-trials.com), WHO International Clinical Trials Registry Platform (ICTRP) (http://www.who.int/ictrp/en). To identify published resources that have not yet been catalogued in the electronic databases, recent editions of key journals will be hand-searched.. Reference lists of included studies will be scanned, experts contacted, and forward citation tracking performed using ISI Web of Science.

For peer review only - http://bmjopen.bmj.com/site/about/guidelines.xhtml

HS&DR Project 17/100/15: End of life care for people with severe mental illness: an evidence synthesis (the MENLOC study)

Reflecting EPPI Centre (Gough et al., 2017) methods, searches will be conducted for non-research material (e.g., social media, personal accounts, policies) using transparent, clearly described, approaches (Mahood et al., 2014). With advice being taken from members of a project stakeholder advisory group, relevant websites will be searched using search terms and strategies carefully tested and refined as necessary to ensure that all items relevant to the care of people with severe mental illness and (a) advanced, incurable, cancer and/or (b) end-stage lung, heart, renal or liver failure and who are likely to die within the next 12 months are equally included. Candidate websites for searching include: the Care Quality Commission (https://www.cqc.org.uk/), Cancer Research UK (http://www.cancerresearchuk.org/), Cancer Research Wales (https://www.cancerresearchwales.co.uk/), Tenovus Cancer Care (http://www.tenovuscancercare.org.uk/), Mental Health Foundation (https://www.mentalhealth.org.uk/), Centre for Mental Health (https://www.centreformentalhealth.org.uk/), St Mungo's (https://www.mungos.org/), National Kidney Foundation (https://www.kidney.org/), British Liver Trust (https://www.britishlivertrust.org.uk/), British Renal Society (http://britishrenal.org/), The Renal Association (https://renal.org/), British Heart Foundation (https://www.bhf.org.uk/), British Lung Foundation (https://www.blf.org.uk/), National Council for Palliative Care (http://www.ncpc.org.uk/), Hospice UK (http://www.hospiceuk.org/), Marie Curie (https://www.mariecurie.org.uk/), Macmillan Cancer Support (http://www.macmillan.org.uk/) and the Scottish Partnership agency for Palliative Care (https://www.palliativecarescotland.org.uk/).

Preliminary database searching using some of the methods described here has already been carried out, as part of an initial scoping undertaken in preparation of this proposal (see below). The initial search strategy once the project commences will build on this, and it is anticipated that the following MeSH terms and keywords will be used, in various combinations, to inform the systematic search process:

- Palliative care
- Hospice care
- Terminal care
- Terminally ill
- End of life care
- Last year of life
- Neoplasms
- Cancer
- Multiple organ failure
- Mental health
- Depression
- Mental disorders
- Depressive disorder
- Personality disorders
- Bipolar disorder
- Schizophrenia
- Mental illness

In July 2018 we took the opportunity to update and extend our scoping exercise (using terms and keywords reproduced above) using four databases: Ovid MEDLINE; Ovid MEDLINE Epub Ahead of Print, In-Process and Other Non-Indexed Citations and Ovid MEDLINE without Revisions; PsycINFO; and EMBASE. To illustrate this process we reproduce below the search run in MEDLINE, which produced 1,387 citations (before the removal of duplicates) for screening:

Database: Ovid MEDLINE(R) ALL <1946 to July 05, 2018>
Search Strategy:

----

1 exp Palliative care/ (48615)
2 exp Hospice care/ (5807)
3 exp Terminal Care/ (47347)
4 exp Terminally ill/ (6150)
5 ("palliative care" or "hospice care" or "end of life care" or end-of-life).tw. (38005)

For peer review only - http://bmjopen.bmj.com/site/about/guidelines.xhtml

6 ((hospice or terminal*) adj3 (care or caring or ill*)).tw. (12527)
7 ("last year of life" or LYOL or "end of life" or "end of their lives").tw. (19101)
8 (end-stage disease* or end stage disease* or end-stage ill* or end stage ill*).tw. (1112)
9 or/1-8 (104642)
10 exp Neoplasms/ (3055654)
11 (cancer* or tumo?r* or neoplas* or malignan* or carcinoma* or adenocarcinoma* or adeno?carcinoma* or choriocrcinoma* or leukemia* or leukaemia* or metastat* or sarcoma* or teratoma* or lymphoma).tw. (3168623)
12 exp Multiple Organ Failure/ (10043)
13 Organ failure.tw. (16597)
14 Pulmonary Disease, Chronic Obstructive/ (32992)
15 (Chronic obstructive pulmonary disease or COPD).tw. (53007)
16 Heart Failure/ (105052)
17 Renal Insufficiency/ (14550)
18 Liver Failure/ (6735)
19 ("cardiac failure" or "liver failure" or "kidney failure" or "renal failure").tw. (114515)
20 Chronic Disease/ (248675)
21 Health service utilization.mp. (1502)
22 lifestyle-related factor*.mp. (337)
23 or/10-22 (4430236)
24 exp Bipolar Disorder/ (37229)
25 exp Schizophrenia/ (97818)
26 (bipolar or mania or Schizophrenia).tw. (148564)
27 exp Depression/ (102372)
28 exp Mental Disorders/ (1125241)
29 ("mental illness" or "mental disorder*" or depression).tw. (333320)
30 mental health condition*.tw. (1794)
31 exp Depressive Disorder/ (99383)
32 exp Mental Health/ (31108)
33 exp Personality Disorders/ (39084)
34 (severe and persistent mental illness).mp. (304)
35 severe mental illness.mp. (3558)
36 or/24-35 (1390277)
37 9 and 23 and 36 (2648)
38 (dementia or Alzheimer).tw. (106344)
39 (Algeria$ or Egypt$ or Liby$ or Morocc$ or Tunisia$ or Western Sahara$ or Angola$ or Benin or Botswana$ or Burkina Faso or Burundi or Cameroon or Cape Verde or Central African Republic or Chad or Comoros or Congo or Djibouti or Eritrea or Ethiopia$ or Gabon or Gambia$ or Ghana or Guinea or Keny$ or Lesotho or Liberia or Madagasca$ or Malawi or Mali or Mauritania or Mauritius or Mayotte or Mozambiq$ or Namibia$ or Niger or Nigeria$ or Reunion or Rwand$ or Saint Helena or Senegal or Seychelles or Sierra Leone or Somalia or South Africa$ or Sudan or Swaziland or Tanzania or Togo or Ugand$ or Zambia$ or Zimbabw$ or China or Chinese or Hong Kong or Macao or Mongolia$ or Taiwan$ or Belarus or Moldov$ or Russia$ or Ukraine or Afghanistan or Armenia$ or Azerbaijan or Bahrain or Cyprus or Cypriot or Georgia$ or Iran$ or Iraq$ or Jordan$ or Kazakhstan or Kuwait or Kyrgyzstan or Leban$ or Oman or Pakistan$ or Palestin$ or Qatar or Saudi Arabia or Syria$ or Tajikistan or Turkmenistan or United Arab Emirates or Uzbekistan or Yemen or Bangladesh$ or Bhutan or British Indian Ocean Territory or Brunei Darussalam or Cambodia$ or India$ or Indonesia$ or Lao or People's Democratic Republic or Malaysia$ or Maldives or Myanmar or Nepal or Philippin$ or Singapore or Sri Lanka or Thai$ or Timor Leste or Vietnam or Albania$ or Andorra or Bosnia$ or Herzegovina$ or Bulgaria$ or Croatia$ or Faroe Islands or Greenland or Liechtenstein or Lithuani$ or Macedonia or Malta or maltese or Romania or Serbia$ or Montenegro or Svalbard or Argentina$ or Belize or Bolivia$ or Brazil$ or Chilean or Colombia$ or Costa Rica$ or Cuba or Ecuador or El Salvador or French Guiana or Guatemala$ or Guyana or Haiti or Honduras or

For peer review only - http://bmjopen.bmj.com/site/about/guidelines.xhtml

Jamaica$ or Nicaragua$ or Panama or Paraguay or Peru or Puerto Rico or Suriname or Uruguay or Venezuela or developing countr$ or south America$).ti,sh. (1190707)

40 Academic Dissertations/ (0)
41 thesis.tw. (7038)
42 book.pt. (0)
43 Books/ (3122)
44 or/38-43 (1303105)
45 37 not 44 (2373)
46 limit 45 to (English language and humans and "all adult (19 plus years)") (1387)

Combining these 1,387 citations with citations from the remaining three databases produced a total of 4,754 citations, which reduced to 3,033 once duplicates had been removed with the help of the bibliographic software programme EndNote. Screening based on titles alone reduced this total to 55. With titles and abstracts entered into the software screening programme Covidence, these 55 were reduced to 26 citations potentially meeting our inclusion criteria. Items identified clearly confirm the existence of a literature ready to be synthesised, and include (as illustrative examples) a report into health care use by people with schizophrenia at the end of life (Chochinov et al., 2012), a qualitative study of end of life care perspectives amongst people living with the diagnosis of schizophrenia (Sweers et al., 2013), an analysis of routine health records to examine associations between pre-existing mental illness and service use at the end of life (Lavin et al., 2017) and a whole-population study conducted in Taiwan to explore patterns of end of life care use amongst people with, and without, a diagnosis of schizophrenia (Huang et al., 2018).

Relevant items were also found in July 2018 by following Mahood et al.'s (2014) methods for the searching of grey literature by combining "end of life care" AND "severe mental illness" in the Google UK search engine, with examples including presentations from Canada [http://www.shared-care.ca/files/2C_-_Optimizing_end_of_life_care_(Whitehead).pdf] and New Zealand [http://www.nzcmhn.org.nz/files/file/1105/End%20of%20Life%20care%20for%20people%20with%20SPMI.pdf]. Relevant grey literature documents produced by statutory bodies and charities were also identified via targeted searching of organisational websites. Examples are: a recent review into end of life care produced by the Care Quality Commission (2016a), which contains a report in which carers of people also living with mental illness describe their experiences (Care Quality Commission, 2016b); and a joint report produced by St Mungo's and Marie Curie into end of life care for homeless people, which includes a case study of a woman with schizophrenia and cervical cancer (Kennedy et al., 2013).

Screening

As in our scoping, all citations retrieved will be imported into EndNote, where duplicate references will be removed. All remaining items will then be independently assessed for relevance by two members of the review team using the information provided in the title and abstract. Where any doubt exists the full text will be retrieved. In all cases the full text will be retrieved for all citations that, at this stage, appear to meet the review's inclusion criteria. To achieve a high level of consistency reviewers will screen each retrieved citation for inclusion using a purposely designed form. Disagreements will be resolved through discussion with a third reviewer. Authors of research studies will be contacted by the project team if further information is required. All English language items relating to the provision and receipt of end of life care for people with severe mental illness and an additional diagnosis of advanced, incurable, cancer and/or end-stage lung, heart, renal or liver failure will be included at this stage.

Quality appraisal

Following searching and screening, information from research publications will be independently assessed for methodological quality by two reviewers using design-specific tools developed by the Critical Appraisal Skills Programme (CASP) (2016). Alternative tools, reflecting the specific design and methods used in individual research outputs, will be used as necessary if suitable CASP tools are not available. Any disagreement on quality will be resolved through discussion with a third reviewer.

For peer review only - http://bmjopen.bmj.com/site/about/guidelines.xhtml

At this stage all research items will be included other than those which are fatally flawed. Non-research evidence (e.g. policies, reports, expert opinion pieces, case studies etc.) will not be subjected to quality appraisal.

Data extraction

All data will be extracted directly into tables and will follow the format recommended by the CRD (2009). One reviewer will extract the data and a second reviewer will independently check the data extraction forms for accuracy and completeness. Any disagreements will be noted and resolved by consensus within the review team.

Data analysis and synthesis

Researchers informed by the EPPI-Centre approach recognise that different strategies exist for the analysis and synthesis of data (Gough et al., 2017). The synthesis in this review will have both configurative (involving the exploration of potentially heterogeneous materials) and aggregative (involving the pooling of data, where possible) elements (Gough et al., 2012).

For intervention studies meta-analyses of data will be performed where possible. Tests for heterogeneity will be applied. Where statistical pooling is not possible the findings, along with data from non-intervention quantitative studies, will be thematically presented (Thomas and Harden, 2008). The software programme NVivo will be used to help manage this process. Qualitative data, and data from non-research items, will presented in configurative fashion using a thematic approach again assisted by NVivo. Themes will be developed inductively based on close reading of the content of all items included.

Assessing confidence

The strength of findings from the meta-analysis of intervention studies will be assessed using the Grading of Recommendations, Assessment, Development and Evaluation (GRADE) approach (Guyatt et al., 2008). The strength of synthesised qualitative and non-intervention findings will be assessed using the Confidence in the Evidence from Reviews of Qualitative Research (CerQual) approach (Lewin et al., 2015). The original CerQual approach was designed for qualitative findings, but we will use a process previously used by members of this research team in HS&DR 11/1024/08 (Hannigan et al., 2015) and in HS&DR 08/1704/211 (Edwards et al., 2014) in additionally adopting CerQual for the assessment of the confidence of synthesised findings from surveys and other non-intervention quantitative studies.

Overall summary

An overarching summary will bring all elements together. It will present key themes arising from this project as a whole, and do so in accessible manner to ensure reach. Help with this will be sought from the stakeholder advisory group and from the NIHR Dissemination Centre. Factors helping and hindering end of life care for people with severe mental illness will be identified, along with evidence relating to services, processes, interventions, views and experiences. Implications will be stated, and recommendations made for future research.

DISSEMINATION AND PROJECTED OUTPUTS

The main output from this project will be a comprehensive, rigorously conducted, synthesis of research and other evidence relating to services, interventions, processes, views and experiences in the context of end of life care for people with severe mental illness who have an additional diagnosis of advanced, incurable, cancer and/or end-stage lung, heart, renal or liver failure and who are likely to die within the next 12 months.

We will work with the NIHR Dissemination Centre to share findings from our study and to make sure they have maximum benefit, and will follow NIHR guidance by paying close attention to stakeholder

For peer review only - http://bmjopen.bmj.com/site/about/guidelines.xhtml

engagement, format, opportunities, context and timing (National Institute for Health Research, undated). Our stakeholders are policymakers, commissioners, managers and practitioners at the interface of the end of life and mental health care fields, along with people using services and carers. We plan the setting up of a stakeholder advisory group (numbering an anticipated 8-12 individuals), building on preliminary discussions held with clinical and managerial leads at relevant, South Wales-based, NHS and charity mental health services, cancer centres and palliative care organisations. Our list of candidate bodies from which stakeholder representation has been secured (or with which we will otherwise engage in order to keep members informed of our project's progress) currently includes: the National End of Life Care Programme Board in England; the End of Life Care Implementation Board in Wales; the National Mental Health Partnership Board in Wales; and the Mental Health and Dementia Programme Board in England. Professional, academic and management stakeholder organisations include: Mental Health Nurse Academics UK (for which chief applicant Ben Hannigan is elected Vice Chair and Chair-elect, and for which co-applicant Michael Coffey is a past Chair); the European Oncology Nursing Society (the immediate past President of which is a colleague of the applicants at Cardiff University); the Palliative Care Research Society UK, and the National Council for Palliative Care. Roger Pratt and Alan Meudell, as service user and carer co-applicants, will be invited to help identify patient and public representatives to also join this stakeholder group. We will work with our advisory group to inform the decisions we take throughout the life of the study, and will particularly work with members to develop a publicity and dissemination strategy.

In our main output (the full and final report for the NIHR 'Health Services and Delivery Research' journal) we will provide a clear statement of the implications of what we have found for services and practice, and offer explicit recommendations for future research where knowledge gaps are uncovered. We also anticipate working with the NIHR Dissemination Centre to promote our findings through NIHR Signals, Highlights and Themed Reviews where opportunities allow. As we have done in other NIHR studies on which members of this team have worked (e.g., HS&DR 11/1024/08) we will produce a high-quality accessible summary for publicising online, via social media and in paper form. Our intended audience will include end of life and mental health care managers, practitioners and educators along with members of the public and patients. We will work with our stakeholder advisory group to make sure this document is understandable. Papers reporting main findings will be published in gold open access form (to maximise reach) in relevant world-leading journals, tailored to audiences; candidate titles include those in the BioMed Central series, including BMC Psychiatry, BMC Health Services Research and BMC Palliative Care. This will allow all parties free access to information to facilitate decisions on service organisation, and to support meaningful decisions on future care commissioning and provision. We will also present findings at key stakeholder conferences in the mental health and end of life fields and take opportunities to provide briefings for key stakeholder organisations such as those identified above. We will create an opportunity to directly engage with stakeholders via an end of project dissemination event, which will be modelled on single-day impact events in which members of the team have previously participated (e.g., in HS&DR 11/2004/12). Throughout the study we will use social media (e.g., using a dedicated Twitter account) and a project website to promote wider interest in our work, and make opportunities to engage with the public via regular fora such as Cardiff PublicUni (https://en-gb.facebook.com/PUBlicengagementcardiff/). As part of our final dissemination strategy we will request a blogpost on the Mental Elf Blog (https://www.nationalelfservice.net/mental-health/), and will continue using our networks to share what we find as widely as possible. We will keep these approaches to dissemination under active review, and will continue to be advised by NIHR Dissemination Centre colleagues and our stakeholder advisory group as appropriate.

In all our outputs, where our findings support this we will say how high-quality end of life care for people with severe mental illness should be organised, providing guidance to commissioners, managers and practitioners concerned with improving services and the user and carer experience. We anticipate that the new knowledge we create will have a significant impact on health services organisation and delivery, informing action (e.g., via future NICE guidance and other initiatives) to tackle the problem of disadvantaged dying and promote parity of esteem. We also intend this project to be the starting point for a larger programme of related research in its field, designed over time to produce an evidence base supporting transformations in how care is organised and provided. Building on this project we therefore anticipate future data-generating studies investigating current services (including the size and spread of provision) and testing innovations and interventions.

For peer review only - http://bmjopen.bmj.com/site/about/guidelines.xhtml

HS&DR Project 17/100/15: End of life care for people with severe mental illness: an evidence synthesis (the MENLOC study)

PLAN OF INVESTIGATION AND TIMETABLE

We are able to directly begin work on this project on November 1st 2018 as all members of our team are in place, and as we are not required to secure independent NHS research ethics committee approval, due to the nature of the study.

Our projected timetable is as follows:

For peer review only

For peer review only - http://bmjopen.bmj.com/site/about/guidelines.xhtml

	-3 Aug 18	-2 Sep 18	-1 Oct-18	1 Nov 18	2 Dec 18	3 Jan-19	4 Feb 19	5 Mar 19	6 Apr-19	7 May 19	8 Jun-19	9 Jul-19	10 Aug 19	11 Sep 19	12 Oct-19	13 Nov 19	14 Dec 19	15 Jan-20	+1 Feb 20
Notification of award																			
Project set-up (refine protocol, agree subcontracts, comms)																			
Populate stakeholder advisory group (SAG)																			
Project begins																			
SAG meeting																			
Searching and screening for evidence																			
Quality appraisal and data extraction																			
Data analysis, data synthesis and assessing confidence																			
Summary and writing up																			
End of project event																			
Project ends																			
Report to funder																			

For peer review only

For peer review only - http://bmjopen.bmj.com/site/about/guidelines.xhtml

HS&DR Project 17/100/15: End of life care for people with severe mental illness: an evidence synthesis (the MENLOC study)

PROJECT MANAGEMENT

We are a strong and balanced research team well-placed to complete this project on time and to a high standard. We have expertise in: leading and managing NIHR-funded evidence syntheses to completion (Ben Hannigan and Deborah Edwards); mental health services and research (Ben Hannigan and Michael Coffey); cancer, palliative and end of life services and research (Sally Anstey); organ failure services and research (Paul Gill); systematic reviewing (Mala Mann, Deborah Edwards, Ben Hannigan); and lived experience (Alan Meudell, Roger Pratt). The project manager for this study, Deborah Edwards, is a health services researcher and systematic reviewer with experience of managing complex projects across institutional boundaries. As the researcher with the greatest time attachment to the project her responsibilities will include the day-to-day management of the study. Oversight and overall responsibility for the project will fall to the chief investigator, Ben Hannigan, who is an experienced mental health services researcher and who (with Deborah Edwards and others) has successfully completed two competitively funded systematic reviews in the mental health field in the past (including HS&DR 11/1024/08).

An independently chaired stakeholder advisory group will be populated by representatives drawn from the mental health and end of life fields, and will meet with the project team, in Cardiff, at three strategic time points in the life of the study (see plan of investigation above). As an important part of the work of this group will advising on dissemination, impact and engagement details about membership have been given above (see 'Dissemination and projected outputs'). A first meeting will be scheduled at the commencement of the project, to refine search terms and strategies for the evidence review. A second meeting will take place at the completion of evidence searching and screening. A final meeting will take place at the commencement of the whole-project synthesis and report writing phase, where progress and plans for dissemination and maximising impact will be discussed. The costs attached to this project include those associated with the convening and running of the advisory group.

Using a model successfully used in HS&DR 11/1024/08 members of the project team will initially maintain weekly contact via email, telephone and/or videoconference to ensure that packages of work are distributed according to team members' identified responsibilities, and to ensure that work plans proceed according to agreed schedules. Close monitoring of overall progress against milestones will ensure project completion on time and within budget.

APPROVAL BY ETHICS COMMITTEES

No ethics approval is needed, as this is an evidence synthesis.

PATIENT AND PUBLIC INVOLVEMENT

This proposal has been shaped by people with experience of mental health problems, cancer and other long-term conditions, and by people with experience of caring. The original idea for the study arose following sustained and critical discussions between two co-applicants, Roger Pratt and Sally Anstey, who together identified that individuals with severe mental illness are disadvantaged when diagnoses of advanced cancer or end-stage organ failure are made. Roger is a retired mental health social worker, who lives with lymphoma (in long term remission) and heart failure. He cared for his wife who died from advanced peritoneal cancer; she received specialist palliative care in the last year of her life and died in a hospice. This discussion observed that professional perceptions and misperceptions such as stigma and fear impact on management (e.g., pain control, supporting choice and place of care/death) in the case of people with severe mental illness receiving end of life care. We have since involved Alan Meudell in the project team. Alan is a mental health service user consultant and researcher. He has worked on two NIHR studies and one Health and Care Research Wales (HCRW) study, and leads training for HCRW on involving service users in research. He is also interested in the physical health care of people living with mental health difficulties, and on the provision of equitable services.

The roles played by Roger and Alan will be as equal partners in all stages of the research process except the literature search (which will be led, uniquely, by Mala Mann as the team's information services specialist). Both will participate in initial training which all applicants will join, and will work as part of the team in the critical analysis of the selected articles using CASP (or other appropriate) tools. To support active roles in this activity they will be mentored by other team members as necessary and

For peer review only - http://bmjopen.bmj.com/site/about/guidelines.xhtml

will have access to training facilitated by Health and Care Research Wales. They will also contribute to the task of identifying carers and service users to join our stakeholder group. Critically, both will also be invited to advise on the ongoing focus and direction of the study, and to contribute to the accessible writing up of the final report and other outputs. They will be invited to act as ambassadors in the dissemination of the results and to link with existing patient/service user, support and self-help groups to raise awareness and maximise impact.

In preparing this proposal for submission to the HS&DR Programme we also presented our plans to the Patient Experience and Evaluation in Research (PEER) group at Swansea University (http://www.swansea.ac.uk/humanandhealthsciences/research/patientexperienceandevaluationinresearchpeergroup/). The PEER group comprises people with experience of using health care services and of caring, and exists to provide a public and patient view of research proposals before they are submitted for funding. When our proposal was considered by the PEER group members gave it a very positive response, stating that this was a much-needed project in an area that is largely ignored. People were particularly interested in our plans for choosing members of our stakeholder advisory group, and advised us to engage with charities such as Macmillan, with hospices, and with palliative care staff such as nurses. They also recognised the difficulty of actually recruiting stakeholder advisory group members from the target population, but believed we should at least attempt to engage by offering opportunities for people with severe mental illness and end of life diagnoses to participate in any way feasible for them. We have noted this advice, and have approached services in South Wales to seek their agreement in principle to help put us in touch with service users and carers (as well as managers and practitioners) able to advise our project once funded. We will pursue these connections further once confirmation of support has been received from the HS&DR Board.

Our engagement with patients and the public reflects commitments and experiences demonstrated in other studies on which members of this project team have worked. Examples include: HS&DR 11/1024/08 (the RiSC study, an evidence synthesis into 'risk' for young people in mental health hospital which actively involved young people as stakeholders in shaping the study's progress); HS&DR 11/2004/12 and HS&DR 13/10/75 (COCAPP and COCAPP-A which investigated care planning and care coordination in mental health services, in which service users and carers collaborated as members of lived experiences advisory groups and in which people with experience of mental health difficulties worked as researchers conducting qualitative interviews with service user participants); and Health and Care Research Wales SC-12-03 (Plan4Recovery, which involved people with experience of using mental health services as members of a lived experiences advisory group and as qualitative interviewers).

EXPERTISE AND JUSTIFICATION OF SUPPORT REQUIRED

We are a strong and balanced team. Ben Hannigan (BH) is a mental health services researcher who has led a previous NIHR evidence synthesis (HS&DR 11/1024/08) and who has worked on other NIHR (HS&DR 11/2004/12, HS&DR 13/10/75) and Health and Care Research Wales (SC-12-03) mental health studies. He will be chief investigator, contributing across the evidence synthesis, writing up and dissemination. Michael Coffey (MC) is a mental health services researcher and chief investigator on Health and Care Research Wales social care research grant (SC-12-03), and co-investigator on NIHR HS&DR 11/2004/12 and HS&DR 13/10/75. He will contribute to the evidence synthesis, writing up and dissemination. Paul Gill (PG) is an experienced researcher in the field of chronic conditions, and end-stage organ failure particularly. He will contribute to the evidence synthesis, writing up and dissemination. Mala Mann (MM) is an information specialist with expertise in advanced literature searching and the development of systematic review methodologies. She will lead the database searching. Sally Anstey (SA) is an experienced clinician, educationalist and researcher in the end of life and cancer care fields. She will contribute to the to the evidence synthesis, writing up and dissemination. Deborah Edwards (DE) is an experienced health services researcher who has successfully completed multiple systematic reviews (including for the NIHR) using a variety of approaches. She will project manage this study, and contribute to the evidence synthesis, writing up and dissemination. Roger Pratt (RP) is a retired mental health social worker, and a past member of the Velindre Cancer Centre Patient Liaison Group. He will contribute to the to the evidence synthesis, writing up and dissemination. Alan Meudell (AM) is a mental health service user consultant, researcher and trainer, and past trustee of the charity Mind. He has experience in NIHR projects and in training. He will contribute to the to the evidence synthesis, writing up and dissemination.

For peer review only - http://bmjopen.bmj.com/site/about/guidelines.xhtml

This division of labour and allocation of time is appropriate for a study of this nature. Additional, essential, non-staff costs included are: travel, including to three project/steering group meetings plus refreshments for the same; a sum to cover the costs of interlibrary loans; attendance for Cardiff staff at two conferences, one of which is anticipated to be the International Mental Health Nursing Research Conference; attendance for Swansea staff at one conference; a sum for the planned end of project impact event, to which key stakeholders from the mental health and end of life fields will be invited; a sum for the production and distribution of our accessible summary; and a sum to cover author processing charges for two gold open access publications. Support for these latter costs is requested to maximise dissemination, reach and impact.

REFERENCES

ADDINGTON-HALL, J. 2000. *Positive partnerships: palliative care for adults with severe mental health problems,* London, National Council for Hospice and Specialist Palliative Care Services.
ALL WALES PALLIATIVE CARE PLANNING GROUP 2008. *Report to Minister for Health and Social Services on palliative care services (the Sugar Report),* Cardiff, Welsh Assembly Government.
ALL WALES PALLIATIVE CARE PLANNING GROUP 2009. *Implementation of palliative care report: palliative care services funding 2008 to 2009 (the Finlay Report),* Cardiff, Welsh Assembly Government.
AMERICAN PSYCHIATRIC ASSOCIATION 2014. *Diagnostic and statistical manual of mental disorders (DSM-V),* Arlington, American Psychiatric Publishing.
BATTY, G. D., WHITLEY, E., GALE, C. R., OSBORN, D., TYNELIUS, P. & RASMUSSEN, F. 2012. Impact of mental health problems on case fatality in male cancer patients. *British Journal of Cancer,* 106**,** 1842-1845.
CARE QUALITY COMMISSION 2016a. *A different ending: end of life care review,* Newcastle upon Tyne, Care Quality Commission.
CARE QUALITY COMMISSION 2016b. *A different ending: end of life care review. People with a mental health condition,* Newcastle upon Tyne, Care Quality Commission.
CARNEY, C. P., JONES, L. & WOOLSON, R. F. 2006. Medical comorbidity in women and men with schizophrenia. *Journal of General Internal Medicine,* 21**,** 1133-1137.
CENTRE FOR MENTAL HEALTH 2010. *The economic and social costs of mental health problems in 2009/10,* London, Centre for Mental Health.
CENTRE FOR REVIEWS AND DISSEMINATION 2009. *Systematic reviews: CRD's guidance for undertaking reviews in health care,* York, University of York.
CHESNEY, E., GOODWIN, G. M. & FAZEL, S. 2014. Risks of all-cause and suicide mortality in mental disorders: a meta-review. *World Psychiatry,* 13**,** 153-160.
CHOCHINOV, H. M., MARTENS, P. J., PRIOR, H. J. & KREDENTSER, M. S. 2012. Comparative health care use patterns of people with schizophrenia near the end of life: A population-based study in Manitoba, Canada. *Schizophrenia Research,* 141**,** 241-246.
CRITICAL APPRAISAL SKILLS PROGRAMME (CASP). 2016. *CASP checklists* [Online]. http://www.casp-uk.net/#!checklists/cb36. [Accessed].
DEPARTMENT OF HEALTH 1995. *Building bridges: a guide to arrangements for interagency working for the care and protection of severely mentally ill people,* London, Department of Health.
DEPARTMENT OF HEALTH 2010. *National cancer patient experience survey programme: 2010 national survey report,* London, Department of Health.
DEPARTMENT OF HEALTH 2016. *Our commitment to you for end of life care,* London, Department of Health.
DEPARTMENT OF HEALTH 2017. *A framework for mental health research,* London, Department of Health.
DESAI, M. M., BRUCE, M. L. & KASL, S. V. 1999. The effects of major depression and phobia on stage at diagnosis of breast cancer. *International Journal of Psychiatry in Medicine,* 29**,** 29-45.
DISABILITY RIGHTS COMMISSION 2006. *Equal treatment: closing the gap. A formal investigation into physical health inequalities experienced by people with learning disabilities and/or mental health problems,* London, Disability Rights Commission.
DIXON, J., KING, D., MATOSEVIC, T., CLARK, M. & KNAPP, M. 2015. *Equity in provision of palliative care in the UK,* London, LSE and Marie Curie.
DRAKEFORD, M. 2014. *Palliative care services in Wales are making an enormous difference to people's quality of life,* Speech given in Ty Gobaith hospice, Conwy, http://www.wired-

For peer review only - http://bmjopen.bmj.com/site/about/guidelines.xhtml

HS&DR Project 17/100/15: End of life care for people with severe mental illness: an evidence synthesis (the MENLOC study)

gov.net/wg/news.nsf/articles/Palliative+care+services+in+Wales+are+making+an+enormous+difference+to+peoples+quality+of+life+Mark+Drakeford+28102014162040?open.

EDWARDS, D., NOYES, J., HAF SPENCER, L. & GREGORY, J. W. 2014. An ongoing struggle: a mixed-method systematic review of interventions, barriers and facilitators to achieving optimal self-care by children and young people with type 1 diabetes in educational settings. *BMC Paediatrics,* 14.

ELLISON, N. 2008. *Mental health and palliative care. Literature review,* London, Mental Health Foundation.

EXLEY, C. 2004. Review article: the sociology of dying, death and bereavement. *Sociology of Health and Illness,* 26**,** 110-122.

FOTI, M. E. 2012. Palliative care for patients with serious mental Illness. *In:* CHOCHINOV, H. M. & BREITBART, W. (eds.) *Handbook of psychiatry in palliative medicine.* 2nd ed. Oxford: Oxford University Press.

FRIEDLI, L. & PARSONAGE, M. 2009. *Promoting mental health and preventing mental illness: the economic case for investment in Wales,* Cardiff, All Wales Mental Health Promotion Network.

GLOBAL BURDEN OF DISEASE STUDY 2013 COLLABORATORS 2015. Global, regional, and national incidence, prevalence, and years lived with disability for 301 acute and chronic diseases and injuries in 188 countries, 1990-2013: a systematic analysis for the Global Burden of Disease Study 2013. *The Lancet,* 386**,** 743-800.

GOUGH, D., OLIVER, S. & THOMAS, J. (eds.) 2017. *An introduction to systematic reviews,* London: Sage.

GOUGH, D., THOMAS, J. & OLIVER, S. 2012. Clarifying differences between review designs and methods. *Systematic Reviews,* 1.

GUYATT, G. H., OXMAN, A. D., VIST, G. E., KUNZ, R., FALCK-YTTER, Y., ALONSO-COELLO, P. & SCHÜNEMANN, H. J. 2008. GRADE: an emerging consensus on rating quality of evidence and strength of recommendations. *BMJ,* 336**,** 924-926.

HACKETT, A. & GAITAN, A. 2007. A qualitative study assessing mental health issues in two hospices in the UK. *International Journal of Palliative Care Nursing,* 13**,** 273-281.

HANNIGAN, B., EDWARDS, D., EVANS, N., GILLEN, E., LONGO, M., PRYJMACHUK, S. & TRAINOR, G. 2015. An evidence synthesis of risk identification, assessment and management for young people using tier 4 inpatient child and adolescent mental health services. *Health Services and Delivery Research,* 3.

HOWARD, L. M., BARLEY, E. A., DAVIES, E., RIGG, A., LEMPP, H., ROSE, D. & THORNICROFT, G. 2010. Cancer diagnosis in people with severe mental illness: practical and ethical issues. *The Lancet Oncology,* 11**,** 797-804.

HUANG, H. K., WANG, Y.W., HSIEH, J. G. & HSIEH, C. J. 2018. Disparity of end-of-life care in cancer patients with and without schizophrenia: A nationwide population-based cohort study. *Schizophrenia Research,* 195**,** 434-440.

HUGHES-HALLET, T., CRAFT, A. & DAVIES, C. 2011. *Palliative care funding review: funding the right care and support for everyone,* London, Department of Health.

INDEPENDENT CANCER TASKFORCE. 2015. *Achieving world-class cancer outcomes: a strategy for England 2015-2020* [Online]. http://www.cancerresearchuk.org/sites/default/files/achieving_world-class_cancer_outcomes_-_a_strategy_for_england_2015-2020.pdf. [Accessed].

IRWIN, K. E., HENDERSON, D. C., KNIGHT, H. P. & PIRL, W. F. 2014. Cancer care for individuals with schizophrenia. *Cancer,* 120**,** 323-334

ISHIKAWA, H., YASUNAGA, H., MATSUI, H., FUSHIMI, K. & KAWAKAMI, N. 2016. Differences in cancer stage, treatment and in-hospital mortality between patients with and without schizophrenia: retrospective matched-pair cohort study. *British Journal of Psychiatry,* 208**,** 239-244.

KENNEDY, P., SARAFI, C. & GREENISH, W. 2013. *Homelessness and end of life care*, Marie Curie Cancer Care and St Mungo's.

KNAPIK, G. P. & GRAOR, C. H. 2013. Engaging persons with severe persistent mental illness into primary care. *Journal for Nurse Practitioners,* 9**,** 283-287.

KRONENBERG, C., DORAN, T., GODDARD, M., KENDRICK, T., GILBODY, S., DARE, C. R., AYLOTT, L. & JACOBS, R. 2017. Identifying primary care quality indicators for people with serious mental illness: a systematic review. *British Journal of General Practice,* 67**,** e519-e530.

LAVIN, K., DAVYDOW, D. S., DOWNEY, L., ENGELBERG, R. A., DUNLAP, B., SIBLEY, J., LOBER, W. B., OKIMOTO, K., KHANDELWAL, N., LOGGERS, E. T., TENO, J. M. & CURTIS, J. R. 2017. Effect of psychiatric illness on acute care utilization at end of life from serious medical illness. *Journal of Pain and Symptom Management,* 54**,** 176-185.

For peer review only - http://bmjopen.bmj.com/site/about/guidelines.xhtml

HS&DR Project 17/100/15: End of life care for people with severe mental illness: an evidence synthesis (the MENLOC study)

LEWIN, S., GLENTON, C., MUNTHE-KAAS, H., CARLSEN, B., COLVIN, C. J., GÜLMEZOGLU, M., NOYES, J., BOOTH, A., GARSIDE, R. & RASHIDIAN, A. 2015. Using qualitative evidence in decision making for health and social interventions: an approach to assess confidence in findings from qualitative evidence syntheses (GRADE-CERQual). *PLOS Medicine,* 12**,** e1001895.
MAHOOD, Q., VAN EERD, D. & IRVIN, E. 2014. Searching for grey literature for systematic reviews: challenges and benefits. *Research Synthesis Methods,* 5**,** 221-234.
MARIE CURIE 2015. *Triggers for palliative care. Improving access to care for people with diseases other than cancer: implications for Wales,* London, Marie Curie.
MARIE CURIE AND THE BEVAN FOUNDATION 2014. *Death and dying in Wales,* London and Cardiff, Marie Curie and the Bevan Foundation.
MCCORMACK, P. & SHARP, D. M. M. 2006. Palliative care for people with mental health problems. *European Journal of Palliative Care,,* 13**,** 198-201.
MENTAL HEALTH TASKFORCE 2016. *The five year forward view for mental health*, Mental Health Taskforce.
MILLMAN, J., GALWAY, K., SANTIN, O. & REID, J. 2016. Cancer and serious mental illness – patient, caregiver and professional perspectives: study protocol. *Journal of Advanced Nursing,* 72**,** 217-226.
MOHER, D., LIBERATI, A., TETZLAFF, J., ALTMAN, D. G. & GROUP, T. P. 2009. Preferred reporting items for systematic reviews and meta-analyses: the PRISMA statement. *PLoS Medicine,* 6**,** e1000097.
MORGAN, B. D. 2016. 'No right place to die'. Nursing attitudes and needs in caring for people with serious mental illness at end-of-life. *Journal of the American Psychiatric Nurses Association,* 22**,** 31-42.
NATIONAL INSTITUTE FOR CLINICAL EXCELLENCE 2004. *Improving supportive and palliative care for adults with cancer,* London, National Institute for Clinical Excellence.
NATIONAL INSTITUTE FOR HEALTH AND CARE EXCELLENCE 2009. *Borderline personality disorder: recognition and management,* London, National Institute for Health and Care Excellence.
NATIONAL INSTITUTE FOR HEALTH AND CARE EXCELLENCE 2011. *Service user experience in adult mental health services,* London, National Institute for Health and Care Excellence.
NATIONAL INSTITUTE FOR HEALTH AND CARE EXCELLENCE 2014a. *Bipolar disorder: assessment and management,* London, National Institute for Health and Care Excellence.
NATIONAL INSTITUTE FOR HEALTH AND CARE EXCELLENCE 2014b. *Psychosis and schizophrenia in adults: prevention and management,* London, National Institute for Health and Care Excellence.
NATIONAL INSTITUTE FOR HEALTH AND CARE EXCELLENCE 2018 [anticipated]. *End of life care for adults in the last year of life: service delivery,* London, National Institute for Health and Care Excellence.
NATIONAL INSTITUTE FOR HEALTH RESEARCH 2017. *The Dissemination Centre's upcoming work schedule*, http://www.dc.nihr.ac.uk/about/upcoming-work.htm.
NATIONAL INSTITUTE FOR HEALTH RESEARCH undated. *How to disseminate your research: getting your message heard - and used,* https://www.nihr.ac.uk/funding-and-support/documents/funding-for-research-studies/manage-my-study/How-to-disseminate-your-research/dissemination-guidance.pdf.
NEWTON, J. N., BRIGGS, A. D. M., MURRAY, C. J. L., DICKER, D., FOREMAN, K. J., WANG, H., NAGHAVI, M., FOROUZANFAR, M., OHNO, S. L., BARBER, R. M., VOS, T., STANAWAY, J. D., SCHMIDT, J. C., HUGHES, A. J., FAY, D. F. J., ECOB, R., GRESSER, C., MCKEE, M., RUTTER, H., ABUBAKAR, I., ALI, R., ANDERSON, H. R., BANERJEE, A., BENNETT, D., BERNABÉ, E., BHUI, K. S., BIRYUKOV, S. M., BOURNE, R. R., BRAYNE, C. E. G., BRUCE, N. G., BRUGHA, T. S., BURCH, M., CAPEWELL, S., CASEY, D., CHOWDHURY, R., COATES, M. M., COOPER, C., CRITCHLEY, J. A., DARGAN, P. I., DHERANI, M. K., ELLIOTT, P., EZZATI, M., FENTON, K. A., FRASER, M. S., FÜRST, T., GREAVES, F., GREEN, M. A., GUNNELL, D. J., HANNIGAN, B. M., HAY, R. J., HAY, S. I., HEMINGWAY, H., LARSON, H. J., LOOKER, K. J., LUNEVICIUS, R., LYONS, R. A., MARCENES, W., MASON-JONES, A. J., MATTHEWS, F. E., MOLLER, H., MURDOCH, M. E., NEWTON, C. R., PEARCE, N., PIEL, F. B., POPE, D., RAHIMI, K., RODRIGUEZ, A., SCARBOROUGH, P., SCHUMACHER, A. E., SHIUE, I., SMEETH, L., TEDSTONE, A., VALABHJI, J., WILLIAMS, H. C., WOLFE, C. D. A., WOOLF, A. D. & DAVIS, A. C. J. 2015. Changes in health in England, with analysis by English regions and areas of deprivation, 1990-2013: a systematic analysis for the Global Burden of Disease Study 2013. *The Lancet,* 386**,** 2257-2274.

For peer review only - http://bmjopen.bmj.com/site/about/guidelines.xhtml

NORTHERN IRELAND EXECUTIVE 2010. *Living matters, dying matters. A palliative and end of life care strategy for adults in Northern Ireland,* Belfast, Northern Ireland Executive.
PAYNE, S. 2011. From here to there: reaching out for palliative care. *European Journal of Palliative Care,* 18**,** 264-265.
PICKER INSTITUTE EUROPE 2017. *Wales Cancer Patient Experience Survey 2016: National Report,* Oxford, Picker Institute Europe.
PICOT, S. A., GLAETZER, K. M. & MYHILL, K. J. 2015. Coordinating end of life care for individuals with a mental illness: a nurse practitioner collaboration. *Collegian,* 22**,** 143-149.
PRICE, A., HOTOPF, M., HIGGINSON, I., MONROE, B. & HENDERSON, M. 2006. Psychological services in hospices in the UK and Republic of Ireland. *Journal of the Royal Society of Medicine,* 99**,** 637-639.
PROSPERO: INTERNATIONAL PROSPECTIVE REGISTER OF SYSTEMATIC REVIEWS undated. https://www.crd.york.ac.uk/prospero/.
SCOTTISH GOVERNMENT 2008a. *Better cancer care: an action plan,* Edinburgh, Scottish Government.
SCOTTISH GOVERNMENT 2008b. *Living and dying well: a national action plan for palliative and end of life care in Scotland,* Edinburgh, Scottish Government.
SCOTTISH GOVERNMENT 2009. *Improving the health and wellbeing of people with long term conditions in Scotland: a national action plan,* Edinburgh, Scottish Government.
SHALEV, D., BREWSTER, K., ARBUCKLE, M. R. & LEVENSON, J. A. 2017. A staggered edge: end-of-life care in patients with severe mental illness. *General Hospital Psychiatry,* 44**,** 1-3.
SWEERS, K., DIERCKX DE CASTERLÉ, B., DETRAUX, J. & DE HERT, M. 2013. End-of-life (care) perspectives and expectations of patients with schizophrenia. *Archives of Psychiatric Nursing,* 27**,** 246-252.
TAYLOR, J., SWETENHAM, K., MYHILL, K., PICOT, S., GLAETZER, K. & VAN LOON, A. 2012. IMhPaCT: an education strategy for cross-training palliative care and mental health clinicians. *International Journal of Palliative Nursing,* 18**,** 290-294.
THOMAS, J. & HARDEN, A. 2008. Methods for the thematic synthesis of qualitative research in systematic reviews. *BMC Medical Research Methodology,* 8.
THORNICROFT, G. 2013. Premature death among people with mental illness. *BMJ,* 346.
WARREN, M. B., LAPID, M. I., MCKEAN, A. J., CHA, S. S., STEVENS, M. A., BREKKE, F. M. & BURTON, M. C. 2014. Code status discussions in psychiatric and medical inpatients. *Journal of Clinical Psychiatry,* 76**,** 49-53.
WELSH ASSEMBLY GOVERNMENT 2005. *Designed for life: creating world class health and social care for Wales in the 21st Century,* Cardiff, Welsh Assembly Government.
WELSH GOVERNMENT 2013. *Together for health: delivering end of life care. A delivery plan up to 2016 for NHS Wales and its partners,* Cardiff, Welsh Government.
WOODS, A., WILLISON, K., KINGTON, C. & GAVIN, A. 2008. Palliative care for people with severe persistent mental illness: A review of the literature. *The Canadian Journal of Psychiatry,* 53**,** 725-736.
WORLD HEALTH ORGANIZATION 1992. *The 10th revision of the international classification of diseases and related health problems (ICD-10),* Geneva, World Health Organization.
ZABORA, J. R., LOSCALZO, M. & SMITH, E. 2000. Psychosocial rehabilitation. *In:* ABELOFF, M. D., ARMITAGE, J. O., LICHTER, A. S. & NIEDERHUBER, J. E. (eds.) *Clinical oncology.* New York: Churchill Livingstone.

For peer review only - http://bmjopen.bmj.com/site/about/guidelines.xhtml

Reporting checklist for systematic review and meta-analysis.

Based on the PRISMA guidelines.

Instructions to authors

Complete this checklist by entering the page numbers from your manuscript where readers will find each of the items listed below.

Your article may not currently address all the items on the checklist. Please modify your text to include the missing information. If you are certain that an item does not apply, please write "n/a" and provide a short explanation.

Upload your completed checklist as an extra file when you submit to a journal.

In your methods section, say that you used the PRISMAreporting guidelines, and cite them as:

Moher D, Liberati A, Tetzlaff J, Altman DG, The PRISMA Group. Preferred Reporting Items for Systematic Reviews and Meta-Analyses: The PRISMA Statement

		Reporting Item	Page Number
**Title**			
	#1	Identify the report as a systematic review, meta-analysis, or both.	1
**Abstract**			
Structured summary	#2	Provide a structured summary including, as applicable: background; objectives; data sources; study eligibility criteria, participants, and interventions; study appraisal and synthesis methods; results; limitations; conclusions and implications of key findings; systematic review registration number	2
**Introduction**			
Rationale	#3	Describe the rationale for the review in the context of what is already known.	5

For peer review only

Objectives	#4	Provide an explicit statement of questions being addressed with reference to participants, interventions, comparisons, outcomes, and study design (PICOS).	2
**Methods**			
Protocol and registration	#5	Indicate if a review protocol exists, if and where it can be accessed (e.g., Web address) and, if available, provide registration information including the registration number.	5
Eligibility criteria	#6	Specify study characteristics (e.g., PICOS, length of follow-up) and report characteristics (e.g., years considered, language, publication status) used as criteria for eligibility, giving rational	6
Information sources	#7	Describe all information sources in the search (e.g., databases with dates of coverage, contact with study authors to identify additional studies) and date last searched.	7
Search	#8	Present full electronic search strategy for at least one database, including any limits used, such that it could be repeated.	7
Study selection	#9	State the process for selecting studies (i.e., for screening, for determining eligibility, for inclusion in the systematic review, and, if applicable, for inclusion in the meta-analysis).	8
Data collection process	#10	Describe the method of data extraction from reports (e.g., piloted forms, independently by two reviewers) and any processes for obtaining and confirming data from investigators.	8
Data items	#11	List and define all variables for which data were sought (e.g., PICOS, funding sources), and any assumptions and simplifications made.	6
Risk of bias in individual studies	#12	Describe methods used for assessing risk of bias in individual studies (including specification of whether this was done at the study or outcome level, or both), and how this information is to be used in any data synthesis.	N/A
Summary measures	#13	State the principal summary measures (e.g., risk ratio, difference in means).	N/A

For peer review only

Planned methods of analyis	#14	Describe the methods of handling data and combining results of studies, if done, including measures of consistency (e.g., I2) for each meta-analysis.	8
Risk of bias across studies	#15	Specify any assessment of risk of bias that may affect the cumulative evidence (e.g., publication bias, selective reporting within studies).	N/A
Additional analyses	#16	Describe methods of additional analyses (e.g., sensitivity or subgroup analyses, meta-regression), if done, indicating which were pre-specified.	8
**Results**			
Study selection	#17	Give numbers of studies screened, assessed for eligibility, and included in the review, with reasons for exclusions at each stage, ideally with a flow diagram.	8
Study characteristics	#18	For each study, present characteristics for which data were extracted (e.g., study size, PICOS, follow-up period) and provide the citation.	9
Risk of bias within studies	#19	Present data on risk of bias of each study and, if available, any outcome-level assessment (see Item 12).	N/A
Results of individual studies	#20	For all outcomes considered (benefits and harms), present, for each study: (a) simple summary data for each intervention group and (b) effect estimates and confidence intervals, ideally with a forest plot.	N/A
Synthesis of results	#21	Present the main results of the review. If meta-analyses are done, include for each, confidence intervals and measures of consistency.	9-15
Risk of bias across studies	#22	Present results of any assessment of risk of bias across studies (see Item 15).	N/A
Additional analysis	#23	Give results of additional analyses, if done (e.g., sensitivity or subgroup analyses, meta-regression [see Item 16]).	10
**Discussion**			
Summary of Evidence	#24	Summarize the main findings, including the strength of evidence for each main outcome; consider their relevance to	15-16

For peer review only

		key groups (e.g., health care providers, users, and policy makers	
Limitations	#25	Discuss limitations at study and outcome level (e.g., risk of bias), and at review level (e.g., incomplete retrieval of identified research, reporting bias).	16
Conclusions	#26	Provide a general interpretation of the results in the context of other evidence, and implications for future research.	17
**Funding**			
Funding	#27	Describe sources of funding or other support (e.g., supply of data) for the systematic review; role of funders for the systematic review.	17

The PRISMA checklist is distributed under the terms of the Creative Commons Attribution License CC-BY. This checklist was completed on 26. April 2021 using https://www.goodreports.org/, a tool made by the EQUATOR Network in collaboration with Penelope.ai

For peer review only

Database search strategies

Database: AMED (Allied and Complementary Medicine)

Search Strategy:

----

1 exp Palliative care/ (6092)

2 exp Hospice care/ (2100)

3 exp Terminal Care/ (5265)

4 ("palliative care" or hospice or "end of life care" or end-of-life).tw. (13126)

5 ((hospice or terminal*) adj3 (care or caring or ill*)).tw. (7843)

6 ("Irreversible condition" or "terminal condition" or fatal illness).tw. (27)

7 ("last year of life" or LYOL or "end of life" or "end of their lives" or "last six months of life" or "last 6 months of life").tw. (3749)

8 (end-stage disease* or end stage disease* or end-stage ill* or end stage ill* or end-stage or end stage).tw. (539)

9 (expected adj3 die).tw. (15)

10 (imminent adj3 death).tw. (44)

11 ("Dying soon" or "expected death" or "imminently dying" or Moribund).tw. (40)

12 (conservative adj2 (treatment or management)).tw. (1010)

13 Withholding Treatment/ (66)

14 Treatment Refusal/ (134)

15 (Refus* adj3 (treat* or care or intervention or dialysis)).tw. (272)

16 ((withdrew or withdraw* or withhold*) adj3 (treat* or car* or intervene* or therap* or dialysis or transplant*)).tw. (363)

17 or/1-16 (16993)

18 exp Neoplasms/ (14717)

19 (cancer* or tumo?r* or neoplas* or malignan* or carcinoma* or adenocarcinoma* or adeno?carcinoma* or choriocrcinoma* or leukemia* or leukaemia* or metastat* or sarcoma* or teratoma* or lymphoma).tw. (19919)

20 enzyme disorder.tw. (1)

21 Hereditary disease/ (147)

For peer review only

22 ("duchenne muscular dystrophy" or "amyotrophic lateral sclerosis" or ALS).tw. (1007)

23 amyotrophic lateral sclerosis/ (223)

24 Muscular dystrophy, Duchenne/ (70)

25 ("Genetic disease" or "genetic condition").tw. (18)

26 Cystic Fibrosis/ (451)

27 ("cystis fibrosis" or CF).tw. (314)

28 "Multi* Organ Failure".tw. (20)

29 ("Organ failure" or "chronic organ failure").tw. (49)

30 Pulmonary Disease, Chronic Obstructive/ (449)

31 ("renal insufficiency" or "serious physical illness").tw. (27)

32 (Chronic obstructive pulmonary disease or COPD).tw. (1665)

33 (chronic adj3 (illness or condition or disease*)).tw. (9365)

34 "chronic medical condition".tw. (10)

35 Heart Failure/ (394)

36 (chronic adj2 ("cardiac failure" or "liver failure" or "kidney failure" or "end-stage renal disease" or ESRD or "renal failure" or "heart failure")).tw. (439)

37 Chronic Disease/ (6305)

38 Health service utilization.mp. (25)

39 lifestyle-related factor*.mp. (3)

40 or/18-39 (31309)

41 exp Bipolar Disorder/ (81)

42 exp Schizophrenia/ (931)

43 (schizo* or "mood disorder*" or "personality disorder*" or psychotic* or psychosis or psychoses).tw. (2312)

44 (bipolar or mania or Schizophrenia).tw. (1447)

45 (Depression adj2 (psychosis or psychotic or severe or major)).tw. (390)

46 exp Mental Disorders/ (27114)

47 mental health condition*.tw. (43)

48 exp Personality Disorders/ (323)

49 exp psychotic disorders/ (424)

50 ("severe mental illness" or "persistent mental illness").tw. (362)

For peer review only

51 ((chronic* or sever* or serious or persistent* or enduring or debilitating) adj2 (mental* or psychological*) adj2 (ill* or disorder* or health)).tw. (936)

52 (SPMI or SMI).tw. (79)

53 or/41-52 (27932)

54 (dementia or Alzheimer).ti. (1706)

55 (bipolar electrocoagulation or bipolar radiofrequency or bipolar tumour probe or bipolar diathermy).tw. (6)

56 ("respiratory depression" or "marrow depression" or "hematologic* depression").tw. (42)

57 (child* or adoles* or pediatric or paediatric).tw. (27044)

58 (Algeria$ or Egypt$ or Liby$ or Morocc$ or Tunisia$ or Western Sahara$ or Angola$ or Benin or Botswana$ or Burkina Faso or Burundi or Cameroon or Cape Verde or Central African Republic or Chad or Comoros or Congo or Djibouti or Eritrea or Ethiopia$ or Gabon or Gambia$ or Ghana or Guinea or Keny$ or Lesotho or Liberia or Madagasca$ or Malawi or Mali or Mauritania or Mauritius or Mayotte or Mozambiq$ or Namibia$ or Niger or Nigeria$ or Reunion or Rwand$ or Saint Helena or Senegal or Seychelles or Sierra Leone or Somalia or South Africa$ or Sudan or Swaziland or Tanzania or Togo or Ugand$ or Zambia$ or Zimbabw$ or China or Chinese or Hong Kong or Macao or Mongolia$ or Taiwan$ or Belarus or Moldov$ or Russia$ or Ukraine or Afghanistan or Armenia$ or Azerbaijan or Bahrain or Cyprus or Cypriot or Georgia$ or Iran$ or Iraq$ or Jordan$ or Kazakhstan or Kuwait or Kyrgyzstan or Leban$ or Oman or Pakistan$ or Palestin$ or Qatar or Saudi Arabia or Syria$ or Tajikistan or Turkmenistan or United Arab Emirates or Uzbekistan or Yemen or Bangladesh$ or Bhutan or British Indian Ocean Territory or Brunei Darussalam or Cambodia$ or India$ or Indonesia$ or Lao or People's Democratic Republic or Malaysia$ or Maldives or Myanmar or Nepal or Philippin$ or Singapore or Sri Lanka or Thai$ or Timor Leste or Vietnam or Albania$ or Andorra or Bosnia$ or Herzegovina$ or Bulgaria$ or Croatia$ or Faroe Islands or Greenland or Liechtenstein or Lithuani$ or Macedonia or Malta or maltese or Romania or Serbia$ or Montenegro or Svalbard or Argentina$ or Belize or Bolivia$ or Brazil$ or Chilean or Colombia$ or Costa Rica$ or Cuba or Ecuador or El Salvador or French Guiana or Guatemala$ or Guyana or Haiti or Honduras or Jamaica$ or Nicaragua$ or Panama or Paraguay or Peru or Puerto Rico or Suriname or Uruguay or Venezuela or developing countr$ or south America$).ti,sh. (9674)

59 or/54-58 (37507)

60 17 and 40 and 53 (230)

61 60 not 59 (193)

Proquest ASSIA

(("Dying soon" or "expected death" or "imminently dying" or Moribund) OR ("end-stage disease*" or "end stage disease*" or "end-stage ill*" or "end stage ill*" or end-stage or “end stage”) OR ("last year of life" or LYOL or "end of life" or "end of their lives" or "last six months of life" or "last 6 months of life") OR ("Irreversible condition" or "terminal condition" or "fatal illness") OR ("palliative care" or hospice or "end of life care" or end-of-life or "Terminal care" or "terminal* ill*")) AND ((Neoplasm* or cancer* or tumor* or tumour or neoplas* or malignan* or carcinoma* or

For peer review only

adenocarcinoma* or choriocrcinoma* or leukemia* or leukaemia* or metastat* or sarcoma* or teratoma* or lymphoma) OR ("duchenne muscular dystrophy" or "amyotrophic lateral sclerosis" or ALS) OR ("Genetic disease" or "genetic condition" or "cystis fibrosis" or CF) OR ("Organ failure" or "chronic organ failure" or "mulitple organ failure") OR (Chronic obstructive pulmonary disease or COPD or "renal insufficiency" or "serious physical illness" or "chronic medical condition") OR (chronic NEAR/3 ("cardiac failure" or "liver failure" or "kidney failure" or "end-stage renal disease" or ESRD or "renal failure" or "heart failure")) OR (chronic NEAR/3 (illness or condition or disease*))) AND ((Bipolar or schizo* or "mood disorder*" or "personality disorder*" or psychotic* or psychosis or psychoses) OR (Depression NEAR/2 (psychosis or psychotic or severe or major)) OR ("psychotic disorders" or "schizoaffective disorder" or "severe and persistent mental illness") OR ("psychotic disorders" or "schizoaffective disorder" or "Paranoid Disorders") OR ("severe mental illness" or "persistent mental illness" or SPMI or SMI))

CINAHL Search History

Select / deselect all

Search ID#	Search Terms	Search Options	Actions
S46	S15 AND S32 AND S45	Limiters - English Language; Age Groups: All Adult<br><br>Search modes - Boolean/Phrase	View Results (356) View Details Edit
S45	S33 OR S34 OR S35 OR S36 OR S37 OR S38 OR S39 OR S40 OR S41 OR S42 OR S43 OR S44	Search modes - Boolean/Phrase	View Results (124,935) View Details Edit
S44	AB SPMI or SMI	Search modes - Boolean/Phrase	View Results (1,211) View Details Edit
S43	AB (chronic* or sever* or serious or persistent* or enduring or debilitating) AND AB (mental* or psychological*) AND AB (ill* or disorder* or health)	Search modes - Boolean/Phrase	View Results (31,921) View Details Edit
S42	AB (chronic* or sever* or serious or persistent* or enduring or debilitating) AND AB (mental* or	Search modes - Boolean/Phrase	View Results (0)

For peer review only

		psychological*) adj2 (ill* or disorder* or health)		View Details Edit
☐	S41	AB (bipolar or mania or Schizophrenia) OR AB (psychotic* or psychosis or psychoses) OR AB (schizo* or "mood disorder*" or "personality disorder*") OR AB "mental health condition*" OR AB "severe mental illness" OR AB "persistent mental illness"	Search modes - Boolean/Phrase	View Results (38,951) View Details Edit
☐	S40	(TX "Mentally Ill person*")	Search modes - Boolean/Phrase	View Results (698) View Details Edit
☐	S39	(MH "Paranoid Disorders")	Search modes - Boolean/Phrase	View Results (617) View Details Edit
☐	S38	(MH "Psychotic Disorders")	Search modes - Boolean/Phrase	View Results (9,609) View Details Edit
☐	S37	(MH "Schizoaffective Disorder")	Search modes - Boolean/Phrase	View Results (223) View Details Edit
☐	S36	(MH "Personality Disorders")	Search modes - Boolean/Phrase	View Results (3,809) View Details Edit
☐	S35	(MH "Mental Disorders")	Search modes - Boolean/Phrase	View Results (48,711) View Details Edit
☐	S34	(MH "Schizophrenia")	Search modes - Boolean/Phrase	View Results (21,058) View Details

For peer review only

☐	S33	(MH "Bipolar Disorder")	Search modes - Boolean/Phrase	Edit View Results (9,957) View Details
☐	S32	S16 OR S17 OR S18 OR S19 OR S20 OR S21 OR S22 OR S23 OR S24 OR S25 OR S26 OR S27 OR S28 OR S29 OR S30 OR S31	Search modes - Boolean/Phrase	Edit View Results (444,331) View Details
☐	S31	TX "lifestyle-related factor*"	Search modes - Boolean/Phrase	Edit View Results (215) View Details
☐	S30	(MH "Chronic Disease")	Search modes - Boolean/Phrase	Edit View Results (53,902) View Details
☐	S29	AB chronic N2 "cardiac failure" OR AB chronic N2 "liver failure" OR AB chronic N2 "kidney failure" OR AB chronic N2 "end-stage renal disease" OR AB chronic N2 ESRD OR AB chronic N2 "renal failure" OR AB chronic N2 "heart failure"	Search modes - Boolean/Phrase	Edit View Results (4,975) View Details
☐	S28	(MH "Liver Failure")	Search modes - Boolean/Phrase	Edit View Results (1,681) View Details
☐	S27	(MH "Renal Insufficiency")	Search modes - Boolean/Phrase	Edit View Results (5,723) View Details
☐	S26	(MH "Heart Failure")	Search modes - Boolean/Phrase	Edit View Results (34,187) View Details
☐	S25	(MH "Pulmonary Disease, Chronic Obstructive") OR	Search modes -	Edit View

For peer review only

		(MH "Lung Diseases, Obstructive") OR (MH "Chronic Disease")	Boolean/Phrase	Results (72,636) View Details Edit
☐	S24	(MH "Multiple Organ Dysfunction Syndrome")	Search modes - Boolean/Phrase	View Results (2,454) View Details Edit
☐	S23	Multiple Organ Failure	Search modes - Boolean/Phrase	View Results (0) View Details Edit
☐	S22	(MH "Cystic Fibrosis")	Search modes - Boolean/Phrase	View Results (6,385) View Details Edit
☐	S21	(MH "Muscular Dystrophy, Duchenne")	Search modes - Boolean/Phrase	View Results (1,331) View Details Edit
☐	S20	(MH "Amyotrophic Lateral Sclerosis")	Search modes - Boolean/Phrase	View Results (3,299) View Details Edit
☐	S19	AB ("renal insufficiency" or "serious physical illness") OR AB (Chronic obstructive pulmonary disease or COPD) OR AB "chronic medical condition"	Search modes - Boolean/Phrase	View Results (14,172) View Details Edit
☐	S18	AB ("Enzymatic disease*" or "enzyme disease*") OR AB ("duchenne muscular dystrophy" or "amyotrophic lateral sclerosis" or ALS) OR AB ("Genetic disease" or "genetic condition") OR AB ("cystis fibrosis" or CF) OR AB ("Organ failure" or "chronic organ failure")	Search modes - Boolean/Phrase	View Results (12,244) View Details Edit
☐	S17	AB cancer* or tumo?r* or neoplas* or malignan* or carcinoma* or adenocarcinoma* or adeno?carcinoma* or choriocrcinoma* or leukemia* or leukaemia* or metastat* or sarcoma* or teratoma* or lymphoma	Search modes - Boolean/Phrase	View Results (274,177) View

For peer review only

				Details<br>Edit
☐	S16	(MH "Neoplasms")	Search modes - Boolean/Phrase	View Results (67,564)<br>View Details<br>Edit
☐	S15	S1 OR S2 OR S3 OR S4 OR S5 OR S6 OR S7 OR S8 OR S9 OR S10 OR S11 OR S12 OR S13 OR S14	Search modes - Boolean/Phrase	View Results (159,654)<br>View Details<br>Edit
☐	S14	AB withdraw* N3 treat* OR AB withdraw* N3 car* OR AB withdraw* N3 intervene* OR AB withdraw* N3 therap* OR AB withdraw* N3 dialysis OR AB withdraw* N3 transplant*	Search modes - Boolean/Phrase	View Results (2,671)<br>View Details<br>Edit
☐	S13	AB withhold* N3 treat* OR AB withhold* N3 car* OR AB withhold* N3 intervene* OR AB withhold* N3 therap* OR AB withhold* N3 dialysis OR AB withhold* N3 transplant*	Search modes - Boolean/Phrase	View Results (561)<br>View Details<br>Edit
☐	S12	AB withdrew N3 treat* OR AB withdrew N3 car* OR AB withdrew N3 intervene* OR AB withdrew N3 dialysis OR AB withdrew N3 transplant* or AB withdrew N3 therap*	Search modes - Boolean/Phrase	View Results (185)<br>View Details<br>Edit
☐	S11	TX Refus* N3 treat* OR TX Refus* N3 care OR TX Refus* N3 intervention OR TX Refus* N3	Search modes - Boolean/Phrase	View Results (13,309)<br>View Details<br>Edit
☐	S10	TX conservative N2 treatment OR TX conservative N2 management OR TX "Withholding Treatment" OR TX "Treatment Refusal"	Search modes - Boolean/Phrase	View Results (20,188)<br>View Details<br>Edit
☐	S9	TX "Dying soon" or "expected death" or "imminently dying" or Moribund	Search modes - Boolean/Phrase	View Results (1,442)<br>View Details<br>Edit

For peer review only

☐	S8	TX ("end-stage disease*" or end stage disease* or end-stage ill* or "end stage ill*" or end-stage or "end stage") OR AB expected N3 die OR AB imminent N3 death	Search modes - Boolean/Phrase	View Results (23,677) View Details Edit
☐	S7	TX ("Irreversible condition" or "terminal condition" or "fatal illness") OR TX ("last year of life" or LYOL or "end of life" or "end of their lives") or TX ("last six months of life" or "last 6 months of life")	Search modes - Boolean/Phrase	View Results (37,619) View Details Edit
☐	S6	AB terminal* N3 care OR AB terminal* N3 caring OR AB terminal* N3 ill*	Search modes - Boolean/Phrase	View Results (3,888) View Details Edit
☐	S5	AB hospice N3 care OR AB hospice N3 caring OR AB hospice N3 ill*	Search modes - Boolean/Phrase	View Results (3,428) View Details Edit
☐	S4	TX "palliative care" or hospice or "end of life care" or end-of-life	Search modes - Boolean/Phrase	View Results (104,201) View Details Edit
☐	S3	(MH "Terminal Care") OR (MH "Terminally Ill Patients")	Search modes - Boolean/Phrase	View Results (23,777) View Details Edit
☐	S2	(MH "Hospice Care")	Search modes - Boolean/Phrase	View Results (7,894) View Details Edit
☐	S1	(MH "Palliative Care")	Search modes - Boolean/Phrase	

Search Name: COCHRANE Menloc final

Last Saved: 13/12/2018 09:55:54

Comment: after editing

For peer review only

ID	Search
#1	MeSH descriptor: [Palliative Care] explode all trees
#2	MeSH descriptor: [Hospice Care] explode all trees
#3	MeSH descriptor: [Terminal Care] this term only
#4	MeSH descriptor: [Terminally Ill] this term only
#5	"palliative care" or hospice or "end of life care" or end-of-life
#6	"Irreversible condition" or "terminal condition" or "fatal illness"
#7	"last year of life" or LYOL or "end of life" or "end of their lives" or "last six months of life" or "last 6 months of life"
#8	end-stage disease* or end stage disease* or end-stage ill* or end stage ill* or end-stage or "end stage"
#9	imminent NEAR/3 death
#10	expected NEAR/3 die
#11	MeSH descriptor: [Conservative Treatment] this term only
#12	conservative NEAR/2 management
#13	conservative NEAR/2 treatment
#14	MeSH descriptor: [Withholding Treatment] explode all trees
#15	MeSH descriptor: [Treatment Refusal] explode all trees
#16	withdrew NEAR/3 treat*
#17	Withdrew NEAR/3 car*
#18	Withdrew NEAR/3 intervene*
#19	Withdrew NEAR/3 therap*
#20	Withdrew NEAR/3 dialysis
#21	#1 or #2 #3 or #4 or #5 or #6 or #7 or #8 or #9 or #10 or #11 or #12 or #13 or #14 or #15 or #16 or #17 or #18 or #19 or 20
#22	cancer* or tumo?r* or neoplas* or malignan* or carcinoma* or adenocarcinoma* or adeno?carcinoma* or choriocrcinoma* or leukemia* or leukaemia* or metastat* or sarcoma* or teratoma* or lymphoma
#23	MeSH descriptor: [Neoplasms] explode all trees
#24	MeSH descriptor: [Amyotrophic Lateral Sclerosis] explode all trees
#25	MeSH descriptor: [Genetic Diseases, Inborn] explode all trees
#26	MeSH descriptor: [Muscular Dystrophy, Duchenne] explode all trees

For peer review only

#27 MeSH descriptor: [Cystic Fibrosis] explode all trees

#28 MeSH descriptor: [Multiple Organ Failure] explode all trees

#29 MeSH descriptor: [Pulmonary Disease, Chronic Obstructive] explode all trees

#30 MeSH descriptor: [Heart Failure] explode all trees

#31 MeSH descriptor: [Renal Insufficiency] explode all trees

#32 MeSH descriptor: [Liver Failure] explode all trees

#33 MeSH descriptor: [Chronic Disease] explode all trees

#34 "duchenne muscular dystrophy" or "amyotrophic lateral sclerosis" or ALS

#35 "Genetic disease" or "genetic condition"

#36 chronic NEAR/2 ESRD

#37 "chronic illness" or "chronic condition" or "chronic disease*"

#38 "chronic medical condition"

#39 #22 or #23 or #24 or #25 or #26 or #27 or #28 or #29 or #30 or #31 or #32 or #33 or #34 or #35 or #36 or #37 or #38

#40 "severe mental illness" or "persistent mental illness"

#41 schizo* or "mood disorder*" or "personality disorder*" or psychotic* or psychosis or psychoses or SPMI or SMI

#42 bipolar or mania or Schizophrenia or psychosis

#43 Depression NEAR/2 psychosis

#44 Depression NEAR/2 psychotic

#45 Depression NEAR/2 severe

#46 Bipolar or "Mental Disorders" or "Personality Disorders"

#47 "psychotic disorders" or "schizoaffective disorder"

#48 "severe and persistent mental illness".

#49 #40 or #41 or #42 or #43 or #44 or #45 or #46 or #47 or #48

#50 #21 and #39 and #49

For peer review only

DARE 1998-2018

DARE and NHS EED archives secure on CRD website until at least 2021

("palliative care" or hospice or "end of life care" or end-of-life) and "severe mental illness" or " persistent mental illness".

Database: EMBASE <1947-Present>

Search Strategy:

----

1 exp palliative therapy/ (102069)

2 exp hospice care/ (9228)

3 exp terminal care/ (63131)

4 exp terminally ill patient/ (8148)

5 ("palliative care" or hospice or "end of life care" or end-of-life).tw. (65572)

6 ((hospice or terminal*) adj3 (care or caring or ill*)).tw. (17540)

7 ("Irreversible condition" or "terminal condition" or fatal illness).tw. (1173)

8 ("last year of life" or LYOL or "end of life" or "end of their lives" or "last six months of life" or "last 6 months of life").tw. (28590)

9 (end-stage disease* or end stage disease* or end-stage ill* or end stage ill* or end-stage or end stage).tw. (88414)

10 (expected adj3 die).tw. (306)

11 (imminent adj3 death).tw. (870)

12 ("Dying soon" or "expected death" or "imminently dying" or Moribund).tw. (3793)

13 conservative treatment/ (78244)

14 (conservative adj2 (treatment or management)).tw. (64421)

15 treatment withdrawal/ (17413)

16 treatment refusal/ (17138)

17 (Refus* adj3 (treat* or care or intervention or dialysis)).tw. (7265)

18 ((withdrew or withdraw* or withhold*) adj3 (treat* or car* or intervene* or therap* or dialysis or transplant*)).tw. (23578)

For peer review only

19 or/1-18 (420975)

20 exp neoplasm/ (4447791)

21 (cancer* or tumo?r* or neoplas* or malignan* or carcinoma* or adenocarcinoma* or adeno?carcinoma* or choriocrcinoma* or leukemia* or leukaemia* or metastat* or sarcoma* or teratoma* or lymphoma).tw. (4510708)

22 ("Enzymatic disease*" or "enzyme disease*").tw. (45)

23 genetic disorder/ (55815)

24 ("duchenne muscular dystrophy" or "amyotrophic lateral sclerosis" or ALS).tw. (55394)

25 amyotrophic lateral sclerosis/ (34903)

26 Duchenne muscular dystrophy/ (14824)

27 ("Genetic disease" or "genetic condition").tw. (10518)

28 cystic fibrosis/ (68731)

29 ("cystis fibrosis" or CF).tw. (62550)

30 exp multiple organ failure/ (35190)

31 ("Organ failure" or "chronic organ failure").tw. (27179)

32 chronic obstructive lung disease/ (115273)

33 ("renal insufficiency" or "serious physical illness").tw. (32224)

34 (Chronic obstructive pulmonary disease or COPD).tw. (93906)

35 (chronic adj3 (illness or condition or disease*)).tw. (374959)

36 "chronic medical condition".tw. (563)

37 exp kidney failure/ (343470)

38 exp heart failure/ (474239)

39 exp liver failure/ (70302)

40 (chronic adj2 ("cardiac failure" or "liver failure" or "kidney failure" or "end-stage renal disease" or ESRD or "renal failure" or "heart failure")).tw. (67182)

41 exp chronic disease/ (187803)

42 lifestyle-related factor*.mp. (445)

43 or/20-42 (6880626)

44 exp bipolar disorder/ (58962)

45 exp schizophrenia/ (184860)

46 (schizo* or "mood disorder*" or "personality disorder*" or psychotic* or psychosis or psychoses).tw. (279001)

For peer review only

47 (bipolar or mania or Schizophrenia).tw. (216783)

48 (Depression adj2 (psychosis or psychotic or severe or major)).tw. (39961)

49 exp mental disease/ (2123169)

50 mental health condition*.tw. (2502)

51 exp personality disorder/ (60988)

52 exp psychosis/ (290561)

53 exp schizoaffective psychosis/ (9378)

54 exp paranoid psychosis/ (18067)

55 "severe mental illness".tw. (4748)

56 " persistent mental illness".tw. (514)

57 ((chronic* or sever* or serious or persistent* or enduring or debilitating) adj2 (mental* or psychological*) adj2 (ill* or disorder* or health)).mp. (16645)

58 mental patient/ (27553)

59 (SPMI or SMI).tw. (5581)

60 or/44-59 (2207382)

61 (dementia or Alzheimer).ti. (70245)

62 (bipolar electrocoagulation or bipolar radiofrequency or bipolar tumour probe or bipolar diathermy).tw. (1641)

63 ("respiratory depression" or "marrow depression" or "hematologic* depression").tw. (10545)

64 (child* or adoles* or pediatric or paediatric).tw. (2085814)

65 (Algeria$ or Egypt$ or Liby$ or Morocc$ or Tunisia$ or Western Sahara$ or Angola$ or Benin or Botswana$ or Burkina Faso or Burundi or Cameroon or Cape Verde or Central African Republic or Chad or Comoros or Congo or Djibouti or Eritrea or Ethiopia$ or Gabon or Gambia$ or Ghana or Guinea or Keny$ or Lesotho or Liberia or Madagasca$ or Malawi or Mali or Mauritania or Mauritius or Mayotte or Mozambiq$ or Namibia$ or Niger or Nigeria$ or Reunion or Rwand$ or Saint Helena or Senegal or Seychelles or Sierra Leone or Somalia or South Africa$ or Sudan or Swaziland or Tanzania or Togo or Ugand$ or Zambia$ or Zimbabw$ or China or Chinese or Hong Kong or Macao or Mongolia$ or Taiwan$ or Belarus or Moldov$ or Russia$ or Ukraine or Afghanistan or Armenia$ or Azerbaijan or Bahrain or Cyprus or Cypriot or Georgia$ or Iran$ or Iraq$ or Jordan$ or Kazakhstan or Kuwait or Kyrgyzstan or Leban$ or Oman or Pakistan$ or Palestin$ or Qatar or Saudi Arabia or Syria$ or Tajikistan or Turkmenistan or United Arab Emirates or Uzbekistan or Yemen or Bangladesh$ or Bhutan or British Indian Ocean Territory or Brunei Darussalam or Cambodia$ or India$ or Indonesia$ or Lao or People's Democratic Republic or Malaysia$ or Maldives or Myanmar or Nepal or Philippin$ or Singapore or Sri Lanka or Thai$ or Timor Leste or Vietnam or Albania$ or Andorra or Bosnia$ or Herzegovina$ or Bulgaria$ or Croatia$ or Faroe Islands or Greenland or Liechtenstein or Lithuani$ or Macedonia or Malta or maltese or Romania or Serbia$ or Montenegro or Svalbard or Argentina$ or Belize or Bolivia$ or Brazil$ or Chilean or Colombia$ or Costa Rica$ or Cuba or Ecuador or El Salvador or French Guiana or Guatemala$ or Guyana or Haiti or Honduras or Jamaica$ or Nicaragua$ or

For peer review only

Panama or Paraguay or Peru or Puerto Rico or Suriname or Uruguay or Venezuela or developing countr$ or south America$).ti,sh. (1653512)

66 scientific literature/ (30246)

67 thesis.tw. (11296)

68 book.pt. (1180)

69 book/ (41676)

70 or/61-69 (3683087)

71 19 and 43 and 60 (13644)

72 71 not 70 (11727)

73 limit 72 to (human and english language and (adult <18 to 64 years> or aged <65+ years>)) (5427)

Database: Ovid MEDLINE(R) ALL <1946 to December 06, 2018>

Search Strategy:

----

1 exp Palliative care/ (49655)

2 exp Hospice care/ (5949)

3 exp Terminal Care/ (48040)

4 exp Terminally ill/ (6227)

5 ("palliative care" or hospice or "end of life care" or end-of-life).tw. (43896)

6 ((hospice or terminal*) adj3 (care or caring or ill*)).tw. (12766)

7 ("Irreversible condition" or "terminal condition" or fatal illness).tw. (791)

8 ("last year of life" or LYOL or "end of life" or "end of their lives" or "last six months of life" or "last 6 months of life").tw. (19924)

9 (end-stage disease* or end stage disease* or end-stage ill* or end stage ill* or end-stage or end stage).tw. (60633)

10 (expected adj3 die).tw. (215)

11 (imminent adj3 death).tw. (561)

12 ("Dying soon" or "expected death" or "imminently dying" or Moribund).tw. (2493)

13 conservative treatment/ (1401)

For peer review only

14 (conservative adj2 (treatment or management)).tw. (42831)

15 Withholding Treatment/ (10909)

16 Treatment Refusal/ (11477)

17 (Refus* adj3 (treat* or care or intervention or dialysis)).tw. (4855)

18 ((withdrew or withdraw* or withhold*) adj3 (treat* or car* or intervene* or therap* or dialysis or transplant*)).tw. (15455)

19 or/1-18 (244771)

20 exp Neoplasms/ (3107101)

21 (cancer* or tumo?r* or neoplas* or malignan* or carcinoma* or adenocarcinoma* or adeno?carcinoma* or choriocrcinoma* or leukemia* or leukaemia* or metastat* or sarcoma* or teratoma* or lymphoma).tw. (3243976)

22 ("Enzymatic disease*" or "enzyme disease*").tw. (25)

23 Genetic Disease, Inborn/ (13207)

24 ("duchenne muscular dystrophy" or "amyotrophic lateral sclerosis" or ALS).tw. (40207)

25 amyotrophic lateral sclerosis/ (16921)

26 Muscular dystrophy, Duchenne/ (4706)

27 ("Genetic disease" or "genetic condition").tw. (7305)

28 Cystic Fibrosis/ (33150)

29 ("cystis fibrosis" or CF).tw. (36968)

30 exp Multiple Organ Failure/ (10209)

31 ("Organ failure" or "chronic organ failure").tw. (17137)

32 Pulmonary Disease, Chronic Obstructive/ (34379)

33 ("renal insufficiency" or "serious physical illness").tw. (21134)

34 (Chronic obstructive pulmonary disease or COPD).tw. (54881)

35 (chronic adj3 (illness or condition or disease*)).tw. (250692)

36 "chronic medical condition".tw. (382)

37 Heart Failure/ (107446)

38 Renal Insufficiency/ (14803)

39 Liver Failure/ (6828)

40 (chronic adj2 ("cardiac failure" or "liver failure" or "kidney failure" or "end-stage renal disease" or ESRD or "renal failure" or "heart failure")).tw. (44143)

41 Chronic Disease/ (251517)

For peer review only

42 Health service utilization.mp. (1569)

43 lifestyle-related factor*.mp. (354)

44 or/20-43 (4717135)

45 exp Bipolar Disorder/ (37764)

46 exp Schizophrenia/ (99011)

47 (schizo* or "mood disorder*" or "personality disorder*" or psychotic* or psychosis or psychoses).tw. (197049)

48 (bipolar or mania or Schizophrenia).tw. (151754)

49 (Depression adj2 (psychosis or psychotic or severe or major)).tw. (28840)

50 exp Mental Disorders/ (1143274)

51 mental health condition*.tw. (1954)

52 exp Personality Disorders/ (39433)

53 exp psychotic disorders/ (48805)

54 exp schizoaffective disorder/ (48805)

55 exp psychosis/ (48805)

56 Paranoid Disorders/ (3987)

57 ("severe mental illness" or "persistent mental illness").tw. (3969)

58 ((chronic* or sever* or serious or persistent* or enduring or debilitating) adj2 (mental* or psychological*) adj2 (ill* or disorder* or health)).tw. (12556)

59 exp Mentally Ill Persons/ (5926)

60 (SPMI or SMI).tw. (3830)

61 or/45-60 (1239759)

62 (dementia or Alzheimer).ti. (52380)

63 (bipolar electrocoagulation or bipolar radiofrequency or bipolar tumour probe or bipolar diathermy).tw. (1123)

64 ("respiratory depression" or "marrow depression" or "hematologic* depression").tw. (6399)

65 (child* or adoles* or pediatric or paediatric).tw. (1520041)

66 (Algeria$ or Egypt$ or Liby$ or Morocc$ or Tunisia$ or Western Sahara$ or Angola$ or Benin or Botswana$ or Burkina Faso or Burundi or Cameroon or Cape Verde or Central African Republic or Chad or Comoros or Congo or Djibouti or Eritrea or Ethiopia$ or Gabon or Gambia$ or Ghana or Guinea or Keny$ or Lesotho or Liberia or Madagasca$ or Malawi or Mali or Mauritania or Mauritius or Mayotte or Mozambiq$ or Namibia$ or Niger or Nigeria$ or Reunion or Rwand$ or Saint Helena or Senegal or Seychelles or Sierra Leone or Somalia or South Africa$ or Sudan or Swaziland or Tanzania or Togo or Ugand$ or Zambia$ or Zimbabw$ or China or Chinese or Hong Kong or Macao or

For peer review only

Mongolia$ or Taiwan$ or Belarus or Moldov$ or Russia$ or Ukraine or Afghanistan or Armenia$ or Azerbaijan or Bahrain or Cyprus or Cypriot or Georgia$ or Iran$ or Iraq$ or Jordan$ or Kazakhstan or Kuwait or Kyrgyzstan or Leban$ or Oman or Pakistan$ or Palestin$ or Qatar or Saudi Arabia or Syria$ or Tajikistan or Turkmenistan or United Arab Emirates or Uzbekistan or Yemen or Bangladesh$ or Bhutan or British Indian Ocean Territory or Brunei Darussalam or Cambodia$ or India$ or Indonesia$ or Lao or People's Democratic Republic or Malaysia$ or Maldives or Myanmar or Nepal or Philippin$ or Singapore or Sri Lanka or Thai$ or Timor Leste or Vietnam or Albania$ or Andorra or Bosnia$ or Herzegovina$ or Bulgaria$ or Croatia$ or Faroe Islands or Greenland or Liechtenstein or Lithuani$ or Macedonia or Malta or maltese or Romania or Serbia$ or Montenegro or Svalbard or Argentina$ or Belize or Bolivia$ or Brazil$ or Chilean or Colombia$ or Costa Rica$ or Cuba or Ecuador or El Salvador or French Guiana or Guatemala$ or Guyana or Haiti or Honduras or Jamaica$ or Nicaragua$ or Panama or Paraguay or Peru or Puerto Rico or Suriname or Uruguay or Venezuela or developing countr$ or south America$).ti,sh. (1225437)

67 Academic Dissertations/ (0)

68 thesis.tw. (7167)

69 book.pt. (0)

70 Books/ (3174)

71 or/62-70 (2653676)

72 19 and 44 and 61 (2434)

73 72 not 71 (2126)

74 limit 73 to (english language and humans and "all adult (19 plus years)") (1217)

Database: PsycINFO <1806 to December Week 1 2018>

Search Strategy:

----

1 exp Palliative Care/ (11016)

2 Hospice/ (3062)

3 exp Terminally Ill Patients/ (4562)

4 exp HOSPICE/ (3062)

5 ("palliative care" or "hospice care" or "end of life care" or end-of-life).tw. (15589)

6 ((hospice or terminal*) adj3 (care or caring or ill*)).tw. (6333)

7 ("Irreversible condition" or "terminal condition" or fatal illness).tw. (216)

8 ("last year of life" or LYOL or "end of life" or "end of their lives" or "last six months of life" or "last 6 months of life").tw. (9038)

For peer review only

9 (end-stage disease* or end stage disease* or end-stage ill* or end stage ill* or end-stage or end stage).tw. (2002)

10 (expected adj3 die).tw. (44)

11 (imminent adj3 death).tw. (272)

12 ("Dying soon" or "expected death" or "imminently dying" or Moribund).tw. (235)

13 (conservative adj2 (treatment or management)).tw. (460)

14 Treatment Withholding/ (462)

15 Treatment Refusal/ (730)

16 (Refus* adj3 (treat* or care or intervention or dialysis)).tw. (1811)

17 ((withdrew or withdraw* or withhold*) adj3 (treat* or car* or intervene* or therap* or dialysis or transplant*)).tw. (3579)

18 or/1-17 (28620)

19 exp NEOPLASMS/ (47829)

20 (cancer* or tumo?r* or neoplas* or malignan* or carcinoma* or adenocarcinoma* or adeno?carcinoma* or choriocrcinoma* or leukemia* or leukaemia* or metastat* or sarcoma* or teratoma* or lymphoma).tw. (76722)

21 ("Enzymatic disease*" or "enzyme disease*").tw. (0)

22 Genetic Disorders/ (4048)

23 ("duchenne muscular dystrophy" or "amyotrophic lateral sclerosis" or ALS).tw. (13477)

24 Amyotrophic Lateral Sclerosis/ (3551)

25 Muscular Dystrophy/ (1293)

26 ("Genetic disease" or "genetic condition").tw. (611)

27 Cystic Fibrosis/ (854)

28 ("cystis fibrosis" or CF).tw. (3217)

29 "Multi* Organ Failure*".tw. (71)

30 ("Organ failure" or "chronic organ failure").tw. (203)

31 exp Chronic Obstructive Pulmonary Disease/ (1272)

32 ("renal insufficiency" or "serious physical illness").tw. (319)

33 (Chronic obstructive pulmonary disease or COPD).tw. (2277)

34 (chronic adj3 (illness or condition or disease*)).tw. (28394)

35 "chronic medical condition".tw. (196)

36 Heart Disorders/ (8989)

For peer review only

37 Kidney Diseases/ (2001)

38 Liver Disorders/ (1145)

39 (chronic adj2 ("cardiac failure" or "liver failure" or "kidney failure" or "end-stage renal disease" or ESRD or "renal failure" or "heart failure")).tw. (866)

40 Chronic Illness/ (10804)

41 lifestyle-related factor*.mp. (51)

42 or/19-41 (139563)

43 exp Bipolar Disorder/ (25413)

44 exp SCHIZOPHRENIA/ (85758)

45 (schizo* or "mood disorder*" or "personality disorder*" or psychotic* or psychosis or psychoses).tw. (208100)

46 (bipolar or mania or Schizophrenia).tw. (137089)

47 (Depression adj2 (psychosis or psychotic or severe or major)).tw. (34922)

48 exp Mental Disorders/ (566262)

49 mental health condition*.tw. (1698)

50 exp Personality Disorders/ (33638)

51 exp Schizoaffective Disorder/ (2961)

52 exp Psychosis/ (109879)

53 "Paranoia (Psychosis)"/ (1215)

54 "Severity (Disorders)"/ (15885)

55 "severe mental illness".tw. (4681)

56 "persistent mental illness".tw. (799)

57 ((chronic* or sever* or serious or persistent* or enduring or debilitating) adj2 (mental* or psychological*) adj2 (ill* or disorder* or health)).tw. (16725)

58 Mentally Ill Persons.mp. (805)

59 (SPMI or SMI).tw. (1991)

60 or/43-59 (637058)

61 (dementia or Alzheimer).ti. (30336)

62 (bipolar electrocoagulation or bipolar radiofrequency or bipolar tumour probe or bipolar diathermy).tw. (5)

63 ("respiratory depression" or "marrow depression" or "hematologic* depression").tw. (407)

64 (child* or adoles* or pediatric or paediatric).tw. (811926)

For peer review only

65 (Algeria$ or Egypt$ or Liby$ or Morocc$ or Tunisia$ or Western Sahara$ or Angola$ or Benin or Botswana$ or Burkina Faso or Burundi or Cameroon or Cape Verde or Central African Republic or Chad or Comoros or Congo or Djibouti or Eritrea or Ethiopia$ or Gabon or Gambia$ or Ghana or Guinea or Keny$ or Lesotho or Liberia or Madagasca$ or Malawi or Mali or Mauritania or Mauritius or Mayotte or Mozambiq$ or Namibia$ or Niger or Nigeria$ or Reunion or Rwand$ or Saint Helena or Senegal or Seychelles or Sierra Leone or Somalia or South Africa$ or Sudan or Swaziland or Tanzania or Togo or Ugand$ or Zambia$ or Zimbabw$ or China or Chinese or Hong Kong or Macao or Mongolia$ or Taiwan$ or Belarus or Moldov$ or Russia$ or Ukraine or Afghanistan or Armenia$ or Azerbaijan or Bahrain or Cyprus or Cypriot or Georgia$ or Iran$ or Iraq$ or Jordan$ or Kazakhstan or Kuwait or Kyrgyzstan or Leban$ or Oman or Pakistan$ or Palestin$ or Qatar or Saudi Arabia or Syria$ or Tajikistan or Turkmenistan or United Arab Emirates or Uzbekistan or Yemen or Bangladesh$ or Bhutan or British Indian Ocean Territory or Brunei Darussalam or Cambodia$ or India$ or Indonesia$ or Lao or People's Democratic Republic or Malaysia$ or Maldives or Myanmar or Nepal or Philippin$ or Singapore or Sri Lanka or Thai$ or Timor Leste or Vietnam or Albania$ or Andorra or Bosnia$ or Herzegovina$ or Bulgaria$ or Croatia$ or Faroe Islands or Greenland or Liechtenstein or Lithuani$ or Macedonia or Malta or maltese or Romania or Serbia$ or Montenegro or Svalbard or Argentina$ or Belize or Bolivia$ or Brazil$ or Chilean or Colombia$ or Costa Rica$ or Cuba or Ecuador or El Salvador or French Guiana or Guatemala$ or Guyana or Haiti or Honduras or Jamaica$ or Nicaragua$ or Panama or Paraguay or Peru or Puerto Rico or Suriname or Uruguay or Venezuela or developing countr$ or south America$).ti,sh. (128851)

66 thesis.tw. (24537)

67 book.pt. (476469)

68 exp BOOKS/ (6110)

69 or/61-68 (1332511)

70 18 and 42 and 60 (838)

71 70 not 69 (609)

# 11	105	#10 AND #7 AND #6<br>Indexes=SCI-EXPANDED, SSCI, CPCI-S, CPCI-SSH<br>Timespan=All years
# 10	394,440	#9 OR #8<br>Indexes=SCI-EXPANDED, SSCI, CPCI-S, CPCI-SSH<br>Timespan=All years
# 9	388,426	TS=(Bipolar or schizo* or "mood disorder*" or "personality disorder*" or psychotic* or psychosis or psychoses) OR TS=("psychotic disorders" or "schizoaffective disorder" or "severe and persistent mental illness")<br>Indexes=SCI-EXPANDED, SSCI, CPCI-S, CPCI-SSH<br>Timespan=All years
# 8	22,785	**TOPIC:** ("schizoaffective disorder" or "severe and persistent mental illness" OR "psychotic disorders" or "schizoaffective disorder" or "Paranoid Disorders" OR "severe mental illness" or "persistent mental illness" or SPMI or SMI)

For peer review only

		Indexes=SCI-EXPANDED, SSCI, CPCI-S, CPCI-SSH Timespan=All years
# 7	4,104,078	**TOPIC:** (Neoplasm* or cancer* or tumor* or tumour or neoplas* or malignan* or carcinoma* or adenocarcinoma* or choriocrcinoma* or leukemia* or leukaemia* or metastat* or sarcoma* or teratoma* or lymphoma) *OR* **TOPIC:** ("duchenne muscular dystrophy" or "amyotrophic lateral sclerosis" or ALS) *OR* **TOPIC:** ("Genetic disease" or "genetic condition" or "cystis fibrosis" or CF) *OR* **TOPIC:** ("Organ failure" or "chronic organ failure" or "mulitple organ failure") *OR* **TOPIC:** (Chronic obstructive pulmonary disease or COPD or "renal insufficiency" or "serious physical illness" or "chronic medical condition") Indexes=SCI-EXPANDED, SSCI, CPCI-S, CPCI-SSH Timespan=All years
# 6	109,890	#5 OR #4 OR #3 OR #2 OR #1 Indexes=SCI-EXPANDED, SSCI, CPCI-S, CPCI-SSH Timespan=All years
# 5	2,468	**TOPIC:** ("Dying soon" or "expected death" or "imminently dying" or Moribund) Indexes=SCI-EXPANDED, SSCI, CPCI-S, CPCI-SSH Timespan=All years
# 4	56,678	**TOPIC:** ("end-stage disease*" or "end stage disease*" or "end-stage ill*" or "end stage ill*" or end-stage or “end stage”) Indexes=SCI-EXPANDED, SSCI, CPCI-S, CPCI-SSH Timespan=All years
# 3	24,428	**TOPIC:** ("last year of life" or LYOL or "end of life" or "end of their lives" or "last six months of life" or "last 6 months of life") Indexes=SCI-EXPANDED, SSCI, CPCI-S, CPCI-SSH Timespan=All years
# 2	773	**TOPIC:** ("Irreversible condition" or "terminal condition" or "fatal illness") Indexes=SCI-EXPANDED, SSCI, CPCI-S, CPCI-SSH Timespan=All years
# 1	54,014	**TOPIC:** ("palliative care" or hospice or "end of life care" or end-of-life or "terminal care") Indexes=SCI-EXPANDED, SSCI, A&HCI, CPCI-S, CPCI-SSH, ESCI Timespan=All years

Other sources

Trial Registers	Search terms
ClinicalTrials.gov	"end of life" or palliative and mental
metaRegister of Controlled Trials (mRCT)	Not Applicable
UK Clinical Trials Gateway (UKCTG)	for "last year of life" or LYOL or "end of life" or "end of their lives" in mental
WHO ICTRP Search Portal International	"end of life" or "end of their lives" and

For peer review only - http://bmjopen.bmj.com/site/about/guidelines.xhtml

	mental

Charity websites	Search terms
Cancer Research UK	**"end of life" and "mental illness"**
Cancer Research Wales	"end of life" and "mental illness"
Tenovus Cancer Care	"end of life" and "mental illness"
Mental Health Foundation	Reports identified browsing publications page
Centre for Mental Health	Searched publications – Mental health Policy https://www.centreformentalhealth.org.uk/publications
National Kidney Foundation	**"end of life" and "mental illness"**
British Liver Trust	**"end of life" and "mental illness"**
British Renal Society	Scanning BRS conference abstract page
The Renal Association	**"end of life" and "mental illness"**
British Heart Foundation	**“last year of life" or LYOL or "end of life" or "end of their lives" and “severe mental” and Healthcare professionals**
British Lung Foundation	**"end of life" and "mental illness"**
National Council for Palliative Care	"end of life care" and mental
Hospice UK	"end of life care" and mental
Marie Curie	"end of life care" and "mental illness" and evaluation
Macmillan Cancer Support	**“last year of life" or LYOL or "end of life" or "end of their lives" and “severe mental”**
Scottish Partnership agency for Palliative Care	"end of life care" and "mental illness" and evaluation
Bipolar UK	"end of life care" and "mental illness" and evaluation
MIND	"end of life care" and "mental illness"
Rethink Mental Illness	"end of life care" and "mental illness"
Sova	"end of life care" and mental
Mental Health UK	No search facility or publications page
Heads Together	No search facility or publications page

Organisation Websites	Search terms
NHS England	"end of life care" and "mental illness" and evaluation
NHS Wales	"end of life care" and "severe mental” and evaluation
Care Inspectorate Wales	"end of life care" and "severe mental”
English CQC	**"end of life" and “severe mental” in Publications**
Ministry of Justice	"end of life care" and "mental illness" and evaluation

Journals	Search terms

Journal of Pain and Symptom Management	**“end of life care”** in *Title/Abs/Keywords* OR **"palliative care"** in *Title/Abs/Keywords* AND **Mental** in *Title/Abs/Keywords*<br>2017-2019
Cancer	“end of life” and mental or palliative and mental 2017-2019
Psycho-Oncology	“end of life” and mental (2017-2019) or palliative and mental (2017-2018)
BMJ Supportive & Palliative Care	""mental illness"" and published between "01 Jan, 2017 and 21 Feb, 2019""mental" and published between "01 Jan, 2017 and 21 Feb, 2019

For peer review only

Supplementary searches

Trial Registers	Search terms
ClinicalTrials.gov	"end of life" or palliative and mental
metaRegister of Controlled Trials (mRCT)	Not Applicable
UK Clinical Trials Gateway (UKCTG)	"last year of life" or LYOL or "end of life" or "end of their lives" in mental
WHO ICTRP Search Portal International<br>https://www.who.int/ictrp/en/	"end of life" or "end of their lives" and mental

Charity websites	Search type	Search terms
1. Cancer Research UK<br>http://www.cancerresearchuk.org/	Electronic search: Main website	"end of life" and "mental illness/health"<br>"palliative care" and mental illness/health"
2. Cancer Research Wales<br>http://www.cancerresearchuk.org/	Electronic search: Main website	"end of life" and "mental illness/health"<br>"palliative care" and mental illness/health"
3. Tenovus Cancer Care<br>http://www.tenovuscancercare.org.uk/	Electronic search: Main website	"end of life" and "mental illness"
4. Mental Health Foundation<br>https://www.mentalhealth.org.uk/	Electronic search: Main website<br><br>Manual search: Policy publications	"end of life" and "mental illness/health"<br>"palliative care" and mental illness/health"<br>As per reviews inclusion criteria
5. Centre for Mental Health<br>https://www.centreformentalhealth.org.uk/	Electronic search: Policy publications	"end of life" and "mental illness/health"

For peer review only

		"palliative care" and mental illness/health"
6. National Kidney Foundation https://www.kidney.org/	Electronic search: Main website<br><br>Manual search: Policy publications	"end of life" and "mental illness/health"<br>"palliative care" and mental illness/health"<br>As per reviews inclusion criteria
7. British Liver Trust https://www.britishlivertrust.org.uk/	Electronic search: Publications	"end of life" and "mental illness"<br>"palliative care" and mental illness"<br>(mental health too broad a term)
8. British Renal Society http://britishrenal.org/	Electronic search: Conference abstracts	"end of life" and "mental illness/health"<br>"palliative care" and mental illness/health"
9. The Renal Association http://britishrenal.org/	Electronic search: Main website	"end of life" and "mental illness/health"<br>"palliative care" and mental illness/health"
10. British Heart Foundation http://britishrenal.org/	Electronic search: Main website	"last year of life" or LYOL or "end of life" or "end of their lives" and "severe mental" and Healthcare professionals<br>"palliative care" and mental illness"<br>(mental health too broad)
11. British Lung Foundation https://www.blf.org.uk/	Electronic search: Main website	"end of life" and "mental illness/health"<br>"palliative care" and mental illness/health"
12. Hospice UK http://www.hospiceuk.org/	Electronic search: Main website	"end of life" and "mental illness/health"<br>"palliative care" and mental illness/health"
13. Marie Curie https://www.mariecurie.org.uk/	Electronic search: Main website	"end of life" and "mental illness/health"<br>"palliative care" and mental illness/health"

For peer review only

	Manual search: Briefings	As per reviews inclusion criteria
14. Macmillan Cancer Support https://www.macmillan.org.uk/	Electronic search: Main website	"last year of life" or LYOL or "end of life" or "end of their lives" and "severe mental" "palliative care" and mental illness/health"
15. Bipolar UK https://www.bipolaruk.org/	Electronic search: Main website	"end of life" and "mental illness/health" Palliative
16. MIND https://www.mind.org.uk/	Electronic search: Main website	"end of life care" and "mental illness/health" Palliative
17. Rethink Mental Illness https://www.rethink.org/	Electronic search: Main website	"end of life care" and "mental illness/health" Palliative
18. Sova https://www.sova.org.uk/	Electronic search: Main website	"end of life care" and mental Palliative
19. Hafal: for recovery for serious mental illness www.hafal.org	Electronic search: Main website	"end of life care" and mental Palliative and mental
20. Age Concern https://www.ageuk.org.uk	Electronic search: Main website	"end of life care " and mental Palliative and mental
21. Salvation Army https://salvationarmy.org.uk	Electronic search: Main website	"end of life care" and mental Palliative and mental
22. Dying Matters https://www.dyingmatters.org/	Electronic search: Main website	"end of life " and mental "palliative care" and mental illness/health"
23. Care Not Killing https://www.carenotkilling.org.uk/	Electronic search: Main website	"end of life care" and then searched within results for the term mental

For peer review only

		"palliative care" and then searched within results for the term mental
24. Northern Ireland Hospice https://www.nihospice.org/	Electronic search: Main website	"end of life care" and mental<br>Palliative
25. Mental Health UK https://www.mentalhealth-uk.org/	No search facility or publications page	
26. Heads Together https://www.headstogether.org.uk/	No search facility or publications page	
27. Kidney Research UK https://www.kidneyresearchuk.org/	Electronic search: Main website	"end of life care" and mental<br>Palliative
28. National Kidney Federation https://www.kidney.org.uk/	Electronic search: Main website	"end of life care" and mental<br>Palliative
29. St Mungos https://www.mungos.org/	Electronic search: Main website	"end of life "<br>Palliative<br>“mental illness”
30. Samaritans https://www.samaritans.org/	Electronic search: Main website	"end of life" and “severe mental”<br>Palliative and “severe mental”
31. Llanmau https://www.llamau.org.uk/	Electronic search: Main website	
32. SSAFA https://www.ssafa.org.uk/	Electronic search: Main website	
33. Community Housing Cymru: https://chcymru.org.uk/	Electronic search: Main website	
34. National Housing Federation: https://www.housing.org.uk/	Electronic search: Main website	
35. Shelter Cymru https://sheltercymru.org.uk/	Electronic search: Main website	

For peer review only

36. Shelter https://www.shelter.org.uk/	Electronic search: Main website	
37. Gofal http://www.gofal.org.uk/	No search function checked projects & services page but no relevant reports.	As per reviews inclusion criteria
38. Compassionate communities https://www.compassionate-communitiesuk.co.uk/	No search function checked projects but no relevant reports	As per reviews inclusion criteria
39. Byw Nawr https://www.dyingmatters.org/wales	Electronic search: Main website	
40. Combat Stress https://www.combatstress.org.uk/	Electronic search: Main website	
41. Royal British Legion https://www.britishlegion.org.uk/	Electronic search: Main website	

Organisation Websites	Search type	Search terms
1. NHS England https://www.england.nhs.uk/cancer/strategy/ https://www.england.nhs.uk/eolc/resources/ - http://endoflifecareambitions.org.uk/	Electronic search: Main website	"mental illness"
2. NHS Wales http://www.wales.nhs.uk/	Electronic search: Main website<br>Advanced search: title field only	"end of life" and "mental illness/health"<br>"palliative care" and mental illness/health"
3. Welsh Government https://gov.wales/	Electronic search: Main website<br>Advanced search<br>Limited to Health and social services	"end of life" and "mental illness/health"<br>"palliative care" and mental illness/health"
4. Department of Health and Social Care https://www.gov.uk/	Electronic search: Publications<br>Limited by:	"end of life" and "mental illness/health"<br>"palliative care" and mental illness/health"

For peer review only

	Department of Health and Social Care<br>Topic: Health and Social Care<br>Sub topic: End of Life	
5. Department of Health: Northern Ireland		"end of life" and "mental illness/health"<br>"palliative care" and mental illness/health"
6. Department of Health, Social Services and Public Safety: Northern Ireland		"end of life" and "mental illness/health"<br>"palliative care" and mental illness/health"
7. Ministry of Justice<br>https://www.gov.uk/	Electronic search: Publications<br>Limited by: Ministry of Justice<br>Topic: Health and Social Care<br>Sub Topic: End of Life	"end of life" and "mental illness/health"<br>"palliative care" and mental illness/health"
8. Care Inspectorate Wales<br>https://www.qcs.co.uk/wales/	Searching only available with practitioner registration<br>Emailed – no relevant publications	
9. Care Quality Commission<br>https://www.cqc.org.uk/	Electronic search: Publications	"end of life" and "severe mental"<br>"palliative care" and "mental illness"
10. Prisons and Probation Ombudsman<br>https://www.ppo.gov.uk	Electronic search: Main website<br>Manual search: Fatal incident reports over a one year period between June 2017-18.<br>Limited by: Deaths of natural causes	"end of life"<br>As per reviews inclusion criteria
11. Royal College of Psychiatrists<br>https://www.rcpsych.ac.uk/	Manual search: College Reports	As per reviews inclusion criteria
12. Royal College of Physicians<br>https://www.rcplondon.ac.uk/	Electronic search: Main website	"end of life" and "mental illness/health"<br>"palliative care" and mental illness/health"
13. The Worldwide Hospice and Palliative Care Alliance	Manual search: Resources	As per reviews inclusion criteria

For peer review only

http://www.thewhpca.org/		
14. European Association of Palliative Care https://www.eapcnet.eu/	Manual search: National Guidelines (UK Countries only)	As per reviews inclusion criteria
15. Scottish Partnership agency for Palliative Care https://www.palliativecarescotland.org.uk/	Electronic search: Main website	"end of life care" and "mental illness" and evaluation<br>"palliative care" and mental illness/health"
16. National Council for Palliative Care http://www.ncpc.org.uk/	Electronic search: Main website	"end of life" and "mental illness/health"<br>"palliative care" and mental illness/health""
17. Social Care Institute for Excellence https://www.scie.org.uk/	Electronic search: Main website	"end of life" and "mental illness"<br>"palliative care" and mental illness"
18. Health Improvement Scotland http://www.healthcareimprovementscotland.org/ Covers publications by NHS Scotland, Scottish Government and Health Improvement Scotland	Electronic search: Main website	"end of life" and "mental illness"<br>"palliative care" and mental illness"
19. National Institute of Clinical Excellence. https://www.nice.org.uk/guidance	Electronic search: Main website	"end of life" or "palliative care"<br>"mental illness"
20. Royal College of Nursing https://www.rcn.org.uk/clinical-topics/end-of-life-care	Manual search through section on Clinical Topics End of Life	As per reviews inclusion criteria
21. Gold Standards Framework http://www.goldstandardsframework.org.uk/	Manual search through library resources	As per reviews inclusion criteria

For peer review only

Hand searching journals	Search terms
Journal of Pain and Symptom Management	"end of life care" in *Title/Abs/Keywords* OR "palliative care" in *Title/Abs/Keywords* AND Mental in *Title/Abs/Keywords* 2017-2019
Cancer	"end of life" and mental or palliative and mental 2017-2019
Psycho-Oncology	"end of life" and mental (2017-2019) or palliative and mental (2017-2018)
BMJ Supportive & Palliative Care	""mental illness"" and published between "01 Jan, 2017 and 21 Feb, 2019""mental" and published between "01 Jan, 2017 and 21 Feb, 2019

Google search using the terms "palliative care" and "mental illness" (searching first 10 pages of output)

Output	Action	
Naylor et al 2016. Bringing together physical and mental health: A new frontier for integrated care. Available from: https://www.kingsfund.org.uk/publications/physical-and-mental-health	Searched and added to table	Relevant report
Help the Hospices. 2016 Hospice and palliative care – Access for All. Available from: http://nican.hscni.net/files/hospice_and_palliative_care_access_for_all.pdf Accessed 31 January 2019	Searched and added to table	Relevant report
Ulster University Repository<br>Walsh, S. Sheridan, A. Leavey, G. Coughlan, B. Frazer, K. O'Toole, S. Kemple, M. Crawley, L. (2014) Identifying and addressing the palliative care needs of people with serious mental illness in Ireland. Irish College of General Practitioners, 31(7). p.1<br><br>Sheridan, A. Couglan, B. Frazer, K. Walsh, S. Bergin, J. A. Crawley, L. Kemple, M. O'Toole, S. (n.d.)The palliative care needs of people with serious mental illness in Ireland. http://www.professionalpalliativehub.com/sites/default/files/Paliative%20Care%20need%20of%20People%20with%20SMI%20June%2015%202017%20Report%20Draft.pdf	Screened<br><br>Email sent to author 20-02-2019 via researchers gate for date of publication	Relevant paper

For peer review only

NHS Lothian. 2010. Living and dying well in Lothian. Available from: https://www.nhslothian.scot.nhs.uk/OurOrganisation/Strategies/ladwinlothian/Documents/Palliative%20Care%20Strategy%202010%20-15%20VER%2023%20FINAL.pdf	Searched and added to table	Relevant Report
alex.mathew@wales.nhs.uk. End of life care for older people with schizophrenia and bipolar affective disorder along with long term physical conditions: a phenomenological enquiry into the experiences of bereaved family carers, mental health and social care providers in South Wales-UK	Email sent 20th Feb 2019	NOT RELEVANT
Queens University. Belfast Repository Millman, J. Galway, K. Santin, O. Reid J. (2016) Cancer and serious mental illness – patient, caregiver and professional perspectives: study protocol. Journal of Advanced Nursing 72(1), 217–226	Author search conducted on PubMed and full study findings not yet published	NOT RELEVANT
Bloomer, M. J. O'Brien, A. P. (2013) Palliative care for the person with a serious mental illness: The need for a partnership approach to care in Australia, Progress in Palliative Care, 21:1, 27-31	Screened	Not relevant

Google search using the terms "end of life" and "mental illness" (searching first 10 pages of output)

Output	Action	
Blog post Paul Gionfriddo and Nathaniel Counts "Providing end-of-life support for elders with serious mental illnesses" Health Affairs Blog, November 16, 2017.DOI: 10.1377/hblog20171114.475106	Screened	1. Not Relevant
Doering, P. End of life care and serious persistent mental illness. Regional Palliative Care Services. June 2018, Issue 42, p. 1-3 https://www.northernhealth.ca/sites/northern_health/files/health-professionals/palliative-care/documents/serious-persistent-mental-illness-issue-42.pdf	Screened	2. Not relevant
Ahearn 2015. The loneliness of mental illness at the end of life.	Screened	3. Not relevant

For peer review only

https://www.wisconsinmedicalsociety.org/_WMS/publications/wmj/pdf/117/3/101.pdf		
WA Cancer and Palliative Care Network 2018. WA End-of-Life and Palliative Care Strategy 2018–2028. Available from: https://ww2.health.wa.gov.au/~/media/Files/Corporate/general%20documents/Health%20Networks/Palliative%20care/WA%20End-of-life%20and%20Palliative%20Care%20Strategy%202018-2028.pdf	Screened	Not relevant
National Association of Social workers 2004. NASW Standards for Palliative & End of Life Care. Available from: https://www.socialworkers.org/LinkClick.aspx?fileticket=xBMd58VwEhk%3D&portalid=0	Screened	Not relevant
NSW Ministry of Health 2015. Dignity, Respect and Choice: Advance Care Planning for End of Life for People with Mental Illness - A Comprehensive Guide. Available at: https://www.health.nsw.gov.au/patients/acp/Pages/comprehensive-guide.aspx. NSW Government	Screened	Not relevant

Google search using the terms using the terms “end of life” and schizophrenia (searching first 5 pages of output)

Output	Action	
Steves and Williams 2016<br>Enhancing end-of-life care for terminally ill psychiatric patients<br>Nursing, 46(8): 54-8	screened	8. Relevant

Google search using the terms “end of life” and bipolar (searching first 5 pages of output)

Output	Action	
Maloney, K. Opinion: My husband refused to believe he had bipolar disorder, and denial destroyed him. May 15th 2014 https://www.thejournal.ie/readme/bipolar-ii-disorder-denial-mental-health-1467162-May2014/	Screened	Relevant

For peer review only

Journal articles and reports suggested by stakeholder advisory members

Output	Action	
Kurella, M. Kimmel, P. L. Young, B.S. Chertow, G. M.<br>Suicide in the United States end-stage renal disease program. Journal of the American Society of Nephrology. 2005: 16(3): 774-81<br>https://jasn.asnjournals.org/content/16/3/774.full	Screened	1. Not relevant
Liu, C-H. Yeh, M-K. Weng, S-C. Bai, M-Y. Chang, J-C.<br>Suicide and chronic kidney disease: a case-control study. Nephrology Dialysis Transplantation. 2017: 2(9): 1524–29	Screened	2. Not relevant
Prison and Probation Ombudsman for England and Wales 2013<br>Learning from PPO Investigations. End of Life Care	Searched and added to table	3. Not relevant
Prison and Probation Ombudsman for England and Wales 2019<br>Fatal Incident Reports.<br>https://www.ppo.gov.uk/document/fii-report/	Searched through 2 years' worth of reports where death was from natural causes 4th February 2019	4. UNSURE
All Wales Palliative Care Planning Group 2008<br>Report to Minister for Health and Social services on Palliative care Services – Sugar Report<br>www,wales.nhs.uk/documents/palliativecareport.pdf	Searched and added to table	5. Not relevant
Secretariat of the Commission of the Bishops' Conferences of the European Community 2016<br>Opinion of the Working Group on Ethics in Research and Medicine On Palliative Care in the European Union<br>http://www.comece.eu/dl/psqlJKJKooNNJqx4KJK/PalliativeCARE_EN.pdf	Searched and added to table	6. Not relevant
Addicott, R. Ashton, R. 2010<br>Delivering Better Care at the End of Life: The next steps. The King's Fund	Searched and added to table	7. Not relevant

https://www.kingsfund.org.uk/sites/default/files/Delivering-better-care-end-of-life-Kings-Fund-January-2010-Leeds-Castle-EOLC.pdf		
Addicott, R. Hiley, J. 2011 Issues Facing Commissioners in End-of-Life Care. London: The King's Fund https://www.kingsfund.org.uk/sites/default/files/issues-facing-commissioners-end-of-life-care-report-september2011.pdf	Searched and added to table	8. Not relevant
Addicott R, Ross S. 2010 Implementing the End of Life Care Strategy. Lessons for Good Practice. The Kings Fund https://www.kingsfund.org.uk/sites/default/files/field/field_publication_file/Implementing-end-of-life-care-Rachael-Addicott-Shilpa-Ross-Kings-Fund-October2010_0.pdf	Searched and added to table	9. Not relevant
General Medical Council 2010 Treatment and Care Towards the End of Life: Good Practice in Decision Making https://www.gmc-uk.org/-/media/documents/treatment-and-care-towards-the-end-of-life---english-1015_pdf-48902105.pdf	Searched and added to table	10. Not relevant
Hall, S. Petkova, H. Tsouros, A. D. Costantini, M. Higginson, I. G. 2011 Palliative Care for Older People: Better Practices. Copenhagen WHO: Regional Office for Europe. http://www.euro.who.int/__data/assets/pdf_file/0017/143153/e95052.pdf	Searched and added to table	11. Not relevant

Email alert HQIP (n=1)

For peer review only

THE JOANNA BRIGGS INSTITUTE

JBI Critical Appraisal Checklist for Case Reports

Reviewer	
Date reviewed	
Author	
Year	

	Yes	No	Unclear	Not applicable
1. Were patient's demographic characteristics clearly described?	☐	☐	☐	☐
2. Was the patient's history clearly described and presented as a timeline?	☐	☐	☐	☐
3. Was the current clinical condition of the patient on presentation clearly described?	☐	☐	☐	☐
4. Were diagnostic tests or assessment methods and the results clearly described?	☐	☐	☐	☐
5. Was the intervention(s) or treatment procedure(s) clearly described?	☐	☐	☐	☐
6. Was the post-intervention clinical condition clearly described?	☐	☐	☐	☐
7. Were adverse events (harms) or unanticipated events identified and described?	☐	☐	☐	☐
8. Does the case report provide takeaway lessons?	☐	☐	☐	☐

Overall appraisal: Include ☐ Exclude ☐ Seek further info ☐

Comments (including reason for exclusion):

For peer review only

Table 1 Characteristics of case studies included in the review

Author, Year<br><br>Country<br><br>Purpose of article	Setting<br><br>Demographic characteristics	Focus of case study	Discussion
1 Ang et al 2009[148]<br><br>Singapore<br><br>Addresses the challenge of what should be done in the case of people with chronic conditions which impair their capacity	Emergency hospital care of a woman in her 40s with a diagnosis of schizoaffective disorder since her early 20s and with recent end stage renal failure	Ethical issues in EoLC for people lacking capacity to make decisions	Autonomy of a patient with regard to medical treatment, assessment of the patient's decisional capacity, and the process of deciding on the appropriate course to take in a patient without the mental capacity to give consent for required treatment in a potentially life-threatening situation<br><br>Legal frameworks, tools to assess capacity and the use of advanced ('Ulysses') contracts are all addressed
2 Badger and Ekham 2011[116]<br><br>USA<br><br>Ethical conflicts at the end of life for patients with personality disorder, substance misuse and self-harming behaviour	A case of a man in his 40s with long-term MH problems whose physical health declines (liver and respiratory failure) and whose care presents an ethical challenge to practitioners	Ethical issues in EoLC for people lacking capacity to make decisions	Decision-making for people lacking capacity is difficult in the absence of ADs and in the case of people with complex MH and substance misuse problems
3 Bakker 2000[109]	A residential service using DSA for older people with complex mental and	The use of the DSA approach, including in the example of people	The DSA approach is offered as a psychosocial counterbalance to more

For peer review only

Netherlands<br><br>Focuses on the PC of patients in a 'reactivation program', this being a service for older people with complex health problems	physical health problems, in which the case is a woman in her 90s	who end their lives in the DSA service, illustrating the phases of the approach in action	exclusively biomedically oriented approaches to care
4 Boyd et al 1997[117]<br><br>USA<br><br>The process of care for a hospice patient described as manipulating family, friends, and hospice staff during her EoLC	A hospice service for people at the EoL, with the case being a woman in her 50s with breast cancer who is described as having a pre-existing (but not clearly described) severe MH problem characterised by 'manipulative' behaviour	The challenges faced by hospice staff in providing care to someone perceived as being manipulative, and how these can be addressed using practices informed by psychodynamic thinking	Hospice staff can provide care for people with 'troubled personality' using psychodynamic approaches, including supporting ego functioning; supporting autonomy; setting limits; and assessing and managing staff emotional responses
5 Cabaret et al 2002[146]<br><br>France<br><br>The provision of EoLC in an inpatient psychiatric hospital setting	An inpatient psychiatric hospital, with the case being a man in his 40s with a diagnosis of schizophrenia and lung cancer	The provision of humane, palliative, care in a MH hospital	Inpatient MH staff are able to provide humane, dignified, care until the EoL for people with SMI
6 Candilis and Foti 1999[118]<br>USA	A case study beginning in an ED and moving to a MH unit, focused on the care of a woman in her 70s with cancer and psychosis	Decision-making in the context of EoLC and psychosis, where the patient's understanding and preferences are not clearly known	Differences in view can exist over the best course of action in cases where patients refuse treatment but do not elaborate supportive, palliative, care or more aggressive intervention

For peer review only

EoLC to someone with psychosis and cancer, where the patient's preferences are not clearly known			
7 Clements-Cortes 2004[143]<br><br>Canada<br><br>The use of music therapy in the emotional care of people who are at the EoL	A discussion on the value of music therapy, followed by three case studies all of people using an older people's PC inpatient service, of which two (women in their 70s) have pre-existing MH problems	The value of music in providing emotional support to people with terminal illnesses	Music therapy can facilitate emotional expression, including in people with SMI
8 Doron and Mendlovic 2008[147]<br><br>Israel<br><br>EoLC in inpatient MH wards	Two case studies of people, one in her 40s at her death and the other a woman whose age at death is not stated, cared for near to the EoL in inpatient MH settings, where both had a diagnosis of SMI and cancer	The challenges for both patients and staff of being cared for during a terminal illness in a MH inpatient setting	Information on the patient's condition and prognosis should be given in the context of their capacity to understand, decisions should be made by patients where possible or otherwise by clinicians and home is where people should die meaning that if this is a psychiatric ward staff should be supported to provide care for as long as possible
9 Feely et al 2013[119]<br><br>USA<br><br>EoLC in a hospice for people with borderline personality disorder	A case study of a woman in her 40s with a diagnosis of borderline personality disorder and terminal metastatic pancreatic cancer, being cared for in a hospice	The difficulties for staff in hospices of providing care for people with complex MH problems characterised by impulsive behaviour and difficulties in relationships	There is a lack of evidence to support interventions with people with borderline personality disorder at the EoL, but behavioural interventions should be tailored to the person based on the creation of genuine, respectful, relationships

10 Feldman and Petriyakoil 2006[120]<br><br>USA<br><br>PTSD at EoL	A case study of a male veteran in his 60s with heart failure and acute onset of PTSD symptoms.	Challenges of recurrence of latent PTSD symptoms seemingly triggered by the threat to life of terminal illness mimicking previous experiences.	Specific challenges in EoLC for people with PTSD may mean they experience psychological symptoms that have been dormant, become anxious and angry, avoid healthcare, distrust medical recommendations leading to nonadherence and refusal of treatment
11 Feldman et al 2014[110]<br><br>USA<br><br>The provision of PC to people with PTSD at the EoL	A re-presentation of a new model of care to people with a diagnosis of PTSD, either pre-existing or post-EoL diagnosis, illustrated by three case studies (men in their 60s) in diverse PC settings	An illustrated model of care for people with PTSD at the EoL, involving therapy over three distinct stages: Palliate Immediate Discomfort and Provide Social Supports; Provide Psychoeducation and Enhance Coping Skills; and Treat Specific Trauma Issues	The SPPC model can be used to support terminally ill patients with PTSD where conventional PTSD interventions are challenged by limited life expectancy, fatigue and other concerns
12 Feldman et al 2017[111]<br><br>USA<br><br>The provision of PC to people with PTSD at the EoL	A case of a male veteran in his 70s with a diagnosis of PTSD and multiple myeloma, admitted for inpatient hospice care at the EoL	How a staged approach to PTSD care is helpful for people at the EoL	A further illustration of how the SPPC model can be used to support terminally ill patients with PTSD where conventional PTSD interventions are challenged by limited life expectancy, fatigue and other concerns
13 Geppert et al 2011[121]<br><br>USA	A detailed case study of a man in his 50s with a pre-existing a diagnosis of schizoaffective disorder who has	The ethical issues around treatment when people have psychosis and lack decisional capacity	Psychosis raises ethical issues for EoLC as it undermines decisional capacity, raises questions about the use of proxies and additional questions about where care ought to

For peer review only

PC and capacity in the context of people with psychosis and terminal cancer	terminal laryngeal cancer and who is admitted to hospital		be provided (MH services, or ned of life services)
14 Gonzalez et al 2009[122]<br><br>USA<br><br>Hospice care of a man with breast cancer and bipolar disorder	A medical case study of a man in his 80s with complex health needs who receives inpatient care, and who has a diagnosis of bipolar disorder, dementia and breast cancer first treated 20 years previously	The complexity of providing care for people with multiple needs, in the context of family members also having significant health needs	Curative care is difficult to provide if treatment is not consistently provided and accepted, and PC is appropriate particularly when caregivers have health needs of their own
15 Griffith 2007[123]<br><br>USA<br><br>Palliative and EoLC for a man with heart and lung failure and schizophrenia [pt 1 of 2]	An extended case study of a man in his 60s, focusing on his schizophrenia diagnosis and his lung and heart disease and his care at home until death	The role of the psychiatrist in the provision of integrated care, including at the EoL, to people with SMI and multi-organ failure	Psychiatrists have an important part to play in treating people who have both severe MH and terminal physical health problems
16 Griffith 2007[124]<br><br>USA<br><br>Palliative and EoLC for a man with heart and lung failure and schizophrenia [pt 2 of 2]	An extended case study of a man in his 60s, focusing on his schizophrenia diagnosis and his lung and heart disease and his care at home until death	The role of the psychiatrist in the provision of integrated care, including at the EoL, to people with SMI and multi-organ failure	Psychiatrists should have cultural and religious competence and be able to work with families
17 Hill 2005[125]<br><br>USA	A case of a woman in her 80s described as having borderline personality disorder and hospice care at home	EoLC for people with a diagnosis of borderline personality disorder and complex physical health problems and family histories	Nurses should develop therapeutic relationships, have good supervision, be consistent in caregiving, and have

For peer review only

Hospice at home care for a person with borderline personality disorder			good working relationships with families and psychiatrists
18 Irwin et al 2014[26]<br><br>USA<br><br>Cancer care, including to EoL, for people with schizophrenia	A detailed review of literature followed by a case study of a woman in her 60s with a diagnosis of paranoid schizophrenia and terminal cancer, whose care moves from home to inpatient hospice	Health inequalities for people with a diagnosis of schizophrenia and cancer	Practitioners need improved understanding of health disparities experienced by people with a diagnosis of schizophrenia and cancer, research is needed to examine factors influencing survival and quality of life and better integration is required between oncology and psychiatric services
19 Kadri et al 2014[144]<br><br>Canada<br><br>Care for a person with schizophrenia and advanced kidney disease who lacks capacity and resists treatment	A case of a woman in her 50s with a diagnosis of schizophrenia and advanced chronic kidney disease admitted to hospital as an emergency and who refuses dialysis, then receives supportive care	Ethical issues in the case of people with SMI and who are unable to make decisions, but who actively resist treatment	Two ethical issues are highlighted: the lack of acceptance of treatment, and the risk of doing immediate harm if treatment is continued nonetheless
20. Kennedy et al 2013[114]<br><br>UK<br><br>Homelessness and EoLC	A case study of a woman in her 50s with diagnosis of schizophrenia and cervical cancer living semi-independently in a one bedroomed flat.	Treatment refusal in someone with severe mental illness who has the capacity to make decisions and her determination to return home to live.	Providing care for a person in their own home involving multiple professional teams in support of her autonomous decision to remain out of hospital and considering her refusal of active treatment
21 Kunkel et al 1997[126]<br><br>USA	A discussion of denial of cancer followed by five illustrative cases:<br><br>a woman in her 30s with a diagnosis of psychosis and breast cancer, who	A review of factors associated with non-compliance in people with cancer, specifically psychoses and cognitive impairment Both are shown to play a role in delayed help-seeking and non-	Key issues affecting cancer treatment are competence, the place for legal intervention, ambivalence towards health care providers and the

Denial of cancer and non-compliance in treatment	misses treatment and dies following an emergency admission;<br><br>a woman in her 50s with presumed 'organic personality syndrome' and squamous cell carcinoma who was psychotic and who died in hospital;<br><br>-a man in his 70s with presumptive pancreatic cancer and pre-existing organic delusional disorder and a diagnosis of major depression with psychosis, who had home hospice care;<br><br>a woman in her 70s with a diagnosis of dementia and breast cancer;<br><br>a woman in her 70s with a diagnosis of schizophrenia, superimposed dementia and lung cancer	compliance with cancer, and EoL, care	importance of early psychiatric intervention
22 Levin and Feldman 1983[127]<br><br>USA (the authors are based in South Africa)<br><br>Ethical issues surrounding the care of people with psychosis and terminal cancer	A case of a woman in her 30s with a diagnosis of schizophrenia and terminal breast cancer who is described as wanting to leave hospital without medical care following the treatment of an acute episode of her mental illness	A discussion of ethical issues associated with the care of people with SMI and terminal illnesses when treatment is refused	People have a right to refuse high-powered medicine at the EoL
23 Lopez et al 2010[128]	The case of a woman in her 30s with a diagnosis of chronic anorexia	Discussion of ethical and legal issues associated with caring for a person with a psychiatric condition who is not	Refusal of treatment can be irrational but must be weighed against workers obligations and duty of care and the

For peer review only

USA<br><br>Explores how medical futility and principles of palliation may contribute to the management of treatment refractory anorexia nervosa	nervosa, treated unsuccessfully for several years	helped by treatment where the condition ultimately leads to her death	autonomy of the individual. Two broad options are considered, 1) involuntary treatment, or 2) attempting to motivate the person until such time as they are willing to engage in treatment. The first option may have kept her alive in the short term but seemed unlikely to reverse the underlying condition
24 Maloney 2014[115]<br><br>USA [blog]<br><br>https://www.thejournal.ie/readme/bipolar-ii-disorder-denial-mental-health-1467162-May2014<br><br>A wife's experiences of her husband's bipolar disorder and terminal cancer	A personal blogpost recording a woman's experiences of her husband's diagnosis with a diagnosis of bipolar disorder, his subsequent terminal cancer and his death at home	A first-hand, narrative, account of long-term experiences ending in the death of a loved one	SMI changes people's lives, and can cause people to lose touch with reality People affected (patients and families) need support
25 Mason and Bowman 2018[112]<br><br>Country unknown (conference abstract)<br><br>The challenges of providing cancer care at the EoL to people with SMI and fluctuating capacity	A conference proceedings abstract describing the care of a man in his 70s with a diagnosis of schizophrenia and fluctuating capacity admitted to hospital for investigations and care into prostate cancer and metastases	An account of the challenges for people with SMI and fluctuating capacity in the context of EoL cancer care	Determining capacity in the context of SMI and acute physical illness is difficult, where standardisation in capacity assessment may help
26 McCasland 2007[129]<br><br>USA	A case of a woman in her 50s with a diagnosis of schizoaffective disorder, alcohol problems, liver damage and	Hospice nurses need better preparation to look after people at the EoL who also have SMI	Communication skills are important for hospice nurses caring for people with SMI at the EoL, and hospice nurses

For peer review only

The care and treatment of people with SMI and cancer, including at the EoL	breast cancer who is cared for in the community until dying in a hospice		needs more training to care for this group of people
27 McKenna et al 1994[130]<br><br>USA<br><br>The treatment with clozapine of people with SMI and cancer, where treatment also includes antineoplastic medication	A letter to the editor describing a man in his 40s with a diagnosis of schizophrenia and lung cancer with a poor prognosis, treated with clozapine, whose care was transferred from a MH to a medical care institution	The combination of clozapine with anti-tumour drugs	Guidance is needed on the combination of clozapine (an anti-psychotic) with anti-cancer drugs
28 Mogg and Bartlett 2005[140]<br><br>UK<br><br>The care and treatment of a man with treatment-resistant psychosis and life-threatening renal failure	The case is a man in his forties with a 30-year history of psychosis and multiple admissions to MH hospital, now discovered to have life-threatening renal disease requiring regular dialysis	Different approaches to the assessment of capacity (outcome, status, functional), set in the context of the legal framework	Capacity can fluctuate, and 'best interests' need to be considered which can mean guidance needing to be sought from the courts
29 Moini and Levinson 2009[131]<br><br>USA<br><br>The care and treatment of people with simple schizophrenia and medical conditions	A case of a woman in her 40s with a diagnosis of simple schizophrenia who is lost to services following diagnosis of breast cancer with metastases, and who is located at a later point at the terminal stage of her illness	Approaches to working with people with a diagnosis of simple schizophrenia and medical problems	Proactive care is needed so that people stay in touch with services, though people with a diagnosis of simple schizophrenia are also likely to have capacity to decide on their courses of treatment
30 Monga et al 2015[132]<br><br>USA	The case is of a man in his 70s with a diagnosis of schizophrenia, treated with clozapine, who continues his antipsychotic medication whilst having	The use of clozapine as an atypical antipsychotic alongside the use of chemotherapy	There is limited information to guide management of clozapine treatment during chemotherapy, and close

For peer review only

The use of clozapine in people with psychosis who are also having chemotherapy	hospice care and treatment for oesophageal cancer		cooperation between psychiatrists and oncologists is needed
31 Muhtaseb et al 2001[141]<br><br>UK<br><br>The treatment of people with SMI and advanced, terminal, cancer	The case is of a man in his 70s with a diagnosis of schizophrenia and advanced basal cell carcinoma, who refuses active intervention	Treatment of advanced, life-threatening, cancer in people with SMI who refuse active intervention	Consent must be freely given, and people (including people with SMI) should be considered able to decide for themselves
32 O'Neill et al 1994[142]<br><br>UK<br><br>Hospice care for woman with anorexia nervosa	The case of a woman in her 20s with a diagnosis of anorexia admitted to a hospice in poor physical condition after 7 years of anorexia nervosa that was not helped by treatment.	Discusses the treatment and rapid decline to eventual death of a young woman from the complications associated with anorexia nervosa.	Recognises that psychiatric units do not have relevant expertise for providing complex physical healthcare. Says that her condition was identified as incurable by the psychiatric team and when the patient and her family accepted this she was then able to access appropriate care. Says hospice staff are familiar with other EoLC and that this largely worked for this woman helped by an excellent referral letter (no clue how this is judged) and ongoing support from the psychiatrist. They conclude that there is a role for carefully selected non-cancer patients to be cared for in SPC centres
33 Picot et al 2015[15]<br><br>Australia	The case is of a woman in her 40s who has a diagnosis of bipolar disorder and metastatic breast cancer,	An illustrative case showcasing the work of nurse practitioners involved in the IMhPaCT programme, which is	People with SMI at the EoL face additional problems of isolation, declining physical abilities, pain, and disintegrating selfhood Collaboration

For peer review only

The IMhPaCT, designed to improve care for people with SMI and life-limiting illnesses	who receives integrated MH and PC at home before dying in hospital	designed to bring together MH and PC for people with SMI	across specialities can enhance outcomes, and MH nurses have a role to play
34 Rice et al 2012[133]<br><br>USA<br><br>Patient-provider communication in EoLC	The case of a male veteran in his 50s with renal disease and a diagnosis of schizophrenia who refuses dialysis treatment.	The case provides an example of man refusing treatment in line with previous history and is found to lack capacity to decide in his best interests. Involvement of his sister who is ill-prepared to take on proxy decisions	Discussion hinges on challenges of enforcing physical treatments on a person who is refusing, handling autonomy versus coercion, recognition of need for building therapeutic alliances with the person and their surrogates
35 Rodriguez-Mayoral 2018[113]<br><br>Mexico<br><br>Integrating MH and PC for people with SMI and cancer	A short conference abstract describing a woman in her 70s with a diagnosis of bipolar disorder and advanced, untreated, colon cancer from which she dies	The case briefly describes how the woman was cared for until her death, free form symptoms of her mental illness	Cancer care regardless of disease stage should be integrated with palliative and MH care to control symptoms and quality of life and death
36 Romm et al 2009[134]<br><br>USA<br><br>Managing cancer care for people using inpatient MH services	The case is a man in his 40s with a diagnosis of schizophrenia and osteosarcoma for which amputation was indicated, and who refused surgical treatment but who was judged to lack capacity His mother assumed decision-making responsibility and concurred with her son, and PC was initiated	Treatment of people with acute MH problems and life-threatening cancer, and patients' capacity to understand and made decisions	Psychiatrists have responsibilities to represent the interests of people who are 'mentally compromised', including those who are also irreversibly medically compromised Collaboration with PC practitioners is important, and whilst SMI can impair capacity this is not necessarily so
37 Shah et al 2008[135]<br><br>USA	The case is a homeless man in his 60s with a diagnosis of schizophrenia and neglected basal cell carcinoma, and who refuses treatment in the	Informed consent is key to the provision of cancer treatment, and in	In the absence of being able to give informed consent interprofessional

For peer review only

The treatment of people with SMI and advanced, terminal, cancer	context of a lack of capacity and is then treated for his mental illness	cases where people lack capacity additional care needs to take	teams need to be involved, along with independent ethics committees
38 Stecker 1993[136]<br><br>USA<br><br>Caring for young people with life-threatening cancer on an acute psychiatric ward	The case is of a man in his 20s with presumed bipolar disorder and sarcoma who is admitted to an inpatient psychiatric unit and who causes staff to struggle with the acceptance of death	Staff in MH wards do not routinely care for young people at the EoL, and this case challenged staff	MH nurses need to be able to identify feelings preventing them from working therapeutically with patients
39 Steves and Williams 2016[137]<br><br>USA<br><br>EoLC for people with terminal illnesses and SMI	A very brief case of a man, of unknown age, who has a diagnosis of schizophrenia and terminal lung cancer but who has capacity and refuses treatment prior to his death	Services for people with SMI at the EoL are in short supply, MH nurses need training in EoLC, attention needs to be paid to the environment, communication needs to be effective, caregivers needs to be supported, family members may be resistant and other inpatients need to be supported following a death	Investments are needed in services and staff to care for increasing numbers of people with SMI who also have terminal medical conditions
40 Terpstra et al 2014[138]<br><br>USA<br><br>EoL hospice care for people with SMI	The case is of a man in his 60s with a diagnosis of schizophrenia living in adult foster care who reports physical symptoms and is found to have bladder cancer and brain masses, moves to an open hospice and whose care and treatment is then described	The complexity of providing hospice care for people with SMI and terminal cancer, and the role of nurses	People with schizophrenia diagnoses are often medically undertreated, have shortened life expectancies, and have care which is challenged by their placements, poor communication and symptom management

For peer review only

41 Thomson and Henry 2012[70]<br><br>USA<br><br>The challenge for oncology nurses of caring for people with SMI and cancer	A paper on oncology nursing for people with pre-existing SMI, including a series of brief case studies: a woman in her 30s with a diagnosis of major depression and then breast cancer; a man in his 40s with a diagnosis of bipolar disorder and advanced pancreatic cancer and a woman in her 20s with a diagnosis of schizophrenia and breast cancer	Care and treatment of people with pre-existing SMI at the EoL, with a particular emphasis on understanding mental illnesses and its treatment	Medical professionals need to be aware of premature mortality in people with SMI, and need to develop skills and knowledge in caring for people with SMI as an underserved group<br>Practice is advancing in the areas of drug treatments, case management, family work and social support
42 Webber 2012[145]<br><br>Canada<br><br>Caring for a man with SMI in his supported home prior to hospice care	A brief case study of a man of unknown age who has a diagnosis of schizophrenia and metastatic gastric cancer and who was cared for in his supported home for as long as possible until being transferred to a hospice, where he died	Pain control, control over of levels of intervention, control over maintaining meaningful, relationships, outcomes of lost autonomy, lack of resources, restoring justice for patients with SMI and terminal illness, and innovation are all discussed	Autonomy has been diminished for people with SMI and palliative illness, yet people with SMI at the life want the same things as everyone else<br>Services and care need to improve,

Key: DSA: dynamic system analysis; EoL: end of life; EoLC: end of life care: IMhPaCT: Integrated Mental Health and Palliative Care Task; PTSD: post-traumatic stress disorder; SMI: severe mental illness; SPC: specialist palliative care: SPPC: stepwise psychosocial palliative care

For peer review only

Citation	Q1	Q2	Q3	Q4	Q5	Q6	Q7	Q8	Score
1. Ang et al 2009[148]	Y	Y	Y	N	Y	Y	Y	Y	7/8
2. Badger and Ekham 2011[116]	Y	Y	Y	N	Y	Y	Y	Y	7/8
3. Bakker 2000[109]	Y	Y	Y	N	Y	Y	Y	Y	7/8
4. Boyd et al 1997[117]	Y	Y	Y	N	Y	Y	Y	Y	7/8
5. Cabaret et al 2002[146]	Y	Y	Y	N	Y	Y	Y	Y	7/8
6. Candilis and Foti 1999[118]	Y	Y	Y	N	Y	Y	Y	Y	7/8
7. Clements-Cortes 2004[143]	Y	Y	Y	N	Y	Y	N	Y	6/8
8. Doron and Mendlovic 2008[147]	Y	Y	Y	Y	Y	Y	Y	Y	8/8
9. Feely et al 2013[119]	Y	Y	Y	N	Y	Y	Y	Y	7/8
10. Feldman and Petriyakoil 2006[120]	Y	UC	Y	N	N	Y	Y	N	4/8
11. Feldman et al 2014[110]	Y	Y	Y	N	Y	Y	Y	Y	7/8
12. Feldman et al 2017[111]	Y	Y	Y	N	Y	Y	Y	Y	7/8
13. Geppert et al 2011[121]	Y	Y	Y	Y	Y	Y	Y	Y	8/8
14. Gonzelez et al 2009[122]	Y	Y	Y	Y	Y	Y	Y	Y	8/8
15. Griffith 2007[123]	Y	Y	N	Y	Y	Y	Y	Y	7/8
16. Griffith 2007[124]	Y	Y	N	Y	Y	Y	Y	Y	7/8
17. Hill 2005[125]	Y	Y	Y	Y	Y	Y	Y	Y	8/8
18. Irwin et al 2014[26]	Y	Y	Y	Y	Y	Y	Y	Y	8/8
19. Kadri et al 2018[144]	Y	N	Y	N	Y	N	Y	Y	5/8
20. Kennedy et al 2013[114]	Y	UC	Y	N	Y	Y	Y	Y	6/8
21. Kunkel et al 1997[126]	Y	Y	Y	Y	Y	Y	Y	Y	8/8
22. Levin and Feldman 1983[127]	Y	Y	Y	N	Y	N	Y	Y	6/8
23. Lopez et al 2010[128]	Y	Y	Y	N	Y	Y	N	Y	6/8
24. Maloney et al 2014[115]	Y	Y	Y	N	Y	Y	Y	Y	7/8
25. Mason and Bowman 2018[112]	Y	Y	Y	Y	Y	Y	Y	Y	8/8
26. McCasland 2007[129]	Y	Y	Y	Y	Y	Y	Y	Y	8/8
27. McKenna et al 1994[130]	Y	N	UC	N	Y	N	Y	Y	4/8
28. Mogg and Bartlett 2005[140]	Y	N	N	N	Y	N	N	Y	2/8
29. Moini and Levenson 2009[131]	Y	Y	Y	N	N	N	Y	Y	5/8
30. Monga et al 2015[132]	Y	Y	Y	Y	Y	Y	Y	Y	8/8
31. Muhtaseb et al 2001[141]	Y	Y	Y	Y	Y	Y	Y	Y	8/8
32. O'Neill et al 1994[142]	Y	N	Y	N	Y	N	N	N	2/8
33. Picot et al 2015[15]	Y	Y	Y	Y	Y	Y	Y	Y	8/8
34. Rice et al 2012[133]	Y	N	Y	N	Y	Y	Y	Y	6/8
35. Rodriguez-Mayoral 2018[113]	Y	N	N	N	N	Y	Y	Y	5/8
36. Romm et al 2009[134]	Y	N	Y	N	N	N	Y	Y	3/8
37. Shah et al 2008[135]	Y	N	Y	Y	Y	Y	Y	Y	7/8
38. Stecker 1993[136]	Y	N	Y	N	N	N	Y	Y	4/8
39. Steves and Willliams 2016[137]	Y	N	N	N	Y	Y	Y	Y	4/8
40. Terpstra et al 2014[138]	Y	Y	Y	Y	Y	Y	Y	Y	8/8
41.Thomson and Henry 2012[139]	Y	Y	N	N	N	N	N	Y	2/8
42. Webber 2012[145]	Y	N	N	N	N	Y	Y	Y	3/8

For peer review only

Key: Y=Yes, N=No, UC=unclear

Table 2: Critical appraisal scores for case studies

For peer review only

BMJ Open

End of life care for people with severe mental illness: mixed methods systematic review and thematic synthesis of published case studies (the MENLOC study)

Journal:	*BMJ Open*
Manuscript ID	bmjopen-2021-053223.R1
Article Type:	Original research
Date Submitted by the Author:	07-Oct-2021
Complete List of Authors:	Coffey, Michael; Swansea University, School of Health and Social Care<br>Edwards, Deborah; Cardiff University, School of Healthcare Sciences<br>Anstey, Sally; Cardiff University, School of Healthcare Sciences<br>Gill, Paul; Cardiff University<br>Mann, Mala; Cardiff University, University Library Services<br>Meudell, Alan; Service User Consultant<br>Hannigan, Ben; Cardiff Univ, School of Healthcare Sciences
**Primary Subject Heading**:	Palliative care
Secondary Subject Heading:	Mental health, Health services research
Keywords:	PALLIATIVE CARE, MENTAL HEALTH, ONCOLOGY, Organisation of health services < HEALTH SERVICES ADMINISTRATION & MANAGEMENT

SCHOLARONE™
Manuscripts

I, the Submitting Author has the right to grant and does grant on behalf of all authors of the Work (as defined in the below author licence), an exclusive licence and/or a non-exclusive licence for contributions from authors who are: i) UK Crown employees; ii) where BMJ has agreed a CC-BY licence shall apply, and/or iii) in accordance with the terms applicable for US Federal Government officers or employees acting as part of their official duties; on a worldwide, perpetual, irrevocable, royalty-free basis to BMJ Publishing Group Ltd ("BMJ") its licensees and where the relevant Journal is co-owned by BMJ to the co-owners of the Journal, to publish the Work in this journal and any other BMJ products and to exploit all rights, as set out in our licence.

The Submitting Author accepts and understands that any supply made under these terms is made by BMJ to the Submitting Author unless you are acting as an employee on behalf of your employer or a postgraduate student of an affiliated institution which is paying any applicable article publishing charge ("APC") for Open Access articles. Where the Submitting Author wishes to make the Work available on an Open Access basis (and intends to pay the relevant APC), the terms of reuse of such Open Access shall be governed by a Creative Commons licence – details of these licences and which Creative Commons licence will apply to this Work are set out in our licence referred to above.

Other than as permitted in any relevant BMJ Author's Self Archiving Policies, I confirm this Work has not been accepted for publication elsewhere, is not being considered for publication elsewhere and does not duplicate material already published. I confirm all authors consent to publication of this Work and authorise the granting of this licence.

For peer review only

End of life care for people with severe mental illness: mixed methods systematic review and thematic synthesis of published case studies (the MENLOC study)

Michael Coffey[a] https://orcid.org/0000-0002-0380-4704

Deborah Edwards[b] https://orcid.org/0000-0003-1885-9297

Sally Anstey[b] https://orcid.org/0000-0003-2295-3761

Paul Gill[b] https://orcid.org/0000-0003-4056-3230

Mala Mann[c] http://orcid.org/0000-0002-2554-9265

Alan Meudell[d] https://orcid.org/0000-0001-8138-4744

Ben Hannigan[b] https://orcid.org/0000-0002-2512-6721

Corresponding author: Michael Coffey, Department of Public Health, Policy and Social Sciences, College of Human and Health Sciences, Swansea University, UK, email: m.j.coffey@swansea.ac.uk

a School of Health and Social Care, Faculty of Medicine, Health and Life Science, Swansea University, UK

b School of Healthcare Sciences, College of Biomedical and Life Sciences, Cardiff University, UK

c Specialist Unit for Review Evidence, University Library Services, Cardiff University, UK

d Independent Service User Researcher, UK

For peer review only - http://bmjopen.bmj.com/site/about/guidelines.xhtml

Abstract:

Objectives: People with severe mental illness have significant co-morbidities and reduced life expectancy. The objective for the review reported in this paper was to synthesise material from case studies relating to the organisation, provision and receipt of care for people with severe mental illness who have an end-of-life diagnosis.

Design: Systematic review and thematic synthesis.

Data sources: MEDLINE, PsycINFO, EMBASE, HMIC, AMED, CINAHL, CENTRAL, ASSIA, DARE and Web of Science from inception to December 2019. Supplementary searching for additional material including grey literature along with 62 organisational websites.

Results: Of the 11,904 citations retrieved, 42 papers reporting 51 case studies were identified and are reported here. Twenty five of the 42 case study papers met seven, or more quality criteria with eight meeting half or less. Attributes of case study subjects included that just over half were men, had a mean age of 55 years, psychotic illnesses dominated, and the end-of-life condition was in most cases a cancer. Analysis generated themes as follows Diagnostic delay and overshadowing, Decision Capacity and Dilemmas, Medical Futility, Individuals and their networks, Care Provision.

Conclusions: In the absence of high-quality intervention studies, this evidence synthesis indicates that cross disciplinary care is supported within the context of established therapeutic relationships. Attention to potential delay and diagnostic overshadowing is required and care provision the values the preferences of individuals with severe mental illness experiencing an end-of-life condition is indicated.

Keywords: Palliative care**,** mental health, oncology, organisation of health services

PROSPERO registration number: CRD42018108988

For peer review only

Article Summary

Strengths and limitations of this study

- This systematic review represents, to our knowledge, the first synthesis of case studies in end-of-life care for people with prior severe mental illness.
- Case studies have a long history in medicine, provide useful insights, and fill an important gap in the absence of intervention research.
- We identified 51 case studies across 42 articles with 25 meeting seven or more of eight quality criteria.
- Most papers were from high income countries thus limiting transferability.
- Future research should focus on generating high quality intervention studies in this field.

For peer review only

For peer review only - http://bmjopen.bmj.com/site/about/guidelines.xhtml

Introduction

Mental ill-health is the leading cause of years lived with disability in 56 countries and the second leading cause in a further 56.[1] The wider economic costs of mental illness in England have been estimated at £105.2 billion each year[2] and £7.2 billion in Wales.[3]

The term 'severe' mental illness (SMI) has longstanding currency within the fields of mental health policy, services and practice.[4] It continues to be used in research,[5] and by the NIHR Dissemination Centre.[6] People with SMI have high co-morbidities,[7] and higher mortality rates and reduced life expectancy compared to the general population[8] across all age groups,[9] with a 10–20-year reduction in life expectancy.[10] Inequities, not limited to care at the end of life specifically, can be explained with reference to individual and system-level factors. People with SMI are less likely to attend health screenings and may respond to symptoms differently.[11] They may delay or avoid help, and are more likely to exhibit disruptive behaviours or miss contacts with health professionals[12,13] putting them at risk of delayed disease detection.[7] Inadequate support systems are also common among those with SMI, affecting their ability to access appropriate clinical care and navigate complex health systems.[14] Other factors influencing variations in mortality and morbidity for people living with SMI include poor previous experiences of seeking help from health care professionals, incorrect attribution of physical symptoms to psychiatric disorder by care staff, and lack of experience by mental health professionals in determining how and when to refer onwards to other appropriate services.[12,15]

End-of-life care refers to the care of people with diagnoses of advanced, incurable, cancer and/or end-stage lung, heart, renal or liver failure and who are likely to die within the next 12 months[16]. It includes care provided in hospitals, hospices, and other institutional settings (such as care homes, prisons, and hostels) and care provided at home and via outreach to people who may also be homeless.

Although cancer incidence amongst people living with SMI is similar to that of the general population, mortality rates are double.[17,18] This disparity may be related in part to late presentation and reduced use of interventions such as surgery, systemic anti-cancer therapies or radiotherapy.[19,20] The experience of SMI can delay access, detection and

For peer review only - http://bmjopen.bmj.com/site/about/guidelines.xhtml

treatment of life-threatening physical disorders and their symptoms, specifically pain.[21] Consequently, this patient cohort is more likely to present advanced cancers that are more complex and costly to treat, being less likely to undergo invasive treatments and more likely to die.[22]

Research at the interface of physical and mental health care is recognised as a priority.[23] Policies focus on improving end-of-life care, where diagnosis is immaterial.[24-32] These policies require the introduction of palliative and supportive care earlier in the illness trajectory. In national policy, the needs of people with SMI who develop advanced incurable cancer and/or end-stage lung, heart, renal or liver failure are acknowledged poorly, or not at all. This group face the prospect of 'disadvantaged dying',[33 34] at a time when quality of care in the last months of life should be uniformly high for all groups.

Our work is distinguished from other work in this field as we adopt an inclusive approach to consider policy papers, primary research and in this paper we report the inclusion of published case studies as a further part of our NIHR funded systematic review focused on published evidence for end-of-life care for people with severe mental illness.[35] For the purposes of this review all descriptions of the care and experiences of individuals with pre-existing SMI and EoL diagnosis identified via our search strategy. Case studies were excluded if no SMI condition was identified, if no EoL condition was identified or if the mental illness was not pre-existing.

Case studies have a long tradition in healthcare[36] aiming to provide insights from the specific, to illustrate broader lessons[37]. Given the limited research into end-of-life care for people experiencing severe mental illness, a synthesis of case studies can contribute new understandings and direct us towards patterns of care that might otherwise remain hidden.

Methods

The protocol is registered in PROSPERO (CRD42018108988) (see supplementary file 1)and followed the Preferred Reporting Items for Systematic Review and Meta-Analysis (PRISMA)[38] (see supplementary file 2). The methods are reported in detail in the full project report[35] and summarised here.

For peer review only - http://bmjopen.bmj.com/site/about/guidelines.xhtml

Ethics Statement

This is a systematic review, no patient data was collected for this review and research ethics approval was not required.

Inclusion criteria

Population

Adult participants (>18 years of age) with SMI who have an additional diagnosis of advanced, incurable, cancer and/or end-stage lung, heart, renal or liver failure and who are likely to die within the next 12 months were considered. SMI was defined as including those with but not limited to schizophrenia, schizophrenia spectrum and other psychotic disorders, schizotypal and delusional disorders, bipolar affective disorder, bipolar and related disorders, major depressive disorder and disorders of adult personality and behaviour. SMI is an imprecise term and definitions also include duration and disability as criteria[4 6]. Therefore, we additionally included studies of enduring conditions such as PTSD and anorexia nervosa where our searches located them[35].

Types of intervention and phenomena of interest

End-of-life care.

Context

End of life care provided in hospitals, hospices, and other institutional settings (such as care homes, prisons, and hostels), and care provided in the home and via outreach to people who may also be homeless.

Types of evidence

Case studies.

Exclusion criteria

Evidence relating to:

- mental health problems (e.g., depression) as a consequence of terminal illness (e.g., cancer or chronic organ failure).

For peer review only - http://bmjopen.bmj.com/site/about/guidelines.xhtml

- End-of-life care for people with mental and behavioural disorders due to psychoactive substance use, except where these coexisted with SMI as specified above.
- End-of-life care for people with dementia or other neurodegenerative diseases except where these coexisted with SMI as specified above.

In this review, we specifically focused on gathering evidence of end-of-life care for people with pre-existing SMI who would, broadly put, have used secondary mental health services. We acknowledge the importance of end-of-life care for people with neurodegenerative disorders, frailty and other conditions but recognise these as a separate population, and in the interests of achieving a more focused review we excluded material in these areas.

The search strategy was developed for Ovid Medline and adapted for the other databases (see supplementary file 3). Searches were run on the following databases from inception for studies published in the English language.

- MEDLINE ALL; EMBASE; HMIC, PsycINFO; AMED;
- CINAHL; CENTRAL;
- ASSIA;
- DARE; Web of Science (WoS).

The keywords that were used to inform these searches included the following:

Palliative care OR Hospice care OR Terminal care OR Terminally ill OR End of life care OR Last year of life

AND

Neoplasms OR Cancer OR heart failure, lung failure, liver failure or lung failure

AND

Mental health OR Depression OR Mental disorders OR Depressive disorder OR Personality disorders OR Bipolar disorder OR Schizophrenia OR Mental illness.

Supplementary searches were conducted for additional papers, information on studies in progress, unpublished research, research reported in the grey literature and personal blogs (see supplementary file 4).

For peer review only - http://bmjopen.bmj.com/site/about/guidelines.xhtml

Searches were also conducted using Google as described by Mahood et al.[39 40] The first 10 pages of each Google output were screened using the terms:

- "palliative care" and "mental illness"
- "end of life" and "mental illness"
- "end of life" and schizophrenia (searching first 5 pages of output)
- "end of life" and bipolar (searching first 5 pages of output)

The contents of the last 2 years of the *Journal of Pain and Symptom Management*, *Cancer*, *Psycho-Oncology* and *BMJ Supportive & Palliative Care* were hand-searched. These journals were selected due to the large number of outputs identified in database searches being published within them. Reference lists of included studies were scanned, and forward citation tracking performed using WoS.

Screening

References were collected and deduplicated using Covidence™ systematic review software (Veritas Health Innovation, Melbourne, Australia).

Data extraction

Demographic data were extracted into tables and followed the format recommended by the CRD.[40] These data were checked by a second reviewer independently for accuracy and completeness. Where multiple publications from the same study were identified, data were extracted and reported as a single study.

Data synthesis

All case studies were available in full text form, were read and re-read, uploaded to NVivo, inductively coded by one reviewer and checked by a second, and then synthesised into five themes[41].

For peer review only

For peer review only - http://bmjopen.bmj.com/site/about/guidelines.xhtml

Assessment of methodological quality

The JBI critical appraisal checklist for case reports was used.[42] This is an eight-item checklist (‘yes’, ‘no’, ‘unclear’, ‘not applicable’) from which an overall score is generated reflecting the number of items answered ‘Yes’ (see supplementary file 5). Items relate to risk of bias, adequate reporting and statistical analysis.

Patient and public involvement

This study included public and patient involvement perspectives commencing with original concept, the definition of search terms and parameters, study steering group, and onwards to impact and dissemination (e.g., co-author on report and papers).

Results

Search results

Of the 11,904 citations retrieved, 42 case study papers were identified and are reported in this current paper. The PRISMA flow diagram is shown in figure 1.

Figure 1 PRISMA flow chart here

Description of case studies

There were 42 publications containing 51 case studies of individuals with an existing mental illness diagnosis who went on to develop an end-of-life condition (see Table 1, supplementary file 6). In four case studies the purpose of the paper was to show the application of a particular model of care e.g. Dynamic System Analysis (DSA),[43] or Stepwise Psychosocial Palliative Care (SPPC).[44 45] Case studies were mostly published in peer reviewed research journals (n=38) with two conference abstracts,[46 47] one appearing in a report[48] and one a first-person account blog.[49] The case studies ranged in depth from discursive papers with little direct detail about the individuals involved, to those that focused mainly on the patients’ physical illness. Case studies reported on the health and/or social care provided to individuals, and as such provided insights into everyday practice and its associated opportunities, challenges, and dilemmas.

For peer review only - http://bmjopen.bmj.com/site/about/guidelines.xhtml

Country of research

Papers were published in the US (n=28);[20,44,45,49-72] UK (n=4);[48 73-75] Canada (n=3);[76-78] Australia (n=1);[9] France (n=1);[79] Israel (n=1);[80] Mexico (n=1);[47] Netherlands (n=1);[43] Singapore (n=1);[81] with one conference abstract not stating a country.[46]

Year of publication

The earliest case study was published in 1983[60] and outputs appeared regularly up until the end of the search period in 2019. Figure 2 displays the year of publication of the 42 case study articles.

Figure 2 HERE

Attributes of service users

The age range across the case studies was 20-91 years (mean 55 years) and was provided for all except two.[49 70] Women were the focus of 24 case studies. Diagnosis was reported for all case study individuals and included psychotic-type diagnoses (schizophrenia, psychosis, schizoaffective and bipolar conditions) (31 publications reporting 35 case studies),[9,20,43,46-49,52,55-57 82,59,60,62-74,76-81] personality disorder (5 publications reporting 5 case studies),[50,51,53,58 59] Post Traumatic Stress Disorder (PTSD) (3 publications reporting 3 case studies)[44,45,54] and anorexia nervosa (n=2).[61,75] The anorexia case studies are two outliers in that the end-of-life condition was a direct result of the mental health issue for those individuals leading to chronic fractures and organ failure.[61,75] The vast majority of end-of-life conditions presented in the case study papers were cancer related diagnoses, and organ failure (heart (n=3 (across four publications),[43,54,57,82] liver (n=1)[50] and renal (n=5)[58,66,73,77,81] made up the remainder.

Critical appraisal scores

The quality of the studies varied overall (see Table 2, supplementary file 6). Twenty five of the 42 papers met seven or more criteria. In most cases the single criterion missing related to the description of diagnostic tests or assessment methods and their results. Eight of the

For peer review only - http://bmjopen.bmj.com/site/about/guidelines.xhtml

42 papers met half or fewer of the eight quality criteria, but for the purposes of inclusivity all 42 papers were included in the subsequent narrative synthesis.

Thematic synthesis

These case studies report on key episodes, or critical junctures,[83] in the lives of people with SMI at the end-of-life. Five themes were identified centring on people with SMI presenting to health services and then disappearing again, or pivotal decisions with profound impact for patients and others. These themes are *Diagnostic delay and overshadowing*; *Decisional capacity and dilemmas*; *Medical futility; Individuals and their networks*; and *Care provision*.

Diagnostic delay and overshadowing

A consistent picture of delayed or late diagnosis and diagnostic overshadowing appears in case studies. Complex and enduring mental health problems may contribute to these issues. The problem of delay is signalled as arising from the mental illness of the individual who fails to recognise the seriousness of their plight, and presents to services only when their condition is advanced and treatment options limited.[52,59,60,64,71] For example, Ms A, an African American woman in her thirties with an unspecified psychosis presented with a 20cm mass in her right breast adhering to the chest wall.[59] Investigations revealed a significant cancer with lymph node involvement with no metastases. Ms A was treated with chemotherapy and surgery, failed to attend for radiation treatment and considered new lesions as nothing more than 'haematoma'. She later presented in respiratory distress with large, presumably malignant effusions, asked not to be resuscitated and died soon after. The case study paper labels Ms A's difficulty with accepting her situation and similar behaviours as 'maladaptive denial'.

The tendency to impute the mental conditions of individuals as a source of late presentation, or indeed for deciding not to continue with treatment, is a recurring feature of case studies. Case studies indicate how diagnosis and treatment of serious life-threatening or life-limiting physical conditions are often complicated by untreated mental illness and the beliefs and behaviours arising from this.[59,60,64] Some case studies appear to merge conceptualisation of behavioural aspects of presentations with mental ill-health and position these as problematic in the delivery of care and treatment with terms such as

For peer review only - http://bmjopen.bmj.com/site/about/guidelines.xhtml

'malingering', 'manipulative', 'dominance and aggression', 'demanding',[43,50,51,53] or problems for staff who 'struggled with how best to provide care'.[54]

Denial or a lack of insight, is a possible complicating factor. It is not unknown for persons diagnosed with other serious conditions to deny the gravity of their situation, and for people with pre-existing mental health conditions it appears that this is no different.[59] Specific issues arise in cases of PTSD where 'the threat to life inherent in terminal illness may mimic the original trauma' leading to exacerbation of psychological symptoms associated with the condition including anxiety, anger, denial, avoidance and distrust of authority impeding medical adherence resulting in refusal of treatment.[54]

Individuals with long histories of pre-existing mental illness with regular access to healthcare professionals nevertheless experience delayed or late diagnosis of conditions that place them on the end-of-life trajectory.[20,47,55,65,78-80] For example, a man in his sixties with a longstanding diagnosis of schizophrenia, living in an adult foster home and under legal guardianship,[71] was seen fortnightly at a mental health clinic and attended a primary care provider to report a new onset cough. His examination was documented as benign but one week later he complained of hypotension and left-sided weakness. A detailed examination revealed advanced bladder cancer and multiple brain metastases not previously noted. The authors locate the problem in the mental condition suggesting people with conditions such as schizophrenia do not willingly verbalise pain or related symptoms. It is however difficult to escape the conclusion that extended contact with healthcare services had failed to identify his condition.

Decisional capacity and dilemmas

Decisional capacity of individuals to consent to treatment and/or to refuse treatments are reported and implicate professional dilemmas of determining the value of attempting curative treatments versus palliation.[46,48,50,55,59,60,73,74,81] It is argued[67] that clinicians have a particular duty to ensure that the interests of people with SMI are defended by offering medical treatment. While SMI can impair decisional capacity, this should not be assumed but instead thoroughly assessed.[67] In some examples the conclusion is that the person

For peer review only

retained capacity to determine their treatment choices, and these choices were then respected.[48]

Treatment refusal is highlighted numerous times.[48,52,55,56,60,63,66,67,70,72-74] Past refusals prompt treatment teams to impute future problems.[66] For example, a patient in their eighties with a 20-year history of a mastectomy for breast cancer and refusal of medical care, re-presented with a bleeding and ulcerating mass on the chest wall.[56] The case study reports that the patient was not a case for curative treatment despite no evidence of metastases.

Treatment refusal is reported as arising from psychiatric symptomatology such as fixed beliefs about damnation or that thoughts could be read by physicians, rather than due to capacity to understand and make decisions based on available information.[20]

Fluctuating mental capacity requiring multiple assessments[46,73] is reported. This can mean resort to the courts in treatment refusal[73] for declaration of lawfulness, being in the best interests of the patient, or of not imposing treatment notwithstanding the patient's inability to accept or refuse treatment. In one case of treatment refusal in a man with decisional incapacity, the medical ethics committee concluded that even with full decision-making capacity a person might reasonably refuse radical procedures due to risks involved and the deforming nature of surgery.[68]

The absence of previously declared wishes on life-saving treatments is a recurring issue and suggests one area for future intervention testing.[59 66 67 77] In these circumstances teams seek agreement of a substitute decision maker, such as a family member[66,67] sometimes with power of attorney. The patient may however indicate by their actions their refusal e.g., repeatedly removing lifesaving treatments such as catheters in renal dialysis[77] or not agreeing to take medication.[59] Enforcing medical treatment when it is actively refused is not supported in this literature and may complicate future alliance building.[66]

Medical futility

The concept of medical futility is invoked in case studies of people with anorexia nervosa indicting how experience of this condition can exhaust the optimism of those doing the

For peer review only - http://bmjopen.bmj.com/site/about/guidelines.xhtml

caring.[61] The language used, e.g. 'refractory' and 'incurable' reflects this.[75] Ms A, a woman in her early thirties with a diagnosis of anorexia nervosa where there is use of palliation and referral to hospice care for the physical consequences of the psychiatric condition.[61] In this case it is also reported that the option of an eating disorder treatment programme was eventually rejected on cost grounds, implying that perhaps the situation was less futile than indicated. Nevertheless, the treatment team and the ethics committee concluded, 'that her physical and psychiatric impairments were likely to lead to her death, despite any plausible attempts at aggressive intervention'.[61, p.373]

Individuals and their networks

Support networks for people with SMI are crucial, and the absence of these is implicated in delayed treatment-seeking. Case studies refer to family involvement,[43,57,82,62,72,81] whilst others indicate the absence of such support.[50,52] The end-of-life condition in some circumstances appears to have led to the re-emergence of family support,[79] or that teams actively supported the person to reconnect with distant family.[48] For some, tensions in family involvement are reported[51,58] and in one example family dynamics are situated as the source of subsequent mental health-related troubles.[75] Case studies also identify family concerns for the individual with the end-of-life condition.[57,82] End of life care places additional demands on families, such as learning to manage symptoms[44] and can lead to exhausted or burned-out family members.[61] One such demand arises from the absence of advance decisions, or where decisional capacity is in question. Families are then drawn into discussions on treatment and do not resuscitate decisions for which they are ill-prepared.[55,59,66,67,77] Families also express concern that the person's mental health problems are overlooked by the palliative care team.[72]

Care Provision

Case studies reveal issues in the provision of care for treatment teams, such as how to handle psychiatric presentations.[43,51,66,72] Case studies also report examples of what has worked in supporting people with mental health problems at the end-of-life, including: the building of rapport and trust especially in people with PTSD;[44] the use of music therapy at end-of-life;[76] having conversations about death;[20,57,82,79] initiating hospice at home;[20,57,82] and multidisciplinary mental health care and palliative care being provided at home.[48]

For peer review only - http://bmjopen.bmj.com/site/about/guidelines.xhtml

Challenges reported include mental health staff being emotionally unprepared for caring for people who are terminally ill.[69] Where people with SMI receive end-of-life care in hospices palliative care staff experience strong emotions, such as anger and frustration.[44] Case study papers occasionally offer psychodynamic interpretations of staff/patient interactions and care.[43,58,69]

Case studies reporting the ongoing delivery of care to individuals with pre-existing SMI and subsequent end-of-life advocate the benefits of a transdisciplinary approach involving palliative care specialists, psychiatric specialists (preferably the team with a pre-existing relationship) and wider community members (e.g., religious ministers).[9,20,47,48,61,67,75]

Liaison consultation and collaboration is reported as beneficial for hospice staff. Examples include psychiatric consultation,[53] psychologists advising on reducing environmental stress[44] and a hospice nurse being supported by a psychiatric nurse.[58] Mutual benefits of hospice and mental health nurses working together in end-of-life care, and the similarities in their work are reported. Hospice staff[72] and mental health staff[79] express interest in learning from each other when providing end of life care for people with mental health problems.

Where models of care are reported these are small scale and do not indicate transferability or generalisability. Examples include treatment models e.g., DSA,[43] SPPC[44,45] and patient, provider, systems model.[20]

In almost every example the place of care is positioned as one arising from the preferences of the individual. These include palliative care in mental health institutions;[55,79,80] hospice care for mental health patients;[53,62,71,75] acute hospital;[9] and home care.[48] Repeated transfer between settings related to psychiatric and/or physical symptomatology is also evident[66] suggesting challenges to continuity of care.

Treatment challenges are reported in managing mental health-related medication alongside the provision of chemotherapy. Two case study papers report issues with the prescription of clozapine, as this is implicated in depressing white blood cell counts and could precipitate a life-threatening infection compounding the neutropenia associated with cytotoxic

For peer review only - http://bmjopen.bmj.com/site/about/guidelines.xhtml

chemotherapy.[63,65] Treatment challenges also arise from people with SMI at the end-of-life absconding or being lost to follow-up.[64,74] A further challenge is in providing palliative care to patients who, despite careful explanation, continue to believe that they are not going to die.[61]

Discussion

This paper is the first to synthesise and present quality appraisal of published case studies of people with pre-existing severe mental illness with end-of-life diagnoses. Case studies demonstrate the complexities and the ethical dilemmas associated with the provision of care to people with SMI at the end-of-life. They reveal stark challenges presented by delayed diagnosis, patients' fluctuating capacity to make decisions and a concern about the futility of treatment. Case studies also demonstrate, the lack of preparedness of professionals to meet patients' multiple needs. For example, palliative care staff may lack mental health knowledge and mental health staff often have little or no experience of palliative care. Despite this, professionals want to learn from each other across specialities to provide better care. Synthesised case study findings also show the dangers of ascribing delays in mobilising (or continuing with) palliative care services in response to patients' challenging presentations, or difficult behaviour.

Access to palliative care is variable, even for the general population in many countries with universal standards lacking, late or delayed referral and limited access to best care practices.[84] Recent reviews and synthesis of evidence describing clinical care in this field reveal similarly variable provision.[85-87] Research on end-of-life care for people with mental health problems is limited[35] and in the absence of high quality intervention research, case studies provide an important source of knowledge. While there is much to learn from case studies, the reporting of clinical details is variable, thus limiting generalisation. A further challenge in synthesis arises in that almost all case studies come from high income countries, limiting transferability to other settings.

Practitioners sometimes struggle with engaging individuals in difficult discussions about their care. However, people with severe mental illness want these conversations.[88] Care provided across specialist boundaries and with the continuing support of already

For peer review only - http://bmjopen.bmj.com/site/about/guidelines.xhtml

established therapeutic relationships is well supported. In providing care at the end-of-life for people with severe mental illness practitioners must work to sustain these relationships, display compassion and sensitivity in difficult but necessary conversations, engage across disciplinary barriers to improve their understanding, and establish preferences of the person in relation to end-of-life decisions. This finding has implications for the education and training of professionals across multiple disciplines and must examine beliefs and attitudes towards mental ill health as well as assumptions about mental capacity and autonomy of people receiving services. In addition, legal contexts differ across and sometimes within countries, meaning the definition and understanding of issues such as mental capacity may vary.

The case studies synthesised here provide a range of clinical and non-clinical experiences of care for people with end-of-life conditions in the context of severe mental illness. These experiences point to a range of possible approaches for improving the prompt diagnosis and care of these individuals. In the absence of intervention studies, it remains unclear how robust these indications are for future clinical care.

Limitations of this review include our focus on papers in the English language which may have led to the absence of important evidence published in other languages. The majority of case studies are from high-income countries and this limits generalisability to some extent, not least because there are likely to be contextual differences in health and social care systems across the globe. Nevertheless, end of life care for people with severe mental illness is a global issue and further studies are required in different socio-economic settings.

Conclusion

This synthesis of case studies indicates that cross disciplinary care provided in the context of established therapeutic relationships and which values the preferences of individuals with severe mental illness experiencing an end-of-life condition is supported in the absence of high-quality intervention research.

Figure 1 PRISMA flow chart

For peer review only - http://bmjopen.bmj.com/site/about/guidelines.xhtml

Figure 2: Histogram of year of publication of case studies

The original protocol for the study (supplementary file 1).

Funding statement:

This work was supported by the National Institute for Health Research (NIHR) Health Services and Delivery Research programme, grant number HS&DR 17/100/15 and will be published in full in *Health Services and Delivery Research* [https://www.journalslibrary.nihr.ac.uk/programmes/hsdr/1710015/#/]

The views expressed are those of the authors and not necessarily those of the NIHR or the Department of Health and Social Care.

A competing interests statement. None declared.

Author Contributions: Conceived and designed the systematic review: MC, DE, SA, PG, MM, AM, BH. Conducted the literature search: MM, DE. Analysed the data: MC, DE, SA, PG, AM, BH. Wrote the paper: MC, DE, SA, PG, MM, AM, BH. Data interpretation and critical revision of manuscript: MC, DE, SA, PG, MM, AM, BH. All authors reviewed and approved the manuscript.

Any checklist and flow diagram for the appropriate reporting statement, (supplementary file 2 for completed PRISMA checklist, supplementary file 5 for JBI checklist,)

Patient consent for publication: Not required.

Data availability statement All data of the current study is present in the main manuscript, figures, tables, and online supplemental material.

For peer review only - http://bmjopen.bmj.com/site/about/guidelines.xhtml

References

1. Vos T, Barber RM, Bell B, et al. Global, regional, and national incidence, prevalence, and years lived with disability for 301 acute and chronic diseases and injuries in 188 countries, 1990–2013: a systematic analysis for the Global Burden of Disease Study 2013. *The Lancet* 2015;386(9995):743-800. doi: https://doi.org/10.1016/S0140-6736(15)60692-4
2. Centre for Mental Health. The economic and social costs of mental health problems in 2009/10. London: Centre for Mental Health, 2010.
3. Friedli L, Parsonage M. Promoting mental health and preventing mental illness: the economic case for investment in Wales. Cardiff: All Wales Mental Health Promotion Network, 2009.
4. Department of Health. Building bridges: A guide to arrangements for interagency working for the care and protection of severely mentally ill people. London: Department of Health, 1995.
5. Kronenberg C, Doran T, Goddard M, et al. Identifying primary care quality indicators for people with serious mental illness: a systematic review. *Br J Gen Pract* 2017;67(661):e519-e30. doi: 10.3399/bjgp17X691721 [published Online First: 2017/07/05]
6. National Institute for Health Research. Forward thinking: NIHR research on support for people with severe mental illness. Southampton: National Institute for Health Research, 2018.
7. Ellison N. Mental health and palliative care. London: Mental Health Foundation 2008.
8. Thornicroft G. Premature death among people with mental illness. *BMJ : British Medical Journal* 2013;346:f2969. doi: 10.1136/bmj.f2969
9. Picot SA, Glaetzer KM, Myhill KJ. Coordinating end of life care for individuals with a mental illness. A nurse practitioner collaboration. *Collegian: Journal of the Royal College of Nursing, Australia* 2015;22(1):143-9.
10. Chesney E, Goodwin GM, Fazel S. Risks of all-cause and suicide mortality in mental disorders: a meta-review. *World Psychiatry* 2014;13(2):153-60. doi: 10.1002/wps.20128 [published Online First: 2014/06/04]
11. Carney CP, Jones L, Woolson RF. Medical comorbidity in women and men with schizophrenia: a population-based controlled study. *J Gen Intern Med* 2006;21(11):1133-7. doi: 10.1111/j.1525-1497.2006.00563.x [published Online First: 2006/10/10]
12. Woods A, Willison K, Kington C, et al. Palliative care for people with severe persistent mental illness: A review of the literature. *Canadian Journal of Psychiatry* 2008;53(11):725-36.
13. Millman J, Galway K, Santin O, et al. Cancer and serious mental illness – patient, caregiver and professional perspectives: Study protocol. *Journal of Advanced Nursing* 2016;72(1):217-26.
14. Knapik GP, Graor CH. Engaging Persons with Severe Persistent Mental Illness into Primary Care. *The Journal for Nurse Practitioners* 2013;9(5):283-87. doi: https://doi.org/10.1016/j.nurpra.2013.02.019
15. Shalev D, Brewster K, Arbuckle MR, et al. A staggered edge: End-of-life care in patients with severe mental illness. *General Hospital Psychiatry* 2017;44:1-3.
16. General Medical Council. Treatment and care towards the end of life: Good practice in decision making. Manchester: General Medical Council, 2010.
17. Howard LM, Barley EA, Davies E, et al. Cancer diagnosis in people with severe mental illness: Practical and ethical issues. *The Lancet Oncology* 2010;11(8):797-804. doi: 10.1016/s1470-2045(10)70085-1
18. Batty GD, Whitley E, Gale CR, et al. Impact of mental health problems on case fatality in male cancer patients. *Br J Cancer* 2012;106(11):1842-5. doi: 10.1038/bjc.2012.150 [published Online First: 2012/04/21]
19. Desai MM, Bruce ML, Kasl SV. The effects of major depression and phobia on stage at diagnosis of breast cancer. *Int J Psychiatry Med* 1999;29(1):29-45. doi: 10.2190/0c63-u15v-5nur-tvxe [published Online First: 1999/06/22]
20. Irwin KE, Henderson DC, Knight HP, et al. Cancer care for individuals with schizophrenia. *Cancer* 2014;120(3):323-34.

For peer review only - http://bmjopen.bmj.com/site/about/guidelines.xhtml

21. Foti ME. Palliative care for patients with serious mental Illness. In: Chochinov HM, Breitbart W, eds. Handbook of Psychiatry in Palliative Medicine. 2nd ed. Oxford: Oxford University Press 2012.
22. Ishikawa H, Yasunaga H, Matsui H, et al. Differences in cancer stage, treatment and in-hospital mortality between patients with and without schizophrenia: Retrospective matched-pair cohort study. *British Journal of Psychiatry* 2016;208(3):239-44. doi: 10.1192/bjp.bp.114.156265
23. Department of Health. A framework for mental health research. London: Department of Health, 2017.
24. Department of Health. Our commitment to you for end of life care the government response to the review of choice in end of life care, 2016.
25. Department of Health SSaPS. Living matters dying matters. A palliative and end of life care strategy for adults in Northern Ireland 2010.
26. Scottish Government. Better cancer care: an action plan. Edinburgh: Scottish Government, 2008.
27. NHS Scotland. Living and dying well. A national action plan for palliative and end of life care in Scotland, 2008.
28. Scottish Government. Improving the health and wellbeing of people with long term conditions in Scotland: A national action plan. Edinburgh: Scottish Government, 2009.
29. Welsh Assembly Government. Designed for life: creating world class health and social care for Wales in the 21st Century. Cardiff: Welsh Assembly Government, 2005.
30. All Wales Palliative Care Planning Group. Report to Minister for health and social services on palliative care services – Sugar Report. Cardiff: Welsh Assembly Government, 2008.
31. All Wales Palliative Care Planning Group. Implementation of palliative care report: Palliative care services funding 2008 to 2009 - the Finlay Report. Cardiff: Welsh Assembly Government, 2009.
32. Welsh Government. Together for health: Delivering end of life care. Cardiff: Welsh Government, 2013.
33. Exley C. Review article: The sociology of dying, death and bereavement. *Sociology of Health and Illness* 2004;26(1):10–22.
34. Payne S. From here to there: reaching out for palliative care. *European Journal of Palliative Care* 2011;18(6):264–5.
35. Hannigan B, Edwards D, Anstey S, et al. End of life care for people with severe mental illness: an evidence synthesis (the MENLOC study) *Health Services and Delivery Research journal* 2021 in press
36. McWhinney IR. The value of case studies,. *The European Journal of General Practice* 2001; 7(3):88-89. doi: 10.3109/13814780109080866
37. Crowe S, Cresswell K, Robertson A, et al. The case study approach. *BMC Medical Research Methodology* 2011;11(1):100. doi: 10.1186/1471-2288-11-100
38. Moher D, Liberati A, Tetzlaff J, et al. Preferred reporting items for systematic reviews and meta-analyses: the PRISMA statement. *Ann Intern Med* 2009;151(4):264-9, w64. doi: 10.7326/0003-4819-151-4-200908180-00135 [published Online First: 2009/07/23]
39. Mahood Q, Van Eerd D, Irvin E. Searching for grey literature for systematic reviews: challenges and benefits. *Res Synth Methods* 2014;5(3):221-34. doi: 10.1002/jrsm.1106 [published Online First: 2015/06/09]
40. Centre for Research Dissemination. CRD's guidance on undertaking reviews in health care. York: University of York CRD, 2009.
41. Thomas J, Harden A. Methods for the thematic synthesis of qualitative research in systematic reviews. *BMC Medical Research Methodology* 2008;8(1):45. doi: 10.1186/1471-2288-8-45
42. Moola S, Munn Z, Tufanaru C, et al. Chapter 7: Systematic reviews of etiology and risk. In: Aromataris E, Munn Z, eds. JBI Manual for Evidence Synthesis. Adelaide: The Joanna Briggs Institute 2020.

For peer review only - http://bmjopen.bmj.com/site/about/guidelines.xhtml

43. Bakker TJ. Palliative care in chronic psycho-geriatrics: A case-study. *Patient Education and Counselling* 2000;41(1):107-13.
44. Feldman DB, Sorocco KH, Bratkovich KL. Treatment of posttraumatic stress disorder at the end-of-life: Application of the Stepwise Psychosocial Palliative Care model. *Palliative & Supportive Care* 2014;12(3):233-43.
45. Feldman DB. Stepwise psychosocial palliative care: A new approach to the treatment of posttraumatic stress disorder at the end of life. *Journal of Social Work in End-of-Life & Palliative Care* 2017;13(2-3):113-33.
46. Mason EN, Bowman EH. Determination of capacity in a critically ill patient with long standing mental illness. 2018 Annual Scientific Meeting: Journal of the American Geriatrics Society, 2018:S97.
47. Rodriguez-Mayoral O. Bipolar disorder and cancer. Management at end of life. Case report. *Bipolar Disorders* 2018;Conference(Supplement 1):79.
48. Kennedy P, Sarafi C, Greenish W. Homelessness and end of life care – Practical information and tools to support the needs of homeless people who are approaching the end of life, and those who are bereaved. London: St Mungos and Marie Curie, 2013.
49. Maloney K. Opinion: My husband refused to believe he had bipolar disorder, and denial destroyed him. TheJournalie. Ireland, 2014.
50. Badger JM, Ekham LR. Conflicting voices: Withhold treatment or not for a patient with chronic self-destructive behavior? *JONA'S Healthcare Law, Ethics and Regulation* 2011;13(3):79-83.
51. Boyd C, Cantone J, Gambrino P, et al. When the hospice patient is manipulative. *Hospice Journal* 1997;12(1):1-16.
52. Candilis PJ, Foti ME. Case presentation: End-of-life care and mental illness: The case of Ms. W. *Journal of Pain and Symptom Management* 1999;18(6):447-8.
53. Feely MA, Havyer RDA, Lapid MI, et al. Management of end-of-life care and of difficult behaviors associated with borderline personality disorder. *Journal of Pain and Symptom Management* 2013;45(5):934-8.
54. Feldman D, Periyakoil V. Posttraumatic stress disorder at the end of life. *Journal of palliative medicine* 2006;9 1:213-8.
55. Geppert CMA, Rabjohn P, Vlaskovits J. To treat or not to treat: Psychosis, palliative care, and ethics at the end-of-life: A case analysis. *Psychosomatics* 2011;52(2):178-84.
56. Gonzalez MR, Marcelo CD, Stringer M. Hospice care for 86 year old male with recurrent breast cancer: A case report. *Cases Journal* 2009;2(6):8357.
57. Griffith LJ. Good palliative care for a patient with schizophrenia dying of emphysema and heart failure. *Psychiatry* 2007;4(8):61-5.
58. Hill R. End-of-life care for the patient with borderline personality disorder. *Journal of Hospital and Palliative Nursing* 2005;7(3):150-63.
59. Kunkel EJ, Woods CM, Rodgers C, et al. Consultations for 'maladaptive denial of illness' in patients with cancer: psychiatric disorders that result in noncompliance. *Psycho-Oncology* 1997;6(2):139-49.
60. Levin SM, Feldman MB. Terminal illness in a psychiatric patient--issues and ethics. *South African Medical Journal* 1983;63(13):492-4.
61. Lopez A, Yager J, Feinstein RE. Medical futility and psychiatry: Palliative care and hospice care as a last resort in the treatment of refractory anorexia nervosa. *International Journal of Eating Disorders* 2010;43(4):372-7.
62. McCasland LA. Providing hospice and palliative care to the seriously and persistently mentally ill. *Journal of Hospice and Palliative Nursing* 2007;9(6):305-13.
63. McKenna RC, Bailey L, Haake J, et al. Clozapine and chemotherapy. *Hospital & Community Psychiatry* 1994;45(8):831.
64. Moini B, Levenson JL. A forgotten diagnosis: simple schizophrenia in a patient with breast cancer. *Psychosomatics* 2009;50(1):87-9.

For peer review only - http://bmjopen.bmj.com/site/about/guidelines.xhtml

65. Monga V. Clozapine and concomitant chemotherapy in a patient with schizophrenia and new onset esophageal cancer. *Psycho-Oncology* 2015;24(8):971-2.
66. Rice TR, Dobry Y, Novakovic V, et al. The importance of patient-provider communication in end-of-life care. *Journal of Bioethical Inquiry* 2012;9(4):439-41. doi: 10.1007/s11673-012-9397-4
67. Romm S, Combs H, Poeschla B. Psychiatry and palliative care: Thoughts on collaboration. *Journal of Hospital and Palliative Nursing* 2009;11(2):79-81.
68. Shah HA, Lee HBH, Nunery WR. Neglected basal cell carcinoma in a schizophrenic patient. *Ophthalmic Plastic & Reconstructive Surgery* 2008;24(6):495-7.
69. Stecker E. The terminally ill patient on an acute psychiatric unit. A case study. *Journal of Psychosocial Nursing and Mental Health Services* 1993;31(5):13-5.
70. Steves F, Williams T. Enhancing end-of-life care for terminally ill psychiatric patients. *Nursing* 2016;46(8):54-8.
71. Terpstra TL, Williamson S, ., Terpstra T. Palliative care for terminally ill individuals with schizophrenia. *Journal of Psychosocial Nursing and Mental Health Services* 2014;52(8):32-8.
72. Thomson K, Henry B. Oncology clinical challenges: Caring for patients with preexisting psychiatric illness. *Clinical Journal of Oncology Nursing* 2012;16(5):471-80.
73. Mogg A, Bartlett A. Refusal of treatment in a patient with fluctuating capacity - Theory and practice. *Journal of Forensic Psychiatry and Psychology* 2005;16(1):60-9.
74. Muhtaseb M, Olver JM, Constantine S. Massive basal cell carcinoma in a schizophrenic patient: Treatment options and constraints. *British Journal of Ophthalmology* 2001;85(1):117-8.
75. O'Neill J, Crowther T, Sampson G. A case study: anorexia nervosa. Palliative care of terminal psychiatric disease. *Am J Hosp Palliat Care* 1994;11(6):36-8. doi: 10.1177/104990919401100609 [published Online First: 1994/11/01]
76. Clements-Cortes A. The use of music in facilitating emotional expression in the terminally ill. *American Journal of Hospice & Palliative Medicine* 2004;21(4):255-60.
77. Kadri A, Blackmer J, Ibrahim M. Obtaining consent to a life-sustaining treatment for a patient with a major psychiatric illness. *CMAJ* 2014;186(14):1087-9.
78. Webber T. End of life care for people with mental illness. *Journal of Ethics in Mental Health* 2012;7:1-4.
79. Cabaret W, Krerbi Y, Saravane D. Palliative care in psychiatric institutions. *European Journal of Palliative Care* 2002;9(4):150-2.
80. Doron A, Mendlovic S. Dying in the psychiatric ward. *Israel Journal of Psychiatry and Related Sciences* 2008;45(2):141-4.
81. Ang A, Loke PCW, Campbell AV, et al. Live or let die: Ethical issues in a psychiatric patient with end-stage renal failure. *Annals of the Academy of Medicine Singapore* 2009;38(4):370-73.
82. Griffith LJ. Brief supportive psychotherapy for a patient with chronic schizophrenia who is dying. *Psychiatry* 2007;4(12):49-54.
83. Hannigan B, Evans N. Critical junctures in health and social care: Service user experiences, work and system connections. *Social Theory & Health* 2013;11(4):428-44. doi: 10.1057/sth.2013.16
84. Love AW, Liversage LM. Barriers to accessing palliative care: A review of the literature. *Progress in Palliative Care* 2014;22(1):9-19. doi: 10.1179/1743291X13Y.0000000055
85. Wilson R, Hepgul N, Higginson IJ, et al. End-of-life care and place of death in adults with serious mental illness: A systematic review and narrative synthesis. *Palliat Med* 202034(1), 49–68;34(1):49-68. doi: 10.1177/0269216319867847 [published Online First: 2019/10/15]
86. Edwards D, Anstey S, Coffey M, et al. End of life care for people with severe mental illness: mixed methods systematic review and thematic synthesis (the Menloc study). *Palliative Medicine* 2021 doi: doi:10.1177/02692163211037480
87. Donald EE, Stajduhar KI. A scoping review of palliative care for persons with severe persistent mental illness. *Palliat Support Care* 2019;17(4):479-87. doi: 10.1017/S1478951519000087 [published Online First: 2019/03/20]

For peer review only - http://bmjopen.bmj.com/site/about/guidelines.xhtml

88. Elie D, Marino A, Torres-Platas SG, et al. End-of-life care preferences in patients with severe and persistent mental illness and chronic medical conditions: A comparative cross-sectional study. *American Journal of Geriatric Psychiatry* 2018;26(1):89-97. doi: 10.1016/j.jagp.2017.09.018

For peer review only

For peer review only - http://bmjopen.bmj.com/site/about/guidelines.xhtml

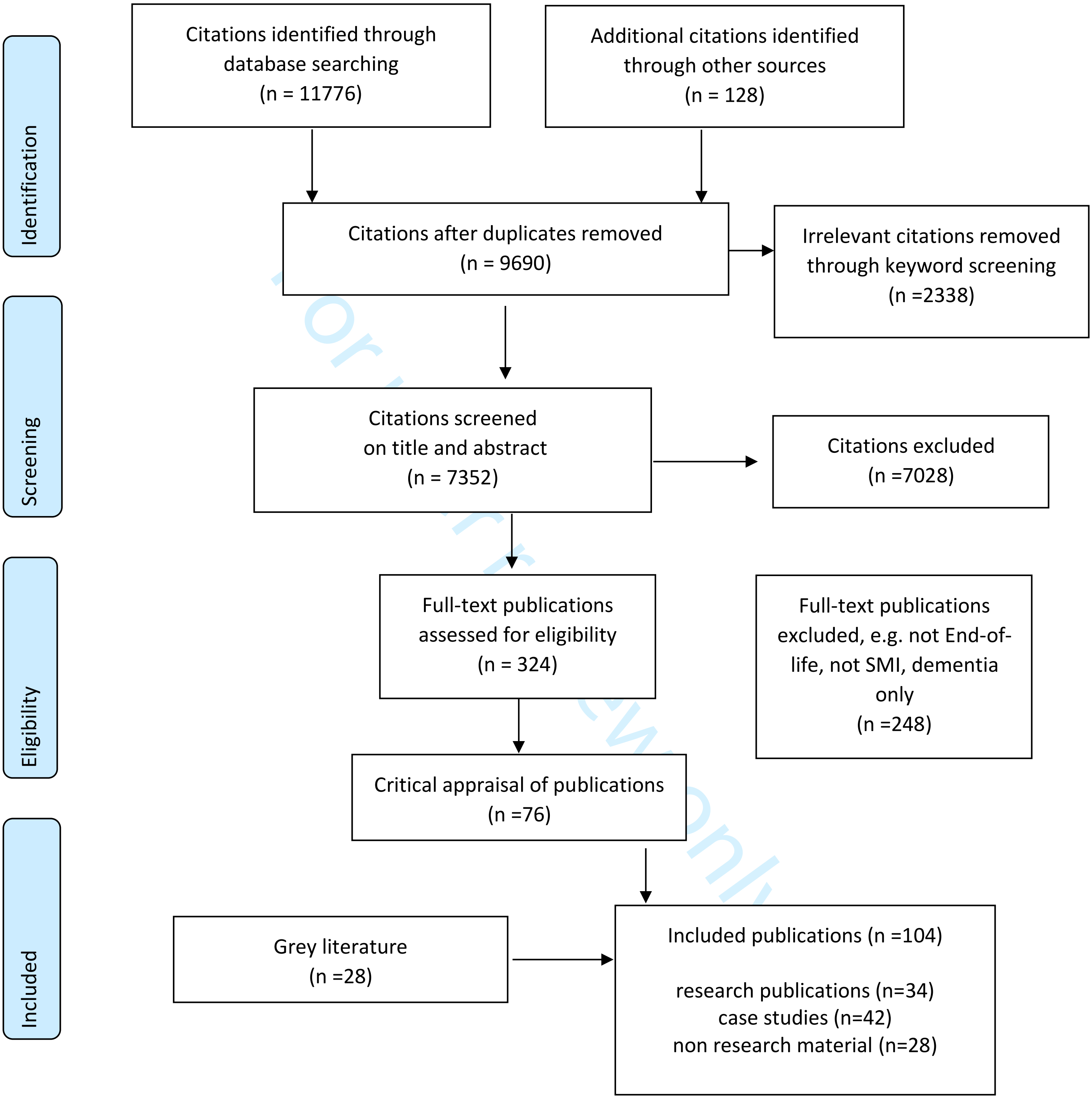

Figure 1: PRISMA Flow chart

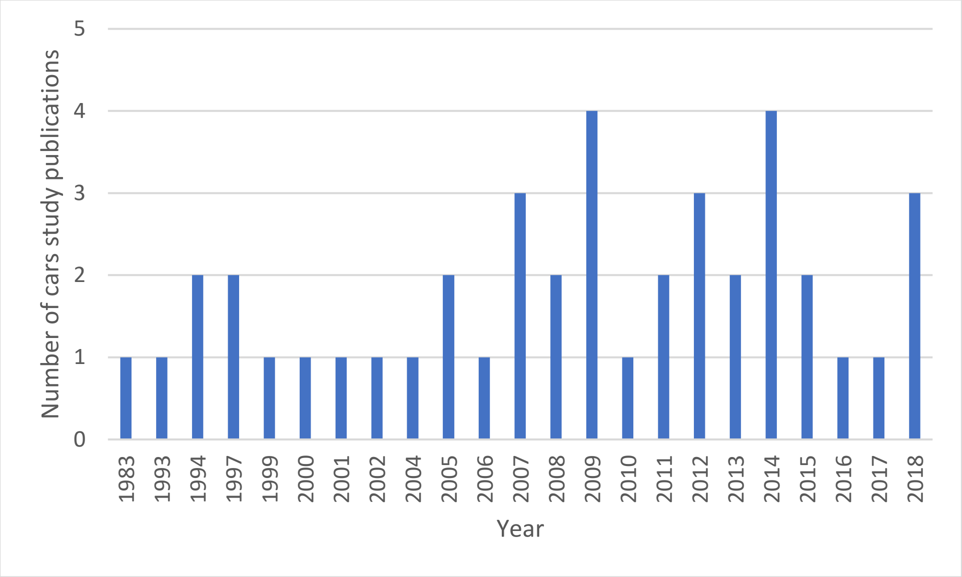

Figure 2: Histogram of year of publication of case studies

For peer review only

National Institute for Health Research (NIHR)
Health Services and Delivery Research (HS&DR) Programme

Protocol

Project title:	End of life care for people with severe mental illness: an evidence synthesis (the MENLOC study)
Project reference:	HS&DR Project 17/100/15
Version:	1.0
Date:	24 September 2018
Chief investigator:	Professor Ben Hannigan
Research team:	Dr Sally Anstey, Dr Paul Gill, Deborah Edwards, Mala Mann, Professor Michael Coffey, Alan Meudell, Roger Pratt
REC approval:	Not applicable
Start date:	1 November 2018
End date:	31 January 2020

Funding acknowledgement: This project is funded by the NIHR HS&DR Programme (ref 17/100/15)

Department of Health and Social Care disclaimer: The views expressed are those of the authors and not necessarily those of the NHS, the NIHR or the Department of Health and Social Care

Protocol version control:

Version	Date	Details
1.0	24.09.2018	Protocol reflecting full project description approved by HS&DR Board

For peer review only - http://bmjopen.bmj.com/site/about/guidelines.xhtml

HS&DR Project 17/100/15: End of life care for people with severe mental illness: an evidence synthesis (the MENLOC study)

FULL TITLE OF PROJECT

End of life care for people with severe mental illness: an evidence synthesis (the MENLOC study)

SUMMARY OF RESEARCH

The aim of this project is to synthesise relevant research and other appropriate evidence relating to the organisation, provision and receipt of end of life care for people with severe mental illness (including schizophrenia, bipolar disorder and other psychoses, major depression and personality disorder) who have an additional diagnosis of advanced, incurable, cancer and/or end-stage lung, heart, renal or liver failure and who are likely to die within the next 12 months. Outputs from the project will be tailored to stakeholders, and clear implications will be drawn for the future commissioning, organisation, management and provision of clinical care. Recommendations will be made for future data-generating studies designed to inform service and practice improvements, guidance and policy.

In this context, summary objectives are to:

1. locate, appraise and synthesise relevant research;
2. locate and synthesise policy, guidance, case reports and other grey and non-research literature;
3. produce outputs with clear implications for service commissioning, organisation and provision;
4. make recommendations for future research designed to inform service improvements, guidance and policy.

This review will be conducted according to the guidance developed by the Centre for Reviews and Dissemination (CRD) and will be reported following the Preferred Reporting Items for Systematic Reviews and Meta-Analyses (PRISMA) statement recommendations. Reflecting Evidence for Policy and Practice Information (EPPI) Centre principles, opportunities will also be embedded into the project to maximise stakeholder engagement for the purposes of both shaping its focus and maximising its reach and impact.

Searches will be developed initially using Medical Subject Headings (MeSH) and text words across health, social care and psychology databases from their inception. In consultation with a stakeholder advisory group, supplementary methods will be developed to identify additional material including policies, reports, expert opinion pieces and case studies. All English language items relating to the provision and receipt of end of life care for people with severe mental illness and an additional diagnosis of advanced, incurable, cancer and/or end-stage lung, heart, renal or liver failure will be included. All included citations will be assessed for quality using tools developed by the Critical Appraisal Skills Programme (CASP), or alternatives as necessary if suitable CASP tools are not available. Data will be extracted into tables, and subjected to meta-analyses where possible or thematic synthesis with help from NVivo. Strength of synthesised findings will be reported where possible using GRADE and CerQual.

Information derived from the processes described above will be drawn on in an accessibly written summary. Uniquely, this synthesis will comprehensively bring together evidence on factors facilitating and hindering high-quality end of life care for people with severe mental illness, who have an additional diagnosis of advanced, incurable, cancer and/or end-stage lung, heart, renal or liver failure, and evidence relating to services, processes, interventions, views and experiences. Implications will be stated for the improvement of relevant NHS and third sector care and recommendations will be made for future research.

BACKGROUND AND RATIONALE

We plan a rigorous synthesis of research and other evidence conducted according to internationally agreed quality standards. The project is within the HS&DR Programme’s remit in addressing quality, organisation and access in health services. In the context of calls for parity of esteem between mental health and physical health care (Mental Health Taskforce, 2016) the health problem this project addresses is a highly relevant, but also neglected, one.

For peer review only - http://bmjopen.bmj.com/site/about/guidelines.xhtml

HS&DR Project 17/100/15: End of life care for people with severe mental illness: an evidence synthesis (the MENLOC study)

In preparing this proposal an initial scoping review was undertaken (with updated searches run in July 2018), and a targeted search was made for relevant policy documents across the four UK nations. A search of the database of NIHR projects was conducted to check for overlapping or related studies. Searches of four databases (combining 'palliative care', 'mental health' and 'service provision' terms: see 'Search strategy' below) produced 4,754 citations, contained within which are numbers of relevant papers including two previous literature reviews (from the UK and Canada respectively), both now out-of-date having been published a decade ago (Ellison, 2008, Woods et al., 2008). Items discovered in this scoping search confirm the timeliness and feasibility of a new, rigorous, evidence synthesis: and particularly an EPPI-Centre style review which is sensitive to the needs of stakeholders and which includes grey and non-research materials (Gough et al., 2017). Items from this initial search, combined with general material addressing what is known about the burden of disease and the physical health of people living with mental health difficulties, have been used to inform this background and rationale (and subsequent) sections of this project proposal.

Burden of disease and costs

The overarching background for this project includes what is already known about the burden of severe mental illness, cancer and end-stage lung, heart, renal or liver failure. Mental ill-health is a leading cause of years lived with disability (YLD) around the world, with major depressive disorder the leading cause of YLD in 56 countries and the second leading cause in a further 56 (Global Burden of Disease Study 2013 Collaborators, 2015). Specific cancers, along with mental health, neurological and drug use disorders and specific organ diseases all feature in the leading 20 causes of disability adjusted life years (DALYs) in England for 2013 (Newton et al., 2015). The wider economic costs of mental illness in England were recently estimated at £105.2 billion each year (Centre for Mental Health, 2010). This figure combines the direct costs of services, lost productivity at work and reduced quality of life with the annual costs of the same in Wales estimated at £7.2 billion (Friedli and Parsonage, 2009).

Meeting the physical health needs of people living with severe mental illness

The term 'severe [or, often used interchangeably, 'serious' or 'serious and enduring'] mental illness' (SMI) as used throughout this project proposal has longstanding currency within the fields of mental health policy, services and practice dating back at least as far as the publication of *Building Bridges* (Department of Health, 1995). It continues to be used in research (see for example: Kronenberg et al., 2017), and has currency with the NIHR Dissemination Centre which published a Themed Review into Severe Mental Illness in 2018 (National Institute for Health Research, 2017). *Building Bridges* recognised the imprecision of the term 'severe mental illness', and endorsed a multidimensional framework definition encompassing five areas: safety; need for informal or formal care; disability; diagnosis; and duration. Diagnosis is therefore an important, but not the only, dimension used in the identification of people with severe mental illness and includes ICD-10 diagnoses of schizophrenia, schizotypal and delusional disorders, bipolar affective disorder, and disorders of adult personality and behaviour (World Health Organization, 1992) along with similar DSM-5 diagnoses including schizophrenia spectrum and other psychotic disorders, bipolar and related disorders, and major depressive disorder (American Psychiatric Association, 2014).

People with severe mental illness have high co-morbidities (Ellison, 2008), and experience higher mortality rates and reduced life expectancy compared to the general population (Taylor et al., 2012, Thornicroft, 2013). Higher mortality and morbidity rates for this group have been found across all age groups (Picot et al., 2015), with a 10-20 year reduction in life expectancy reported (Chesney et al., 2014). Inequities can be explained with reference to individual and system-level factors. People with severe mental illness are less likely to attend health screenings and may respond to symptoms differently (Disability Rights Commission, 2006, Carney et al., 2006). They may delay or avoid help, and are more likely to exhibit noncompliant and disruptive behaviours (Woods et al., 2008, Millman et al., 2016) putting them at risk of delayed disease detection (Ellison, 2008). Inadequate support systems are also common among those with severe mental illness, affecting their ability to access appropriate clinical care and navigate complex health systems (Knapik and Graor, 2013). Other factors influencing variations in mortality and morbidity for people living with severe mental illness include poor previous experiences of seeking help from health care professionals, the incorrect

For peer review only - http://bmjopen.bmj.com/site/about/guidelines.xhtml

attribution of physical symptoms to psychiatric disorder by workers and lack of experience by mental health professionals in determining how and when to refer onwards to other appropriate services (Woods et al., 2008, Shalev et al., 2017).

End of life care for people with severe mental illness

In this study end of life care is used to refer to the care of people with diagnoses of advanced, incurable, cancer and/or end-stage lung, heart, renal or liver failure and who are likely to die within the next 12 months. It includes care provided in hospitals, hospices and other institutional settings (such as prisons and hostels) and care provided in the home and via outreach to people who may also be homeless.

Beyond the inequities identified above, commitments to parity of esteem demand that end of life care for people with severe illness should be as timely and as high-quality as it is for others. However, evidence suggests that this group is poorly served, with England's Cancer Strategy 2015-2020 (Independent Cancer Taskforce, 2015) recognising people with severe mental illness as needing a targeted focus to improve diagnosis and care. Although cancer incidence amongst people living with chronic mental health difficulties is similar to that of the general population, mortality rates are double (Howard et al., 2010, Batty et al., 2012). This disparity may be related in part to late presentation and reduced use of interventions such as surgery, chemotherapy or radiotherapy (Desai et al., 1999, Irwin et al., 2014). The experience of severe mental illness can delay detection and treatment of life-threatening physical disorders as people are less likely to seek treatment, to verbalise pain and to access timely healthcare (Foti, 2012). Consequently, this patient cohort is more likely to present with more advanced cancers which are invariably more complex and costly to treat, with patients less likely to undergo invasive treatments and more likely to die (Ishikawa et al., 2016). Some cancers, other terminal conditions and/or related treatments may also compound mental illness and precipitate potential 'problematic' behaviours (McCormack and Sharp, 2006). For many patients, palliative care is therefore often the only meaningful treatment option available.

Once in touch with end of life services the symptoms of people with severe mental illness may be poorly recognised and undertreated, with staff working in end of life services lacking knowledge, training and experience in this area (Addington-Hall, 2000). Undetected and hence untreated mental illness can jeopardise treatment outcomes, reduce patient satisfaction and increase health care costs (Zabora et al., 2000). Variable adherence can be a complicating factor (Knapik and Graor, 2013), compounded by comorbid disorders such as substance misuse and social factors such as homelessness, isolation or lack of transportation all of which can exert an impact on care planning for end of life care and treatment (Woods et al., 2008, Picot et al., 2015). Assumptions about the capacity of people with severe mental illness to make end of life decisions, and concerns that end of life discussions would be too distressing or exacerbate mental health problems, may lead to inadequate consultation (Morgan, 2016). This is reflected in the fact that people with severe mental illness have a higher percentage of 'do not resuscitate' orders than other groups and are less likely to have had discussions about their explicit wishes for end of life care (Warren et al., 2014).

Palliative care

Whilst not all people at the end of life need palliative care an NAO survey (Dixon et al., 2015) confirmed inequities in palliative care provision for both cancer and non-cancer patient populations. In Wales, an estimated 24,000 of the 32,000 people dying would benefit from palliative care but over a quarter do not have access to it (Hughes-Hallet et al., 2011, Dixon et al., 2015). Access to palliative care services for people who die from cancer is 46% compared to 5% from those dying from other conditions (Marie Curie and the Bevan Foundation, 2014), including end-stage lung, heart, renal or liver failure.

People with mental illness and advanced incurable cancer and/or lung, heart, renal or liver failure face inequities and discrimination (Hughes-Hallet et al., 2011) and a lack of integrated care (Ellison, 2008). Some may be excluded from end of life care planning (Woods et al., 2008), and even from hospitals or hospices entirely (McCormack and Sharp, 2006). They may be referred back to mental health services, where staff are largely inadequately prepared to provide appropriate end of life care (Woods

For peer review only - http://bmjopen.bmj.com/site/about/guidelines.xhtml

et al., 2008). Difficulties accessing appropriate services mean that for people with mental illness and life-threatening disease palliative care may be the first line of treatment (Ellison, 2008). The specific provision of palliative care for people with severe mental illness is known to be poorer than for the general population. People with severe mental illness are approximately 50% less likely to access appropriate palliative care, including symptom control and pain relief (Woods et al., 2008, Shalev et al., 2017). Palliative care and hospice staff often feel unskilled (Shalev et al., 2017), and lack confidence and training in conducting discussions about end-of-life care with people severe mental illness (Morgan, 2016). Existing evidence also indicates that there is a lack of coordinated end of life care and access to appropriate psychosocial support is often limited (Ellison, 2008, Woods et al., 2008). Medical and nursing staff working in hospices have also been shown to be unprepared for working with people with severe mental illness, basing their assessments on instinct rather than using evidence-based approaches (Hackett and Gaitan, 2007). In a survey of psychological services in hospices in the UK and Republic of Ireland only 30% of hospices had access to a psychiatrist, whilst 41% had access to a clinical psychologist and 45% had neither (Price et al., 2006). Patient experience data underscores these observations, with England's National Cancer Patient Experience Survey showing that people with a long-term mental health condition (2% of those surveyed, n=1184) reporting less positive experiences of cancer care (Department of Health, 2010). In Wales, the most recent National Cancer Patient Experience Survey found that the lowest proportion of respondents reporting positive experiences of their cancer care were those identifying as also having mental health problems (Picker Institute Europe, 2017).

EVIDENCE EXPLAINING WHY THIS RESEARCH IS NEEDED NOW

Research at the interface of physical and mental health is recognised as a UK priority (Department of Health, 2017). Against the background presented above, this project will create generalisable knowledge to improve end of life care and services for an underserved group. Policies from the four UK governments focus on improving end of life care, where diagnosis is immaterial (Department of Health, 2016, Northern Ireland Executive, 2010, Scottish Government, 2008b, Scottish Government, 2008a, Scottish Government, 2009, Welsh Assembly Government, 2005, All Wales Palliative Care Planning Group, 2008, All Wales Palliative Care Planning Group, 2009, Welsh Government, 2013). These require the introduction of palliative and supportive care earlier in the illness trajectory, with patient surveys showing that this is rated very highly by those receiving it (Drakeford, 2014). Marie Curie identified triggers that should initiate palliative care for people with diagnoses other than cancer (Marie Curie, 2015), but apart from dementia they do not mention those with pre-existing severe mental illness. In national policy the particular needs of people with severe mental illness who develop advanced incurable cancer and/or end-stage lung, heart, renal or liver failure are acknowledged poorly, or not at all. This group face the prospect of 'disadvantaged dying' (Exley, 2004, Payne, 2011), at a time when quality of care in the last months of life should be uniformly high for all groups.

Uniquely amongst the cancer, palliative and end of life strategies developed across the four countries of the UK, England's Cancer Strategy 2015-2020 makes the specific recommendation that the NIHR commission research in the area of cancer care for people living with severe mental illness (Independent Cancer Taskforce, 2015). This project responds to this call, and expands it to also cover end of life care for people living with severe mental illness and facing end-stage lung, heart, renal or liver failure. It will answer a question which is both timely and relevant: what evidence is there relating to the organisation, provision and receipt of care for people with severe mental illness who have an additional diagnosis of advanced, incurable, cancer and/or end-stage lung, heart, renal or liver failure and who are likely to die within the next 12 months?

Our rigorously conducted evidence synthesis will bring together reports of approaches to service organisation, processes and interventions shown to both facilitate and hinder the provision of high-quality, accessible, equitable and acceptable end of life care to people with severe mental illness. We will also gather research and other evidence reporting the views and experiences of service users, families and health and social care staff. The project will have an impact on services and practice by presenting its findings in accessible ways to NHS and other managers, practitioners and educators. We anticipate findings will inform future NICE guidelines, and thereby help shape the provision of services. Current, relevant, NICE guidance (National Institute for Clinical Excellence, 2004, National Institute for Health and Care Excellence, 2009, National Institute for Health and Care Excellence, 2011, National Institute for Health and Care Excellence, 2014b, National Institute for Health and Care

For peer review only - http://bmjopen.bmj.com/site/about/guidelines.xhtml

Excellence, 2014a) lacks standards or recommendations particularly addressing end of life care for people with severe mental illness, with documents supporting in-progress guideline development (National Institute for Health and Care Excellence, 2018 [anticipated]) also lacking a focus in this area. We anticipate findings from this study will help redress this, informing future guidance and thereby shaping the provision of services.

A search of NIHR databases finds studies which have investigated: the physical health of people with severe mental illness (e.g., HTA 12/28/05); services for this group across organisations (e.g., HS&DR 11/1023/13); and care for people experiencing mental health difficulties after receiving cancer diagnosis (e.g., HTA 09/33/02). As no research has been commissioned in the area we propose here, this project effectively begins a new and important field of work with value to the NHS and its partners. Using a methodical, systematic and transparent approach our team plans to use this study as the starting point for a programme of research which builds on the existing literature, is designed with people who have experience of mental health difficulties and with people who have lived with cancer and have cared for family at the end of life, and which generates new evidence of what works with value to the NHS and other relevant organisations. Further studies planned include data generating investigations into the provision and receipt of end of life care for people with severe mental illness.

AIMS AND OBJECTIVES

The aim of this project is to synthesise relevant research and other appropriate evidence relating to the organisation, provision and receipt of end of life care for people with severe mental illness (including schizophrenia, bipolar disorder and other psychoses, major depression and personality disorder). Specifically, it will answer the question, 'what evidence is there relating to the organisation, provision and receipt of care for people with severe mental illness who have an additional diagnosis of advanced, incurable, cancer and/or end-stage lung, heart, renal or liver failure and who are likely to die within the next 12 months?

Detailed objectives are to:

1. use internationally recognised, transparent, literature review approaches to locate, appraise and synthesise the relevant research evidence relating to the organisation, provision and receipt of care in the expected last year of life for people with severe mental illness who have additional diagnoses of advanced, incurable, cancer and/or end-stage lung, heart, renal or liver failure and who are likely to die within the next 12 months;
2. locate and synthesise policy, guidance, case reports and other grey and non-research literature relating to the organisation, provision and receipt of care in the expected last year of life for people with severe mental illness who have additional diagnoses of advanced, incurable, cancer and/or end-stage lung, heart, renal or liver failure and who are likely to die within the next 12 months;
3. produce outputs with clear implications for service commissioning, organisation and provision;
4. make recommendations for future research designed to inform service improvements, guidance and policy.

RESEARCH PLAN/METHODS

Quality and standards

The protocol for this evidence synthesis will be registered with the International Prospective Register of Systematic Reviews (PROSPERO) (PROSPERO: International prospective register of systematic reviews, undated). It will be conducted with the involvement of colleagues from the Cardiff Specialist Unit for Review Evidence (SURE). It will follow guidance for undertaking reviews in health care published by the Centre for Reviews and Dissemination (CRD) (2009) and will use methods informed by the Evidence for Policy and Practice Information (EPPI) Centre (Gough et al., 2017). To ensure rigour the review will be reported following the Preferred Reporting Items for Systematic Reviews and Meta-Analyses (PRISMA) statement (Moher et al., 2009). Findings will be accessibly presented, with the help of a stakeholder advisory group and the NIHR Dissemination Centre, to ensure reach. Factors facilitating and hindering high-quality end of life care for people with severe mental illness will

For peer review only - http://bmjopen.bmj.com/site/about/guidelines.xhtml

be identified, along with evidence relating to services, processes, interventions, views and experiences.

Search strategy

The search strategy will be comprehensive and designed to ensure that all relevant literature is obtained. While some terminology is equivocal in this area, the search strategy will be designed to identify the relevant evidence relating to end of life care (i.e., in the last year of life) in those with severe mental illness. Reflecting the importance of diagnosis in the framework definition of severe mental illness, diagnostic terms to be included in the search strategy include: schizophrenia, schizophrenia spectrum and other psychotic disorders, schizotypal and delusional disorders, bipolar affective disorder, bipolar and related disorders, major depressive disorder and disorders of adult personality and behaviour. Reflecting prevailing definitions of severe mental illness (for example, as used by the NIHR Dissemination Centre) searches will not be made for studies into mental and behavioural disorders due to psychoactive substance use or for studies into dementia or other neurodegenerative diseases, and items in these areas will not be included in the review except where participants' diagnoses coexist with the disorders included above. To ensure appropriateness of evidence, inclusion/exclusion criteria have been developed (Table 1):

Table 1: Inclusion/exclusion criteria

Inclusion	Exclusion
Relevant evidence specifically relating to adult participants (>18 years of age) with severe mental illness (including schizophrenia, schizophrenia spectrum and other psychotic disorders, schizotypal and delusional disorders, bipolar affective disorder, bipolar and related disorders, major depressive disorder and disorders of adult personality and behaviour) who have an additional diagnosis of advanced, incurable, cancer and/or end-stage lung, heart, renal or liver failure and who are likely to die within the next 12 months.<br><br>Published in English Language only.	Where reporting allows the distinction to be made, mental health problems (e.g. depression) as a consequence of terminal illness (e.g., cancer or chronic organ failure).<br><br>Evidence relating to end of life care for people with mental and behavioural disorders due to psychoactive substance use, except where these coexist with disorders included in the column to the left.<br><br>Evidence relating to end of life care for people with dementia or other neurodegenerative diseases except where these coexist with disorders included in the column to the left.<br><br>Evidence from animal studies.

Comprehensive searches will be conducted across multiple databases from their inception, including: Ovid Medline; Ovid Allied and Complementary Medicine Database (AMED); Proquest Applied Social Sciences Index and Abstracts (ASSIA); Ebsco Cumulative Index of Nursing and Allied Health Literature (CINAHL); Wiley Cochrane Central Register of Controlled Trials; Database of Abstracts of Reviews of Effectiveness; Ovid Embase; Ovid Health Management Information Centre (HMIC); Ovid MEDLINE Epub Ahead of Print and In-Process and Other Non-Indexed Citations; Ovid PsycInfo; Web of Knowledge Science Citation Indexes. A preliminary search will be developed in Ovid Medline using MeSH and text words as piloted in our initial scoping (see 'Background and Rationale' above, and example of search strategy below), then translated to other databases.

Supplementary searches will be undertaken to identify additional papers, information on studies in progress, unpublished research or research reported in the grey literature. Relevant websites and trial registers will be searched (e.g., NIH ClinicalTrials.gov (http://www.clinicaltrials.gov), metaRegister of Controlled Trials (http://www.controlled-trials.com), WHO International Clinical Trials Registry Platform (ICTRP) (http://www.who.int/ictrp/en). To identify published resources that have not yet been catalogued in the electronic databases, recent editions of key journals will be hand-searched.. Reference lists of included studies will be scanned, experts contacted, and forward citation tracking performed using ISI Web of Science.

For peer review only - http://bmjopen.bmj.com/site/about/guidelines.xhtml

HS&DR Project 17/100/15: End of life care for people with severe mental illness: an evidence synthesis (the MENLOC study)

Reflecting EPPI Centre (Gough et al., 2017) methods, searches will be conducted for non-research material (e.g., social media, personal accounts, policies) using transparent, clearly described, approaches (Mahood et al., 2014). With advice being taken from members of a project stakeholder advisory group, relevant websites will be searched using search terms and strategies carefully tested and refined as necessary to ensure that all items relevant to the care of people with severe mental illness and (a) advanced, incurable, cancer and/or (b) end-stage lung, heart, renal or liver failure and who are likely to die within the next 12 months are equally included. Candidate websites for searching include: the Care Quality Commission (https://www.cqc.org.uk/), Cancer Research UK (http://www.cancerresearchuk.org/), Cancer Research Wales (https://www.cancerresearchwales.co.uk/), Tenovus Cancer Care (http://www.tenovuscancercare.org.uk/), Mental Health Foundation (https://www.mentalhealth.org.uk/), Centre for Mental Health (https://www.centreformentalhealth.org.uk/), St Mungo's (https://www.mungos.org/), National Kidney Foundation (https://www.kidney.org/), British Liver Trust (https://www.britishlivertrust.org.uk/), British Renal Society (http://britishrenal.org/), The Renal Association (https://renal.org/), British Heart Foundation (https://www.bhf.org.uk/), British Lung Foundation (https://www.blf.org.uk/), National Council for Palliative Care (http://www.ncpc.org.uk/), Hospice UK (http://www.hospiceuk.org/), Marie Curie (https://www.mariecurie.org.uk/), Macmillan Cancer Support (http://www.macmillan.org.uk/) and the Scottish Partnership agency for Palliative Care (https://www.palliativecarescotland.org.uk/).

Preliminary database searching using some of the methods described here has already been carried out, as part of an initial scoping undertaken in preparation of this proposal (see below). The initial search strategy once the project commences will build on this, and it is anticipated that the following MeSH terms and keywords will be used, in various combinations, to inform the systematic search process:

- Palliative care
- Hospice care
- Terminal care
- Terminally ill
- End of life care
- Last year of life
- Neoplasms
- Cancer
- Multiple organ failure
- Mental health
- Depression
- Mental disorders
- Depressive disorder
- Personality disorders
- Bipolar disorder
- Schizophrenia
- Mental illness

In July 2018 we took the opportunity to update and extend our scoping exercise (using terms and keywords reproduced above) using four databases: Ovid MEDLINE; Ovid MEDLINE Epub Ahead of Print, In-Process and Other Non-Indexed Citations and Ovid MEDLINE without Revisions; PsycINFO; and EMBASE. To illustrate this process we reproduce below the search run in MEDLINE, which produced 1,387 citations (before the removal of duplicates) for screening:

Database: Ovid MEDLINE(R) ALL <1946 to July 05, 2018>
Search Strategy:

----

1 exp Palliative care/ (48615)
2 exp Hospice care/ (5807)
3 exp Terminal Care/ (47347)
4 exp Terminally ill/ (6150)
5 ("palliative care" or "hospice care" or "end of life care" or end-of-life).tw. (38005)

For peer review only - http://bmjopen.bmj.com/site/about/guidelines.xhtml

HS&DR Project 17/100/15: End of life care for people with severe mental illness: an evidence synthesis (the MENLOC study)

6 ((hospice or terminal*) adj3 (care or caring or ill*)).tw. (12527)
7 ("last year of life" or LYOL or "end of life" or "end of their lives").tw. (19101)
8 (end-stage disease* or end stage disease* or end-stage ill* or end stage ill*).tw. (1112)
9 or/1-8 (104642)
10 exp Neoplasms/ (3055654)
11 (cancer* or tumo?r* or neoplas* or malignan* or carcinoma* or adenocarcinoma* or adeno?carcinoma* or choriocrcinoma* or leukemia* or leukaemia* or metastat* or sarcoma* or teratoma* or lymphoma).tw. (3168623)
12 exp Multiple Organ Failure/ (10043)
13 Organ failure.tw. (16597)
14 Pulmonary Disease, Chronic Obstructive/ (32992)
15 (Chronic obstructive pulmonary disease or COPD).tw. (53007)
16 Heart Failure/ (105052)
17 Renal Insufficiency/ (14550)
18 Liver Failure/ (6735)
19 ("cardiac failure" or "liver failure" or "kidney failure" or "renal failure").tw. (114515)
20 Chronic Disease/ (248675)
21 Health service utilization.mp. (1502)
22 lifestyle-related factor*.mp. (337)
23 or/10-22 (4430236)
24 exp Bipolar Disorder/ (37229)
25 exp Schizophrenia/ (97818)
26 (bipolar or mania or Schizophrenia).tw. (148564)
27 exp Depression/ (102372)
28 exp Mental Disorders/ (1125241)
29 ("mental illness" or "mental disorder*" or depression).tw. (333320)
30 mental health condition*.tw. (1794)
31 exp Depressive Disorder/ (99383)
32 exp Mental Health/ (31108)
33 exp Personality Disorders/ (39084)
34 (severe and persistent mental illness).mp. (304)
35 severe mental illness.mp. (3558)
36 or/24-35 (1390277)
37 9 and 23 and 36 (2648)
38 (dementia or Alzheimer).tw. (106344)
39 (Algeria$ or Egypt$ or Liby$ or Morocc$ or Tunisia$ or Western Sahara$ or Angola$ or Benin or Botswana$ or Burkina Faso or Burundi or Cameroon or Cape Verde or Central African Republic or Chad or Comoros or Congo or Djibouti or Eritrea or Ethiopia$ or Gabon or Gambia$ or Ghana or Guinea or Keny$ or Lesotho or Liberia or Madagasca$ or Malawi or Mali or Mauritania or Mauritius or Mayotte or Mozambiq$ or Namibia$ or Niger or Nigeria$ or Reunion or Rwand$ or Saint Helena or Senegal or Seychelles or Sierra Leone or Somalia or South Africa$ or Sudan or Swaziland or Tanzania or Togo or Ugand$ or Zambia$ or Zimbabw$ or China or Chinese or Hong Kong or Macao or Mongolia$ or Taiwan$ or Belarus or Moldov$ or Russia$ or Ukraine or Afghanistan or Armenia$ or Azerbaijan or Bahrain or Cyprus or Cypriot or Georgia$ or Iran$ or Iraq$ or Jordan$ or Kazakhstan or Kuwait or Kyrgyzstan or Leban$ or Oman or Pakistan$ or Palestin$ or Qatar or Saudi Arabia or Syria$ or Tajikistan or Turkmenistan or United Arab Emirates or Uzbekistan or Yemen or Bangladesh$ or Bhutan or British Indian Ocean Territory or Brunei Darussalam or Cambodia$ or India$ or Indonesia$ or Lao or People's Democratic Republic or Malaysia$ or Maldives or Myanmar or Nepal or Philippin$ or Singapore or Sri Lanka or Thai$ or Timor Leste or Vietnam or Albania$ or Andorra or Bosnia$ or Herzegovina$ or Bulgaria$ or Croatia$ or Faroe Islands or Greenland or Liechtenstein or Lithuani$ or Macedonia or Malta or maltese or Romania or Serbia$ or Montenegro or Svalbard or Argentina$ or Belize or Bolivia$ or Brazil$ or Chilean or Colombia$ or Costa Rica$ or Cuba or Ecuador or El Salvador or French Guiana or Guatemala$ or Guyana or Haiti or Honduras or

For peer review only - http://bmjopen.bmj.com/site/about/guidelines.xhtml

For peer review only

Jamaica$ or Nicaragua$ or Panama or Paraguay or Peru or Puerto Rico or Suriname or Uruguay or Venezuela or developing countr$ or south America$).ti,sh. (1190707)

40 Academic Dissertations/ (0)

41 thesis.tw. (7038)

42 book.pt. (0)

43 Books/ (3122)

44 or/38-43 (1303105)

45 37 not 44 (2373)

46 limit 45 to (English language and humans and "all adult (19 plus years)") (1387)

Combining these 1,387 citations with citations from the remaining three databases produced a total of 4,754 citations, which reduced to 3,033 once duplicates had been removed with the help of the bibliographic software programme EndNote. Screening based on titles alone reduced this total to 55. With titles and abstracts entered into the software screening programme Covidence, these 55 were reduced to 26 citations potentially meeting our inclusion criteria. Items identified clearly confirm the existence of a literature ready to be synthesised, and include (as illustrative examples) a report into health care use by people with schizophrenia at the end of life (Chochinov et al., 2012), a qualitative study of end of life care perspectives amongst people living with the diagnosis of schizophrenia (Sweers et al., 2013), an analysis of routine health records to examine associations between pre-existing mental illness and service use at the end of life (Lavin et al., 2017) and a whole-population study conducted in Taiwan to explore patterns of end of life care use amongst people with, and without, a diagnosis of schizophrenia (Huang et al., 2018).

Relevant items were also found in July 2018 by following Mahood et al.'s (2014) methods for the searching of grey literature by combining "end of life care" AND "severe mental illness" in the Google UK search engine, with examples including presentations from Canada [http://www.shared-care.ca/files/2C_-_Optimizing_end_of_life_care_(Whitehead).pdf] and New Zealand [http://www.nzcmhn.org.nz/files/file/1105/End%20of%20Life%20care%20for%20people%20with%20SPMI.pdf]. Relevant grey literature documents produced by statutory bodies and charities were also identified via targeted searching of organisational websites. Examples are: a recent review into end of life care produced by the Care Quality Commission (2016a), which contains a report in which carers of people also living with mental illness describe their experiences (Care Quality Commission, 2016b); and a joint report produced by St Mungo's and Marie Curie into end of life care for homeless people, which includes a case study of a woman with schizophrenia and cervical cancer (Kennedy et al., 2013).

Screening

As in our scoping, all citations retrieved will be imported into EndNote, where duplicate references will be removed. All remaining items will then be independently assessed for relevance by two members of the review team using the information provided in the title and abstract. Where any doubt exists the full text will be retrieved. In all cases the full text will be retrieved for all citations that, at this stage, appear to meet the review's inclusion criteria. To achieve a high level of consistency reviewers will screen each retrieved citation for inclusion using a purposely designed form. Disagreements will be resolved through discussion with a third reviewer. Authors of research studies will be contacted by the project team if further information is required. All English language items relating to the provision and receipt of end of life care for people with severe mental illness and an additional diagnosis of advanced, incurable, cancer and/or end-stage lung, heart, renal or liver failure will be included at this stage.

Quality appraisal

Following searching and screening, information from research publications will be independently assessed for methodological quality by two reviewers using design-specific tools developed by the Critical Appraisal Skills Programme (CASP) (2016). Alternative tools, reflecting the specific design and methods used in individual research outputs, will be used as necessary if suitable CASP tools are not available. Any disagreement on quality will be resolved through discussion with a third reviewer.

For peer review only - http://bmjopen.bmj.com/site/about/guidelines.xhtml

At this stage all research items will be included other than those which are fatally flawed. Non-research evidence (e.g. policies, reports, expert opinion pieces, case studies etc.) will not be subjected to quality appraisal.

Data extraction

All data will be extracted directly into tables and will follow the format recommended by the CRD (2009). One reviewer will extract the data and a second reviewer will independently check the data extraction forms for accuracy and completeness. Any disagreements will be noted and resolved by consensus within the review team.

Data analysis and synthesis

Researchers informed by the EPPI-Centre approach recognise that different strategies exist for the analysis and synthesis of data (Gough et al., 2017). The synthesis in this review will have both configurative (involving the exploration of potentially heterogeneous materials) and aggregative (involving the pooling of data, where possible) elements (Gough et al., 2012).

For intervention studies meta-analyses of data will be performed where possible. Tests for heterogeneity will be applied. Where statistical pooling is not possible the findings, along with data from non-intervention quantitative studies, will be thematically presented (Thomas and Harden, 2008). The software programme NVivo will be used to help manage this process. Qualitative data, and data from non-research items, will presented in configurative fashion using a thematic approach again assisted by NVivo. Themes will be developed inductively based on close reading of the content of all items included.

Assessing confidence

The strength of findings from the meta-analysis of intervention studies will be assessed using the Grading of Recommendations, Assessment, Development and Evaluation (GRADE) approach (Guyatt et al., 2008). The strength of synthesised qualitative and non-intervention findings will be assessed using the Confidence in the Evidence from Reviews of Qualitative Research (CerQual) approach (Lewin et al., 2015). The original CerQual approach was designed for qualitative findings, but we will use a process previously used by members of this research team in HS&DR 11/1024/08 (Hannigan et al., 2015) and in HS&DR 08/1704/211 (Edwards et al., 2014) in additionally adopting CerQual for the assessment of the confidence of synthesised findings from surveys and other non-intervention quantitative studies.

Overall summary

An overarching summary will bring all elements together. It will present key themes arising from this project as a whole, and do so in accessible manner to ensure reach. Help with this will be sought from the stakeholder advisory group and from the NIHR Dissemination Centre. Factors helping and hindering end of life care for people with severe mental illness will be identified, along with evidence relating to services, processes, interventions, views and experiences. Implications will be stated, and recommendations made for future research.

DISSEMINATION AND PROJECTED OUTPUTS

The main output from this project will be a comprehensive, rigorously conducted, synthesis of research and other evidence relating to services, interventions, processes, views and experiences in the context of end of life care for people with severe mental illness who have an additional diagnosis of advanced, incurable, cancer and/or end-stage lung, heart, renal or liver failure and who are likely to die within the next 12 months.

We will work with the NIHR Dissemination Centre to share findings from our study and to make sure they have maximum benefit, and will follow NIHR guidance by paying close attention to stakeholder

For peer review only - http://bmjopen.bmj.com/site/about/guidelines.xhtml

engagement, format, opportunities, context and timing (National Institute for Health Research, undated). Our stakeholders are policymakers, commissioners, managers and practitioners at the interface of the end of life and mental health care fields, along with people using services and carers. We plan the setting up of a stakeholder advisory group (numbering an anticipated 8-12 individuals), building on preliminary discussions held with clinical and managerial leads at relevant, South Wales-based, NHS and charity mental health services, cancer centres and palliative care organisations. Our list of candidate bodies from which stakeholder representation has been secured (or with which we will otherwise engage in order to keep members informed of our project's progress) currently includes: the National End of Life Care Programme Board in England; the End of Life Care Implementation Board in Wales; the National Mental Health Partnership Board in Wales; and the Mental Health and Dementia Programme Board in England. Professional, academic and management stakeholder organisations include: Mental Health Nurse Academics UK (for which chief applicant Ben Hannigan is elected Vice Chair and Chair-elect, and for which co-applicant Michael Coffey is a past Chair); the European Oncology Nursing Society (the immediate past President of which is a colleague of the applicants at Cardiff University); the Palliative Care Research Society UK, and the National Council for Palliative Care. Roger Pratt and Alan Meudell, as service user and carer co-applicants, will be invited to help identify patient and public representatives to also join this stakeholder group. We will work with our advisory group to inform the decisions we take throughout the life of the study, and will particularly work with members to develop a publicity and dissemination strategy.

In our main output (the full and final report for the NIHR 'Health Services and Delivery Research' journal) we will provide a clear statement of the implications of what we have found for services and practice, and offer explicit recommendations for future research where knowledge gaps are uncovered. We also anticipate working with the NIHR Dissemination Centre to promote our findings through NIHR Signals, Highlights and Themed Reviews where opportunities allow. As we have done in other NIHR studies on which members of this team have worked (e.g., HS&DR 11/1024/08) we will produce a high-quality accessible summary for publicising online, via social media and in paper form. Our intended audience will include end of life and mental health care managers, practitioners and educators along with members of the public and patients. We will work with our stakeholder advisory group to make sure this document is understandable. Papers reporting main findings will be published in gold open access form (to maximise reach) in relevant world-leading journals, tailored to audiences; candidate titles include those in the BioMed Central series, including BMC Psychiatry, BMC Health Services Research and BMC Palliative Care. This will allow all parties free access to information to facilitate decisions on service organisation, and to support meaningful decisions on future care commissioning and provision. We will also present findings at key stakeholder conferences in the mental health and end of life fields and take opportunities to provide briefings for key stakeholder organisations such as those identified above. We will create an opportunity to directly engage with stakeholders via an end of project dissemination event, which will be modelled on single-day impact events in which members of the team have previously participated (e.g., in HS&DR 11/2004/12). Throughout the study we will use social media (e.g., using a dedicated Twitter account) and a project website to promote wider interest in our work, and make opportunities to engage with the public via regular fora such as Cardiff PublicUni (https://en-gb.facebook.com/PUBlicengagementcardiff/). As part of our final dissemination strategy we will request a blogpost on the Mental Elf Blog (https://www.nationalelfservice.net/mental-health/), and will continue using our networks to share what we find as widely as possible. We will keep these approaches to dissemination under active review, and will continue to be advised by NIHR Dissemination Centre colleagues and our stakeholder advisory group as appropriate.

In all our outputs, where our findings support this we will say how high-quality end of life care for people with severe mental illness should be organised, providing guidance to commissioners, managers and practitioners concerned with improving services and the user and carer experience. We anticipate that the new knowledge we create will have a significant impact on health services organisation and delivery, informing action (e.g., via future NICE guidance and other initiatives) to tackle the problem of disadvantaged dying and promote parity of esteem. We also intend this project to be the starting point for a larger programme of related research in its field, designed over time to produce an evidence base supporting transformations in how care is organised and provided. Building on this project we therefore anticipate future data-generating studies investigating current services (including the size and spread of provision) and testing innovations and interventions.

For peer review only - http://bmjopen.bmj.com/site/about/guidelines.xhtml

HS&DR Project 17/100/15: End of life care for people with severe mental illness: an evidence synthesis (the MENLOC study)

PLAN OF INVESTIGATION AND TIMETABLE

We are able to directly begin work on this project on November 1st 2018 as all members of our team are in place, and as we are not required to secure independent NHS research ethics committee approval, due to the nature of the study.

Our projected timetable is as follows:

For peer review only

For peer review only - http://bmjopen.bmj.com/site/about/guidelines.xhtml

	-3 Aug 18	-2 Sep 18	-1 Oct-18	1 Nov 18	2 Dec 18	3 Jan-19	4 Feb 19	5 Mar 19	6 Apr-19	7 May 19	8 Jun-19	9 Jul-19	10 Aug 19	11 Sep 19	12 Oct-19	13 Nov 19	14 Dec 19	15 Jan-20	+1 Feb 20
Notification of award																			
Project set-up (refine protocol, agree subcontracts, comms)																			
Populate stakeholder advisory group (SAG)																			
Project begins																			
SAG meeting																			
Searching and screening for evidence																			
Quality appraisal and data extraction																			
Data analysis, data synthesis and assessing confidence																			
Summary and writing up																			
End of project event																			
Project ends																			
Report to funder																			

For peer review only

For peer review only - http://bmjopen.bmj.com/site/about/guidelines.xhtml

HS&DR Project 17/100/15: End of life care for people with severe mental illness: an evidence synthesis (the MENLOC study)

PROJECT MANAGEMENT

We are a strong and balanced research team well-placed to complete this project on time and to a high standard. We have expertise in: leading and managing NIHR-funded evidence syntheses to completion (Ben Hannigan and Deborah Edwards); mental health services and research (Ben Hannigan and Michael Coffey); cancer, palliative and end of life services and research (Sally Anstey); organ failure services and research (Paul Gill); systematic reviewing (Mala Mann, Deborah Edwards, Ben Hannigan); and lived experience (Alan Meudell, Roger Pratt). The project manager for this study, Deborah Edwards, is a health services researcher and systematic reviewer with experience of managing complex projects across institutional boundaries. As the researcher with the greatest time attachment to the project her responsibilities will include the day-to-day management of the study. Oversight and overall responsibility for the project will fall to the chief investigator, Ben Hannigan, who is an experienced mental health services researcher and who (with Deborah Edwards and others) has successfully completed two competitively funded systematic reviews in the mental health field in the past (including HS&DR 11/1024/08).

An independently chaired stakeholder advisory group will be populated by representatives drawn from the mental health and end of life fields, and will meet with the project team, in Cardiff, at three strategic time points in the life of the study (see plan of investigation above). As an important part of the work of this group will advising on dissemination, impact and engagement details about membership have been given above (see 'Dissemination and projected outputs'). A first meeting will be scheduled at the commencement of the project, to refine search terms and strategies for the evidence review. A second meeting will take place at the completion of evidence searching and screening. A final meeting will take place at the commencement of the whole-project synthesis and report writing phase, where progress and plans for dissemination and maximising impact will be discussed. The costs attached to this project include those associated with the convening and running of the advisory group.

Using a model successfully used in HS&DR 11/1024/08 members of the project team will initially maintain weekly contact via email, telephone and/or videoconference to ensure that packages of work are distributed according to team members' identified responsibilities, and to ensure that work plans proceed according to agreed schedules. Close monitoring of overall progress against milestones will ensure project completion on time and within budget.

APPROVAL BY ETHICS COMMITTEES

No ethics approval is needed, as this is an evidence synthesis.

PATIENT AND PUBLIC INVOLVEMENT

This proposal has been shaped by people with experience of mental health problems, cancer and other long-term conditions, and by people with experience of caring. The original idea for the study arose following sustained and critical discussions between two co-applicants, Roger Pratt and Sally Anstey, who together identified that individuals with severe mental illness are disadvantaged when diagnoses of advanced cancer or end-stage organ failure are made. Roger is a retired mental health social worker, who lives with lymphoma (in long term remission) and heart failure. He cared for his wife who died from advanced peritoneal cancer; she received specialist palliative care in the last year of her life and died in a hospice. This discussion observed that professional perceptions and misperceptions such as stigma and fear impact on management (e.g., pain control, supporting choice and place of care/death) in the case of people with severe mental illness receiving end of life care. We have since involved Alan Meudell in the project team. Alan is a mental health service user consultant and researcher. He has worked on two NIHR studies and one Health and Care Research Wales (HCRW) study, and leads training for HCRW on involving service users in research. He is also interested in the physical health care of people living with mental health difficulties, and on the provision of equitable services.

The roles played by Roger and Alan will be as equal partners in all stages of the research process except the literature search (which will be led, uniquely, by Mala Mann as the team's information services specialist). Both will participate in initial training which all applicants will join, and will work as part of the team in the critical analysis of the selected articles using CASP (or other appropriate) tools. To support active roles in this activity they will be mentored by other team members as necessary and

For peer review only - http://bmjopen.bmj.com/site/about/guidelines.xhtml

will have access to training facilitated by Health and Care Research Wales. They will also contribute to the task of identifying carers and service users to join our stakeholder group. Critically, both will also be invited to advise on the ongoing focus and direction of the study, and to contribute to the accessible writing up of the final report and other outputs. They will be invited to act as ambassadors in the dissemination of the results and to link with existing patient/service user, support and self-help groups to raise awareness and maximise impact.

In preparing this proposal for submission to the HS&DR Programme we also presented our plans to the Patient Experience and Evaluation in Research (PEER) group at Swansea University (http://www.swansea.ac.uk/humanandhealthsciences/research/patientexperienceandevaluationinresearchpeergroup/). The PEER group comprises people with experience of using health care services and of caring, and exists to provide a public and patient view of research proposals before they are submitted for funding. When our proposal was considered by the PEER group members gave it a very positive response, stating that this was a much-needed project in an area that is largely ignored. People were particularly interested in our plans for choosing members of our stakeholder advisory group, and advised us to engage with charities such as Macmillan, with hospices, and with palliative care staff such as nurses. They also recognised the difficulty of actually recruiting stakeholder advisory group members from the target population, but believed we should at least attempt to engage by offering opportunities for people with severe mental illness and end of life diagnoses to participate in any way feasible for them. We have noted this advice, and have approached services in South Wales to seek their agreement in principle to help put us in touch with service users and carers (as well as managers and practitioners) able to advise our project once funded. We will pursue these connections further once confirmation of support has been received from the HS&DR Board.

Our engagement with patients and the public reflects commitments and experiences demonstrated in other studies on which members of this project team have worked. Examples include: HS&DR 11/1024/08 (the RiSC study, an evidence synthesis into 'risk' for young people in mental health hospital which actively involved young people as stakeholders in shaping the study's progress); HS&DR 11/2004/12 and HS&DR 13/10/75 (COCAPP and COCAPP-A which investigated care planning and care coordination in mental health services, in which service users and carers collaborated as members of lived experiences advisory groups and in which people with experience of mental health difficulties worked as researchers conducting qualitative interviews with service user participants); and Health and Care Research Wales SC-12-03 (Plan4Recovery, which involved people with experience of using mental health services as members of a lived experiences advisory group and as qualitative interviewers).

EXPERTISE AND JUSTIFICATION OF SUPPORT REQUIRED

We are a strong and balanced team. Ben Hannigan (BH) is a mental health services researcher who has led a previous NIHR evidence synthesis (HS&DR 11/1024/08) and who has worked on other NIHR (HS&DR 11/2004/12, HS&DR 13/10/75) and Health and Care Research Wales (SC-12-03) mental health studies. He will be chief investigator, contributing across the evidence synthesis, writing up and dissemination. Michael Coffey (MC) is a mental health services researcher and chief investigator on Health and Care Research Wales social care research grant (SC-12-03), and co-investigator on NIHR HS&DR 11/2004/12 and HS&DR 13/10/75. He will contribute to the evidence synthesis, writing up and dissemination. Paul Gill (PG) is an experienced researcher in the field of chronic conditions, and end-stage organ failure particularly. He will contribute to the evidence synthesis, writing up and dissemination. Mala Mann (MM) is an information specialist with expertise in advanced literature searching and the development of systematic review methodologies. She will lead the database searching. Sally Anstey (SA) is an experienced clinician, educationalist and researcher in the end of life and cancer care fields. She will contribute to the to the evidence synthesis, writing up and dissemination. Deborah Edwards (DE) is an experienced health services researcher who has successfully completed multiple systematic reviews (including for the NIHR) using a variety of approaches. She will project manage this study, and contribute to the evidence synthesis, writing up and dissemination. Roger Pratt (RP) is a retired mental health social worker, and a past member of the Velindre Cancer Centre Patient Liaison Group. He will contribute to the to the evidence synthesis, writing up and dissemination. Alan Meudell (AM) is a mental health service user consultant, researcher and trainer, and past trustee of the charity Mind. He has experience in NIHR projects and in training. He will contribute to the to the evidence synthesis, writing up and dissemination.

For peer review only - http://bmjopen.bmj.com/site/about/guidelines.xhtml

HS&DR Project 17/100/15: End of life care for people with severe mental illness: an evidence synthesis (the MENLOC study)

This division of labour and allocation of time is appropriate for a study of this nature. Additional, essential, non-staff costs included are: travel, including to three project/steering group meetings plus refreshments for the same; a sum to cover the costs of interlibrary loans; attendance for Cardiff staff at two conferences, one of which is anticipated to be the International Mental Health Nursing Research Conference; attendance for Swansea staff at one conference; a sum for the planned end of project impact event, to which key stakeholders from the mental health and end of life fields will be invited; a sum for the production and distribution of our accessible summary; and a sum to cover author processing charges for two gold open access publications. Support for these latter costs is requested to maximise dissemination, reach and impact.

REFERENCES

ADDINGTON-HALL, J. 2000. *Positive partnerships: palliative care for adults with severe mental health problems,* London, National Council for Hospice and Specialist Palliative Care Services.

ALL WALES PALLIATIVE CARE PLANNING GROUP 2008. *Report to Minister for Health and Social Services on palliative care services (the Sugar Report),* Cardiff, Welsh Assembly Government.

ALL WALES PALLIATIVE CARE PLANNING GROUP 2009. *Implementation of palliative care report: palliative care services funding 2008 to 2009 (the Finlay Report),* Cardiff, Welsh Assembly Government.

AMERICAN PSYCHIATRIC ASSOCIATION 2014. *Diagnostic and statistical manual of mental disorders (DSM-V),* Arlington, American Psychiatric Publishing.

BATTY, G. D., WHITLEY, E., GALE, C. R., OSBORN, D., TYNELIUS, P. & RASMUSSEN, F. 2012. Impact of mental health problems on case fatality in male cancer patients. *British Journal of Cancer,* 106**,** 1842-1845.

CARE QUALITY COMMISSION 2016a. *A different ending: end of life care review,* Newcastle upon Tyne, Care Quality Commission.

CARE QUALITY COMMISSION 2016b. *A different ending: end of life care review. People with a mental health condition,* Newcastle upon Tyne, Care Quality Commission.

CARNEY, C. P., JONES, L. & WOOLSON, R. F. 2006. Medical comorbidity in women and men with schizophrenia. *Journal of General Internal Medicine,* 21**,** 1133-1137.

CENTRE FOR MENTAL HEALTH 2010. *The economic and social costs of mental health problems in 2009/10,* London, Centre for Mental Health.

CENTRE FOR REVIEWS AND DISSEMINATION 2009. *Systematic reviews: CRD's guidance for undertaking reviews in health care,* York, University of York.

CHESNEY, E., GOODWIN, G. M. & FAZEL, S. 2014. Risks of all-cause and suicide mortality in mental disorders: a meta-review. *World Psychiatry,* 13**,** 153-160.

CHOCHINOV, H. M., MARTENS, P. J., PRIOR, H. J. & KREDENTSER, M. S. 2012. Comparative health care use patterns of people with schizophrenia near the end of life: A population-based study in Manitoba, Canada. *Schizophrenia Research,* 141**,** 241-246.

CRITICAL APPRAISAL SKILLS PROGRAMME (CASP). 2016. *CASP checklists* [Online]. http://www.casp-uk.net/#!checklists/cb36. [Accessed].

DEPARTMENT OF HEALTH 1995. *Building bridges: a guide to arrangements for interagency working for the care and protection of severely mentally ill people,* London, Department of Health.

DEPARTMENT OF HEALTH 2010. *National cancer patient experience survey programme: 2010 national survey report,* London, Department of Health.

DEPARTMENT OF HEALTH 2016. *Our commitment to you for end of life care,* London, Department of Health.

DEPARTMENT OF HEALTH 2017. *A framework for mental health research,* London, Department of Health.

DESAI, M. M., BRUCE, M. L. & KASL, S. V. 1999. The effects of major depression and phobia on stage at diagnosis of breast cancer. *International Journal of Psychiatry in Medicine,* 29**,** 29-45.

DISABILITY RIGHTS COMMISSION 2006. *Equal treatment: closing the gap. A formal investigation into physical health inequalities experienced by people with learning disabilities and/or mental health problems,* London, Disability Rights Commission.

DIXON, J., KING, D., MATOSEVIC, T., CLARK, M. & KNAPP, M. 2015. *Equity in provision of palliative care in the UK,* London, LSE and Marie Curie.

DRAKEFORD, M. 2014. *Palliative care services in Wales are making an enormous difference to people's quality of life,* Speech given in Ty Gobaith hospice, Conwy, http://www.wired-

For peer review only - http://bmjopen.bmj.com/site/about/guidelines.xhtml

HS&DR Project 17/100/15: End of life care for people with severe mental illness: an evidence synthesis (the MENLOC study)

gov.net/wg/news.nsf/articles/Palliative+care+services+in+Wales+are+making+an+enormous+difference+to+peoples+quality+of+life+Mark+Drakeford+28102014162040?open.
EDWARDS, D., NOYES, J., HAF SPENCER, L. & GREGORY, J. W. 2014. An ongoing struggle: a mixed-method systematic review of interventions, barriers and facilitators to achieving optimal self-care by children and young people with type 1 diabetes in educational settings. *BMC Paediatrics,* 14.
ELLISON, N. 2008. *Mental health and palliative care. Literature review,* London, Mental Health Foundation.
EXLEY, C. 2004. Review article: the sociology of dying, death and bereavement. *Sociology of Health and Illness,* 26**,** 110-122.
FOTI, M. E. 2012. Palliative care for patients with serious mental Illness. *In:* CHOCHINOV, H. M. & BREITBART, W. (eds.) *Handbook of psychiatry in palliative medicine.* 2nd ed. Oxford: Oxford University Press.
FRIEDLI, L. & PARSONAGE, M. 2009. *Promoting mental health and preventing mental illness: the economic case for investment in Wales,* Cardiff, All Wales Mental Health Promotion Network.
GLOBAL BURDEN OF DISEASE STUDY 2013 COLLABORATORS 2015. Global, regional, and national incidence, prevalence, and years lived with disability for 301 acute and chronic diseases and injuries in 188 countries, 1990-2013: a systematic analysis for the Global Burden of Disease Study 2013. *The Lancet,* 386**,** 743-800.
GOUGH, D., OLIVER, S. & THOMAS, J. (eds.) 2017. *An introduction to systematic reviews,* London: Sage.
GOUGH, D., THOMAS, J. & OLIVER, S. 2012. Clarifying differences between review designs and methods. *Systematic Reviews,* 1.
GUYATT, G. H., OXMAN, A. D., VIST, G. E., KUNZ, R., FALCK-YTTER, Y., ALONSO-COELLO, P. & SCHÜNEMANN, H. J. 2008. GRADE: an emerging consensus on rating quality of evidence and strength of recommendations. *BMJ,* 336**,** 924-926.
HACKETT, A. & GAITAN, A. 2007. A qualitative study assessing mental health issues in two hospices in the UK. *International Journal of Palliative Care Nursing,* 13**,** 273-281.
HANNIGAN, B., EDWARDS, D., EVANS, N., GILLEN, E., LONGO, M., PRYJMACHUK, S. & TRAINOR, G. 2015. An evidence synthesis of risk identification, assessment and management for young people using tier 4 inpatient child and adolescent mental health services. *Health Services and Delivery Research,* 3.
HOWARD, L. M., BARLEY, E. A., DAVIES, E., RIGG, A., LEMPP, H., ROSE, D. & THORNICROFT, G. 2010. Cancer diagnosis in people with severe mental illness: practical and ethical issues. *The Lancet Oncology,* 11**,** 797-804.
HUANG, H. K., WANG, Y.W., HSIEH, J. G. & HSIEH, C. J. 2018. Disparity of end-of-life care in cancer patients with and without schizophrenia: A nationwide population-based cohort study. *Schizophrenia Research,* 195**,** 434-440.
HUGHES-HALLET, T., CRAFT, A. & DAVIES, C. 2011. *Palliative care funding review: funding the right care and support for everyone,* London, Department of Health.
INDEPENDENT CANCER TASKFORCE. 2015. *Achieving world-class cancer outcomes: a strategy for England 2015-2020* [Online]. http://www.cancerresearchuk.org/sites/default/files/achieving_world-class_cancer_outcomes_-_a_strategy_for_england_2015-2020.pdf. [Accessed].
IRWIN, K. E., HENDERSON, D. C., KNIGHT, H. P. & PIRL, W. F. 2014. Cancer care for individuals with schizophrenia. *Cancer,* 120**,** 323-334
ISHIKAWA, H., YASUNAGA, H., MATSUI, H., FUSHIMI, K. & KAWAKAMI, N. 2016. Differences in cancer stage, treatment and in-hospital mortality between patients with and without schizophrenia: retrospective matched-pair cohort study. *British Journal of Psychiatry,* 208**,** 239-244.
KENNEDY, P., SARAFI, C. & GREENISH, W. 2013. *Homelessness and end of life care*, Marie Curie Cancer Care and St Mungo's.
KNAPIK, G. P. & GRAOR, C. H. 2013. Engaging persons with severe persistent mental illness into primary care. *Journal for Nurse Practitioners,* 9**,** 283-287.
KRONENBERG, C., DORAN, T., GODDARD, M., KENDRICK, T., GILBODY, S., DARE, C. R., AYLOTT, L. & JACOBS, R. 2017. Identifying primary care quality indicators for people with serious mental illness: a systematic review. *British Journal of General Practice,* 67**,** e519-e530.
LAVIN, K., DAVYDOW, D. S., DOWNEY, L., ENGELBERG, R. A., DUNLAP, B., SIBLEY, J., LOBER, W. B., OKIMOTO, K., KHANDELWAL, N., LOGGERS, E. T., TENO, J. M. & CURTIS, J. R. 2017. Effect of psychiatric illness on acute care utilization at end of life from serious medical illness. *Journal of Pain and Symptom Management,* 54**,** 176-185.

For peer review only - http://bmjopen.bmj.com/site/about/guidelines.xhtml

HS&DR Project 17/100/15: End of life care for people with severe mental illness: an evidence synthesis (the MENLOC study)

LEWIN, S., GLENTON, C., MUNTHE-KAAS, H., CARLSEN, B., COLVIN, C. J., GÜLMEZOGLU, M., NOYES, J., BOOTH, A., GARSIDE, R. & RASHIDIAN, A. 2015. Using qualitative evidence in decision making for health and social interventions: an approach to assess confidence in findings from qualitative evidence syntheses (GRADE-CERQual). *PLOS Medicine,* 12**,** e1001895.

MAHOOD, Q., VAN EERD, D. & IRVIN, E. 2014. Searching for grey literature for systematic reviews: challenges and benefits. *Research Synthesis Methods,* 5**,** 221-234.

MARIE CURIE 2015. *Triggers for palliative care. Improving access to care for people with diseases other than cancer: implications for Wales,* London, Marie Curie.

MARIE CURIE AND THE BEVAN FOUNDATION 2014. *Death and dying in Wales,* London and Cardiff, Marie Curie and the Bevan Foundation.

MCCORMACK, P. & SHARP, D. M. M. 2006. Palliative care for people with mental health problems. *European Journal of Palliative Care,,* 13**,** 198-201.

MENTAL HEALTH TASKFORCE 2016. *The five year forward view for mental health*, Mental Health Taskforce.

MILLMAN, J., GALWAY, K., SANTIN, O. & REID, J. 2016. Cancer and serious mental illness – patient, caregiver and professional perspectives: study protocol. *Journal of Advanced Nursing,* 72**,** 217-226.

MOHER, D., LIBERATI, A., TETZLAFF, J., ALTMAN, D. G. & GROUP, T. P. 2009. Preferred reporting items for systematic reviews and meta-analyses: the PRISMA statement. *PLoS Medicine,* 6**,** e1000097.

MORGAN, B. D. 2016. 'No right place to die'. Nursing attitudes and needs in caring for people with serious mental illness at end-of-life. *Journal of the American Psychiatric Nurses Association,* 22**,** 31-42.

NATIONAL INSTITUTE FOR CLINICAL EXCELLENCE 2004. *Improving supportive and palliative care for adults with cancer,* London, National Institute for Clinical Excellence.

NATIONAL INSTITUTE FOR HEALTH AND CARE EXCELLENCE 2009. *Borderline personality disorder: recognition and management,* London, National Institute for Health and Care Excellence.

NATIONAL INSTITUTE FOR HEALTH AND CARE EXCELLENCE 2011. *Service user experience in adult mental health services,* London, National Institute for Health and Care Excellence.

NATIONAL INSTITUTE FOR HEALTH AND CARE EXCELLENCE 2014a. *Bipolar disorder: assessment and management,* London, National Institute for Health and Care Excellence.

NATIONAL INSTITUTE FOR HEALTH AND CARE EXCELLENCE 2014b. *Psychosis and schizophrenia in adults: prevention and management,* London, National Institute for Health and Care Excellence.

NATIONAL INSTITUTE FOR HEALTH AND CARE EXCELLENCE 2018 [anticipated]. *End of life care for adults in the last year of life: service delivery,* London, National Institute for Health and Care Excellence.

NATIONAL INSTITUTE FOR HEALTH RESEARCH 2017. *The Dissemination Centre's upcoming work schedule*, http://www.dc.nihr.ac.uk/about/upcoming-work.htm.

NATIONAL INSTITUTE FOR HEALTH RESEARCH undated. *How to disseminate your research: getting your message heard - and used,* https://www.nihr.ac.uk/funding-and-support/documents/funding-for-research-studies/manage-my-study/How-to-disseminate-your-research/dissemination-guidance.pdf.

NEWTON, J. N., BRIGGS, A. D. M., MURRAY, C. J. L., DICKER, D., FOREMAN, K. J., WANG, H., NAGHAVI, M., FOROUZANFAR, M., OHNO, S. L., BARBER, R. M., VOS, T., STANAWAY, J. D., SCHMIDT, J. C., HUGHES, A. J., FAY, D. F. J., ECOB, R., GRESSER, C., MCKEE, M., RUTTER, H., ABUBAKAR, I., ALI, R., ANDERSON, H. R., BANERJEE, A., BENNETT, D., BERNABÉ, E., BHUI, K. S., BIRYUKOV, S. M., BOURNE, R. R., BRAYNE, C. E. G., BRUCE, N. G., BRUGHA, T. S., BURCH, M., CAPEWELL, S., CASEY, D., CHOWDHURY, R., COATES, M. M., COOPER, C., CRITCHLEY, J. A., DARGAN, P. I., DHERANI, M. K., ELLIOTT, P., EZZATI, M., FENTON, K. A., FRASER, M. S., FÜRST, T., GREAVES, F., GREEN, M. A., GUNNELL, D. J., HANNIGAN, B. M., HAY, R. J., HAY, S. I., HEMINGWAY, H., LARSON, H. J., LOOKER, K. J., LUNEVICIUS, R., LYONS, R. A., MARCENES, W., MASON-JONES, A. J., MATTHEWS, F. E., MOLLER, H., MURDOCH, M. E., NEWTON, C. R., PEARCE, N., PIEL, F. B., POPE, D., RAHIMI, K., RODRIGUEZ, A., SCARBOROUGH, P., SCHUMACHER, A. E., SHIUE, I., SMEETH, L., TEDSTONE, A., VALABHJI, J., WILLIAMS, H. C., WOLFE, C. D. A., WOOLF, A. D. & DAVIS, A. C. J. 2015. Changes in health in England, with analysis by English regions and areas of deprivation, 1990-2013: a systematic analysis for the Global Burden of Disease Study 2013. *The Lancet,* 386**,** 2257-2274.

For peer review only - http://bmjopen.bmj.com/site/about/guidelines.xhtml

NORTHERN IRELAND EXECUTIVE 2010. *Living matters, dying matters. A palliative and end of life care strategy for adults in Northern Ireland,* Belfast, Northern Ireland Executive.
PAYNE, S. 2011. From here to there: reaching out for palliative care. *European Journal of Palliative Care,* 18**,** 264-265.
PICKER INSTITUTE EUROPE 2017. *Wales Cancer Patient Experience Survey 2016: National Report,* Oxford, Picker Institute Europe.
PICOT, S. A., GLAETZER, K. M. & MYHILL, K. J. 2015. Coordinating end of life care for individuals with a mental illness: a nurse practitioner collaboration. *Collegian,* 22**,** 143-149.
PRICE, A., HOTOPF, M., HIGGINSON, I., MONROE, B. & HENDERSON, M. 2006. Psychological services in hospices in the UK and Republic of Ireland. *Journal of the Royal Society of Medicine,* 99**,** 637-639.
PROSPERO: INTERNATIONAL PROSPECTIVE REGISTER OF SYSTEMATIC REVIEWS undated. https://www.crd.york.ac.uk/prospero/.
SCOTTISH GOVERNMENT 2008a. *Better cancer care: an action plan,* Edinburgh, Scottish Government.
SCOTTISH GOVERNMENT 2008b. *Living and dying well: a national action plan for palliative and end of life care in Scotland,* Edinburgh, Scottish Government.
SCOTTISH GOVERNMENT 2009. *Improving the health and wellbeing of people with long term conditions in Scotland: a national action plan,* Edinburgh, Scottish Government.
SHALEV, D., BREWSTER, K., ARBUCKLE, M. R. & LEVENSON, J. A. 2017. A staggered edge: end-of-life care in patients with severe mental illness. *General Hospital Psychiatry,* 44**,** 1-3.
SWEERS, K., DIERCKX DE CASTERLÉ, B., DETRAUX, J. & DE HERT, M. 2013. End-of-life (care) perspectives and expectations of patients with schizophrenia. *Archives of Psychiatric Nursing,* 27**,** 246-252.
TAYLOR, J., SWETENHAM, K., MYHILL, K., PICOT, S., GLAETZER, K. & VAN LOON, A. 2012. IMhPaCT: an education strategy for cross-training palliative care and mental health clinicians. *International Journal of Palliative Nursing,* 18**,** 290-294.
THOMAS, J. & HARDEN, A. 2008. Methods for the thematic synthesis of qualitative research in systematic reviews. *BMC Medical Research Methodology,* 8.
THORNICROFT, G. 2013. Premature death among people with mental illness. *BMJ,* 346.
WARREN, M. B., LAPID, M. I., MCKEAN, A. J., CHA, S. S., STEVENS, M. A., BREKKE, F. M. & BURTON, M. C. 2014. Code status discussions in psychiatric and medical inpatients. *Journal of Clinical Psychiatry,* 76**,** 49-53.
WELSH ASSEMBLY GOVERNMENT 2005. *Designed for life: creating world class health and social care for Wales in the 21st Century,* Cardiff, Welsh Assembly Government.
WELSH GOVERNMENT 2013. *Together for health: delivering end of life care. A delivery plan up to 2016 for NHS Wales and its partners,* Cardiff, Welsh Government.
WOODS, A., WILLISON, K., KINGTON, C. & GAVIN, A. 2008. Palliative care for people with severe persistent mental illness: A review of the literature. *The Canadian Journal of Psychiatry,* 53**,** 725-736.
WORLD HEALTH ORGANIZATION 1992. *The 10th revision of the international classification of diseases and related health problems (ICD-10),* Geneva, World Health Organization.
ZABORA, J. R., LOSCALZO, M. & SMITH, E. 2000. Psychosocial rehabilitation. *In:* ABELOFF, M. D., ARMITAGE, J. O., LICHTER, A. S. & NIEDERHUBER, J. E. (eds.) *Clinical oncology.* New York: Churchill Livingstone.

For peer review only - http://bmjopen.bmj.com/site/about/guidelines.xhtml

Reporting checklist for systematic review and meta-analysis.

Based on the PRISMA guidelines.

Instructions to authors

Complete this checklist by entering the page numbers from your manuscript where readers will find each of the items listed below.

Your article may not currently address all the items on the checklist. Please modify your text to include the missing information. If you are certain that an item does not apply, please write "n/a" and provide a short explanation.

Upload your completed checklist as an extra file when you submit to a journal.

In your methods section, say that you used the PRISMAreporting guidelines, and cite them as:

Moher D, Liberati A, Tetzlaff J, Altman DG, The PRISMA Group. Preferred Reporting Items for Systematic Reviews and Meta-Analyses: The PRISMA Statement

		Reporting Item	Page Number
**Title**			
	#1	Identify the report as a systematic review, meta-analysis, or both.	1
**Abstract**			
Structured summary	#2	Provide a structured summary including, as applicable: background; objectives; data sources; study eligibility criteria, participants, and interventions; study appraisal and synthesis methods; results; limitations; conclusions and implications of key findings; systematic review registration number	2
**Introduction**			
Rationale	#3	Describe the rationale for the review in the context of what is already known.	5

For peer review only

Objectives	#4	Provide an explicit statement of questions being addressed with reference to participants, interventions, comparisons, outcomes, and study design (PICOS).	2
**Methods**			
Protocol and registration	#5	Indicate if a review protocol exists, if and where it can be accessed (e.g., Web address) and, if available, provide registration information including the registration number.	5
Eligibility criteria	#6	Specify study characteristics (e.g., PICOS, length of follow-up) and report characteristics (e.g., years considered, language, publication status) used as criteria for eligibility, giving rational	6
Information sources	#7	Describe all information sources in the search (e.g., databases with dates of coverage, contact with study authors to identify additional studies) and date last searched.	7
Search	#8	Present full electronic search strategy for at least one database, including any limits used, such that it could be repeated.	7
Study selection	#9	State the process for selecting studies (i.e., for screening, for determining eligibility, for inclusion in the systematic review, and, if applicable, for inclusion in the meta-analysis).	8
Data collection process	#10	Describe the method of data extraction from reports (e.g., piloted forms, independently by two reviewers) and any processes for obtaining and confirming data from investigators.	8
Data items	#11	List and define all variables for which data were sought (e.g., PICOS, funding sources), and any assumptions and simplifications made.	6
Risk of bias in individual studies	#12	Describe methods used for assessing risk of bias in individual studies (including specification of whether this was done at the study or outcome level, or both), and how this information is to be used in any data synthesis.	N/A
Summary measures	#13	State the principal summary measures (e.g., risk ratio, difference in means).	N/A

For peer review only

Planned methods of analyis	#14	Describe the methods of handling data and combining results of studies, if done, including measures of consistency (e.g., I2) for each meta-analysis.	8
Risk of bias across studies	#15	Specify any assessment of risk of bias that may affect the cumulative evidence (e.g., publication bias, selective reporting within studies).	N/A
Additional analyses	#16	Describe methods of additional analyses (e.g., sensitivity or subgroup analyses, meta-regression), if done, indicating which were pre-specified.	8
**Results**			
Study selection	#17	Give numbers of studies screened, assessed for eligibility, and included in the review, with reasons for exclusions at each stage, ideally with a flow diagram.	8
Study characteristics	#18	For each study, present characteristics for which data were extracted (e.g., study size, PICOS, follow-up period) and provide the citation.	9
Risk of bias within studies	#19	Present data on risk of bias of each study and, if available, any outcome-level assessment (see Item 12).	N/A
Results of individual studies	#20	For all outcomes considered (benefits and harms), present, for each study: (a) simple summary data for each intervention group and (b) effect estimates and confidence intervals, ideally with a forest plot.	N/A
Synthesis of results	#21	Present the main results of the review. If meta-analyses are done, include for each, confidence intervals and measures of consistency.	9-15
Risk of bias across studies	#22	Present results of any assessment of risk of bias across studies (see Item 15).	N/A
Additional analysis	#23	Give results of additional analyses, if done (e.g., sensitivity or subgroup analyses, meta-regression [see Item 16]).	10
**Discussion**			
Summary of Evidence	#24	Summarize the main findings, including the strength of evidence for each main outcome; consider their relevance to	15-16

For peer review only

		key groups (e.g., health care providers, users, and policy makers	
Limitations	#25	Discuss limitations at study and outcome level (e.g., risk of bias), and at review level (e.g., incomplete retrieval of identified research, reporting bias).	16
Conclusions	#26	Provide a general interpretation of the results in the context of other evidence, and implications for future research.	17
**Funding**			
Funding	#27	Describe sources of funding or other support (e.g., supply of data) for the systematic review; role of funders for the systematic review.	17

The PRISMA checklist is distributed under the terms of the Creative Commons Attribution License CC-BY. This checklist was completed on 26. April 2021 using https://www.goodreports.org/, a tool made by the EQUATOR Network in collaboration with Penelope.ai

For peer review only

Database search strategies

Database: AMED (Allied and Complementary Medicine)

Search Strategy:

----

1 exp Palliative care/ (6092)

2 exp Hospice care/ (2100)

3 exp Terminal Care/ (5265)

4 ("palliative care" or hospice or "end of life care" or end-of-life).tw. (13126)

5 ((hospice or terminal*) adj3 (care or caring or ill*)).tw. (7843)

6 ("Irreversible condition" or "terminal condition" or fatal illness).tw. (27)

7 ("last year of life" or LYOL or "end of life" or "end of their lives" or "last six months of life" or "last 6 months of life").tw. (3749)

8 (end-stage disease* or end stage disease* or end-stage ill* or end stage ill* or end-stage or end stage).tw. (539)

9 (expected adj3 die).tw. (15)

10 (imminent adj3 death).tw. (44)

11 ("Dying soon" or "expected death" or "imminently dying" or Moribund).tw. (40)

12 (conservative adj2 (treatment or management)).tw. (1010)

13 Withholding Treatment/ (66)

14 Treatment Refusal/ (134)

15 (Refus* adj3 (treat* or care or intervention or dialysis)).tw. (272)

16 ((withdrew or withdraw* or withhold*) adj3 (treat* or car* or intervene* or therap* or dialysis or transplant*)).tw. (363)

17 or/1-16 (16993)

18 exp Neoplasms/ (14717)

19 (cancer* or tumo?r* or neoplas* or malignan* or carcinoma* or adenocarcinoma* or adeno?carcinoma* or choriocrcinoma* or leukemia* or leukaemia* or metastat* or sarcoma* or teratoma* or lymphoma).tw. (19919)

20 enzyme disorder.tw. (1)

21 Hereditary disease/ (147)

For peer review only

22 ("duchenne muscular dystrophy" or "amyotrophic lateral sclerosis" or ALS).tw. (1007)

23 amyotrophic lateral sclerosis/ (223)

24 Muscular dystrophy, Duchenne/ (70)

25 ("Genetic disease" or "genetic condition").tw. (18)

26 Cystic Fibrosis/ (451)

27 ("cystis fibrosis" or CF).tw. (314)

28 "Multi* Organ Failure".tw. (20)

29 ("Organ failure" or "chronic organ failure").tw. (49)

30 Pulmonary Disease, Chronic Obstructive/ (449)

31 ("renal insufficiency" or "serious physical illness").tw. (27)

32 (Chronic obstructive pulmonary disease or COPD).tw. (1665)

33 (chronic adj3 (illness or condition or disease*)).tw. (9365)

34 "chronic medical condition".tw. (10)

35 Heart Failure/ (394)

36 (chronic adj2 ("cardiac failure" or "liver failure" or "kidney failure" or "end-stage renal disease" or ESRD or "renal failure" or "heart failure")).tw. (439)

37 Chronic Disease/ (6305)

38 Health service utilization.mp. (25)

39 lifestyle-related factor*.mp. (3)

40 or/18-39 (31309)

41 exp Bipolar Disorder/ (81)

42 exp Schizophrenia/ (931)

43 (schizo* or "mood disorder*" or "personality disorder*" or psychotic* or psychosis or psychoses).tw. (2312)

44 (bipolar or mania or Schizophrenia).tw. (1447)

45 (Depression adj2 (psychosis or psychotic or severe or major)).tw. (390)

46 exp Mental Disorders/ (27114)

47 mental health condition*.tw. (43)

48 exp Personality Disorders/ (323)

49 exp psychotic disorders/ (424)

50 ("severe mental illness" or "persistent mental illness").tw. (362)

For peer review only

51 ((chronic* or sever* or serious or persistent* or enduring or debilitating) adj2 (mental* or psychological*) adj2 (ill* or disorder* or health)).tw. (936)

52 (SPMI or SMI).tw. (79)

53 or/41-52 (27932)

54 (dementia or Alzheimer).ti. (1706)

55 (bipolar electrocoagulation or bipolar radiofrequency or bipolar tumour probe or bipolar diathermy).tw. (6)

56 ("respiratory depression" or "marrow depression" or "hematologic* depression").tw. (42)

57 (child* or adoles* or pediatric or paediatric).tw. (27044)

58 (Algeria$ or Egypt$ or Liby$ or Morocc$ or Tunisia$ or Western Sahara$ or Angola$ or Benin or Botswana$ or Burkina Faso or Burundi or Cameroon or Cape Verde or Central African Republic or Chad or Comoros or Congo or Djibouti or Eritrea or Ethiopia$ or Gabon or Gambia$ or Ghana or Guinea or Keny$ or Lesotho or Liberia or Madagasca$ or Malawi or Mali or Mauritania or Mauritius or Mayotte or Mozambiq$ or Namibia$ or Niger or Nigeria$ or Reunion or Rwand$ or Saint Helena or Senegal or Seychelles or Sierra Leone or Somalia or South Africa$ or Sudan or Swaziland or Tanzania or Togo or Ugand$ or Zambia$ or Zimbabw$ or China or Chinese or Hong Kong or Macao or Mongolia$ or Taiwan$ or Belarus or Moldov$ or Russia$ or Ukraine or Afghanistan or Armenia$ or Azerbaijan or Bahrain or Cyprus or Cypriot or Georgia$ or Iran$ or Iraq$ or Jordan$ or Kazakhstan or Kuwait or Kyrgyzstan or Leban$ or Oman or Pakistan$ or Palestin$ or Qatar or Saudi Arabia or Syria$ or Tajikistan or Turkmenistan or United Arab Emirates or Uzbekistan or Yemen or Bangladesh$ or Bhutan or British Indian Ocean Territory or Brunei Darussalam or Cambodia$ or India$ or Indonesia$ or Lao or People's Democratic Republic or Malaysia$ or Maldives or Myanmar or Nepal or Philippin$ or Singapore or Sri Lanka or Thai$ or Timor Leste or Vietnam or Albania$ or Andorra or Bosnia$ or Herzegovina$ or Bulgaria$ or Croatia$ or Faroe Islands or Greenland or Liechtenstein or Lithuani$ or Macedonia or Malta or maltese or Romania or Serbia$ or Montenegro or Svalbard or Argentina$ or Belize or Bolivia$ or Brazil$ or Chilean or Colombia$ or Costa Rica$ or Cuba or Ecuador or El Salvador or French Guiana or Guatemala$ or Guyana or Haiti or Honduras or Jamaica$ or Nicaragua$ or Panama or Paraguay or Peru or Puerto Rico or Suriname or Uruguay or Venezuela or developing countr$ or south America$).ti,sh. (9674)

59 or/54-58 (37507)

60 17 and 40 and 53 (230)

61 60 not 59 (193)

Proquest ASSIA

(("Dying soon" or "expected death" or "imminently dying" or Moribund) OR ("end-stage disease*" or "end stage disease*" or "end-stage ill*" or "end stage ill*" or end-stage or “end stage”) OR ("last year of life" or LYOL or "end of life" or "end of their lives" or "last six months of life" or "last 6 months of life") OR ("Irreversible condition" or "terminal condition" or "fatal illness") OR ("palliative care" or hospice or "end of life care" or end-of-life or "Terminal care" or "terminal* ill*")) AND ((Neoplasm* or cancer* or tumor* or tumour or neoplas* or malignan* or carcinoma* or

For peer review only

adenocarcinoma* or choriocrcinoma* or leukemia* or leukaemia* or metastat* or sarcoma* or teratoma* or lymphoma) OR ("duchenne muscular dystrophy" or "amyotrophic lateral sclerosis" or ALS) OR ("Genetic disease" or "genetic condition" or "cystis fibrosis" or CF) OR ("Organ failure" or "chronic organ failure" or "mulitple organ failure") OR (Chronic obstructive pulmonary disease or COPD or "renal insufficiency" or "serious physical illness" or "chronic medical condition") OR (chronic NEAR/3 ("cardiac failure" or "liver failure" or "kidney failure" or "end-stage renal disease" or ESRD or "renal failure" or "heart failure")) OR (chronic NEAR/3 (illness or condition or disease*))) AND ((Bipolar or schizo* or "mood disorder*" or "personality disorder*" or psychotic* or psychosis or psychoses) OR (Depression NEAR/2 (psychosis or psychotic or severe or major)) OR ("psychotic disorders" or "schizoaffective disorder" or "severe and persistent mental illness") OR ("psychotic disorders" or "schizoaffective disorder" or "Paranoid Disorders") OR ("severe mental illness" or "persistent mental illness" or SPMI or SMI))

CINAHL Search History

Select / deselect all

	Search ID#	Search Terms	Search Options	Actions
	S46	S15 AND S32 AND S45	Limiters - English Language; Age Groups: All Adult<br>Search modes - Boolean/Phrase	View Results (356) View Details Edit
	S45	S33 OR S34 OR S35 OR S36 OR S37 OR S38 OR S39 OR S40 OR S41 OR S42 OR S43 OR S44	Search modes - Boolean/Phrase	View Results (124,935) View Details Edit
	S44	AB SPMI or SMI	Search modes - Boolean/Phrase	View Results (1,211) View Details Edit
	S43	AB (chronic* or sever* or serious or persistent* or enduring or debilitating) AND AB (mental* or psychological*) AND AB (ill* or disorder* or health)	Search modes - Boolean/Phrase	View Results (31,921) View Details Edit
	S42	AB (chronic* or sever* or serious or persistent* or enduring or debilitating) AND AB (mental* or	Search modes - Boolean/Phrase	View Results (0)

For peer review only

		psychological*) adj2 (ill* or disorder* or health)		View Details Edit
☐	S41	AB (bipolar or mania or Schizophrenia) OR AB (psychotic* or psychosis or psychoses) OR AB (schizo* or "mood disorder*" or "personality disorder*") OR AB "mental health condition*" OR AB "severe mental illness" OR AB "persistent mental illness"	Search modes - Boolean/Phrase	View Results (38,951) View Details Edit
☐	S40	(TX "Mentally Ill person*")	Search modes - Boolean/Phrase	View Results (698) View Details Edit
☐	S39	(MH "Paranoid Disorders")	Search modes - Boolean/Phrase	View Results (617) View Details Edit
☐	S38	(MH "Psychotic Disorders")	Search modes - Boolean/Phrase	View Results (9,609) View Details Edit
☐	S37	(MH "Schizoaffective Disorder")	Search modes - Boolean/Phrase	View Results (223) View Details Edit
☐	S36	(MH "Personality Disorders")	Search modes - Boolean/Phrase	View Results (3,809) View Details Edit
☐	S35	(MH "Mental Disorders")	Search modes - Boolean/Phrase	View Results (48,711) View Details Edit
☐	S34	(MH "Schizophrenia")	Search modes - Boolean/Phrase	View Results (21,058) View Details

For peer review only

☐	S33	(MH "Bipolar Disorder")	Search modes - Boolean/Phrase	Edit View Results (9,957) View Details
☐	S32	S16 OR S17 OR S18 OR S19 OR S20 OR S21 OR S22 OR S23 OR S24 OR S25 OR S26 OR S27 OR S28 OR S29 OR S30 OR S31	Search modes - Boolean/Phrase	Edit View Results (444,331) View Details
☐	S31	TX "lifestyle-related factor*"	Search modes - Boolean/Phrase	Edit View Results (215) View Details
☐	S30	(MH "Chronic Disease")	Search modes - Boolean/Phrase	Edit View Results (53,902) View Details
☐	S29	AB chronic N2 "cardiac failure" OR AB chronic N2 "liver failure" OR AB chronic N2 "kidney failure" OR AB chronic N2 "end-stage renal disease" OR AB chronic N2 ESRD OR AB chronic N2 "renal failure" OR AB chronic N2 "heart failure"	Search modes - Boolean/Phrase	Edit View Results (4,975) View Details
☐	S28	(MH "Liver Failure")	Search modes - Boolean/Phrase	Edit View Results (1,681) View Details
☐	S27	(MH "Renal Insufficiency")	Search modes - Boolean/Phrase	Edit View Results (5,723) View Details
☐	S26	(MH "Heart Failure")	Search modes - Boolean/Phrase	Edit View Results (34,187) View Details
☐	S25	(MH "Pulmonary Disease, Chronic Obstructive") OR	Search modes -	Edit View

For peer review only

		(MH "Lung Diseases, Obstructive") OR (MH "Chronic Disease")	Boolean/Phrase	Results (72,636) View Details Edit
☐	S24	(MH "Multiple Organ Dysfunction Syndrome")	Search modes - Boolean/Phrase	View Results (2,454) View Details Edit
☐	S23	Multiple Organ Failure	Search modes - Boolean/Phrase	View Results (0) View Details Edit
☐	S22	(MH "Cystic Fibrosis")	Search modes - Boolean/Phrase	View Results (6,385) View Details Edit
☐	S21	(MH "Muscular Dystrophy, Duchenne")	Search modes - Boolean/Phrase	View Results (1,331) View Details Edit
☐	S20	(MH "Amyotrophic Lateral Sclerosis")	Search modes - Boolean/Phrase	View Results (3,299) View Details Edit
☐	S19	AB ("renal insufficiency" or "serious physical illness") OR AB (Chronic obstructive pulmonary disease or COPD) OR AB "chronic medical condition"	Search modes - Boolean/Phrase	View Results (14,172) View Details Edit
☐	S18	AB ("Enzymatic disease*" or "enzyme disease*") OR AB ("duchenne muscular dystrophy" or "amyotrophic lateral sclerosis" or ALS) OR AB ("Genetic disease" or "genetic condition") OR AB ("cystis fibrosis" or CF) OR AB ("Organ failure" or "chronic organ failure")	Search modes - Boolean/Phrase	View Results (12,244) View Details Edit
☐	S17	AB cancer* or tumo?r* or neoplas* or malignan* or carcinoma* or adenocarcinoma* or adeno?carcinoma* or choriocrcinoma* or leukemia* or leukaemia* or metastat* or sarcoma* or teratoma* or lymphoma	Search modes - Boolean/Phrase	View Results (274,177) View

For peer review only

				Details<br>Edit
☐	S16	(MH "Neoplasms")	Search modes - Boolean/Phrase	View Results (67,564)<br>View Details<br>Edit
☐	S15	S1 OR S2 OR S3 OR S4 OR S5 OR S6 OR S7 OR S8 OR S9 OR S10 OR S11 OR S12 OR S13 OR S14	Search modes - Boolean/Phrase	View Results (159,654)<br>View Details<br>Edit
☐	S14	AB withdraw* N3 treat* OR AB withdraw* N3 car* OR AB withdraw* N3 intervene* OR AB withdraw* N3 therap* OR AB withdraw* N3 dialysis OR AB withdraw* N3 transplant*	Search modes - Boolean/Phrase	View Results (2,671)<br>View Details<br>Edit
☐	S13	AB withhold* N3 treat* OR AB withhold* N3 car* OR AB withhold* N3 intervene* OR AB withhold* N3 therap* OR AB withhold* N3 dialysis OR AB withhold* N3 transplant*	Search modes - Boolean/Phrase	View Results (561)<br>View Details<br>Edit
☐	S12	AB withdrew N3 treat* OR AB withdrew N3 car* OR AB withdrew N3 intervene* OR AB withdrew N3 dialysis OR AB withdrew N3 transplant* or AB withdrew N3 therap*	Search modes - Boolean/Phrase	View Results (185)<br>View Details<br>Edit
☐	S11	TX Refus* N3 treat* OR TX Refus* N3 care OR TX Refus* N3 intervention OR TX Refus* N3	Search modes - Boolean/Phrase	View Results (13,309)<br>View Details<br>Edit
☐	S10	TX conservative N2 treatment OR TX conservative N2 management OR TX "Withholding Treatment" OR TX "Treatment Refusal"	Search modes - Boolean/Phrase	View Results (20,188)<br>View Details<br>Edit
☐	S9	TX "Dying soon" or "expected death" or "imminently dying" or Moribund	Search modes - Boolean/Phrase	View Results (1,442)<br>View Details<br>Edit

For peer review only

☐	S8	TX ("end-stage disease*" or end stage disease* or end-stage ill* or "end stage ill*" or end-stage or "end stage") OR AB expected N3 die OR AB imminent N3 death	Search modes - Boolean/Phrase	View Results (23,677) View Details Edit
☐	S7	TX ("Irreversible condition" or "terminal condition" or "fatal illness") OR TX ("last year of life" or LYOL or "end of life" or "end of their lives") or TX ("last six months of life" or "last 6 months of life")	Search modes - Boolean/Phrase	View Results (37,619) View Details Edit
☐	S6	AB terminal* N3 care OR AB terminal* N3 caring OR AB terminal* N3 ill*	Search modes - Boolean/Phrase	View Results (3,888) View Details Edit
☐	S5	AB hospice N3 care OR AB hospice N3 caring OR AB hospice N3 ill*	Search modes - Boolean/Phrase	View Results (3,428) View Details Edit
☐	S4	TX "palliative care" or hospice or "end of life care" or end-of-life	Search modes - Boolean/Phrase	View Results (104,201) View Details Edit
☐	S3	(MH "Terminal Care") OR (MH "Terminally Ill Patients")	Search modes - Boolean/Phrase	View Results (23,777) View Details Edit
☐	S2	(MH "Hospice Care")	Search modes - Boolean/Phrase	View Results (7,894) View Details Edit
☐	S1	(MH "Palliative Care")	Search modes - Boolean/Phrase	

Search Name: COCHRANE Menloc final

Last Saved: 13/12/2018 09:55:54

Comment: after editing

For peer review only

ID	Search
#1	MeSH descriptor: [Palliative Care] explode all trees
#2	MeSH descriptor: [Hospice Care] explode all trees
#3	MeSH descriptor: [Terminal Care] this term only
#4	MeSH descriptor: [Terminally Ill] this term only
#5	"palliative care" or hospice or "end of life care" or end-of-life
#6	"Irreversible condition" or "terminal condition" or "fatal illness"
#7	"last year of life" or LYOL or "end of life" or "end of their lives" or "last six months of life" or "last 6 months of life"
#8	end-stage disease* or end stage disease* or end-stage ill* or end stage ill* or end-stage or "end stage"
#9	imminent NEAR/3 death
#10	expected NEAR/3 die
#11	MeSH descriptor: [Conservative Treatment] this term only
#12	conservative NEAR/2 management
#13	conservative NEAR/2 treatment
#14	MeSH descriptor: [Withholding Treatment] explode all trees
#15	MeSH descriptor: [Treatment Refusal] explode all trees
#16	withdrew NEAR/3 treat*
#17	Withdrew NEAR/3 car*
#18	Withdrew NEAR/3 intervene*
#19	Withdrew NEAR/3 therap*
#20	Withdrew NEAR/3 dialysis
#21	#1 or #2 #3 or #4 or #5 or #6 or #7 or #8 or #9 or #10 or #11 or #12 or #13 or #14 or #15 or #16 or #17 or #18 or #19 or 20
#22	cancer* or tumo?r* or neoplas* or malignan* or carcinoma* or adenocarcinoma* or adeno?carcinoma* or choriocrcinoma* or leukemia* or leukaemia* or metastat* or sarcoma* or teratoma* or lymphoma
#23	MeSH descriptor: [Neoplasms] explode all trees
#24	MeSH descriptor: [Amyotrophic Lateral Sclerosis] explode all trees
#25	MeSH descriptor: [Genetic Diseases, Inborn] explode all trees
#26	MeSH descriptor: [Muscular Dystrophy, Duchenne] explode all trees

For peer review only

#27 MeSH descriptor: [Cystic Fibrosis] explode all trees

#28 MeSH descriptor: [Multiple Organ Failure] explode all trees

#29 MeSH descriptor: [Pulmonary Disease, Chronic Obstructive] explode all trees

#30 MeSH descriptor: [Heart Failure] explode all trees

#31 MeSH descriptor: [Renal Insufficiency] explode all trees

#32 MeSH descriptor: [Liver Failure] explode all trees

#33 MeSH descriptor: [Chronic Disease] explode all trees

#34 "duchenne muscular dystrophy" or "amyotrophic lateral sclerosis" or ALS

#35 "Genetic disease" or "genetic condition"

#36 chronic NEAR/2 ESRD

#37 "chronic illness" or "chronic condition" or "chronic disease*"

#38 "chronic medical condition"

#39 #22 or #23 or #24 or #25 or #26 or #27 or #28 or #29 or #30 or #31 or #32 or #33 or #34 or #35 or #36 or #37 or #38

#40 "severe mental illness" or "persistent mental illness"

#41 schizo* or "mood disorder*" or "personality disorder*" or psychotic* or psychosis or psychoses or SPMI or SMI

#42 bipolar or mania or Schizophrenia or psychosis

#43 Depression NEAR/2 psychosis

#44 Depression NEAR/2 psychotic

#45 Depression NEAR/2 severe

#46 Bipolar or "Mental Disorders" or "Personality Disorders"

#47 "psychotic disorders" or "schizoaffective disorder"

#48 "severe and persistent mental illness".

#49 #40 or #41 or #42 or #43 or #44 or #45 or #46 or #47 or #48

#50 #21 and #39 and #49

For peer review only

DARE 1998-2018

DARE and NHS EED archives secure on CRD website until at least 2021

("palliative care" or hospice or "end of life care" or end-of-life) and "severe mental illness" or " persistent mental illness".

Database: EMBASE <1947-Present>

Search Strategy:

----

1 exp palliative therapy/ (102069)

2 exp hospice care/ (9228)

3 exp terminal care/ (63131)

4 exp terminally ill patient/ (8148)

5 ("palliative care" or hospice or "end of life care" or end-of-life).tw. (65572)

6 ((hospice or terminal*) adj3 (care or caring or ill*)).tw. (17540)

7 ("Irreversible condition" or "terminal condition" or fatal illness).tw. (1173)

8 ("last year of life" or LYOL or "end of life" or "end of their lives" or "last six months of life" or "last 6 months of life").tw. (28590)

9 (end-stage disease* or end stage disease* or end-stage ill* or end stage ill* or end-stage or end stage).tw. (88414)

10 (expected adj3 die).tw. (306)

11 (imminent adj3 death).tw. (870)

12 ("Dying soon" or "expected death" or "imminently dying" or Moribund).tw. (3793)

13 conservative treatment/ (78244)

14 (conservative adj2 (treatment or management)).tw. (64421)

15 treatment withdrawal/ (17413)

16 treatment refusal/ (17138)

17 (Refus* adj3 (treat* or care or intervention or dialysis)).tw. (7265)

18 ((withdrew or withdraw* or withhold*) adj3 (treat* or car* or intervene* or therap* or dialysis or transplant*)).tw. (23578)

For peer review only

19 or/1-18 (420975)

20 exp neoplasm/ (4447791)

21 (cancer* or tumo?r* or neoplas* or malignan* or carcinoma* or adenocarcinoma* or adeno?carcinoma* or choriocrcinoma* or leukemia* or leukaemia* or metastat* or sarcoma* or teratoma* or lymphoma).tw. (4510708)

22 ("Enzymatic disease*" or "enzyme disease*").tw. (45)

23 genetic disorder/ (55815)

24 ("duchenne muscular dystrophy" or "amyotrophic lateral sclerosis" or ALS).tw. (55394)

25 amyotrophic lateral sclerosis/ (34903)

26 Duchenne muscular dystrophy/ (14824)

27 ("Genetic disease" or "genetic condition").tw. (10518)

28 cystic fibrosis/ (68731)

29 ("cystis fibrosis" or CF).tw. (62550)

30 exp multiple organ failure/ (35190)

31 ("Organ failure" or "chronic organ failure").tw. (27179)

32 chronic obstructive lung disease/ (115273)

33 ("renal insufficiency" or "serious physical illness").tw. (32224)

34 (Chronic obstructive pulmonary disease or COPD).tw. (93906)

35 (chronic adj3 (illness or condition or disease*)).tw. (374959)

36 "chronic medical condition".tw. (563)

37 exp kidney failure/ (343470)

38 exp heart failure/ (474239)

39 exp liver failure/ (70302)

40 (chronic adj2 ("cardiac failure" or "liver failure" or "kidney failure" or "end-stage renal disease" or ESRD or "renal failure" or "heart failure")).tw. (67182)

41 exp chronic disease/ (187803)

42 lifestyle-related factor*.mp. (445)

43 or/20-42 (6880626)

44 exp bipolar disorder/ (58962)

45 exp schizophrenia/ (184860)

46 (schizo* or "mood disorder*" or "personality disorder*" or psychotic* or psychosis or psychoses).tw. (279001)

For peer review only

47 (bipolar or mania or Schizophrenia).tw. (216783)

48 (Depression adj2 (psychosis or psychotic or severe or major)).tw. (39961)

49 exp mental disease/ (2123169)

50 mental health condition*.tw. (2502)

51 exp personality disorder/ (60988)

52 exp psychosis/ (290561)

53 exp schizoaffective psychosis/ (9378)

54 exp paranoid psychosis/ (18067)

55 "severe mental illness".tw. (4748)

56 " persistent mental illness".tw. (514)

57 ((chronic* or sever* or serious or persistent* or enduring or debilitating) adj2 (mental* or psychological*) adj2 (ill* or disorder* or health)).mp. (16645)

58 mental patient/ (27553)

59 (SPMI or SMI).tw. (5581)

60 or/44-59 (2207382)

61 (dementia or Alzheimer).ti. (70245)

62 (bipolar electrocoagulation or bipolar radiofrequency or bipolar tumour probe or bipolar diathermy).tw. (1641)

63 ("respiratory depression" or "marrow depression" or "hematologic* depression").tw. (10545)

64 (child* or adoles* or pediatric or paediatric).tw. (2085814)

65 (Algeria$ or Egypt$ or Liby$ or Morocc$ or Tunisia$ or Western Sahara$ or Angola$ or Benin or Botswana$ or Burkina Faso or Burundi or Cameroon or Cape Verde or Central African Republic or Chad or Comoros or Congo or Djibouti or Eritrea or Ethiopia$ or Gabon or Gambia$ or Ghana or Guinea or Keny$ or Lesotho or Liberia or Madagasca$ or Malawi or Mali or Mauritania or Mauritius or Mayotte or Mozambiq$ or Namibia$ or Niger or Nigeria$ or Reunion or Rwand$ or Saint Helena or Senegal or Seychelles or Sierra Leone or Somalia or South Africa$ or Sudan or Swaziland or Tanzania or Togo or Ugand$ or Zambia$ or Zimbabw$ or China or Chinese or Hong Kong or Macao or Mongolia$ or Taiwan$ or Belarus or Moldov$ or Russia$ or Ukraine or Afghanistan or Armenia$ or Azerbaijan or Bahrain or Cyprus or Cypriot or Georgia$ or Iran$ or Iraq$ or Jordan$ or Kazakhstan or Kuwait or Kyrgyzstan or Leban$ or Oman or Pakistan$ or Palestin$ or Qatar or Saudi Arabia or Syria$ or Tajikistan or Turkmenistan or United Arab Emirates or Uzbekistan or Yemen or Bangladesh$ or Bhutan or British Indian Ocean Territory or Brunei Darussalam or Cambodia$ or India$ or Indonesia$ or Lao or People's Democratic Republic or Malaysia$ or Maldives or Myanmar or Nepal or Philippin$ or Singapore or Sri Lanka or Thai$ or Timor Leste or Vietnam or Albania$ or Andorra or Bosnia$ or Herzegovina$ or Bulgaria$ or Croatia$ or Faroe Islands or Greenland or Liechtenstein or Lithuani$ or Macedonia or Malta or maltese or Romania or Serbia$ or Montenegro or Svalbard or Argentina$ or Belize or Bolivia$ or Brazil$ or Chilean or Colombia$ or Costa Rica$ or Cuba or Ecuador or El Salvador or French Guiana or Guatemala$ or Guyana or Haiti or Honduras or Jamaica$ or Nicaragua$ or

For peer review only

Panama or Paraguay or Peru or Puerto Rico or Suriname or Uruguay or Venezuela or developing countr$ or south America$).ti,sh. (1653512)

66 scientific literature/ (30246)

67 thesis.tw. (11296)

68 book.pt. (1180)

69 book/ (41676)

70 or/61-69 (3683087)

71 19 and 43 and 60 (13644)

72 71 not 70 (11727)

73 limit 72 to (human and english language and (adult <18 to 64 years> or aged <65+ years>)) (5427)

Database: Ovid MEDLINE(R) ALL <1946 to December 06, 2018>

Search Strategy:

----

1 exp Palliative care/ (49655)

2 exp Hospice care/ (5949)

3 exp Terminal Care/ (48040)

4 exp Terminally ill/ (6227)

5 ("palliative care" or hospice or "end of life care" or end-of-life).tw. (43896)

6 ((hospice or terminal*) adj3 (care or caring or ill*)).tw. (12766)

7 ("Irreversible condition" or "terminal condition" or fatal illness).tw. (791)

8 ("last year of life" or LYOL or "end of life" or "end of their lives" or "last six months of life" or "last 6 months of life").tw. (19924)

9 (end-stage disease* or end stage disease* or end-stage ill* or end stage ill* or end-stage or end stage).tw. (60633)

10 (expected adj3 die).tw. (215)

11 (imminent adj3 death).tw. (561)

12 ("Dying soon" or "expected death" or "imminently dying" or Moribund).tw. (2493)

13 conservative treatment/ (1401)

14 (conservative adj2 (treatment or management)).tw. (42831)

15 Withholding Treatment/ (10909)

16 Treatment Refusal/ (11477)

17 (Refus* adj3 (treat* or care or intervention or dialysis)).tw. (4855)

18 ((withdrew or withdraw* or withhold*) adj3 (treat* or car* or intervene* or therap* or dialysis or transplant*)).tw. (15455)

19 or/1-18 (244771)

20 exp Neoplasms/ (3107101)

21 (cancer* or tumo?r* or neoplas* or malignan* or carcinoma* or adenocarcinoma* or adeno?carcinoma* or choriocrcinoma* or leukemia* or leukaemia* or metastat* or sarcoma* or teratoma* or lymphoma).tw. (3243976)

22 ("Enzymatic disease*" or "enzyme disease*").tw. (25)

23 Genetic Disease, Inborn/ (13207)

24 ("duchenne muscular dystrophy" or "amyotrophic lateral sclerosis" or ALS).tw. (40207)

25 amyotrophic lateral sclerosis/ (16921)

26 Muscular dystrophy, Duchenne/ (4706)

27 ("Genetic disease" or "genetic condition").tw. (7305)

28 Cystic Fibrosis/ (33150)

29 ("cystis fibrosis" or CF).tw. (36968)

30 exp Multiple Organ Failure/ (10209)

31 ("Organ failure" or "chronic organ failure").tw. (17137)

32 Pulmonary Disease, Chronic Obstructive/ (34379)

33 ("renal insufficiency" or "serious physical illness").tw. (21134)

34 (Chronic obstructive pulmonary disease or COPD).tw. (54881)

35 (chronic adj3 (illness or condition or disease*)).tw. (250692)

36 "chronic medical condition".tw. (382)

37 Heart Failure/ (107446)

38 Renal Insufficiency/ (14803)

39 Liver Failure/ (6828)

40 (chronic adj2 ("cardiac failure" or "liver failure" or "kidney failure" or "end-stage renal disease" or ESRD or "renal failure" or "heart failure")).tw. (44143)

41 Chronic Disease/ (251517)

For peer review only

42 Health service utilization.mp. (1569)

43 lifestyle-related factor*.mp. (354)

44 or/20-43 (4717135)

45 exp Bipolar Disorder/ (37764)

46 exp Schizophrenia/ (99011)

47 (schizo* or "mood disorder*" or "personality disorder*" or psychotic* or psychosis or psychoses).tw. (197049)

48 (bipolar or mania or Schizophrenia).tw. (151754)

49 (Depression adj2 (psychosis or psychotic or severe or major)).tw. (28840)

50 exp Mental Disorders/ (1143274)

51 mental health condition*.tw. (1954)

52 exp Personality Disorders/ (39433)

53 exp psychotic disorders/ (48805)

54 exp schizoaffective disorder/ (48805)

55 exp psychosis/ (48805)

56 Paranoid Disorders/ (3987)

57 ("severe mental illness" or "persistent mental illness").tw. (3969)

58 ((chronic* or sever* or serious or persistent* or enduring or debilitating) adj2 (mental* or psychological*) adj2 (ill* or disorder* or health)).tw. (12556)

59 exp Mentally Ill Persons/ (5926)

60 (SPMI or SMI).tw. (3830)

61 or/45-60 (1239759)

62 (dementia or Alzheimer).ti. (52380)

63 (bipolar electrocoagulation or bipolar radiofrequency or bipolar tumour probe or bipolar diathermy).tw. (1123)

64 ("respiratory depression" or "marrow depression" or "hematologic* depression").tw. (6399)

65 (child* or adoles* or pediatric or paediatric).tw. (1520041)

66 (Algeria$ or Egypt$ or Liby$ or Morocc$ or Tunisia$ or Western Sahara$ or Angola$ or Benin or Botswana$ or Burkina Faso or Burundi or Cameroon or Cape Verde or Central African Republic or Chad or Comoros or Congo or Djibouti or Eritrea or Ethiopia$ or Gabon or Gambia$ or Ghana or Guinea or Keny$ or Lesotho or Liberia or Madagasca$ or Malawi or Mali or Mauritania or Mauritius or Mayotte or Mozambiq$ or Namibia$ or Niger or Nigeria$ or Reunion or Rwand$ or Saint Helena or Senegal or Seychelles or Sierra Leone or Somalia or South Africa$ or Sudan or Swaziland or Tanzania or Togo or Ugand$ or Zambia$ or Zimbabw$ or China or Chinese or Hong Kong or Macao or

For peer review only

Mongolia$ or Taiwan$ or Belarus or Moldov$ or Russia$ or Ukraine or Afghanistan or Armenia$ or Azerbaijan or Bahrain or Cyprus or Cypriot or Georgia$ or Iran$ or Iraq$ or Jordan$ or Kazakhstan or Kuwait or Kyrgyzstan or Leban$ or Oman or Pakistan$ or Palestin$ or Qatar or Saudi Arabia or Syria$ or Tajikistan or Turkmenistan or United Arab Emirates or Uzbekistan or Yemen or Bangladesh$ or Bhutan or British Indian Ocean Territory or Brunei Darussalam or Cambodia$ or India$ or Indonesia$ or Lao or People's Democratic Republic or Malaysia$ or Maldives or Myanmar or Nepal or Philippin$ or Singapore or Sri Lanka or Thai$ or Timor Leste or Vietnam or Albania$ or Andorra or Bosnia$ or Herzegovina$ or Bulgaria$ or Croatia$ or Faroe Islands or Greenland or Liechtenstein or Lithuani$ or Macedonia or Malta or maltese or Romania or Serbia$ or Montenegro or Svalbard or Argentina$ or Belize or Bolivia$ or Brazil$ or Chilean or Colombia$ or Costa Rica$ or Cuba or Ecuador or El Salvador or French Guiana or Guatemala$ or Guyana or Haiti or Honduras or Jamaica$ or Nicaragua$ or Panama or Paraguay or Peru or Puerto Rico or Suriname or Uruguay or Venezuela or developing countr$ or south America$).ti,sh. (1225437)

67 Academic Dissertations/ (0)

68 thesis.tw. (7167)

69 book.pt. (0)

70 Books/ (3174)

71 or/62-70 (2653676)

72 19 and 44 and 61 (2434)

73 72 not 71 (2126)

74 limit 73 to (english language and humans and "all adult (19 plus years)") (1217)

Database: PsycINFO <1806 to December Week 1 2018>

Search Strategy:

----

1 exp Palliative Care/ (11016)

2 Hospice/ (3062)

3 exp Terminally Ill Patients/ (4562)

4 exp HOSPICE/ (3062)

5 ("palliative care" or "hospice care" or "end of life care" or end-of-life).tw. (15589)

6 ((hospice or terminal*) adj3 (care or caring or ill*)).tw. (6333)

7 ("Irreversible condition" or "terminal condition" or fatal illness).tw. (216)

8 ("last year of life" or LYOL or "end of life" or "end of their lives" or "last six months of life" or "last 6 months of life").tw. (9038)

For peer review only

9 (end-stage disease* or end stage disease* or end-stage ill* or end stage ill* or end-stage or end stage).tw. (2002)

10 (expected adj3 die).tw. (44)

11 (imminent adj3 death).tw. (272)

12 ("Dying soon" or "expected death" or "imminently dying" or Moribund).tw. (235)

13 (conservative adj2 (treatment or management)).tw. (460)

14 Treatment Withholding/ (462)

15 Treatment Refusal/ (730)

16 (Refus* adj3 (treat* or care or intervention or dialysis)).tw. (1811)

17 ((withdrew or withdraw* or withhold*) adj3 (treat* or car* or intervene* or therap* or dialysis or transplant*)).tw. (3579)

18 or/1-17 (28620)

19 exp NEOPLASMS/ (47829)

20 (cancer* or tumo?r* or neoplas* or malignan* or carcinoma* or adenocarcinoma* or adeno?carcinoma* or choriocrcinoma* or leukemia* or leukaemia* or metastat* or sarcoma* or teratoma* or lymphoma).tw. (76722)

21 ("Enzymatic disease*" or "enzyme disease*").tw. (0)

22 Genetic Disorders/ (4048)

23 ("duchenne muscular dystrophy" or "amyotrophic lateral sclerosis" or ALS).tw. (13477)

24 Amyotrophic Lateral Sclerosis/ (3551)

25 Muscular Dystrophy/ (1293)

26 ("Genetic disease" or "genetic condition").tw. (611)

27 Cystic Fibrosis/ (854)

28 ("cystis fibrosis" or CF).tw. (3217)

29 "Multi* Organ Failure*".tw. (71)

30 ("Organ failure" or "chronic organ failure").tw. (203)

31 exp Chronic Obstructive Pulmonary Disease/ (1272)

32 ("renal insufficiency" or "serious physical illness").tw. (319)

33 (Chronic obstructive pulmonary disease or COPD).tw. (2277)

34 (chronic adj3 (illness or condition or disease*)).tw. (28394)

35 "chronic medical condition".tw. (196)

36 Heart Disorders/ (8989)

For peer review only

37 Kidney Diseases/ (2001)

38 Liver Disorders/ (1145)

39 (chronic adj2 ("cardiac failure" or "liver failure" or "kidney failure" or "end-stage renal disease" or ESRD or "renal failure" or "heart failure")).tw. (866)

40 Chronic Illness/ (10804)

41 lifestyle-related factor*.mp. (51)

42 or/19-41 (139563)

43 exp Bipolar Disorder/ (25413)

44 exp SCHIZOPHRENIA/ (85758)

45 (schizo* or "mood disorder*" or "personality disorder*" or psychotic* or psychosis or psychoses).tw. (208100)

46 (bipolar or mania or Schizophrenia).tw. (137089)

47 (Depression adj2 (psychosis or psychotic or severe or major)).tw. (34922)

48 exp Mental Disorders/ (566262)

49 mental health condition*.tw. (1698)

50 exp Personality Disorders/ (33638)

51 exp Schizoaffective Disorder/ (2961)

52 exp Psychosis/ (109879)

53 "Paranoia (Psychosis)"/ (1215)

54 "Severity (Disorders)"/ (15885)

55 "severe mental illness".tw. (4681)

56 "persistent mental illness".tw. (799)

57 ((chronic* or sever* or serious or persistent* or enduring or debilitating) adj2 (mental* or psychological*) adj2 (ill* or disorder* or health)).tw. (16725)

58 Mentally Ill Persons.mp. (805)

59 (SPMI or SMI).tw. (1991)

60 or/43-59 (637058)

61 (dementia or Alzheimer).ti. (30336)

62 (bipolar electrocoagulation or bipolar radiofrequency or bipolar tumour probe or bipolar diathermy).tw. (5)

63 ("respiratory depression" or "marrow depression" or "hematologic* depression").tw. (407)

64 (child* or adoles* or pediatric or paediatric).tw. (811926)

For peer review only

65 (Algeria$ or Egypt$ or Liby$ or Morocc$ or Tunisia$ or Western Sahara$ or Angola$ or Benin or Botswana$ or Burkina Faso or Burundi or Cameroon or Cape Verde or Central African Republic or Chad or Comoros or Congo or Djibouti or Eritrea or Ethiopia$ or Gabon or Gambia$ or Ghana or Guinea or Keny$ or Lesotho or Liberia or Madagasca$ or Malawi or Mali or Mauritania or Mauritius or Mayotte or Mozambiq$ or Namibia$ or Niger or Nigeria$ or Reunion or Rwand$ or Saint Helena or Senegal or Seychelles or Sierra Leone or Somalia or South Africa$ or Sudan or Swaziland or Tanzania or Togo or Ugand$ or Zambia$ or Zimbabw$ or China or Chinese or Hong Kong or Macao or Mongolia$ or Taiwan$ or Belarus or Moldov$ or Russia$ or Ukraine or Afghanistan or Armenia$ or Azerbaijan or Bahrain or Cyprus or Cypriot or Georgia$ or Iran$ or Iraq$ or Jordan$ or Kazakhstan or Kuwait or Kyrgyzstan or Leban$ or Oman or Pakistan$ or Palestin$ or Qatar or Saudi Arabia or Syria$ or Tajikistan or Turkmenistan or United Arab Emirates or Uzbekistan or Yemen or Bangladesh$ or Bhutan or British Indian Ocean Territory or Brunei Darussalam or Cambodia$ or India$ or Indonesia$ or Lao or People's Democratic Republic or Malaysia$ or Maldives or Myanmar or Nepal or Philippin$ or Singapore or Sri Lanka or Thai$ or Timor Leste or Vietnam or Albania$ or Andorra or Bosnia$ or Herzegovina$ or Bulgaria$ or Croatia$ or Faroe Islands or Greenland or Liechtenstein or Lithuani$ or Macedonia or Malta or maltese or Romania or Serbia$ or Montenegro or Svalbard or Argentina$ or Belize or Bolivia$ or Brazil$ or Chilean or Colombia$ or Costa Rica$ or Cuba or Ecuador or El Salvador or French Guiana or Guatemala$ or Guyana or Haiti or Honduras or Jamaica$ or Nicaragua$ or Panama or Paraguay or Peru or Puerto Rico or Suriname or Uruguay or Venezuela or developing countr$ or south America$).ti,sh. (128851)

66 thesis.tw. (24537)

67 book.pt. (476469)

68 exp BOOKS/ (6110)

69 or/61-68 (1332511)

70 18 and 42 and 60 (838)

71 70 not 69 (609)

# 11	105	#10 AND #7 AND #6<br>Indexes=SCI-EXPANDED, SSCI, CPCI-S, CPCI-SSH<br>Timespan=All years
# 10	394,440	#9 OR #8<br>Indexes=SCI-EXPANDED, SSCI, CPCI-S, CPCI-SSH<br>Timespan=All years
# 9	388,426	TS=(Bipolar or schizo* or "mood disorder*" or "personality disorder*" or psychotic* or psychosis or psychoses) OR TS=("psychotic disorders" or "schizoaffective disorder" or "severe and persistent mental illness")<br>Indexes=SCI-EXPANDED, SSCI, CPCI-S, CPCI-SSH<br>Timespan=All years
# 8	22,785	**TOPIC:** ("schizoaffective disorder" or "severe and persistent mental illness" OR "psychotic disorders" or "schizoaffective disorder" or "Paranoid Disorders" OR "severe mental illness" or "persistent mental illness" or SPMI or SMI)

		Indexes=SCI-EXPANDED, SSCI, CPCI-S, CPCI-SSH Timespan=All years
# 7	4,104,078	**TOPIC:** (Neoplasm* or cancer* or tumor* or tumour or neoplas* or malignan* or carcinoma* or adenocarcinoma* or choriocrcinoma* or leukemia* or leukaemia* or metastat* or sarcoma* or teratoma* or lymphoma) *OR* **TOPIC:** ("duchenne muscular dystrophy" or "amyotrophic lateral sclerosis" or ALS) *OR* **TOPIC:** ("Genetic disease" or "genetic condition" or "cystis fibrosis" or CF) *OR* **TOPIC:** ("Organ failure" or "chronic organ failure" or "mulitple organ failure") *OR* **TOPIC:** (Chronic obstructive pulmonary disease or COPD or "renal insufficiency" or "serious physical illness" or "chronic medical condition") Indexes=SCI-EXPANDED, SSCI, CPCI-S, CPCI-SSH Timespan=All years
# 6	109,890	#5 OR #4 OR #3 OR #2 OR #1 Indexes=SCI-EXPANDED, SSCI, CPCI-S, CPCI-SSH Timespan=All years
# 5	2,468	**TOPIC:** ("Dying soon" or "expected death" or "imminently dying" or Moribund) Indexes=SCI-EXPANDED, SSCI, CPCI-S, CPCI-SSH Timespan=All years
# 4	56,678	**TOPIC:** ("end-stage disease*" or "end stage disease*" or "end-stage ill*" or "end stage ill*" or end-stage or “end stage”) Indexes=SCI-EXPANDED, SSCI, CPCI-S, CPCI-SSH Timespan=All years
# 3	24,428	**TOPIC:** ("last year of life" or LYOL or "end of life" or "end of their lives" or "last six months of life" or "last 6 months of life") Indexes=SCI-EXPANDED, SSCI, CPCI-S, CPCI-SSH Timespan=All years
# 2	773	**TOPIC:** ("Irreversible condition" or "terminal condition" or "fatal illness") Indexes=SCI-EXPANDED, SSCI, CPCI-S, CPCI-SSH Timespan=All years
# 1	54,014	**TOPIC:** ("palliative care" or hospice or "end of life care" or end-of-life or "terminal care") Indexes=SCI-EXPANDED, SSCI, A&HCI, CPCI-S, CPCI-SSH, ESCI Timespan=All years

Other sources

Trial Registers	Search terms
ClinicalTrials.gov	"end of life" or palliative and mental
metaRegister of Controlled Trials (mRCT)	Not Applicable
UK Clinical Trials Gateway (UKCTG)	for "last year of life" or LYOL or "end of life" or "end of their lives" in mental
WHO ICTRP Search Portal International	"end of life" or "end of their lives" and

For peer review only

	mental

Charity websites	Search terms
Cancer Research UK	**"end of life" and "mental illness"**
Cancer Research Wales	"end of life" and "mental illness"
Tenovus Cancer Care	"end of life" and "mental illness"
Mental Health Foundation	Reports identified browsing publications page
Centre for Mental Health	Searched publications – Mental health Policy https://www.centreformentalhealth.org.uk/publications
National Kidney Foundation	**"end of life" and "mental illness"**
British Liver Trust	**"end of life" and "mental illness"**
British Renal Society	Scanning BRS conference abstract page
The Renal Association	**"end of life" and "mental illness"**
British Heart Foundation	**"last year of life" or LYOL or "end of life" or "end of their lives" and "severe mental" and Healthcare professionals**
British Lung Foundation	**"end of life" and "mental illness"**
National Council for Palliative Care	"end of life care" and mental
Hospice UK	"end of life care" and mental
Marie Curie	"end of life care" and "mental illness" and evaluation
Macmillan Cancer Support	**"last year of life" or LYOL or "end of life" or "end of their lives" and "severe mental"**
Scottish Partnership agency for Palliative Care	"end of life care" and "mental illness" and evaluation
Bipolar UK	"end of life care" and "mental illness" and evaluation
MIND	"end of life care" and "mental illness"
Rethink Mental Illness	"end of life care" and "mental illness"
Sova	"end of life care" and mental
Mental Health UK	No search facility or publications page
Heads Together	No search facility or publications page

Organisation Websites	Search terms
NHS England	"end of life care" and "mental illness" and evaluation
NHS Wales	"end of life care" and "severe mental" and evaluation
Care Inspectorate Wales	"end of life care" and "severe mental"
English CQC	**"end of life" and "severe mental" in Publications**
Ministry of Justice	"end of life care" and "mental illness" and evaluation

Journals	Search terms

For peer review only

Journal of Pain and Symptom Management	**"end of life care"** in *Title/Abs/Keywords* OR **"palliative care"** in *Title/Abs/Keywords* AND **Mental** in *Title/Abs/Keywords*<br>2017-2019
Cancer	"end of life" and mental or palliative and mental 2017-2019
Psycho-Oncology	"end of life" and mental (2017-2019) or palliative and mental (2017-2018)
BMJ Supportive & Palliative Care	""mental illness"" and published between "01 Jan, 2017 and 21 Feb, 2019""mental" and published between "01 Jan, 2017 and 21 Feb, 2019

For peer review only

Supplementary searches

Trial Registers	Search terms
ClinicalTrials.gov	"end of life" or palliative and mental
metaRegister of Controlled Trials (mRCT)	Not Applicable
UK Clinical Trials Gateway (UKCTG)	"last year of life" or LYOL or "end of life" or "end of their lives" in mental
WHO ICTRP Search Portal International<br>https://www.who.int/ictrp/en/	"end of life" or "end of their lives" and mental

Charity websites	Search type	Search terms
1. Cancer Research UK http://www.cancerresearchuk.org/	Electronic search: Main website	"end of life" and "mental illness/health"<br>"palliative care" and mental illness/health"
2. Cancer Research Wales http://www.cancerresearchuk.org/	Electronic search: Main website	"end of life" and "mental illness/health"<br>"palliative care" and mental illness/health"
3. Tenovus Cancer Care http://www.tenovuscancercare.org.uk/	Electronic search: Main website	"end of life" and "mental illness"
4. Mental Health Foundation https://www.mentalhealth.org.uk/	Electronic search: Main website<br><br>Manual search: Policy publications	"end of life" and "mental illness/health"<br>"palliative care" and mental illness/health"<br>As per reviews inclusion criteria
5. Centre for Mental Health https://www.centreformentalhealth.org.uk/	Electronic search: Policy publications	"end of life" and "mental illness/health"

For peer review only

		"palliative care" and mental illness/health"
6. National Kidney Foundation https://www.kidney.org/	Electronic search: Main website<br><br>Manual search: Policy publications	"end of life" and "mental illness/health"<br>"palliative care" and mental illness/health"<br>As per reviews inclusion criteria
7. British Liver Trust https://www.britishlivertrust.org.uk/	Electronic search: Publications	"end of life" and "mental illness"<br>"palliative care" and mental illness"<br>(mental health too broad a term)
8. British Renal Society http://britishrenal.org/	Electronic search: Conference abstracts	"end of life" and "mental illness/health"<br>"palliative care" and mental illness/health"
9. The Renal Association http://britishrenal.org/	Electronic search: Main website	"end of life" and "mental illness/health"<br>"palliative care" and mental illness/health"
10. British Heart Foundation http://britishrenal.org/	Electronic search: Main website	"last year of life" or LYOL or "end of life" or "end of their lives" and "severe mental" and Healthcare professionals<br>"palliative care" and mental illness"<br>(mental health too broad)
11. British Lung Foundation https://www.blf.org.uk/	Electronic search: Main website	"end of life" and "mental illness/health"<br>"palliative care" and mental illness/health"
12. Hospice UK http://www.hospiceuk.org/	Electronic search: Main website	"end of life" and "mental illness/health"<br>"palliative care" and mental illness/health"
13. Marie Curie https://www.mariecurie.org.uk/	Electronic search: Main website	"end of life" and "mental illness/health"<br>"palliative care" and mental illness/health"

	Manual search: Briefings	As per reviews inclusion criteria
14. Macmillan Cancer Support https://www.macmillan.org.uk/	Electronic search: Main website	“last year of life" or LYOL or "end of life" or "end of their lives" and “severe mental” "palliative care" and mental illness/health"
15. Bipolar UK https://www.bipolaruk.org/	Electronic search: Main website	"end of life" and "mental illness/health" Palliative
16. MIND https://www.mind.org.uk/	Electronic search: Main website	"end of life care" and "mental illness/health" Palliative
17. Rethink Mental Illness https://www.rethink.org/	Electronic search: Main website	"end of life care" and "mental illness/health" Palliative
18. Sova https://www.sova.org.uk/	Electronic search: Main website	"end of life care" and mental Palliative
19. Hafal: for recovery for serious mental illness www.hafal.org	Electronic search: Main website	"end of life care" and mental Palliative and mental
20. Age Concern https://www.ageuk.org.uk	Electronic search: Main website	"end of life care " and mental Palliative and mental
21. Salvation Army https://salvationarmy.org.uk	Electronic search: Main website	"end of life care" and mental Palliative and mental
22. Dying Matters https://www.dyingmatters.org/	Electronic search: Main website	"end of life " and mental "palliative care" and mental illness/health"
23. Care Not Killing https://www.carenotkilling.org.uk/	Electronic search: Main website	"end of life care" and then searched within results for the term mental

For peer review only

		"palliative care" and then searched within results for the term mental
24. Northern Ireland Hospice https://www.nihospice.org/	Electronic search: Main website	"end of life care" and mental<br>Palliative
25. Mental Health UK https://www.mentalhealth-uk.org/	No search facility or publications page	
26. Heads Together https://www.headstogether.org.uk/	No search facility or publications page	
27. Kidney Research UK https://www.kidneyresearchuk.org/	Electronic search: Main website	"end of life care" and mental<br>Palliative
28. National Kidney Federation https://www.kidney.org.uk/	Electronic search: Main website	"end of life care" and mental<br>Palliative
29. St Mungos https://www.mungos.org/	Electronic search: Main website	"end of life "<br>Palliative<br>“mental illness”
30. Samaritans https://www.samaritans.org/	Electronic search: Main website	"end of life" and “severe mental”<br>Palliative and “severe mental”
31. Llanmau https://www.llamau.org.uk/	Electronic search: Main website	
32. SSAFA https://www.ssafa.org.uk/	Electronic search: Main website	
33. Community Housing Cymru: https://chcymru.org.uk/	Electronic search: Main website	
34. National Housing Federation: https://www.housing.org.uk/	Electronic search: Main website	
35. Shelter Cymru https://sheltercymru.org.uk/	Electronic search: Main website	

For peer review only

36. Shelter https://www.shelter.org.uk/	Electronic search: Main website	
37. Gofal http://www.gofal.org.uk/	No search function checked projects & services page but no relevant reports.	As per reviews inclusion criteria
38. Compassionate communities https://www.compassionate-communitiesuk.co.uk/	No search function checked projects but no relevant reports	As per reviews inclusion criteria
39. Byw Nawr https://www.dyingmatters.org/wales	Electronic search: Main website	
40. Combat Stress https://www.combatstress.org.uk/	Electronic search: Main website	
41. Royal British Legion https://www.britishlegion.org.uk/	Electronic search: Main website	

Organisation Websites	Search type	Search terms
1. NHS England https://www.england.nhs.uk/cancer/strategy/ https://www.england.nhs.uk/eolc/resources/ - http://endoflifecareambitions.org.uk/	Electronic search: Main website	"mental illness"
2. NHS Wales http://www.wales.nhs.uk/	Electronic search: Main website<br>Advanced search: title field only	"end of life" and "mental illness/health"<br>"palliative care" and mental illness/health"
3. Welsh Government https://gov.wales/	Electronic search: Main website<br>Advanced search<br>Limited to Health and social services	"end of life" and "mental illness/health"<br>"palliative care" and mental illness/health"
4. Department of Health and Social Care https://www.gov.uk/	Electronic search: Publications<br>Limited by:	"end of life" and "mental illness/health"<br>"palliative care" and mental illness/health"

For peer review only

	Department of Health and Social Care<br>Topic: Health and Social Care<br>Sub topic: End of Life	
5. Department of Health: Northern Ireland		"end of life" and "mental illness/health"<br>"palliative care" and mental illness/health"
6. Department of Health, Social Services and Public Safety: Northern Ireland		"end of life" and "mental illness/health"<br>"palliative care" and mental illness/health"
7. Ministry of Justice<br>https://www.gov.uk/	Electronic search: Publications<br>Limited by: Ministry of Justice<br>Topic: Health and Social Care<br>Sub Topic: End of Life	"end of life" and "mental illness/health"<br>"palliative care" and mental illness/health"
8. Care Inspectorate Wales<br>https://www.qcs.co.uk/wales/	Searching only available with practitioner registration<br>Emailed – no relevant publications	
9. Care Quality Commission<br>https://www.cqc.org.uk/	Electronic search: Publications	"end of life" and "severe mental"<br>"palliative care" and "mental illness"
10. Prisons and Probation Ombudsman<br>https://www.ppo.gov.uk	Electronic search: Main website<br>Manual search: Fatal incident reports over a one year period between June 2017-18.<br>Limited by: Deaths of natural causes	"end of life"<br>As per reviews inclusion criteria
11. Royal College of Psychiatrists<br>https://www.rcpsych.ac.uk/	Manual search: College Reports	As per reviews inclusion criteria
12. Royal College of Physicians<br>https://www.rcplondon.ac.uk/	Electronic search: Main website	"end of life" and "mental illness/health"<br>"palliative care" and mental illness/health"
13. The Worldwide Hospice and Palliative Care Alliance	Manual search: Resources	As per reviews inclusion criteria

For peer review only

http://www.thewhpca.org/		
14. European Association of Palliative Care https://www.eapcnet.eu/	Manual search: National Guidelines (UK Countries only)	As per reviews inclusion criteria
15. Scottish Partnership agency for Palliative Care https://www.palliativecarescotland.org.uk/	Electronic search: Main website	"end of life care" and "mental illness" and evaluation<br>"palliative care" and mental illness/health"
16. National Council for Palliative Care http://www.ncpc.org.uk/	Electronic search: Main website	"end of life" and "mental illness/health" "palliative care" and mental illness/health""
17. Social Care Institute for Excellence https://www.scie.org.uk/	Electronic search: Main website	"end of life" and "mental illness" "palliative care" and mental illness"
18. Health Improvement Scotland http://www.healthcareimprovementscotland.org/ Covers publications by NHS Scotland, Scottish Government and Health Improvement Scotland	Electronic search: Main website	"end of life" and "mental illness" "palliative care" and mental illness"
19. National Institute of Clinical Excellence. https://www.nice.org.uk/guidance	Electronic search: Main website	"end of life" or "palliative care" “mental illness"
20. Royal College of Nursing https://www.rcn.org.uk/clinical-topics/end-of-life-care	Manual search through section on Clinical Topics End of Life	As per reviews inclusion criteria
21. Gold Standards Framework http://www.goldstandardsframework.org.uk/	Manual search through library resources	As per reviews inclusion criteria

For peer review only

**Hand searching journals**	**Search terms**
Journal of Pain and Symptom Management	"end of life care" in *Title/Abs/Keywords* OR "palliative care" in *Title/Abs/Keywords* AND Mental in *Title/Abs/Keywords* 2017-2019
Cancer	"end of life" and mental or palliative and mental 2017-2019
Psycho-Oncology	"end of life" and mental (2017-2019) or palliative and mental (2017-2018)
BMJ Supportive & Palliative Care	""mental illness"" and published between "01 Jan, 2017 and 21 Feb, 2019""mental" and published between "01 Jan, 2017 and 21 Feb, 2019

Google search using the terms "palliative care" and "mental illness" (searching first 10 pages of output)

Output	Action	
Naylor et al 2016. Bringing together physical and mental health: A new frontier for integrated care. Available from: https://www.kingsfund.org.uk/publications/physical-and-mental-health	Searched and added to table	Relevant report
Help the Hospices. 2016 Hospice and palliative care – Access for All. Available from: http://nican.hscni.net/files/hospice_and_palliative_care_access_for_all.pdf Accessed 31 January 2019	Searched and added to table	Relevant report
Ulster University Repository<br>Walsh, S. Sheridan, A. Leavey, G. Coughlan, B. Frazer, K. O'Toole, S. Kemple, M. Crawley, L. (2014) Identifying and addressing the palliative care needs of people with serious mental illness in Ireland. Irish College of General Practitioners, 31(7). p.1<br><br>Sheridan, A. Couglan, B. Frazer, K. Walsh, S. Bergin, J. A. Crawley, L. Kemple, M. O'Toole, S. (n.d.)The palliative care needs of people with serious mental illness in Ireland. http://www.professionalpalliativehub.com/sites/default/files/Paliative%20Care%20need%20of%20People%20with%20SMI%20June%2015%202017%20Report%20Draft.pdf	Screened<br><br>Email sent to author 20-02-2019 via researchers gate for date of publication	Relevant paper

For peer review only

NHS Lothian. 2010. Living and dying well in Lothian. Available from: https://www.nhslothian.scot.nhs.uk/OurOrganisation/Strategies/ladwinlothian/Documents/Palliative%20Care%20Strategy%202010%20-15%20VER%2023%20FINAL.pdf	Searched and added to table	Relevant Report
alex.mathew@wales.nhs.uk. End of life care for older people with schizophrenia and bipolar affective disorder along with long term physical conditions: a phenomenological enquiry into the experiences of bereaved family carers, mental health and social care providers in South Wales-UK	Email sent 20th Feb 2019	NOT RELEVANT
Queens University. Belfast Repository Millman, J. Galway, K. Santin, O. Reid J. (2016) Cancer and serious mental illness – patient, caregiver and professional perspectives: study protocol. Journal of Advanced Nursing 72(1), 217–226	Author search conducted on PubMed and full study findings not yet published	NOT RELEVANT
Bloomer, M. J. O'Brien, A. P. (2013) Palliative care for the person with a serious mental illness: The need for a partnership approach to care in Australia, Progress in Palliative Care, 21:1, 27-31	Screened	Not relevant

Google search using the terms "end of life" and "mental illness" (searching first 10 pages of output)

Output	Action	
Blog post Paul Gionfriddo and Nathaniel Counts "Providing end-of-life support for elders with serious mental illnesses" Health Affairs Blog, November 16, 2017.DOI: 10.1377/hblog20171114.475106	Screened	1. Not Relevant
Doering, P. End of life care and serious persistent mental illness. Regional Palliative Care Services. June 2018, Issue 42, p. 1-3 https://www.northernhealth.ca/sites/northern_health/files/health-professionals/palliative-care/documents/serious-persistent-mental-illness-issue-42.pdf	Screened	2. Not relevant
Ahearn 2015. The loneliness of mental illness at the end of life.	Screened	3. Not relevant

For peer review only

https://www.wisconsinmedicalsociety.org/_WMS/publications/wmj/pdf/117/3/101.pdf		
WA Cancer and Palliative Care Network 2018. WA End-of-Life and Palliative Care Strategy 2018–2028. Available from: https://ww2.health.wa.gov.au/~/media/Files/Corporate/general%20documents/Health%20Networks/Palliative%20care/WA%20End-of-life%20and%20Palliative%20Care%20Strategy%202018-2028.pdf	Screened	Not relevant
National Association of Social workers 2004. NASW Standards for Palliative & End of Life Care. Available from: https://www.socialworkers.org/LinkClick.aspx?fileticket=xBMd58VwEhk%3D&portalid=0	Screened	Not relevant
NSW Ministry of Health 2015. Dignity, Respect and Choice: Advance Care Planning for End of Life for People with Mental Illness - A Comprehensive Guide. Available at: https://www.health.nsw.gov.au/patients/acp/Pages/comprehensive-guide.aspx. NSW Government	Screened	Not relevant

Google search using the terms using the terms “end of life” and schizophrenia (searching first 5 pages of output)

Output	Action	
Steves and Williams 2016<br>Enhancing end-of-life care for terminally ill psychiatric patients<br>Nursing, 46(8): 54-8	screened	8. Relevant

Google search using the terms “end of life” and bipolar (searching first 5 pages of output)

Output	Action	
Maloney, K. Opinion: My husband refused to believe he had bipolar disorder, and denial destroyed him. May 15th 2014 https://www.thejournal.ie/readme/bipolar-ii-disorder-denial-mental-health-1467162-May2014/	Screened	Relevant

For peer review only

Journal articles and reports suggested by stakeholder advisory members

Output	Action	
Kurella, M. Kimmel, P. L. Young, B.S. Chertow, G. M.<br>Suicide in the United States end-stage renal disease program. Journal of the American Society of Nephrology. 2005: 16(3): 774-81<br>https://jasn.asnjournals.org/content/16/3/774.full	Screened	1. Not relevant
Liu, C-H. Yeh, M-K. Weng, S-C. Bai, M-Y. Chang, J-C.<br>Suicide and chronic kidney disease: a case-control study. Nephrology Dialysis Transplantation. 2017: 2(9): 1524–29	Screened	2. Not relevant
Prison and Probation Ombudsman for England and Wales 2013<br>Learning from PPO Investigations. End of Life Care	Searched and added to table	3. Not relevant
Prison and Probation Ombudsman for England and Wales 2019<br>Fatal Incident Reports.<br>https://www.ppo.gov.uk/document/fii-report/	Searched through 2 years' worth of reports where death was from natural causes 4th February 2019	4. UNSURE
All Wales Palliative Care Planning Group 2008<br>Report to Minister for Health and Social services on Palliative care Services – Sugar Report<br>www,wales.nhs.uk/documents/palliativecarereport.pdf	Searched and added to table	5. Not relevant
Secretariat of the Commission of the Bishops' Conferences of the European Community 2016<br>Opinion of the Working Group on Ethics in Research and Medicine On Palliative Care in the European Union<br>http://www.comece.eu/dl/psqlJKJKooNNJqx4KJK/PalliativeCARE_EN.pdf	Searched and added to table	6. Not relevant
Addicott, R. Ashton, R. 2010<br>Delivering Better Care at the End of Life: The next steps. The King's Fund	Searched and added to table	7. Not relevant

For peer review only

https://www.kingsfund.org.uk/sites/default/files/Delivering-better-care-end-of-life-Kings-Fund-January-2010-Leeds-Castle-EOLC.pdf		
Addicott, R. Hiley, J. 2011<br>Issues Facing Commissioners in End-of-Life Care. London: The King's Fund<br>https://www.kingsfund.org.uk/sites/default/files/issues-facing-commissioners-end-of-life-care-report-september2011.pdf	Searched and added to table	8. Not relevant
Addicott R, Ross S. 2010<br>Implementing the End of Life Care Strategy. Lessons for Good Practice. The Kings Fund<br>https://www.kingsfund.org.uk/sites/default/files/field/field_publication_file/Implementing-end-of-life-care-Rachael-Addicott-Shilpa-Ross-Kings-Fund-October2010_0.pdf	Searched and added to table	9. Not relevant
General Medical Council 2010<br>Treatment and Care Towards the End of Life: Good Practice in Decision Making<br>https://www.gmc-uk.org/-/media/documents/treatment-and-care-towards-the-end-of-life---english-1015_pdf-48902105.pdf	Searched and added to table	10. Not relevant
Hall, S. Petkova, H. Tsouros, A. D. Costantini, M. Higginson, I. G. 2011<br>Palliative Care for Older People: Better Practices. Copenhagen WHO: Regional Office for Europe.<br>http://www.euro.who.int/__data/assets/pdf_file/0017/143153/e95052.pdf	Searched and added to table	11. Not relevant

For peer review only

THE JOANNA BRIGGS INSTITUTE

JBI Critical Appraisal Checklist for Case Reports

Reviewer	
Date reviewed	
Author	
Year	

	Yes	No	Unclear	Not applicable
1. Were patient's demographic characteristics clearly described?	☐	☐	☐	☐
2. Was the patient's history clearly described and presented as a timeline?	☐	☐	☐	☐
3. Was the current clinical condition of the patient on presentation clearly described?	☐	☐	☐	☐
4. Were diagnostic tests or assessment methods and the results clearly described?	☐	☐	☐	☐
5. Was the intervention(s) or treatment procedure(s) clearly described?	☐	☐	☐	☐
6. Was the post-intervention clinical condition clearly described?	☐	☐	☐	☐
7. Were adverse events (harms) or unanticipated events identified and described?	☐	☐	☐	☐
8. Does the case report provide takeaway lessons?	☐	☐	☐	☐

Overall appraisal: Include ☐ Exclude ☐ Seek further info ☐

Comments (including reason for exclusion):

For peer review only

Table 1 Characteristics of case studies included in the review

Author, Year<br><br>Country<br><br>Purpose of article	Setting<br><br>Demographic characteristics	Focus of case study	Discussion
1 Ang et al 2009[148]<br><br>Singapore<br><br>Addresses the challenge of what should be done in the case of people with chronic conditions which impair their capacity	Emergency hospital care of a woman in her 40s with a diagnosis of schizoaffective disorder since her early 20s and with recent end stage renal failure	Ethical issues in EoLC for people lacking capacity to make decisions	Autonomy of a patient with regard to medical treatment, assessment of the patient's decisional capacity, and the process of deciding on the appropriate course to take in a patient without the mental capacity to give consent for required treatment in a potentially life-threatening situation<br><br>Legal frameworks, tools to assess capacity and the use of advanced ('Ulysses') contracts are all addressed
2 Badger and Ekham 2011[116]<br><br>USA<br><br>Ethical conflicts at the end of life for patients with personality disorder, substance misuse and self-harming behaviour	A case of a man in his 40s with long-term MH problems whose physical health declines (liver and respiratory failure) and whose care presents an ethical challenge to practitioners	Ethical issues in EoLC for people lacking capacity to make decisions	Decision-making for people lacking capacity is difficult in the absence of ADs and in the case of people with complex MH and substance misuse problems
3 Bakker 2000[109]	A residential service using DSA for older people with complex mental and	The use of the DSA approach, including in the example of people	The DSA approach is offered as a psychosocial counterbalance to more

For peer review only

Netherlands<br><br>Focuses on the PC of patients in a 'reactivation program', this being a service for older people with complex health problems	physical health problems, in which the case is a woman in her 90s	who end their lives in the DSA service, illustrating the phases of the approach in action	exclusively biomedically oriented approaches to care
4 Boyd et al 1997[117]<br><br>USA<br><br>The process of care for a hospice patient described as manipulating family, friends, and hospice staff during her EoLC	A hospice service for people at the EoL, with the case being a woman in her 50s with breast cancer who is described as having a pre-existing (but not clearly described) severe MH problem characterised by 'manipulative' behaviour	The challenges faced by hospice staff in providing care to someone perceived as being manipulative, and how these can be addressed using practices informed by psychodynamic thinking	Hospice staff can provide care for people with 'troubled personality' using psychodynamic approaches, including supporting ego functioning; supporting autonomy; setting limits; and assessing and managing staff emotional responses
5 Cabaret et al 2002[146]<br><br>France<br><br>The provision of EoLC in an inpatient psychiatric hospital setting	An inpatient psychiatric hospital, with the case being a man in his 40s with a diagnosis of schizophrenia and lung cancer	The provision of humane, palliative, care in a MH hospital	Inpatient MH staff are able to provide humane, dignified, care until the EoL for people with SMI
6 Candilis and Foti 1999[118]<br><br>USA	A case study beginning in an ED and moving to a MH unit, focused on the care of a woman in her 70s with cancer and psychosis	Decision-making in the context of EoLC and psychosis, where the patient's understanding and preferences are not clearly known	Differences in view can exist over the best course of action in cases where patients refuse treatment but do not elaborate supportive, palliative, care or more aggressive intervention

For peer review only

EoLC to someone with psychosis and cancer, where the patient's preferences are not clearly known			
7 Clements-Cortes 2004[143]<br><br>Canada<br><br>The use of music therapy in the emotional care of people who are at the EoL	A discussion on the value of music therapy, followed by three case studies all of people using an older people's PC inpatient service, of which two (women in their 70s) have pre-existing MH problems	The value of music in providing emotional support to people with terminal illnesses	Music therapy can facilitate emotional expression, including in people with SMI
8 Doron and Mendlovic 2008[147]<br><br>Israel<br><br>EoLC in inpatient MH wards	Two case studies of people, one in her 40s at her death and the other a woman whose age at death is not stated, cared for near to the EoL in inpatient MH settings, where both had a diagnosis of SMI and cancer	The challenges for both patients and staff of being cared for during a terminal illness in a MH inpatient setting	Information on the patient's condition and prognosis should be given in the context of their capacity to understand, decisions should be made by patients where possible or otherwise by clinicians and home is where people should die meaning that if this is a psychiatric ward staff should be supported to provide care for as long as possible
9 Feely et al 2013[119]<br><br>USA<br><br>EoLC in a hospice for people with borderline personality disorder	A case study of a woman in her 40s with a diagnosis of borderline personality disorder and terminal metastatic pancreatic cancer, being cared for in a hospice	The difficulties for staff in hospices of providing care for people with complex MH problems characterised by impulsive behaviour and difficulties in relationships	There is a lack of evidence to support interventions with people with borderline personality disorder at the EoL, but behavioural interventions should be tailored to the person based on the creation of genuine, respectful, relationships

10 Feldman and Petriyakoil 2006[120]<br><br>USA<br><br>PTSD at EoL	A case study of a male veteran in his 60s with heart failure and acute onset of PTSD symptoms.	Challenges of recurrence of latent PTSD symptoms seemingly triggered by the threat to life of terminal illness mimicking previous experiences.	Specific challenges in EoLC for people with PTSD may mean they experience psychological symptoms that have been dormant, become anxious and angry, avoid healthcare, distrust medical recommendations leading to nonadherence and refusal of treatment
11 Feldman et al 2014[110]<br><br>USA<br><br>The provision of PC to people with PTSD at the EoL	A re-presentation of a new model of care to people with a diagnosis of PTSD, either pre-existing or post-EoL diagnosis, illustrated by three case studies (men in their 60s) in diverse PC settings	An illustrated model of care for people with PTSD at the EoL, involving therapy over three distinct stages: Palliate Immediate Discomfort and Provide Social Supports; Provide Psychoeducation and Enhance Coping Skills; and Treat Specific Trauma Issues	The SPPC model can be used to support terminally ill patients with PTSD where conventional PTSD interventions are challenged by limited life expectancy, fatigue and other concerns
12 Feldman et al 2017[111]<br><br>USA<br><br>The provision of PC to people with PTSD at the EoL	A case of a male veteran in his 70s with a diagnosis of PTSD and multiple myeloma, admitted for inpatient hospice care at the EoL	How a staged approach to PTSD care is helpful for people at the EoL	A further illustration of how the SPPC model can be used to support terminally ill patients with PTSD where conventional PTSD interventions are challenged by limited life expectancy, fatigue and other concerns
13 Geppert et al 2011[121]<br><br>USA	A detailed case study of a man in his 50s with a pre-existing a diagnosis of schizoaffective disorder who has	The ethical issues around treatment when people have psychosis and lack decisional capacity	Psychosis raises ethical issues for EoLC as it undermines decisional capacity, raises questions about the use of proxies and additional questions about where care ought to

For peer review only

PC and capacity in the context of people with psychosis and terminal cancer	terminal laryngeal cancer and who is admitted to hospital		be provided (MH services, or ned of life services)
14 Gonzalez et al 2009[122]<br><br>USA<br><br>Hospice care of a man with breast cancer and bipolar disorder	A medical case study of a man in his 80s with complex health needs who receives inpatient care, and who has a diagnosis of bipolar disorder, dementia and breast cancer first treated 20 years previously	The complexity of providing care for people with multiple needs, in the context of family members also having significant health needs	Curative care is difficult to provide if treatment is not consistently provided and accepted, and PC is appropriate particularly when caregivers have health needs of their own
15 Griffith 2007[123]<br><br>USA<br><br>Palliative and EoLC for a man with heart and lung failure and schizophrenia [pt 1 of 2]	An extended case study of a man in his 60s, focusing on his schizophrenia diagnosis and his lung and heart disease and his care at home until death	The role of the psychiatrist in the provision of integrated care, including at the EoL, to people with SMI and multi-organ failure	Psychiatrists have an important part to play in treating people who have both severe MH and terminal physical health problems
16 Griffith 2007[124]<br><br>USA<br><br>Palliative and EoLC for a man with heart and lung failure and schizophrenia [pt 2 of 2]	An extended case study of a man in his 60s, focusing on his schizophrenia diagnosis and his lung and heart disease and his care at home until death	The role of the psychiatrist in the provision of integrated care, including at the EoL, to people with SMI and multi-organ failure	Psychiatrists should have cultural and religious competence and be able to work with families
17 Hill 2005[125]<br><br>USA	A case of a woman in her 80s described as having borderline personality disorder and hospice care at home	EoLC for people with a diagnosis of borderline personality disorder and complex physical health problems and family histories	Nurses should develop therapeutic relationships, have good supervision, be consistent in caregiving, and have

For peer review only

Hospice at home care for a person with borderline personality disorder			good working relationships with families and psychiatrists
18 Irwin et al 2014[26]<br><br>USA<br><br>Cancer care, including to EoL, for people with schizophrenia	A detailed review of literature followed by a case study of a woman in her 60s with a diagnosis of paranoid schizophrenia and terminal cancer, whose care moves from home to inpatient hospice	Health inequalities for people with a diagnosis of schizophrenia and cancer	Practitioners need improved understanding of health disparities experienced by people with a diagnosis of schizophrenia and cancer, research is needed to examine factors influencing survival and quality of life and better integration is required between oncology and psychiatric services
19 Kadri et al 2014[144]<br><br>Canada<br><br>Care for a person with schizophrenia and advanced kidney disease who lacks capacity and resists treatment	A case of a woman in her 50s with a diagnosis of schizophrenia and advanced chronic kidney disease admitted to hospital as an emergency and who refuses dialysis, then receives supportive care	Ethical issues in the case of people with SMI and who are unable to make decisions, but who actively resist treatment	Two ethical issues are highlighted: the lack of acceptance of treatment, and the risk of doing immediate harm if treatment is continued nonetheless
20. Kennedy et al 2013[114]<br><br>UK<br><br>Homelessness and EoLC	A case study of a woman in her 50s with diagnosis of schizophrenia and cervical cancer living semi-independently in a one bedroomed flat.	Treatment refusal in someone with severe mental illness who has the capacity to make decisions and her determination to return home to live.	Providing care for a person in their own home involving multiple professional teams in support of her autonomous decision to remain out of hospital and considering her refusal of active treatment
21 Kunkel et al 1997[126]<br><br>USA	A discussion of denial of cancer followed by five illustrative cases:<br><br>a woman in her 30s with a diagnosis of psychosis and breast cancer, who	A review of factors associated with non-compliance in people with cancer, specifically psychoses and cognitive impairment Both are shown to play a role in delayed help-seeking and non-	Key issues affecting cancer treatment are competence, the place for legal intervention, ambivalence towards health care providers and the

Denial of cancer and non-compliance in treatment	misses treatment and dies following an emergency admission;<br><br>a woman in her 50s with presumed 'organic personality syndrome' and squamous cell carcinoma who was psychotic and who died in hospital;<br><br>-a man in his 70s with presumptive pancreatic cancer and pre-existing organic delusional disorder and a diagnosis of major depression with psychosis, who had home hospice care;<br><br>a woman in her 70s with a diagnosis of dementia and breast cancer;<br><br>a woman in her 70s with a diagnosis of schizophrenia, superimposed dementia and lung cancer	compliance with cancer, and EoL, care	importance of early psychiatric intervention
22 Levin and Feldman 1983[127]<br><br>USA (the authors are based in South Africa)<br><br>Ethical issues surrounding the care of people with psychosis and terminal cancer	A case of a woman in her 30s with a diagnosis of schizophrenia and terminal breast cancer who is described as wanting to leave hospital without medical care following the treatment of an acute episode of her mental illness	A discussion of ethical issues associated with the care of people with SMI and terminal illnesses when treatment is refused	People have a right to refuse high-powered medicine at the EoL
23 Lopez et al 2010[128]	The case of a woman in her 30s with a diagnosis of chronic anorexia	Discussion of ethical and legal issues associated with caring for a person with a psychiatric condition who is not	Refusal of treatment can be irrational but must be weighed against workers obligations and duty of care and the

For peer review only

USA<br><br>Explores how medical futility and principles of palliation may contribute to the management of treatment refractory anorexia nervosa	nervosa, treated unsuccessfully for several years	helped by treatment where the condition ultimately leads to her death	autonomy of the individual. Two broad options are considered, 1) involuntary treatment, or 2) attempting to motivate the person until such time as they are willing to engage in treatment. The first option may have kept her alive in the short term but seemed unlikely to reverse the underlying condition
24 Maloney 2014[115]<br><br>USA [blog]<br><br>https://www.thejournal.ie/readme/bipolar-ii-disorder-denial-mental-health-1467162-May2014<br><br>A wife's experiences of her husband's bipolar disorder and terminal cancer	A personal blogpost recording a woman's experiences of her husband's diagnosis with a diagnosis of bipolar disorder, his subsequent terminal cancer and his death at home	A first-hand, narrative, account of long-term experiences ending in the death of a loved one	SMI changes people's lives, and can cause people to lose touch with reality People affected (patients and families) need support
25 Mason and Bowman 2018[112]<br><br>Country unknown (conference abstract)<br><br>The challenges of providing cancer care at the EoL to people with SMI and fluctuating capacity	A conference proceedings abstract describing the care of a man in his 70s with a diagnosis of schizophrenia and fluctuating capacity admitted to hospital for investigations and care into prostate cancer and metastases	An account of the challenges for people with SMI and fluctuating capacity in the context of EoL cancer care	Determining capacity in the context of SMI and acute physical illness is difficult, where standardisation in capacity assessment may help
26 McCasland 2007[129]<br><br>USA	A case of a woman in her 50s with a diagnosis of schizoaffective disorder, alcohol problems, liver damage and	Hospice nurses need better preparation to look after people at the EoL who also have SMI	Communication skills are important for hospice nurses caring for people with SMI at the EoL, and hospice nurses

For peer review only

The care and treatment of people with SMI and cancer, including at the EoL	breast cancer who is cared for in the community until dying in a hospice		needs more training to care for this group of people
27 McKenna et al 1994[130]<br><br>USA<br><br>The treatment with clozapine of people with SMI and cancer, where treatment also includes antineoplastic medication	A letter to the editor describing a man in his 40s with a diagnosis of schizophrenia and lung cancer with a poor prognosis, treated with clozapine, whose care was transferred from a MH to a medical care institution	The combination of clozapine with anti-tumour drugs	Guidance is needed on the combination of clozapine (an anti-psychotic) with anti-cancer drugs
28 Mogg and Bartlett 2005[140]<br><br>UK<br><br>The care and treatment of a man with treatment-resistant psychosis and life-threatening renal failure	The case is a man in his forties with a 30-year history of psychosis and multiple admissions to MH hospital, now discovered to have life-threatening renal disease requiring regular dialysis	Different approaches to the assessment of capacity (outcome, status, functional), set in the context of the legal framework	Capacity can fluctuate, and 'best interests' need to be considered which can mean guidance needing to be sought from the courts
29 Moini and Levinson 2009[131]<br><br>USA<br><br>The care and treatment of people with simple schizophrenia and medical conditions	A case of a woman in her 40s with a diagnosis of simple schizophrenia who is lost to services following diagnosis of breast cancer with metastases, and who is located at a later point at the terminal stage of her illness	Approaches to working with people with a diagnosis of simple schizophrenia and medical problems	Proactive care is needed so that people stay in touch with services, though people with a diagnosis of simple schizophrenia are also likely to have capacity to decide on their courses of treatment
30 Monga et al 2015[132]<br><br>USA	The case is of a man in his 70s with a diagnosis of schizophrenia, treated with clozapine, who continues his antipsychotic medication whilst having	The use of clozapine as an atypical antipsychotic alongside the use of chemotherapy	There is limited information to guide management of clozapine treatment during chemotherapy, and close

For peer review only

The use of clozapine in people with psychosis who are also having chemotherapy	hospice care and treatment for oesophageal cancer		cooperation between psychiatrists and oncologists is needed
31 Muhtaseb et al 2001[141]<br><br>UK<br><br>The treatment of people with SMI and advanced, terminal, cancer	The case is of a man in his 70s with a diagnosis of schizophrenia and advanced basal cell carcinoma, who refuses active intervention	Treatment of advanced, life-threatening, cancer in people with SMI who refuse active intervention	Consent must be freely given, and people (including people with SMI) should be considered able to decide for themselves
32 O'Neill et al 1994[142]<br><br>UK<br><br>Hospice care for woman with anorexia nervosa	The case of a woman in her 20s with a diagnosis of anorexia admitted to a hospice in poor physical condition after 7 years of anorexia nervosa that was not helped by treatment.	Discusses the treatment and rapid decline to eventual death of a young woman from the complications associated with anorexia nervosa.	Recognises that psychiatric units do not have relevant expertise for providing complex physical healthcare. Says that her condition was identified as incurable by the psychiatric team and when the patient and her family accepted this she was then able to access appropriate care. Says hospice staff are familiar with other EoLC and that this largely worked for this woman helped by an excellent referral letter (no clue how this is judged) and ongoing support from the psychiatrist. They conclude that there is a role for carefully selected non-cancer patients to be cared for in SPC centres
33 Picot et al 2015[15]<br><br>Australia	The case is of a woman in her 40s who has a diagnosis of bipolar disorder and metastatic breast cancer,	An illustrative case showcasing the work of nurse practitioners involved in the IMhPaCT programme, which is	People with SMI at the EoL face additional problems of isolation, declining physical abilities, pain, and disintegrating selfhood Collaboration

For peer review only

The IMhPaCT, designed to improve care for people with SMI and life-limiting illnesses	who receives integrated MH and PC at home before dying in hospital	designed to bring together MH and PC for people with SMI	across specialities can enhance outcomes, and MH nurses have a role to play
34 Rice et al 2012[133]<br><br>USA<br><br>Patient-provider communication in EoLC	The case of a male veteran in his 50s with renal disease and a diagnosis of schizophrenia who refuses dialysis treatment.	The case provides an example of man refusing treatment in line with previous history and is found to lack capacity to decide in his best interests. Involvement of his sister who is ill-prepared to take on proxy decisions	Discussion hinges on challenges of enforcing physical treatments on a person who is refusing, handling autonomy versus coercion, recognition of need for building therapeutic alliances with the person and their surrogates
35 Rodriguez-Mayoral 2018[113]<br><br>Mexico<br><br>Integrating MH and PC for people with SMI and cancer	A short conference abstract describing a woman in her 70s with a diagnosis of bipolar disorder and advanced, untreated, colon cancer from which she dies	The case briefly describes how the woman was cared for until her death, free form symptoms of her mental illness	Cancer care regardless of disease stage should be integrated with palliative and MH care to control symptoms and quality of life and death
36 Romm et al 2009[134]<br><br>USA<br><br>Managing cancer care for people using inpatient MH services	The case is a man in his 40s with a diagnosis of schizophrenia and osteosarcoma for which amputation was indicated, and who refused surgical treatment but who was judged to lack capacity His mother assumed decision-making responsibility and concurred with her son, and PC was initiated	Treatment of people with acute MH problems and life-threatening cancer, and patients' capacity to understand and made decisions	Psychiatrists have responsibilities to represent the interests of people who are 'mentally compromised', including those who are also irreversibly medically compromised Collaboration with PC practitioners is important, and whilst SMI can impair capacity this is not necessarily so
37 Shah et al 2008[135]<br><br>USA	The case is a homeless man in his 60s with a diagnosis of schizophrenia and neglected basal cell carcinoma, and who refuses treatment in the	Informed consent is key to the provision of cancer treatment, and in	In the absence of being able to give informed consent interprofessional

For peer review only

The treatment of people with SMI and advanced, terminal, cancer	context of a lack of capacity and is then treated for his mental illness	cases where people lack capacity additional care needs to take	teams need to be involved, along with independent ethics committees
38 Stecker 1993[136]<br>USA<br>Caring for young people with life-threatening cancer on an acute psychiatric ward	The case is of a man in his 20s with presumed bipolar disorder and sarcoma who is admitted to an inpatient psychiatric unit and who causes staff to struggle with the acceptance of death	Staff in MH wards do not routinely care for young people at the EoL, and this case challenged staff	MH nurses need to be able to identify feelings preventing them from working therapeutically with patients
39 Steves and Williams 2016[137]<br>USA<br>EoLC for people with terminal illnesses and SMI	A very brief case of a man, of unknown age, who has a diagnosis of schizophrenia and terminal lung cancer but who has capacity and refuses treatment prior to his death	Services for people with SMI at the EoL are in short supply, MH nurses need training in EoLC, attention needs to be paid to the environment, communication needs to be effective, caregivers needs to be supported, family members may be resistant and other inpatients need to be supported following a death	Investments are needed in services and staff to care for increasing numbers of people with SMI who also have terminal medical conditions
40 Terpstra et al 2014[138]<br>USA<br>EoL hospice care for people with SMI	The case is of a man in his 60s with a diagnosis of schizophrenia living in adult foster care who reports physical symptoms and is found to have bladder cancer and brain masses, moves to an open hospice and whose care and treatment is then described	The complexity of providing hospice care for people with SMI and terminal cancer, and the role of nurses	People with schizophrenia diagnoses are often medically undertreated, have shortened life expectancies, and have care which is challenged by their placements, poor communication and symptom management

For peer review only

41 Thomson and Henry 2012[70]<br><br>USA<br><br>The challenge for oncology nurses of caring for people with SMI and cancer	A paper on oncology nursing for people with pre-existing SMI, including a series of brief case studies: a woman in her 30s with a diagnosis of major depression and then breast cancer; a man in his 40s with a diagnosis of bipolar disorder and advanced pancreatic cancer and a woman in her 20s with a diagnosis of schizophrenia and breast cancer	Care and treatment of people with pre-existing SMI at the EoL, with a particular emphasis on understanding mental illnesses and its treatment	Medical professionals need to be aware of premature mortality in people with SMI, and need to develop skills and knowledge in caring for people with SMI as an underserved group Practice is advancing in the areas of drug treatments, case management, family work and social support
42 Webber 2012[145]<br><br>Canada<br><br>Caring for a man with SMI in his supported home prior to hospice care	A brief case study of a man of unknown age who has a diagnosis of schizophrenia and metastatic gastric cancer and who was cared for in his supported home for as long as possible until being transferred to a hospice, where he died	Pain control, control over of levels of intervention, control over maintaining meaningful, relationships, outcomes of lost autonomy, lack of resources, restoring justice for patients with SMI and terminal illness, and innovation are all discussed	Autonomy has been diminished for people with SMI and palliative illness, yet people with SMI at the life want the same things as everyone else Services and care need to improve,

Key: DSA: dynamic system analysis; EoL: end of life; EoLC: end of life care: IMhPaCT: Integrated Mental Health and Palliative Care Task; PTSD: post-traumatic stress disorder; SMI: severe mental illness; SPC: specialist palliative care: SPPC: stepwise psychosocial palliative care

For peer review only

Table 2 Critical appraisal scores

For peer review only

Citation	Q1	Q2	Q3	Q4	Q5	Q6	Q7	Q8	Score
1. Ang et al 2009[148]	Y	Y	Y	N	Y	Y	Y	Y	7/8
2. Badger and Ekham 2011[116]	Y	Y	Y	N	Y	Y	Y	Y	7/8
3. Bakker 2000[109]	Y	Y	Y	N	Y	Y	Y	Y	7/8
4. Boyd et al 1997[117]	Y	Y	Y	N	Y	Y	Y	Y	7/8
5. Cabaret et al 2002[146]	Y	Y	Y	N	Y	Y	Y	Y	7/8
6. Candilis and Foti 1999[118]	Y	Y	Y	N	Y	Y	Y	Y	7/8
7. Clements-Cortes 2004[143]	Y	Y	Y	N	Y	Y	N	Y	6/8
8. Doron and Mendlovic 2008[147]	Y	Y	Y	Y	Y	Y	Y	Y	8/8
9. Feely et al 2013[119]	Y	Y	Y	N	Y	Y	Y	Y	7/8
10. Feldman and Petriyakoil 2006[120]	Y	UC	Y	N	N	Y	Y	N	4/8
11. Feldman et al 2014[110]	Y	Y	Y	N	Y	Y	Y	Y	7/8
12. Feldman et al 2017[111]	Y	Y	Y	N	Y	Y	Y	Y	7/8
13. Geppert et al 2011[121]	Y	Y	Y	Y	Y	Y	Y	Y	8/8
14. Gonzelez et al 2009[122]	Y	Y	Y	Y	Y	Y	Y	Y	8/8
15. Griffith 2007[123]	Y	Y	N	Y	Y	Y	Y	Y	7/8
16. Griffith 2007[124]	Y	Y	N	Y	Y	Y	Y	Y	7/8
17. Hill 2005[125]	Y	Y	Y	Y	Y	Y	Y	Y	8/8
18. Irwin et al 2014[26]	Y	Y	Y	Y	Y	Y	Y	Y	8/8
19. Kadri et al 2018[144]	Y	N	Y	N	Y	N	Y	Y	5/8
20. Kennedy et al 2013[114]	Y	UC	Y	N	Y	Y	Y	Y	6/8
21. Kunkel et al 1997[126]	Y	Y	Y	Y	Y	Y	Y	Y	8/8
22. Levin and Feldman 1983[127]	Y	Y	Y	N	Y	N	Y	Y	6/8
23. Lopez et al 2010[128]	Y	Y	Y	N	Y	Y	N	Y	6/8
24. Maloney et al 2014[115]	Y	Y	Y	N	Y	Y	Y	Y	7/8
25. Mason and Bowman 2018[112]	Y	Y	Y	Y	Y	Y	Y	Y	8/8

For peer review only

26. McCasland 2007[129]	Y	Y	Y	Y	Y	Y	Y	Y	8/8
27. McKenna et al 1994[130]	Y	N	UC	N	Y	N	Y	Y	4/8
28. Mogg and Bartlett 2005[140]	Y	N	N	N	Y	N	N	Y	2/8
29. Moini and Levenson 2009[131]	Y	Y	Y	N	N	N	Y	Y	5/8
30. Monga et al 2015[132]	Y	Y	Y	Y	Y	Y	Y	Y	8/8
31. Muhtaseb et al 2001[141]	Y	Y	Y	Y	Y	Y	Y	Y	8/8
32. O'Neill et al 1994[142]	Y	N	Y	N	Y	N	N	N	2/8
33. Picot et al 2015[15]	Y	Y	Y	Y	Y	Y	Y	Y	8/8
34. Rice et al 2012[133]	Y	N	Y	N	Y	Y	Y	Y	6/8
35. Rodriguez-Mayoral 2018[113]	Y	N	N	N	N	Y	Y	Y	5/8
36. Romm et al 2009[134]	Y	N	Y	N	N	N	Y	Y	3/8
37. Shah et al 2008[135]	Y	N	Y	Y	Y	Y	Y	Y	7/8
38. Stecker 1993[136]	Y	N	Y	N	N	N	Y	Y	4/8
39. Steves and Willliams 2016[137]	Y	N	N	N	Y	Y	Y	Y	4/8
40. Terpstra et al 2014[138]	Y	Y	Y	Y	Y	Y	Y	Y	8/8
41.Thomson and Henry 2012[139]	Y	Y	N	N	N	N	N	Y	2/8
42. Webber 2012[145]	Y	N	N	N	N	Y	Y	Y	3/8

Key: Y=Yes, N=No, UC=unclear

For peer review only
